# Supplementary material for: Nuclear paxillin functions as a molecular switch for alternative splicing in neurons during a critical period of brain development
Source: EMBO J. 2025 Sep 9;44(21):5965–92. doi: 10.1038/s44318-025-00560-8 (PMC12583701; doi:10.1038/s44318-025-00560-8)
Supplement: Supplementary file 1 — Appendix [file 44318_2025_560_MOESM1_ESM.pdf]

Appendix for: "Nuclear paxillin functions as a molecular switch for alternative splicing in neurons during a critical period of brain development"

Table of contents:

Appendix Figure S1	1-2
Appendix Figure S2	3-4
Appendix Figure S3	5
Appendix Figure S4	6
Appendix Figure S5	7
Appendix Figure S6	8-9
Appendix Figure S7	10
Appendix Figure S8	11
Appendix Figure S9	12-13

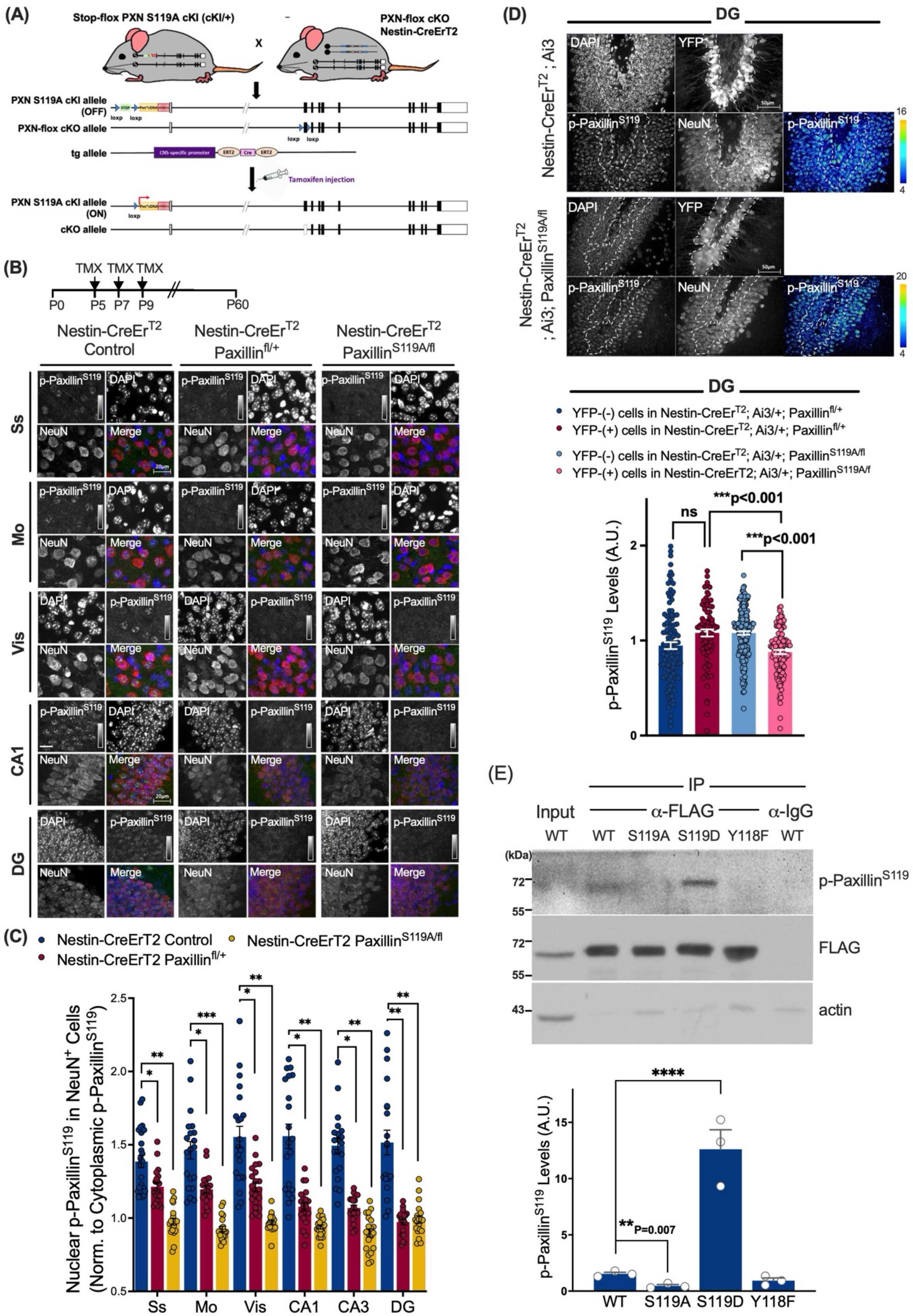
(A)
Stop-flox PXN S119A cKI (cKI/+)
PXN-flox cKO
Nestin-CreErT2
X
PXN S119A cKI allele (OFF)
PXN-flox cKO allele
loxp
tg allele
CNS-specific promoter
ERT2
Cre
Tamoxifen injection
PXN S119A cKI allele (ON)
cKO allele
(B)
TMX TMX TMX
P0 P5 P7 P9 P60
Nestin-CreErT2 Control
Nestin-CreErT2 Paxillinfl/+
Nestin-CreErT2 PaxillinS119A/fl
Ss
Mo
Vis
CA1
DG
p-PaxillinS119
DAPI
NeuN
Merge
20μm
(C)
Nestin-CreErT2 Control
Nestin-CreErT2 Paxillinfl/+
Nestin-CreErT2 PaxillinS119A/fl
Nuclear p-PaxillinS119 in NeuN+ Cells (Norm. to Cytoplasmic p-PaxillinS119)
Ss
Mo
Vis
CA1
CA3
DG
(D)
DG
Nestin-CreErT2 ; Ai3
Nestin-CreErT2 ; Ai3; PaxillinS119A/fl
DAPI
YFP
p-PaxillinS119
NeuN
50μm
YFP-(-) cells in Nestin-CreErT2; Ai3/+; Paxillinfl/+
YFP-(+) cells in Nestin-CreErT2; Ai3/+; Paxillinfl/+
YFP-(-) cells in Nestin-CreErT2; Ai3/+; PaxillinS119A/fl
YFP-(+) cells in Nestin-CreErT2; Ai3/+; PaxillinS119A/f
ns
***p<0.001
***p<0.001
p-PaxillinS119 Levels (A.U.)
(E)
IP
Input
α-FLAG
α-IgG
WT
WT
S119A
S119D
Y118F
WT
(kDa)
p-PaxillinS119
FLAG
actin
**P=0.007
****
p-PaxillinS119 Levels (A.U.)
WT
S119A
S119D
Y118F

Appendix Figure S1. Validation of p-Paxillin^{S119} Antibody Specificity.

(A) Schematic illustrating engineering of doubly heterozygous, conditional paxillin^{S119A} knock-in (cKI) and paxillin-floxed (cKO) mouse lines.

(B) Images of brain sections obtained from neuronal paxillin^{S119A} cKI or paxillin cKO mice and control littermates stained at P65 with DAPI and with antibodies against p-Paxillin S119 and NeuN. **(C)** Histogram summarizing average p-Paxillin^{S119} intensity ± SEM, normalized to E17 in different brain regions from experiments similar to those shown in **(B)** (n = 3 cortices per brain area; >20 NeuN+ cells per group; *p < 0.05, **p < 0.01, ***p < 0.001 compared to controls, one-way ANOVA followed by Dunnett's Multiple Comparison test). Ss: somatosensory cortex. Mo: motor cortex. Vis: visual cortex. DG: dentate gyrus.

(D) Similar to **(B&C)**, except Ai3^{+}NeuN^{+} dentate gyrus granule cells were selected for quantification. Data represents means ± SEM (normalized to E17; n = 3 cortices per brain area; 79-181 Ai3^{+}NeuN^{+} cells per group; *p < 0.05, **p < 0.01, ***p < 0.001 compared to controls, one-way ANOVA followed by Dunnett's Multiple Comparison test). In some right panels, p-Paxillin^{S119} staining intensity is indicated by maps on a linear scale.

(E) Western blots of FLAG immunoprecipitates from Neuro2A cells transfected with FLAG-tagged WT or indicated paxillin mutants, based on immunoblotting with antibodies against phospho-paxillin^{S119} and FLAG. Histograms summarize quantitative measurements of mean intensity ± SEM of phospho-paxillin^{S119} (n = 3 independent experiments; **p < 0.01, ****p < 0.0001 compared to WT control group by multiple unpaired t-test).

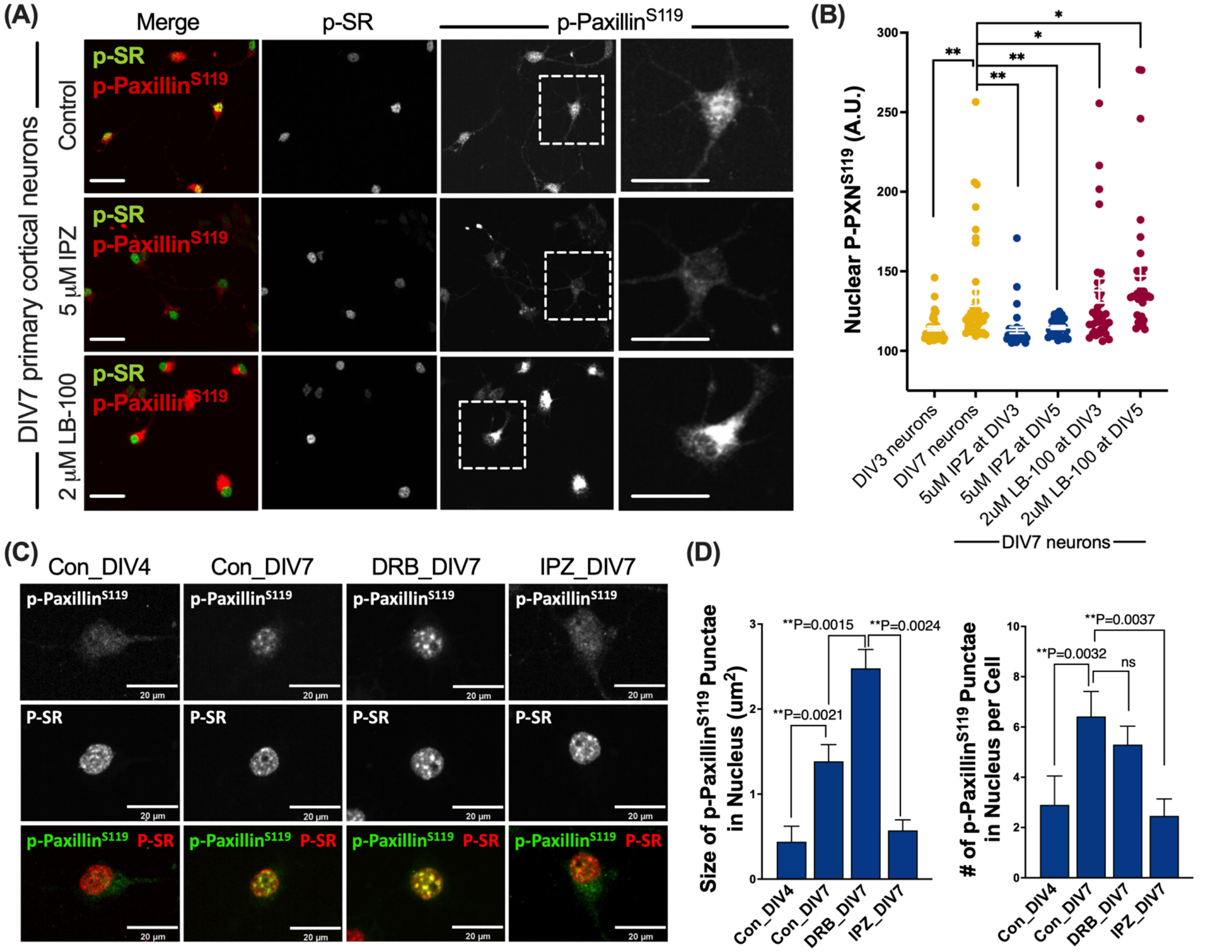

Appendix Figure S2. p-PaxillinS119 Localization to Nuclear Speckles in DIV7 Neurons is Dependent on Active Nuclear Import.

(A & B) Nuclear importin activity is required for p-Paxillin^{S119} nuclear localization in DIV7 neurons. Representative images of DIV 7 rat primary neuronal cultures treated with the importin inhibitor IPZ (5 µM) or the protein phosphatase 2A inhibitor LB-100 (2 µM), stained with antibodies against p-Paxillin^{S119} and the nuclear speckle marker, i.e., a collection of phosphorylated SR splicing factors (p-SR), as indicated. Scale bar, 20 µm. **(B)** Plot summarizing levels ± SEM of nuclear p-Paxillin^{S119} in treatment conditions shown in **(A)** (n = 3 independent cultures; *p < 0.05, **p < 0.01 compared to untreated DIV7 neurons, multiple t-tests)**.**

(C & D) Similar to (A&B), except transcription inhibitor 5,6-Dichloro-1-beta-D-ribofuranosylbenzimidazole (DRB) was used to treat DIV 7 neurons. Note that both nuclear speckles and p-Paxillin S119 punctae are enlarged following DRB treatment. Data represents average size ± SEM (n = 3 independent cultures; 30-40 cells per group; **p < 0.01 compared to untreated DIV7 neurons by multiple t-test; left panel) and number ± SEM (n =30-40 cells from 3

independent experiments; ns, non-significant; **$p < 0.01$ compared to untreated DIV7 neurons; right panel) of p-Paxillin^{S119} punctae in the nucleus.

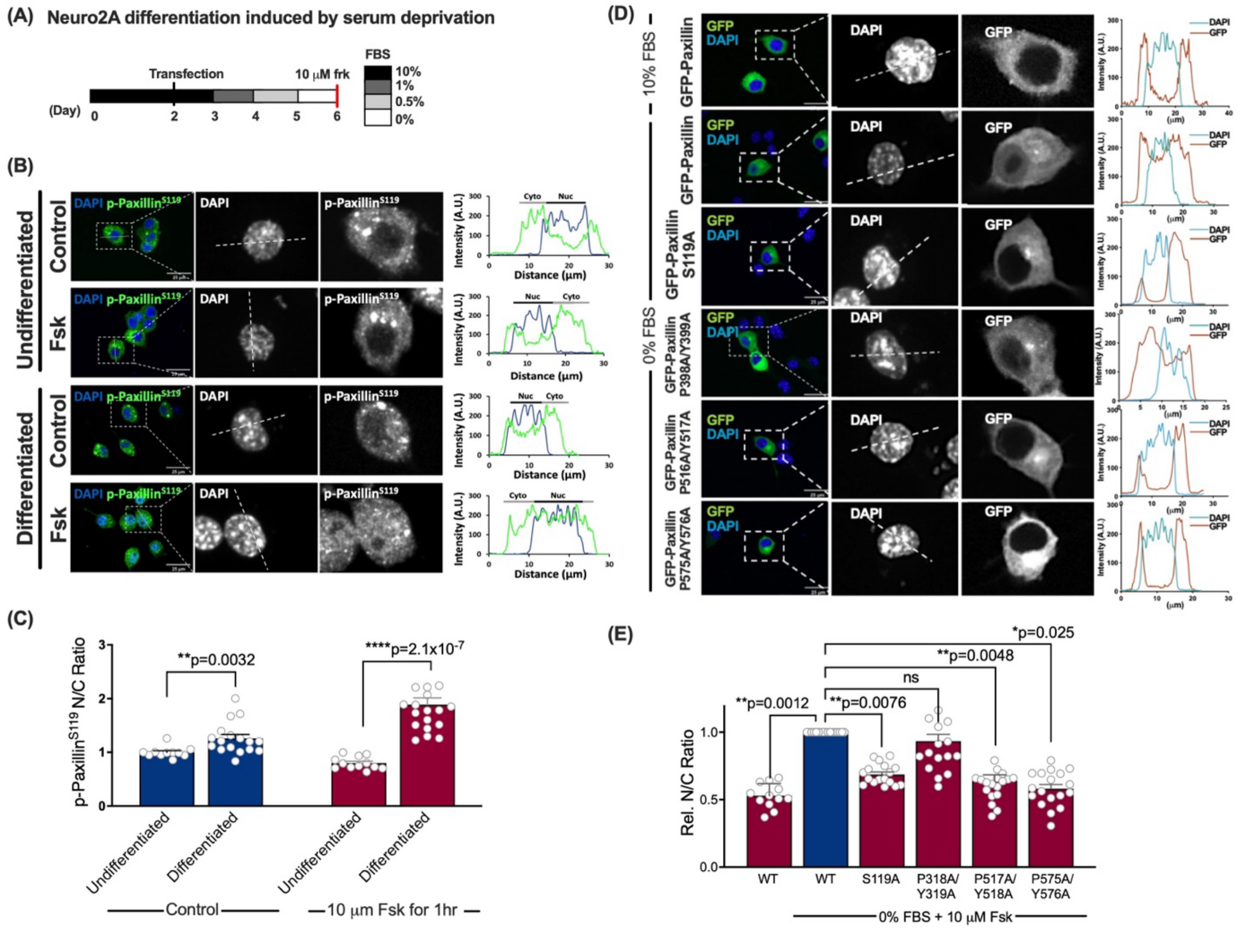

Appendix Figure S3. S119 Phosphorylation and the PY-NLS Motif Facilitate Paxillin Nuclear Translocation.

(A) Schematic of the serial serum-deprivation procedure used to induce Neuro2A cell differentiation.

(B) Images of undifferentiated and differentiated Neuro2A cells treated with or without 10 μM forskolin (Fsk), and stained with DAPI and antibodies against endogenous p-Paxillin^{S119}.

(C) Histograms show the increased nuclear-to-cytoplasmic (N/C) ratio ± SEM (n = 3 independent experiments; 15–20 cells per condition) of p-Paxillin(S119) in undifferentiated versus differentiated Neuro2A cells, with or without forskolin treatment (**p < 0.01, ****p < 0.0001 compared to undifferentiated cells, multiple t-tests).

(D) Similar to B & C, except cells were transfected with plasmid encoding GFP fused with either WT or indicated paxillin variants. Data represents mean ± SEM (n = 3 independent experiments; 15-20 cells each group, ns, non-significant, *p < 0.05, **p < 0.01; multiple t-tests).

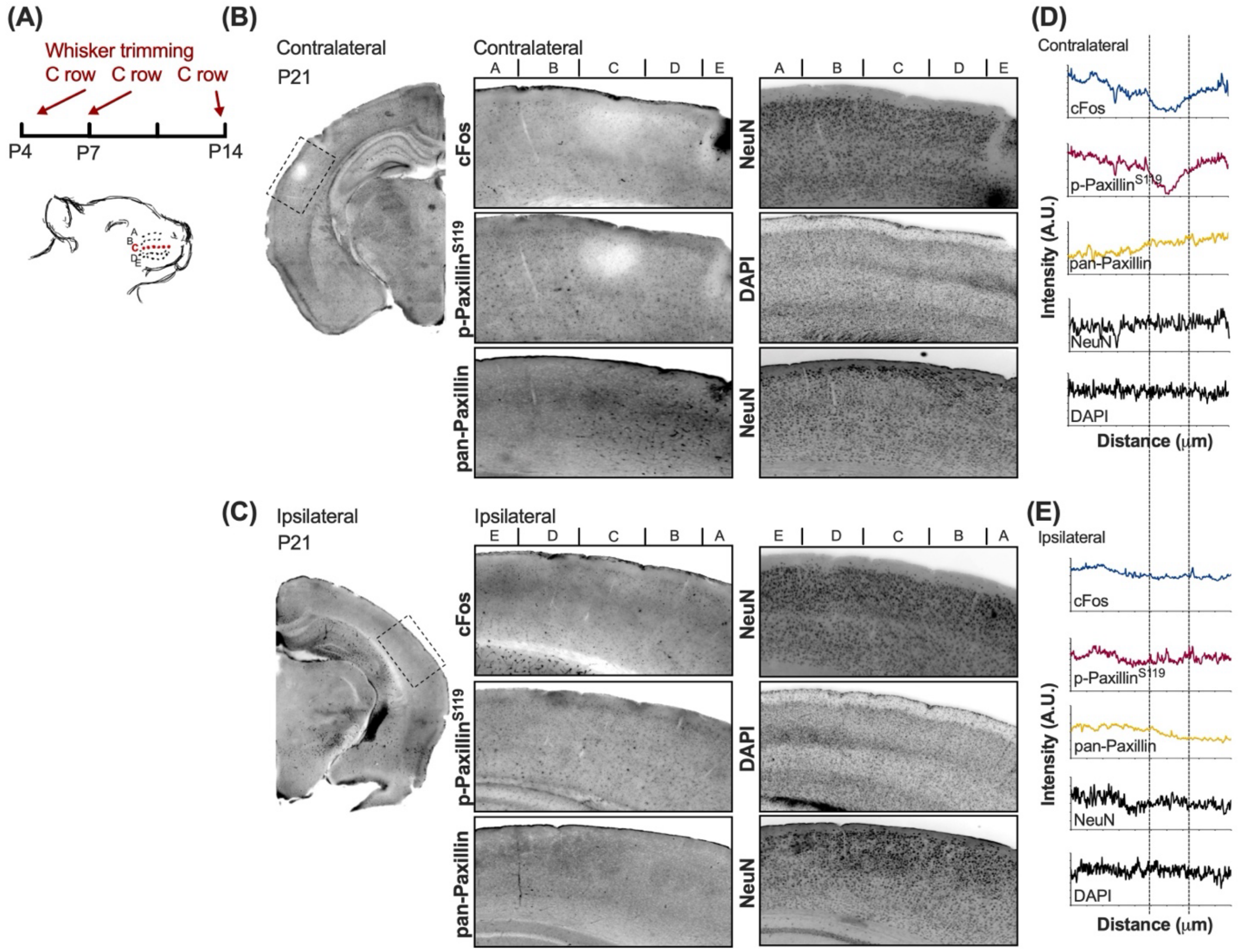

Appendix S4. Reduced Paxillin S119 Phosphorylation Coincides with Decreased c-Fos Expression in the Barrel Cortex Following Whisker Deprivation.

(A) Schematic of the whisker-trimming timeline, in which all of C-row was plucked on indicated days.

(B–E) Representative fluorescent immunohistochemical images **(B & C)** and corresponding intensity profiles **(D, E)** from the barrel cortex of P21 mice subjected to whisker deprivation. Note that in the hemisphere contralateral to trimmed whiskers **(B & D)**, p-Paxillin^{S119} and c-Fos levels are reduced in the C-column, while no reduction is observed in pan-Paxillin levels or in the ipsilateral hemisphere **(C & E)**.

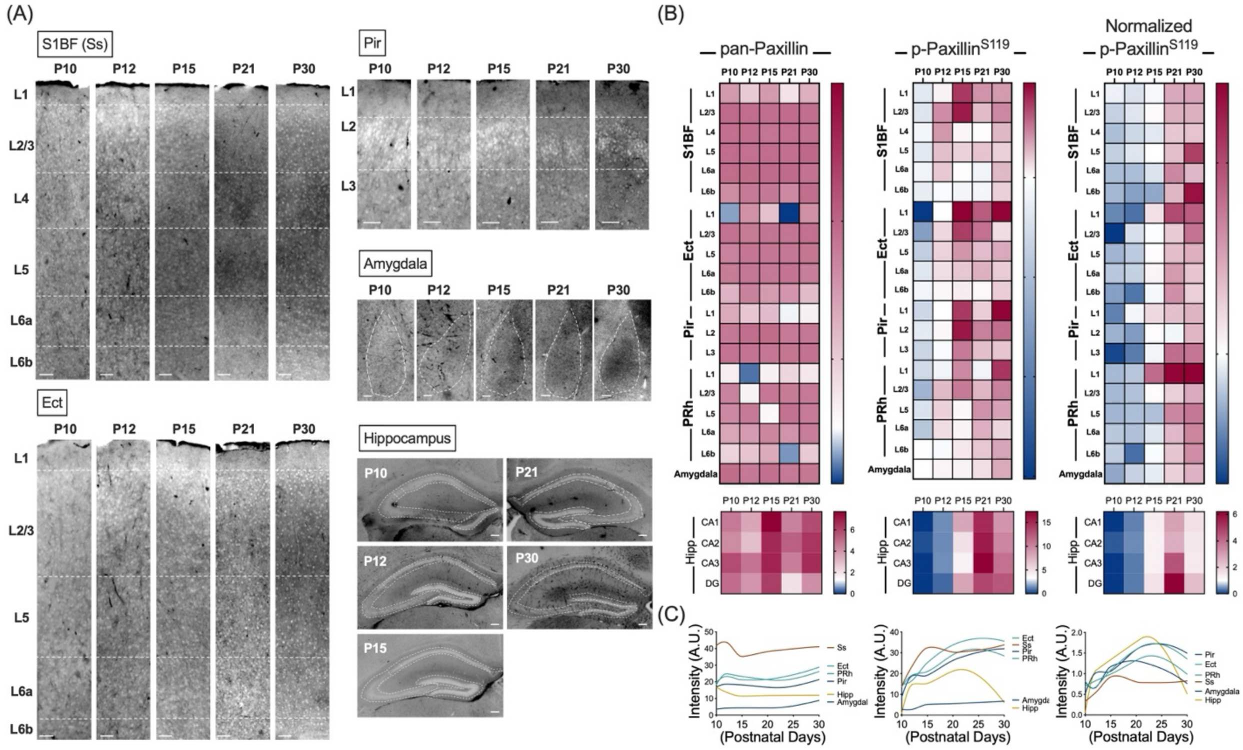

Appendix Figure S5. Expression Patterns of pan-Paxillin and p-Paxillin^{S119} in the Developing Mouse Brain.

(A) Immunohistochemistry of coronal sections of mouse brain showing pan-Paxillin staining at indicated postnatal days. Scar bar, 50 μm. **(B)** Heatmaps (n = 3 cortices per brain regions) showing quantification of staining intensity for pan-Paxillin, p-Paxillin^{S119}, and normalized p-Paxillin^{S119} (p-Paxillin^{S119} / pan-Paxillin) of in indicated brain regions, at indicated postnatal days. S1BF/Ss,somatosensory barrel field; Pir, piriform cortex; Ect, ectorhinal cortex; PRh, perirhinal cortex**. (C)** Akima spline fits of pan-Paxillin, p-Paxillin^{S119}, and normalized p-Paxillin^{S119}. Traces (n = 3 cortices per brain area) show staggered peak expression of p-Paxillin^{S119}, but not pan-Paxillin, across brain regions, with intensity maxima at P15 (red), P21 (yellow), P25 (green-blue), and P30 (dark blue).

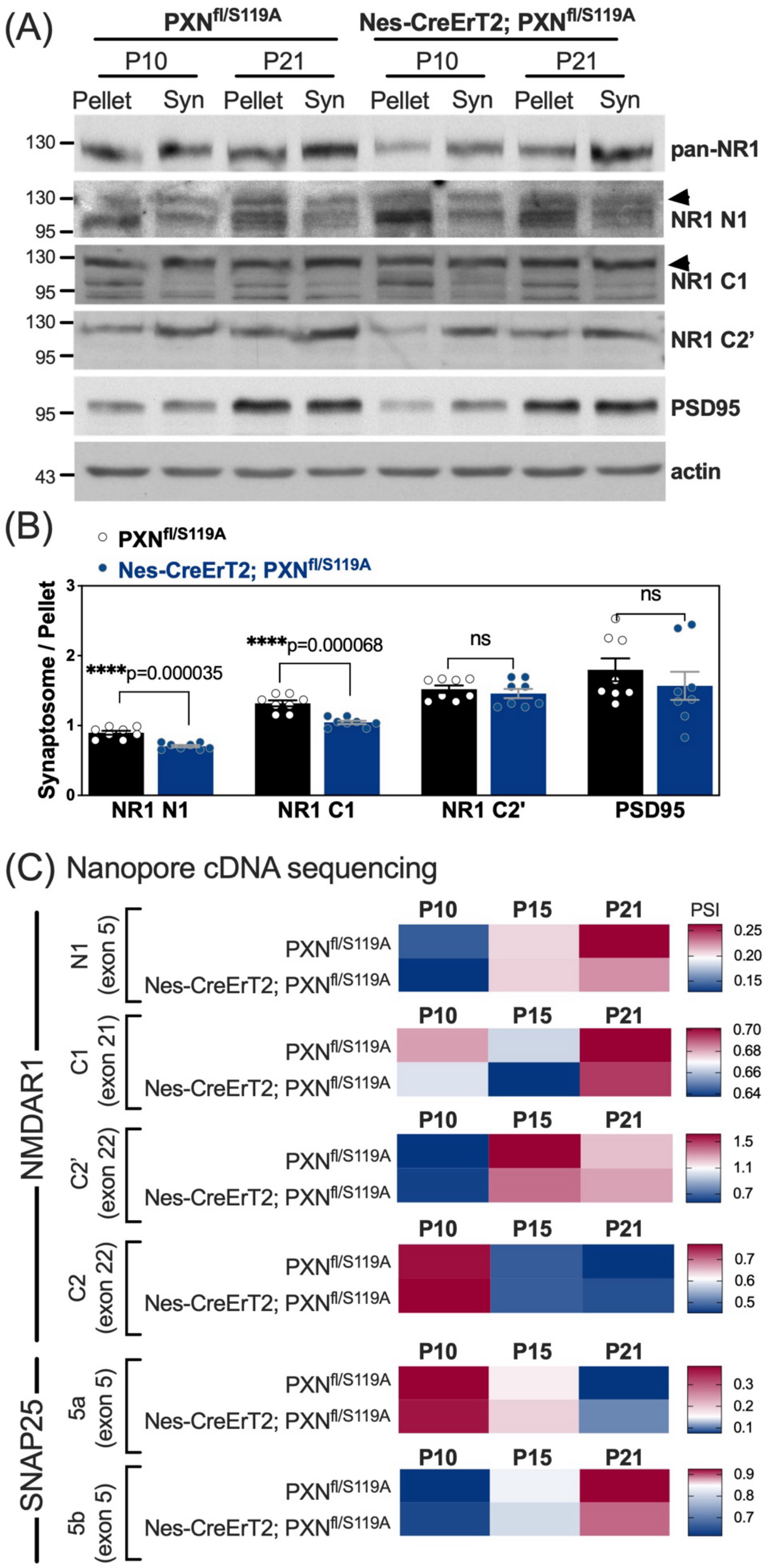
(A)
PXN fl/S119A
Nes-CreErT2; PXN fl/S119A
P10
P21
Pellet
Syn
130
95
43
pan-NR1
NR1 N1
NR1 C1
NR1 C2'
PSD95
actin
(B)
PXN fl/S119A
Nes-CreErT2; PXN fl/S119A
Synaptosome / Pellet
****p=0.000035
****p=0.000068
ns
NR1 N1
NR1 C1
NR1 C2'
PSD95
(C) Nanopore cDNA sequencing
P10
P15
P21
PSI
NMDAR1
SNAP25
N1 (exon 5)
C1 (exon 21)
C2' (exon 22)
C2 (exon 22)
5a (exon 5)
5b (exon 5)

Appendix Figure S6. Loss of Paxillin S119 Phosphorylation Alters NMDAR1 Splicing and Impacts Isoform Enrichment in Synaptosomes.

(A) Western blotting of crude synaptosome (Syn) and membrane pellet fractions from hippocampal tissue of Nes-CreErT2; $PXN^{fl/S119A}$ mice and littermate controls ($PXN^{fl/S119A}$) at indicated postnatal days. Blots were probed with antibodies recognizing total NMDAR1 ("pan-NR1"), splice variants containing the N1, C1, and/or C2' domains, along with PSD95 and actin as loading controls.

(B) Quantification of synaptosome-to-pellet (Syn/Pellet) ratios for the indicated NR1 variants and PSD95. Data represent mean ± SEM (n = 8 hippocampi per group; ****$p < 0.0001$; ns, not significant, by multiple unpaired two-tailed t-tests)

(C) Heatmaps (n = 2 hippocampi per time point) from nanopore-based long-read cDNA sequencing showing percent spliced-in (PSI) values of NMDAR1 and Snap25 RNA isoforms in $PXN^{fl/S119A}$ and Nes-CreErT2; $PXN^{fl/S119A}$ hippocampus at indicated time points.

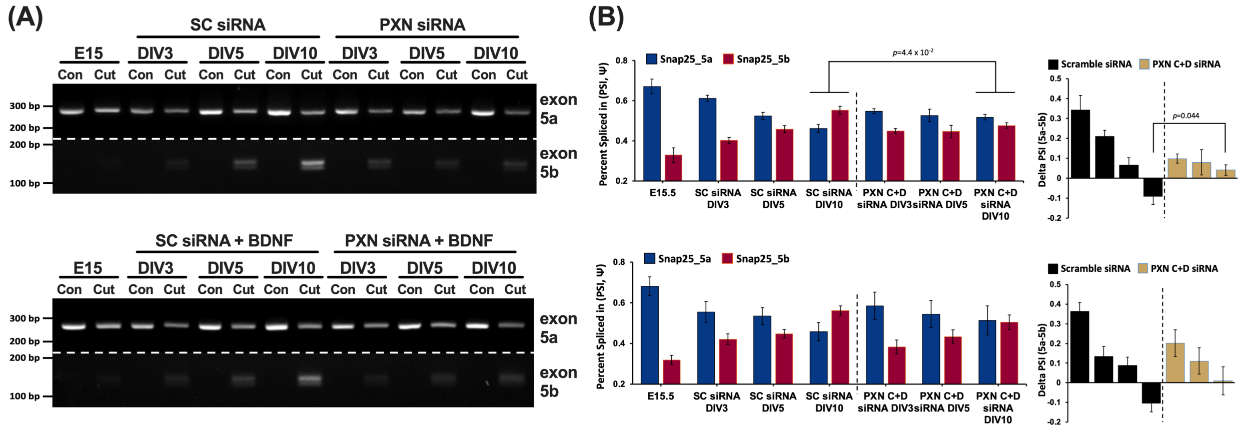

Appendix Figure S7. Related to Figure 5. Paxillin Knockdown Decreases Levels of SNAP25 Isoform 5b mRNA.

(A) Representative images of agarose gels showing RT-PCR products from primary neuronal cultures treated with scrambled control or paxillin siRNA, in the presence or absence of 20 ng/ml BDNF, as indicated. Gels show Snap25 isoforms 5a (270 bp) and 5b (160 bp), identified by AvrII digestion ("cut") or left undigested ("Con").

(B) Histogram summarizing relative inclusion (PSI) and changes in relative inclusion ($\Delta PSI = PSI_{5a} - PSI_{5b}$) for exons 5a and 5b in all experiments shown in **(A)**. Data represent mean ± SEM (n = 4 independent cultures per group; *p<0.05, multiple t-test).

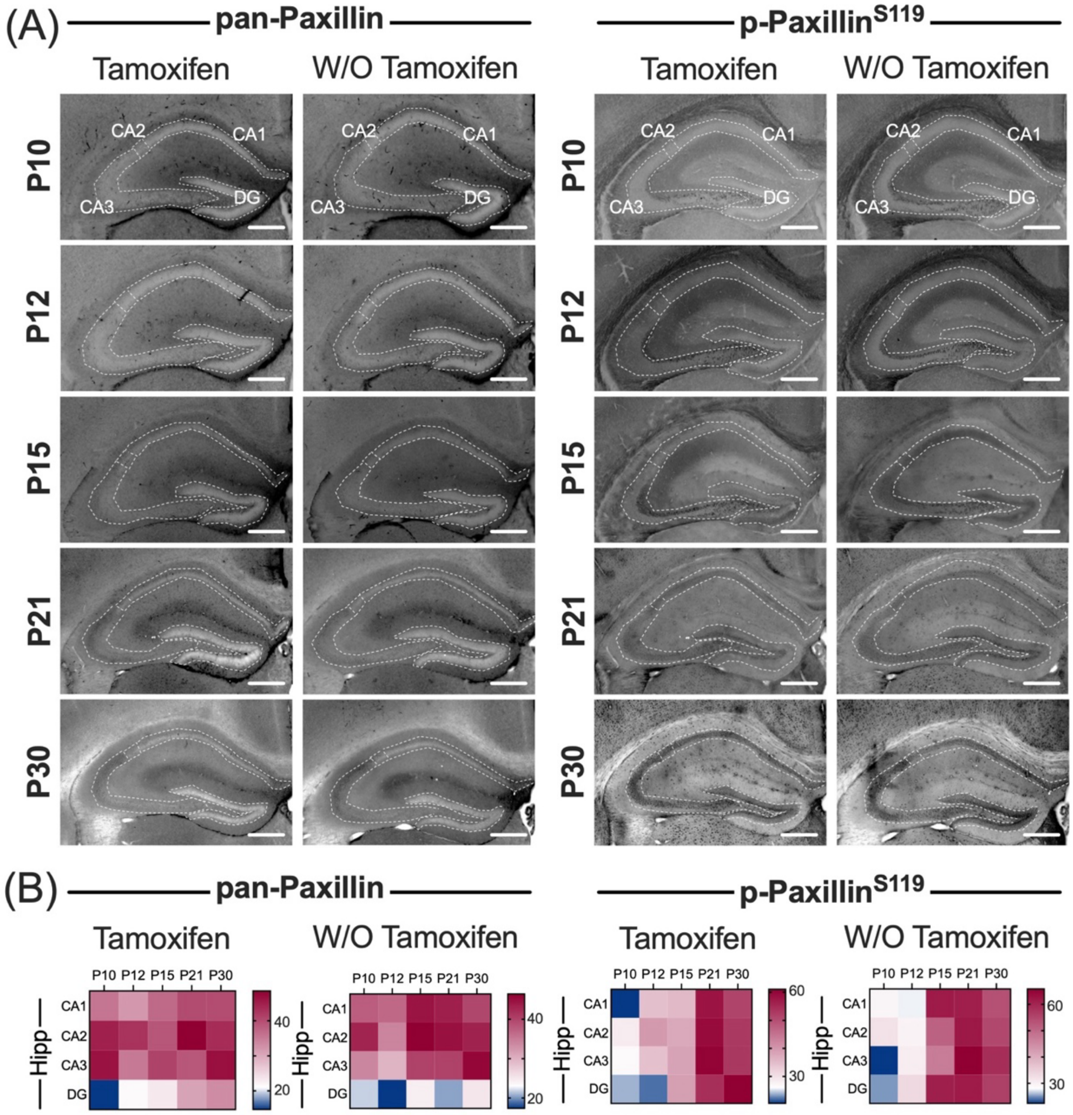

Appendix Figure S8. Validation of p-Paxillin^{S119} Expression in Mice with or without Tamoxifen Administration.

(A) Representative immunohistochemistry images of hippocampal sections at indicated postnatal days from mice administered with or without tamoxifen at P5, P7, and P9. Sections were stained with antibodies against total Paxillin ("pan-Paxillin"; left panels) or phosphorylated Paxillin at serine 119 ("p-Paxillin^{S119}"; right panels). Hippocampal subregions CA1, CA2, CA3, and dentate gyrus (DG) are outlined with dotted lines. Scale bar, 200 µm. **(B)** Heatmap (n = 3 hippocampi per time point) summarizing average intensity of pan-Paxillin and p-Paxillin^{S119} in all experiments shown in (**A**).

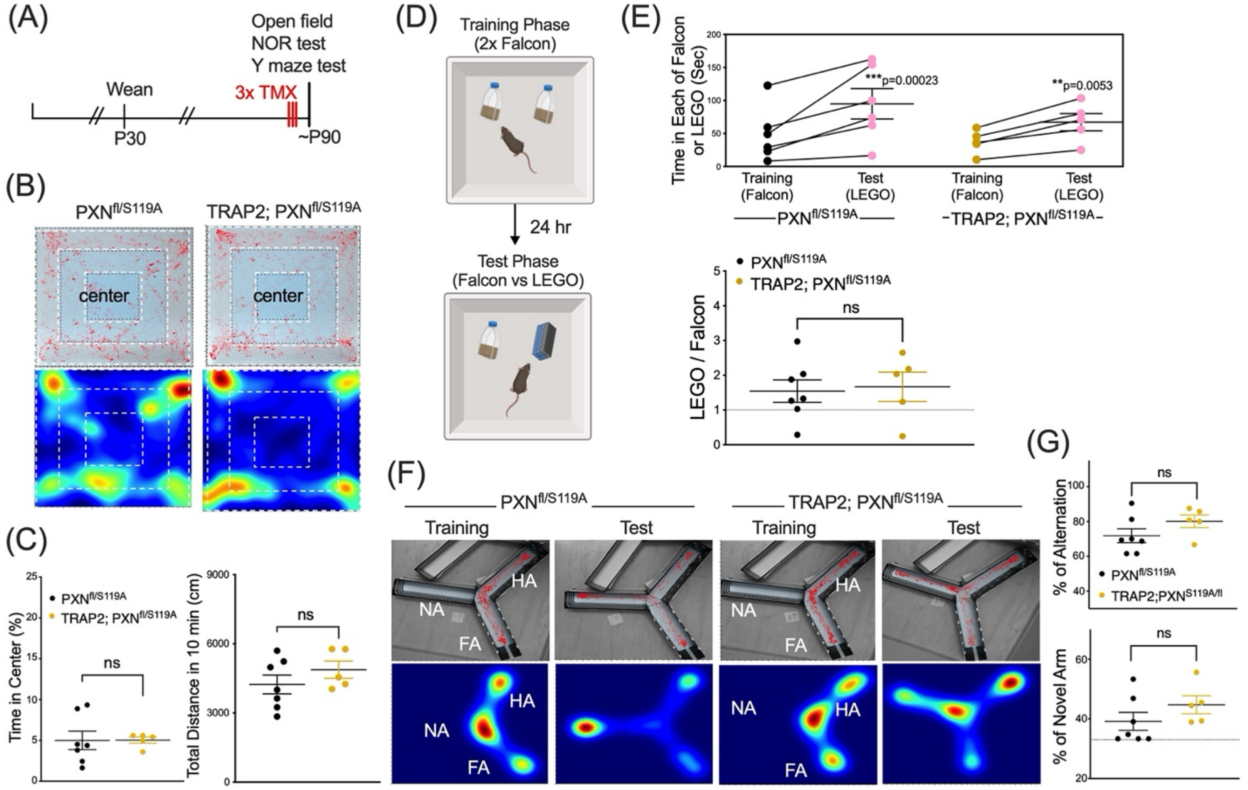

Appendix Figure S9. Late Induction of Postnatal p-Paxillin^{S119} Deficiency Does Not Promote Significant Deficits in Working Memory in Mice.

(A) Schematic showing induction of TRAP2 adult mice (**~P90)** via tamoxifen (TMX) injections, delivered three times (once every other day) starting one week prior to behavioral testing**.**

(B&C) Open field test assessing locomotor activity and anxiety-like behavior in TRAP2; PXN$^{S119A/fl}$ mice and littermate controls. **(B)** Representative movement traces and corresponding heatmaps over a 10 min test session. **(C)** Plot shows percentage of time and total distance traveled by mouse groups (± SEM; n > 5 mice per group; ns, not significant compared to controls by t test) in a 10-min session.

(D&E) Novel object recognition test designed to measure the ability to discriminate a novel (LEGO) from a familiar (Falcon tube) object. **(D)** Schematic of training (two identical Falcon tubes) and test phase (Falcon vs. novel LEGO object) with a 24-hour retention interval. **(E)** Plot showing that mean time spent exploring each object during training and test phases (top). TRAP2; PXN$^{fl/S119A}$ and control mice both spent significantly more time with the novel object during the test. LEGO/Falcon exploration ratios (bottom) were not significantly different between groups. Data represent mean ± SEM (n > 5 mice per group; **p < 0.01, ***p < 0.001; ns, not significant compared to training phase; paired t-test).

(F, G) Y-maze spontaneous alternation test assessing spatial working memory. **(F)** Representative exploration traces and heatmaps from training (exposure to two arms) and test (access to all three arms).

(G) Quantification of spontaneous alternation (top) and percent entries into the novel arm (bottom) showed no significant differences between groups. Data represent mean ± SEM ($n > 5$ mice per group; ns, not significant; paired t-test). HA, home arm; NA, novel arm; FA, familiar arm.
