# Supplementary figures and images for: Nuclear paxillin functions as a molecular switch for alternative splicing in neurons during a critical period of brain development (part 1 of 2)
Source: EMBO J. 2025 Sep 9;44(21):5965–92. doi: 10.1038/s44318-025-00560-8 (PMC12583701; doi:10.1038/s44318-025-00560-8)

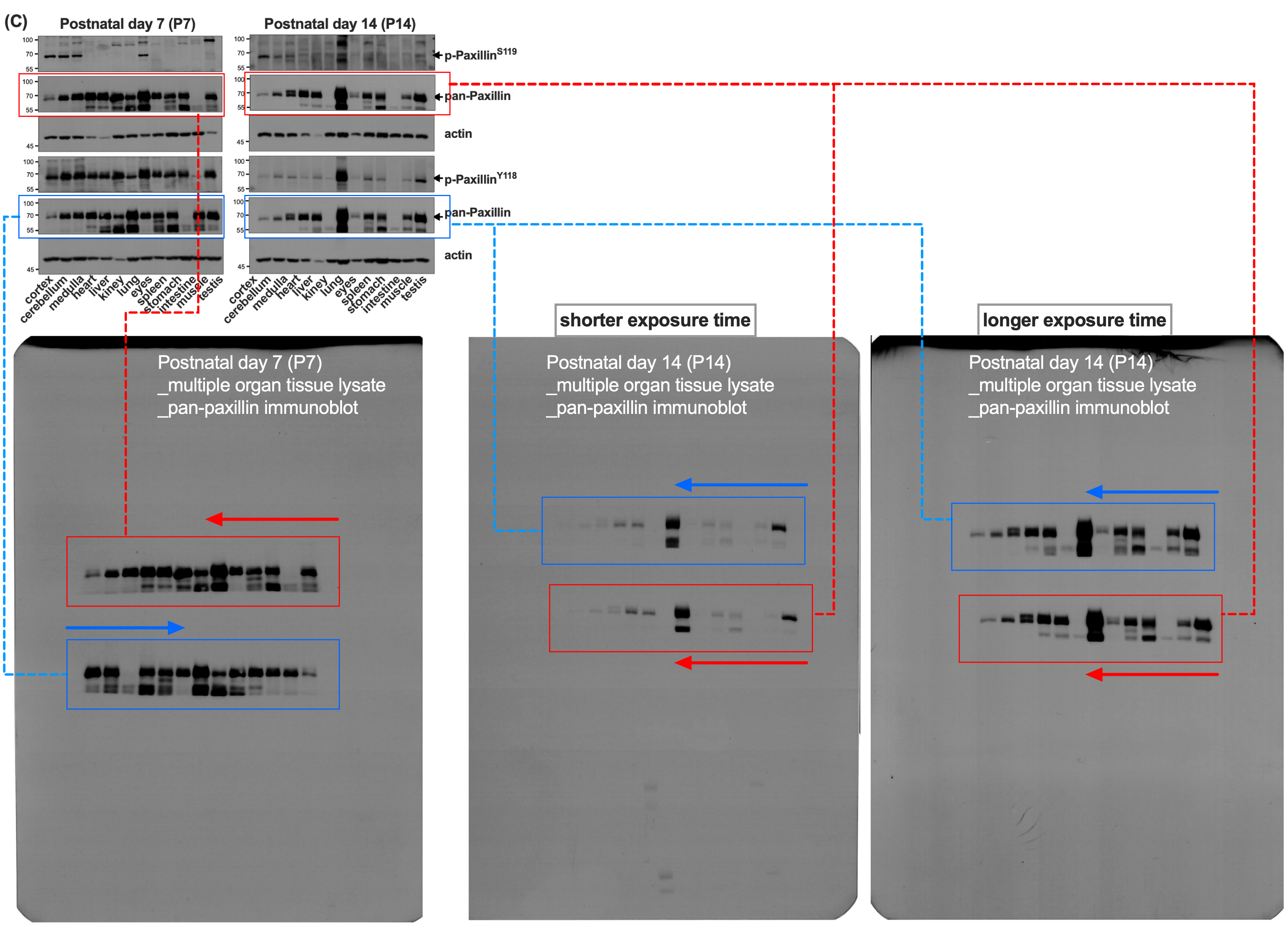

Supplement: Supplementary file 3 — Source data Fig. 1 [file 44318_2025_560_MOESM3_ESM.zip › Figure1/source data_Figure EV1C (pan-Paxillin)_final.tiff]

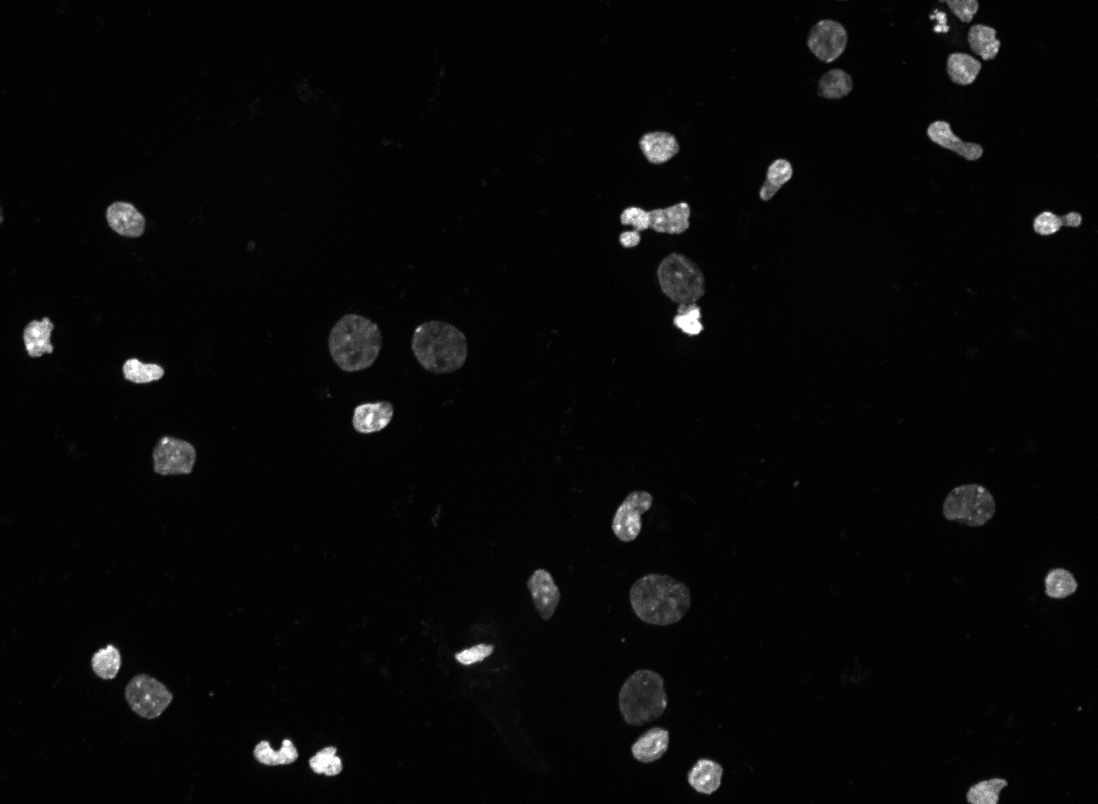

Supplement: Supplementary file 3 — Source data Fig. 1 [file 44318_2025_560_MOESM3_ESM.zip › Figure1/1A/Primary neuron_DIV10_DAPI.tif]

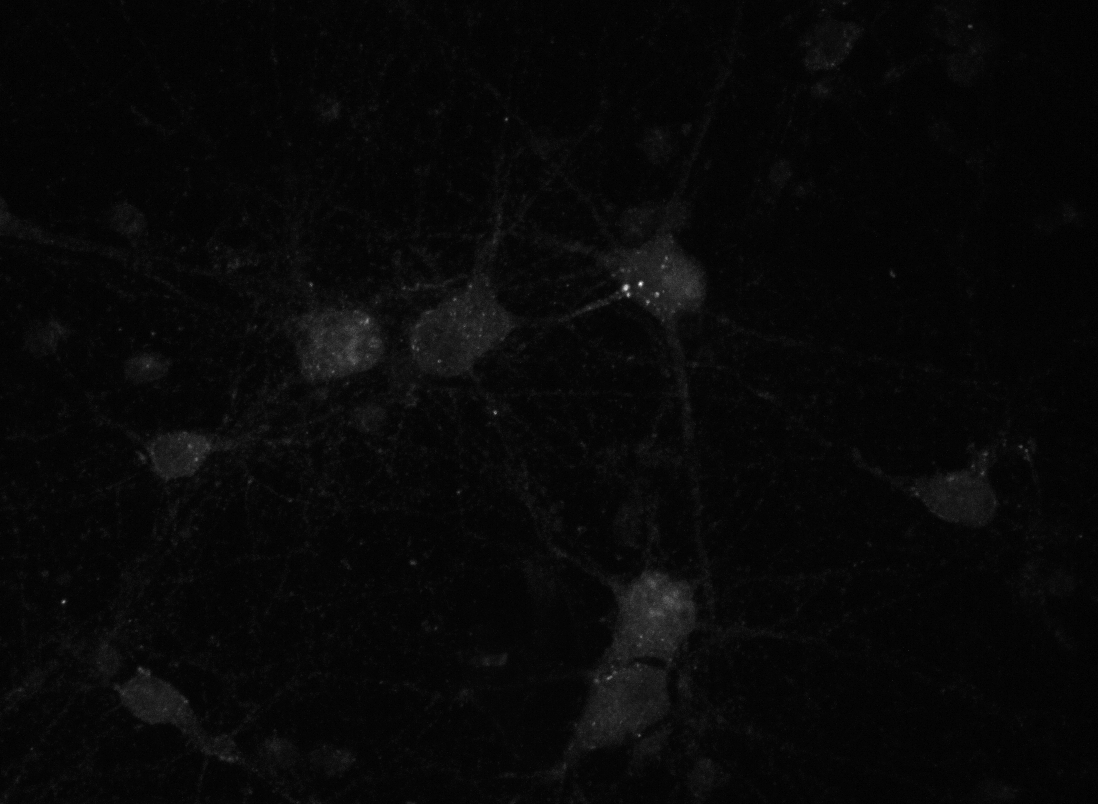

Supplement: Supplementary file 3 — Source data Fig. 1 [file 44318_2025_560_MOESM3_ESM.zip › Figure1/1A/Primary neuron_DIV10_p-PaxillinS119.tif]

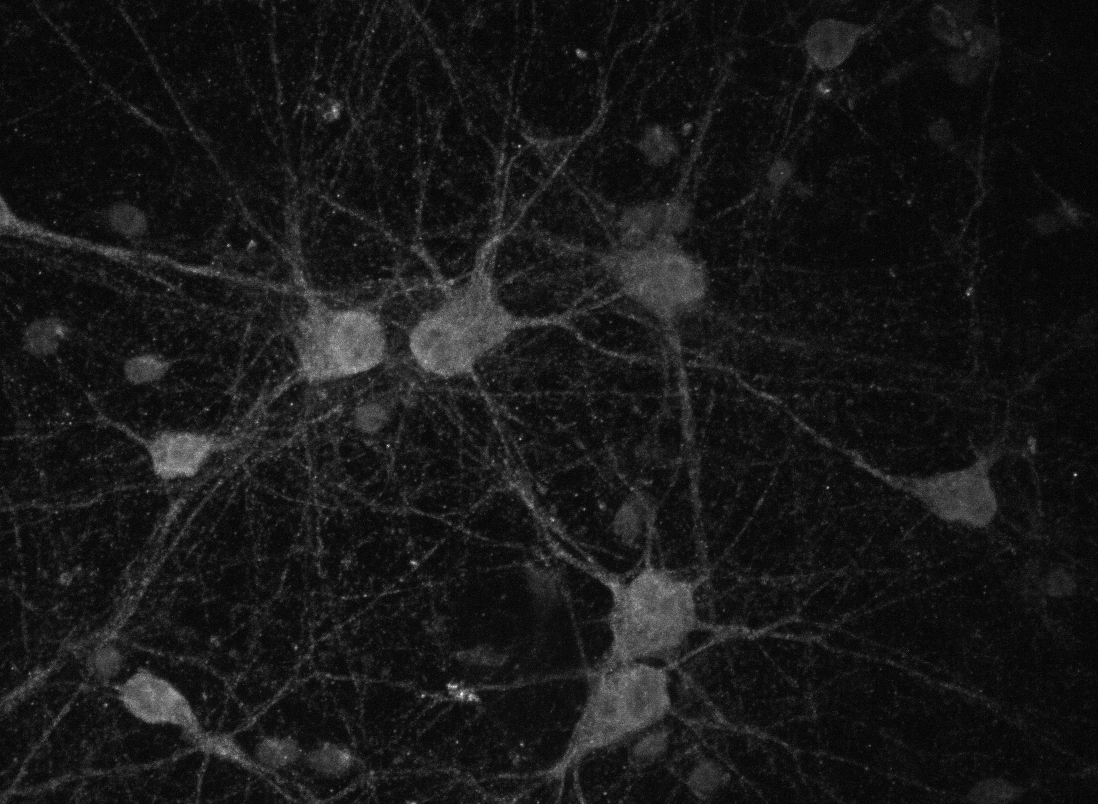

Supplement: Supplementary file 3 — Source data Fig. 1 [file 44318_2025_560_MOESM3_ESM.zip › Figure1/1A/Primary neuron_DIV10_Tuj-1.tif]

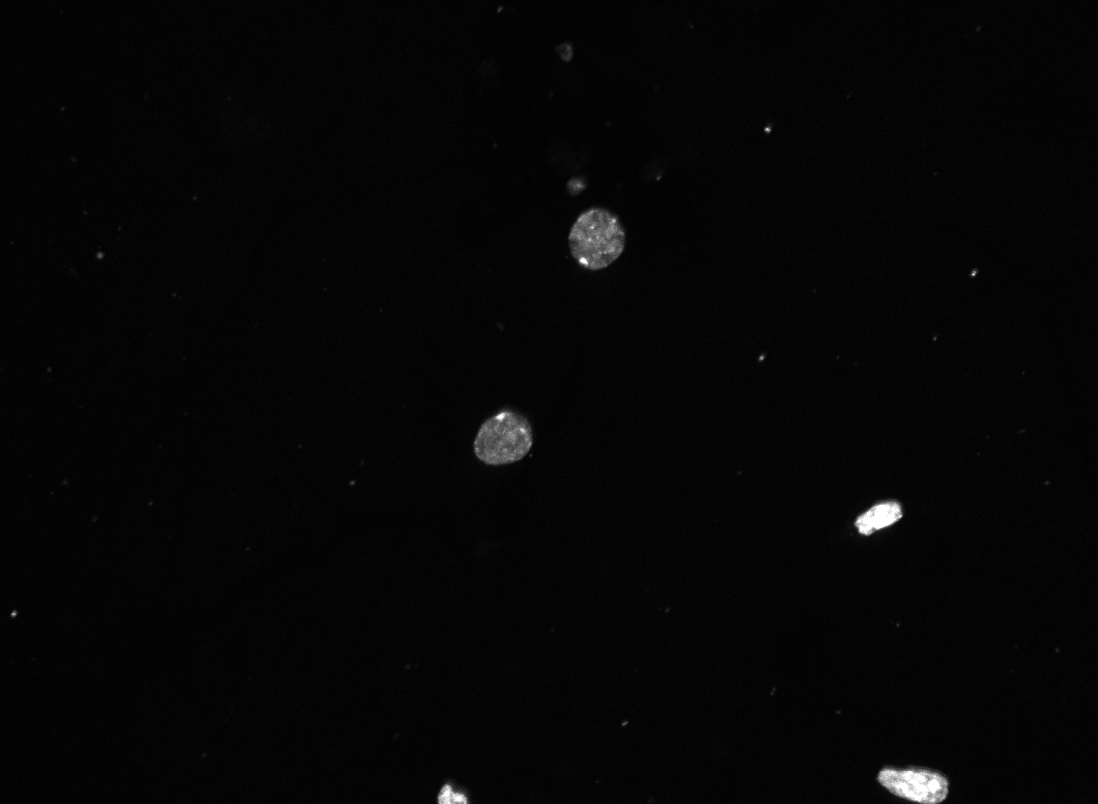

Supplement: Supplementary file 3 — Source data Fig. 1 [file 44318_2025_560_MOESM3_ESM.zip › Figure1/1A/Primary neuron_DIV14_DAPI.tif]

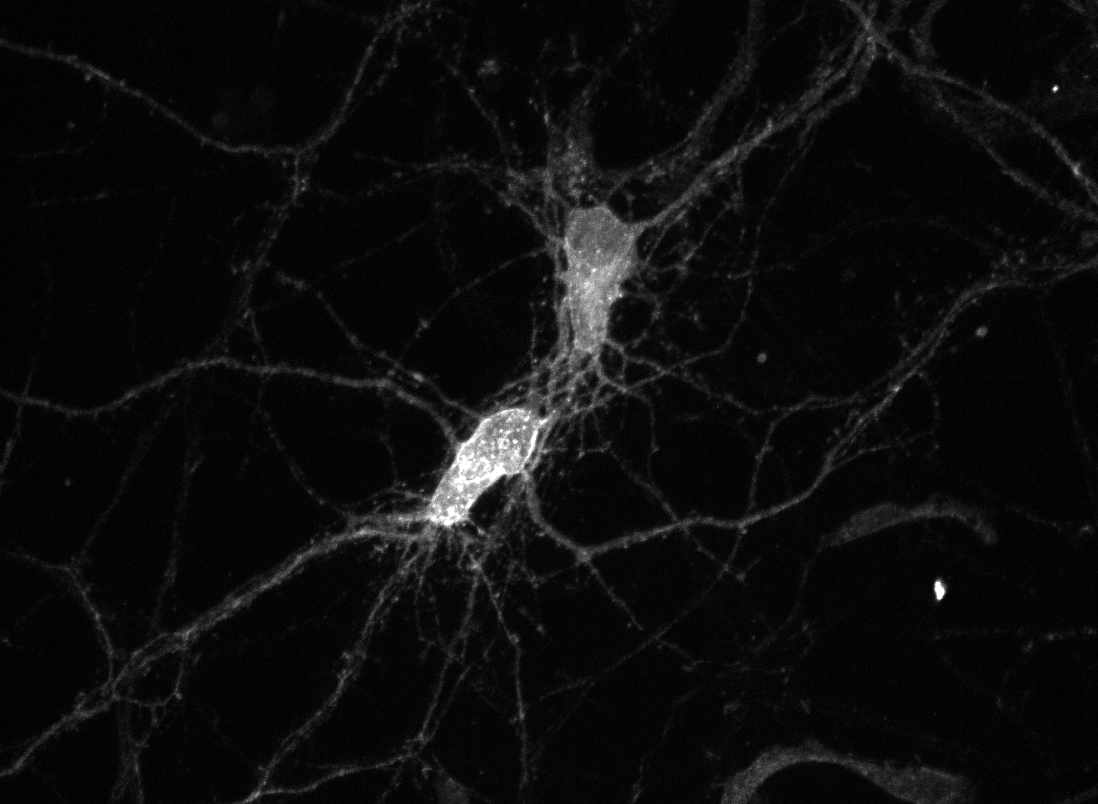

Supplement: Supplementary file 3 — Source data Fig. 1 [file 44318_2025_560_MOESM3_ESM.zip › Figure1/1A/Primary neuron_DIV14_p-PaxillinS119.tif]

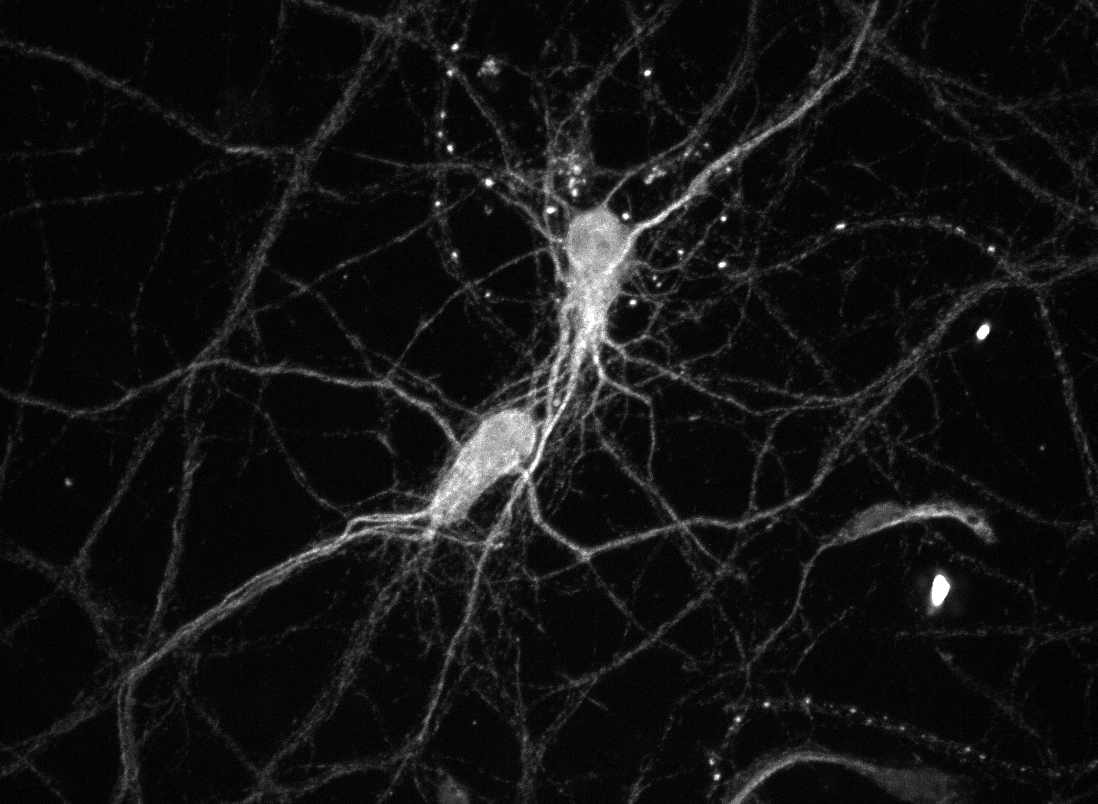

Supplement: Supplementary file 3 — Source data Fig. 1 [file 44318_2025_560_MOESM3_ESM.zip › Figure1/1A/Primary neuron_DIV14_Tuj-1.tif]

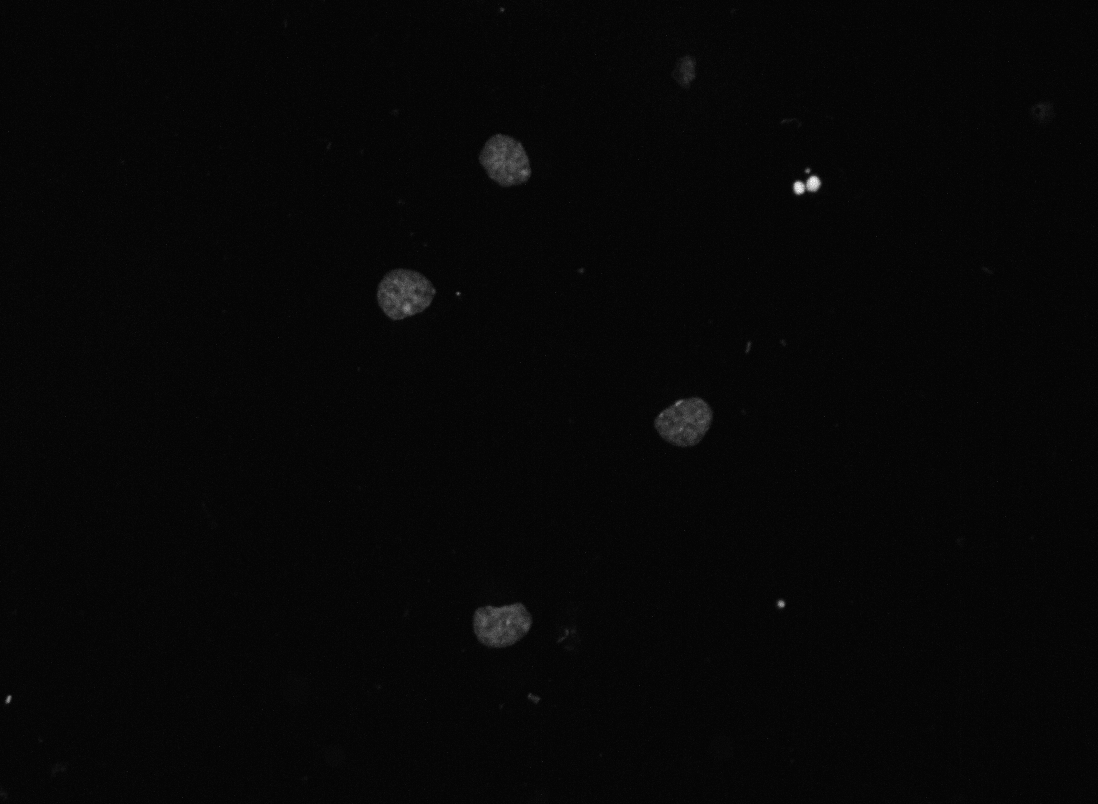

Supplement: Supplementary file 3 — Source data Fig. 1 [file 44318_2025_560_MOESM3_ESM.zip › Figure1/1A/Primary neuron_DIV3_DAPI.tif]

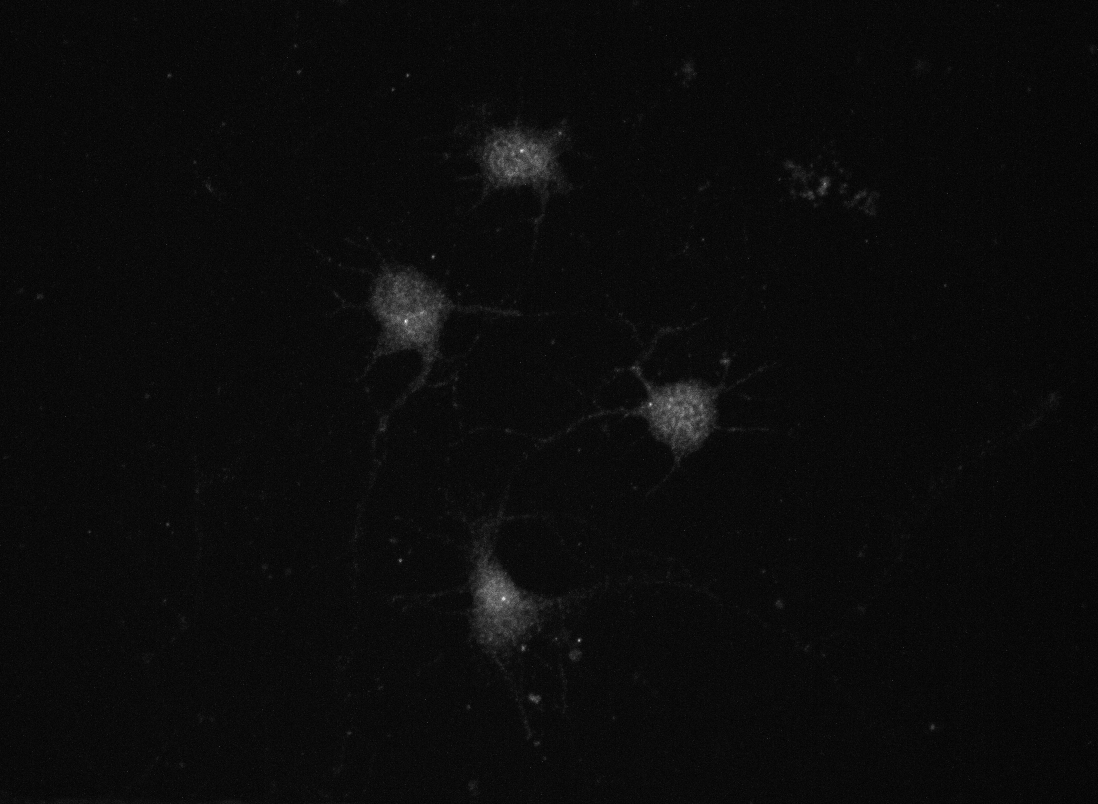

Supplement: Supplementary file 3 — Source data Fig. 1 [file 44318_2025_560_MOESM3_ESM.zip › Figure1/1A/Primary neuron_DIV3_p-PaxillinS119.tif]

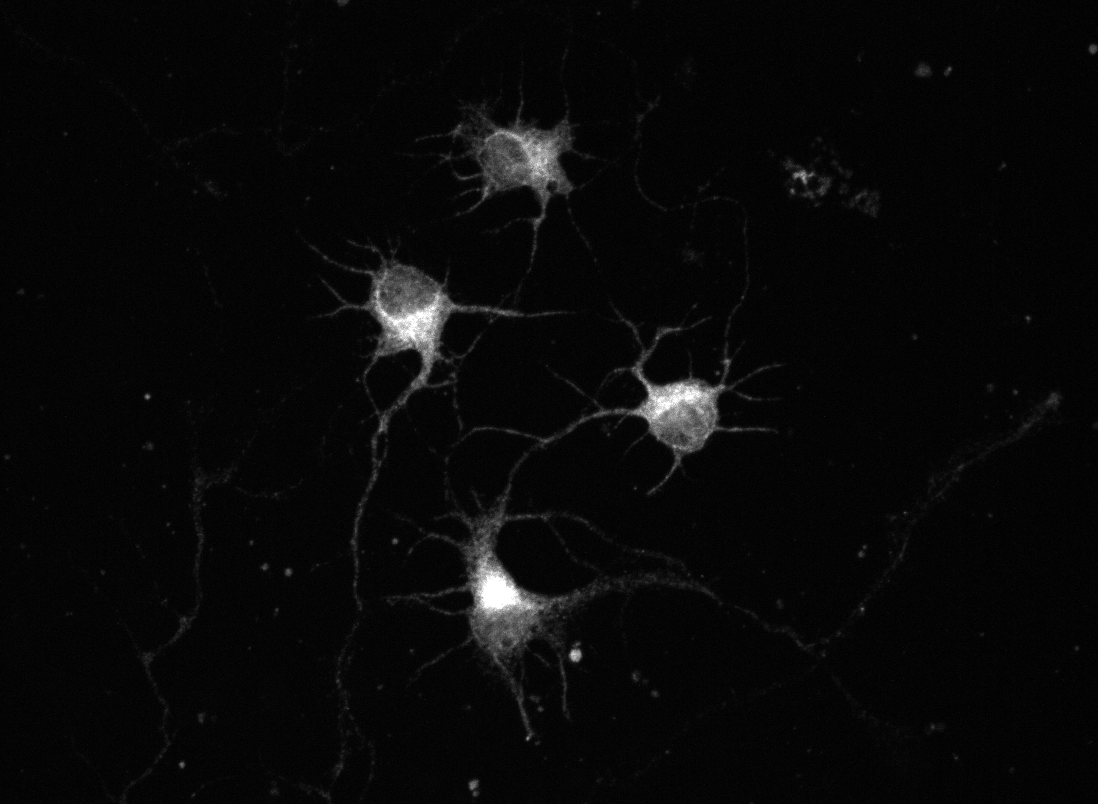

Supplement: Supplementary file 3 — Source data Fig. 1 [file 44318_2025_560_MOESM3_ESM.zip › Figure1/1A/Primary neuron_DIV3_Tuj-1.tif]

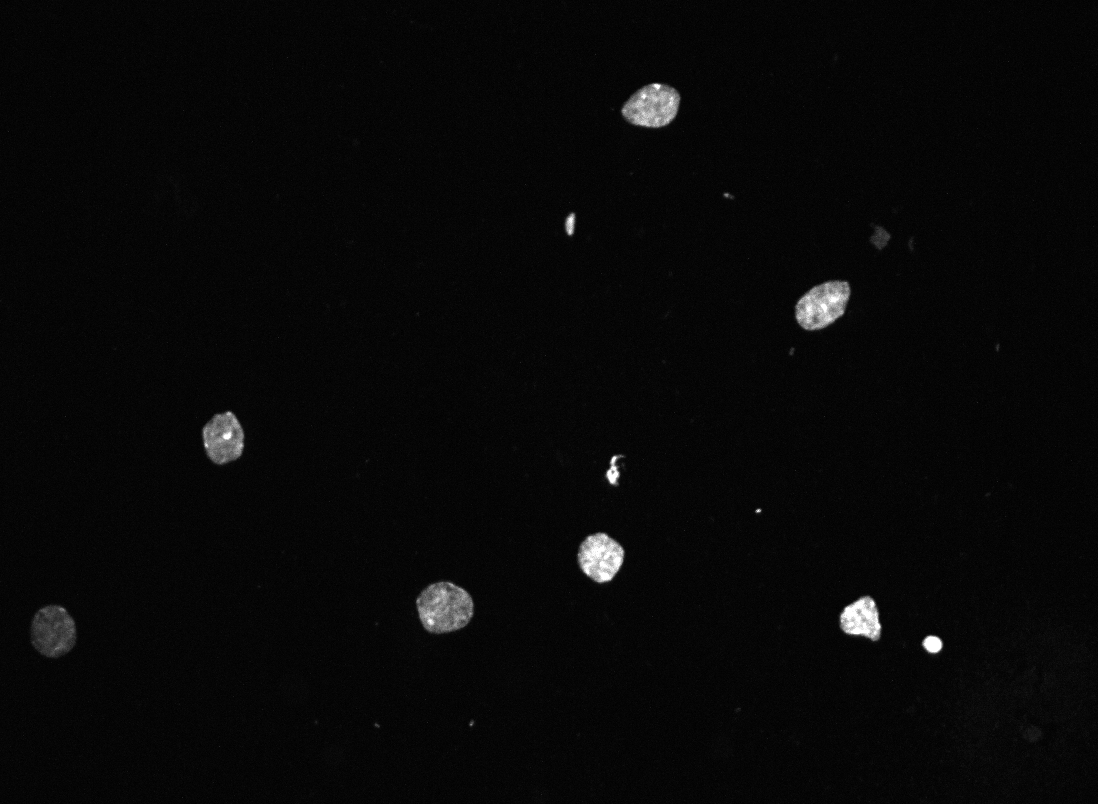

Supplement: Supplementary file 3 — Source data Fig. 1 [file 44318_2025_560_MOESM3_ESM.zip › Figure1/1A/Primary neuron_DIV5_DAPI.tif]

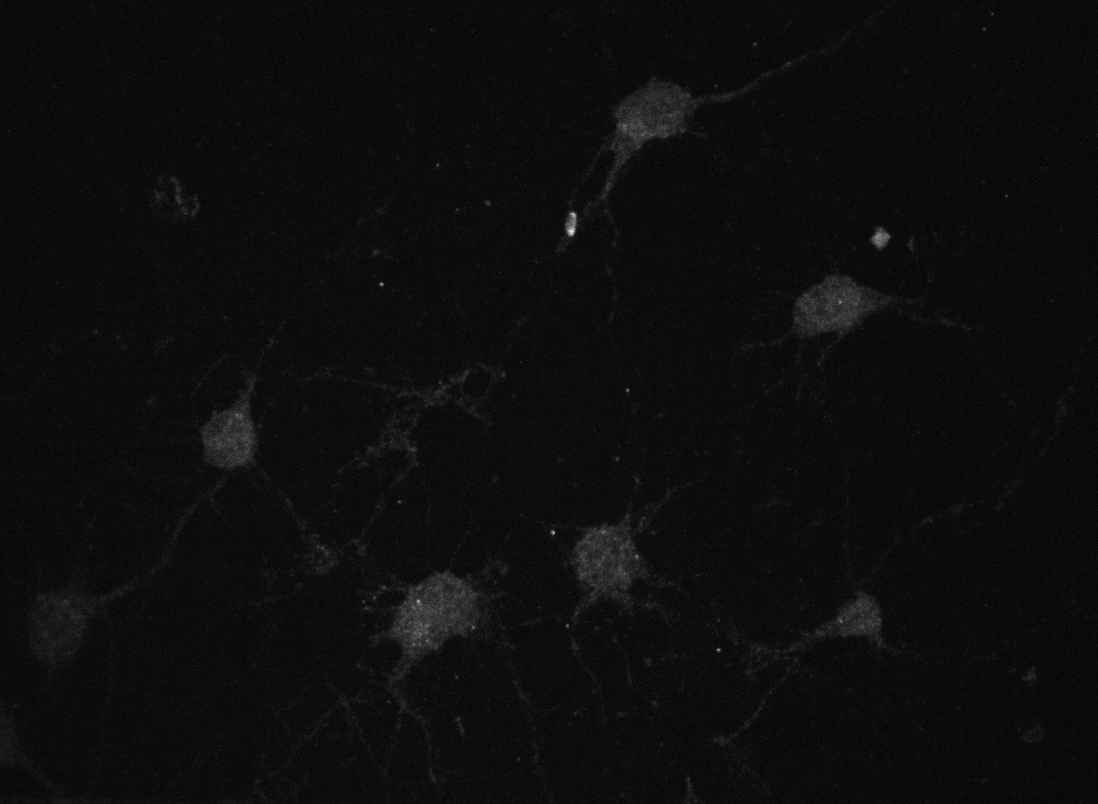

Supplement: Supplementary file 3 — Source data Fig. 1 [file 44318_2025_560_MOESM3_ESM.zip › Figure1/1A/Primary neuron_DIV5_p-PaxillinS119.tif]

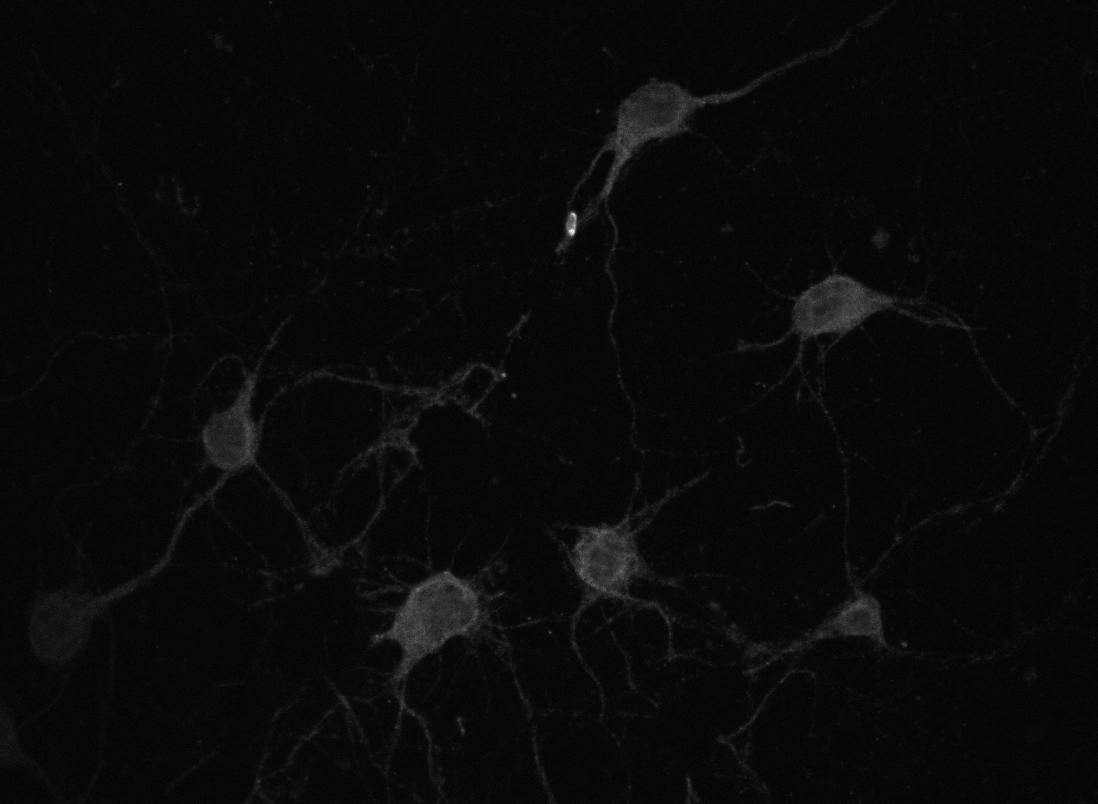

Supplement: Supplementary file 3 — Source data Fig. 1 [file 44318_2025_560_MOESM3_ESM.zip › Figure1/1A/Primary neuron_DIV5_Tuj-1.tif]

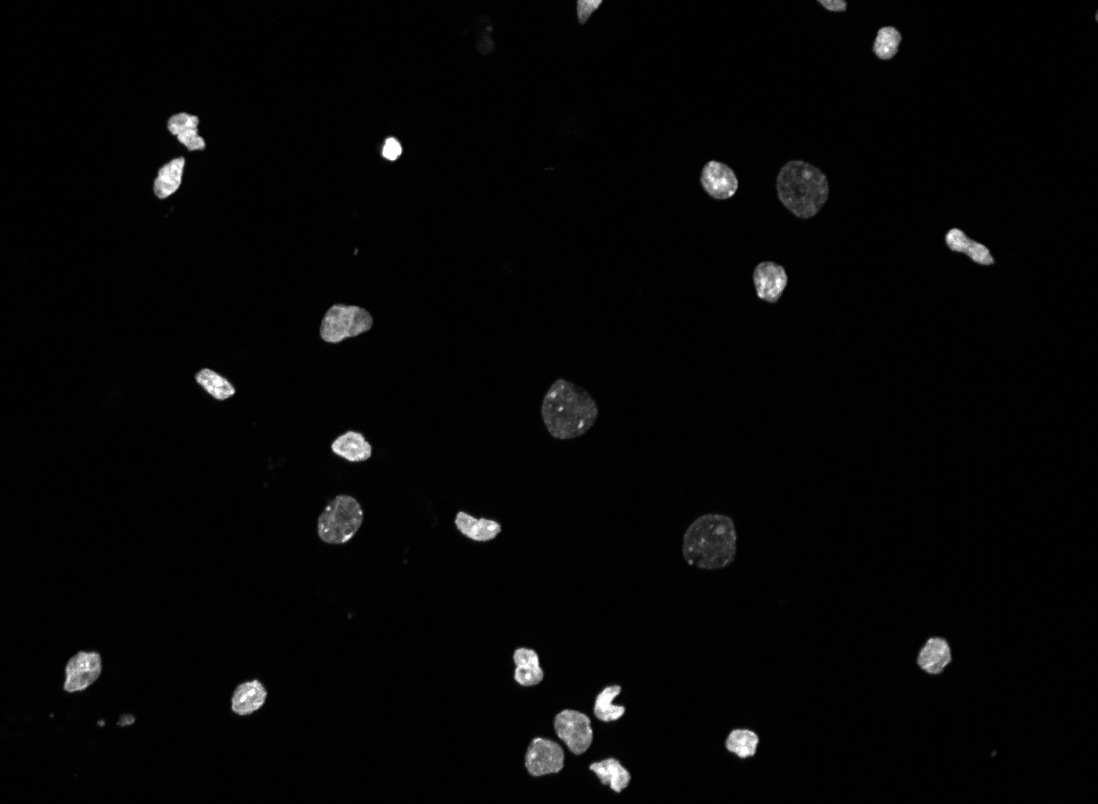

Supplement: Supplementary file 3 — Source data Fig. 1 [file 44318_2025_560_MOESM3_ESM.zip › Figure1/1A/Primary neuron_DIV7_DAPI.tif]

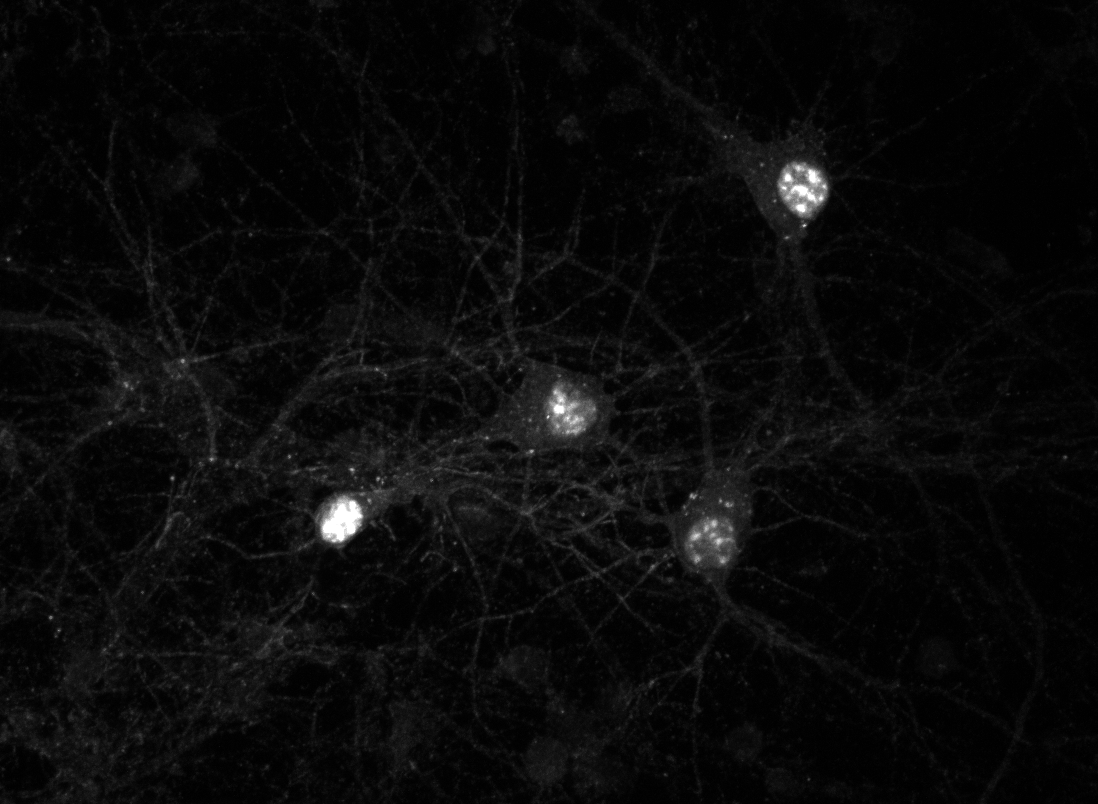

Supplement: Supplementary file 3 — Source data Fig. 1 [file 44318_2025_560_MOESM3_ESM.zip › Figure1/1A/Primary neuron_DIV7_p-PaxillinS119.tif]

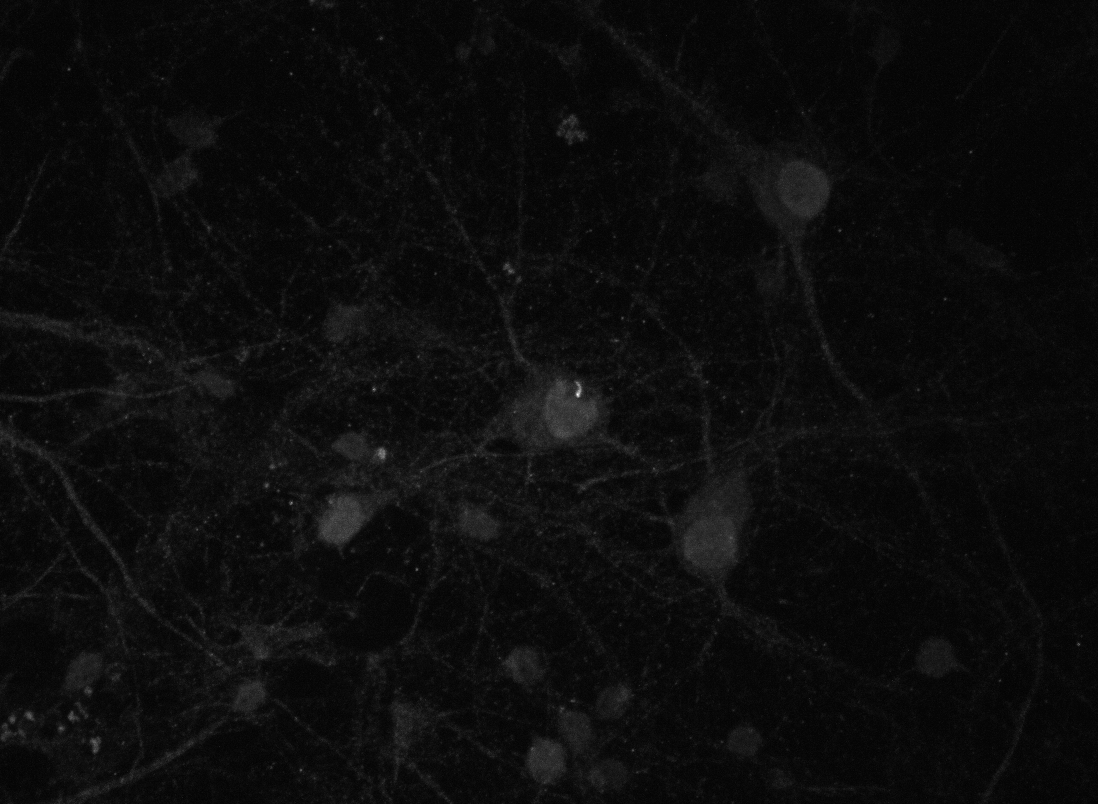

Supplement: Supplementary file 3 — Source data Fig. 1 [file 44318_2025_560_MOESM3_ESM.zip › Figure1/1A/Primary neuron_DIV7_Tuj-1.tif]

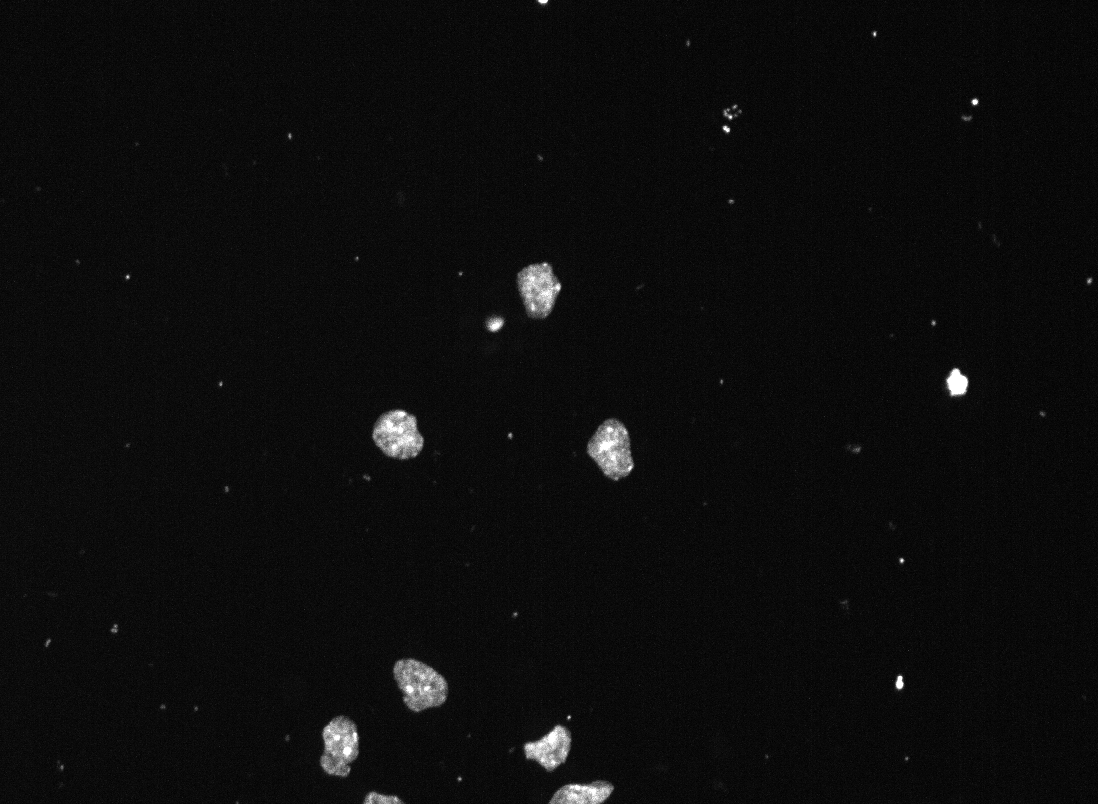

Supplement: Supplementary file 3 — Source data Fig. 1 [file 44318_2025_560_MOESM3_ESM.zip › Figure1/1G/Primary neuron_Control_DIV4_DAPI.tif]

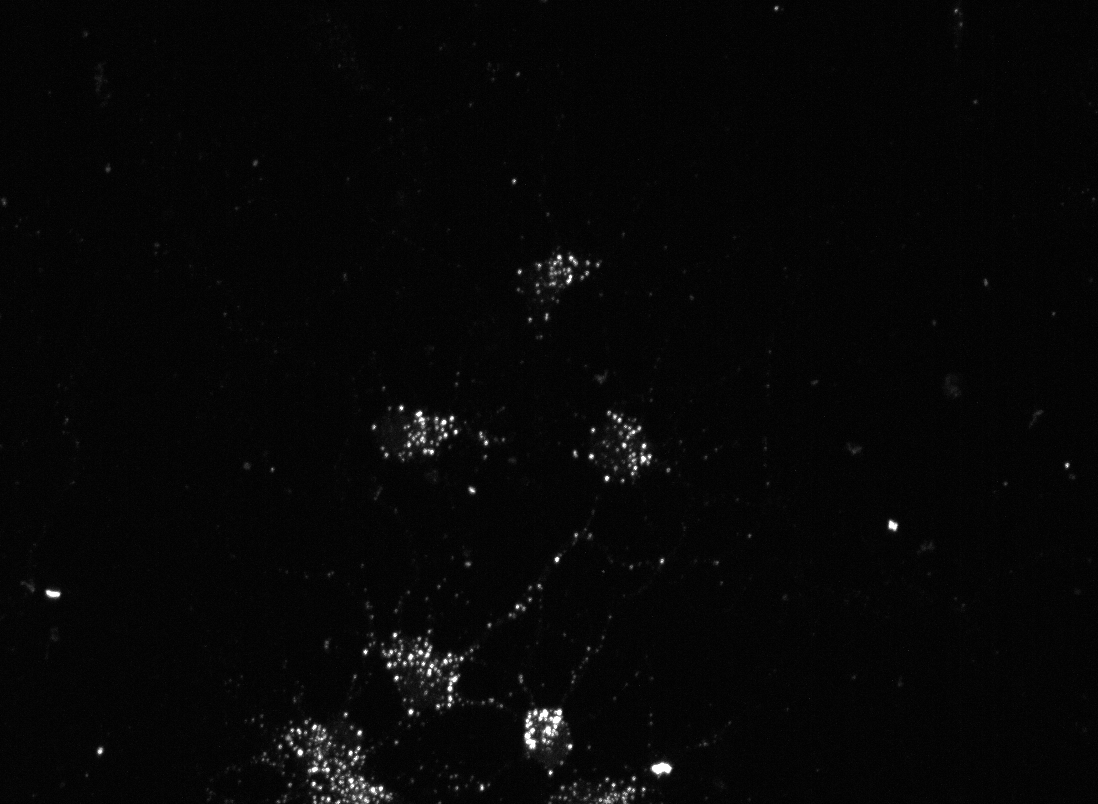

Supplement: Supplementary file 3 — Source data Fig. 1 [file 44318_2025_560_MOESM3_ESM.zip › Figure1/1G/Primary neuron_Control_DIV4_importinB2.tif]

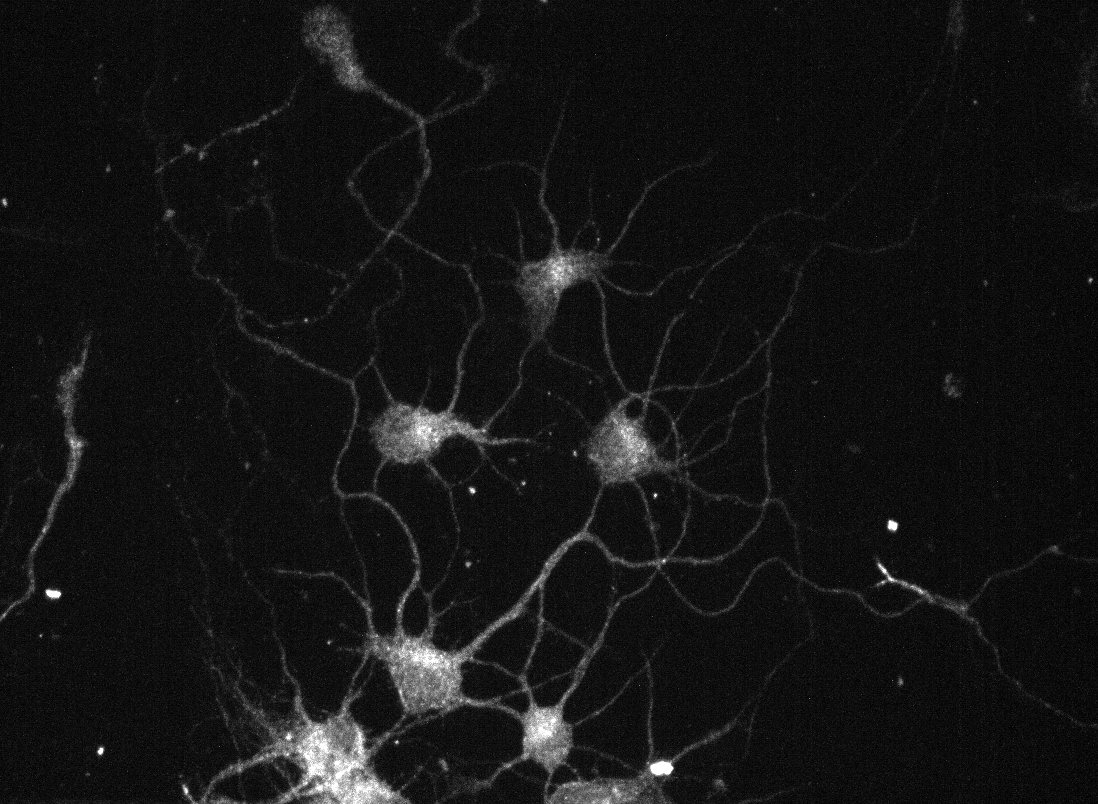

Supplement: Supplementary file 3 — Source data Fig. 1 [file 44318_2025_560_MOESM3_ESM.zip › Figure1/1G/Primary neuron_Control_DIV4_p-PaxillinS119.tif]

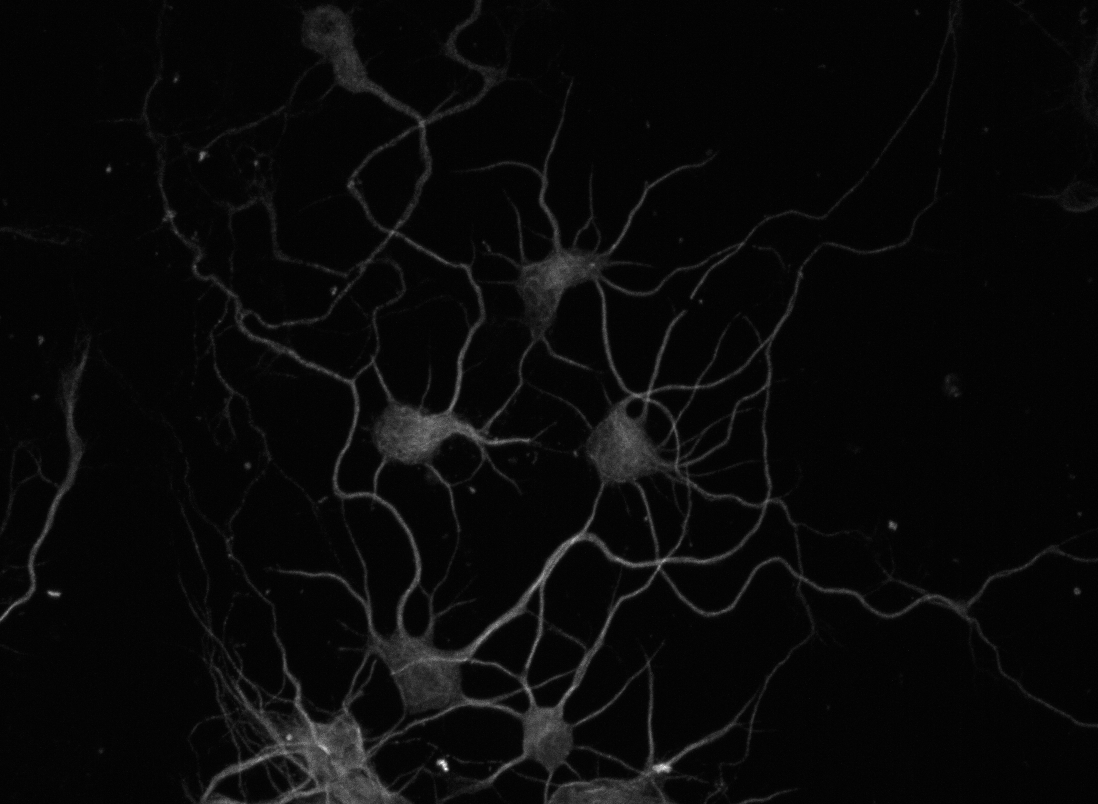

Supplement: Supplementary file 3 — Source data Fig. 1 [file 44318_2025_560_MOESM3_ESM.zip › Figure1/1G/Primary neuron_Control_DIV4_Tuj-1.tif]

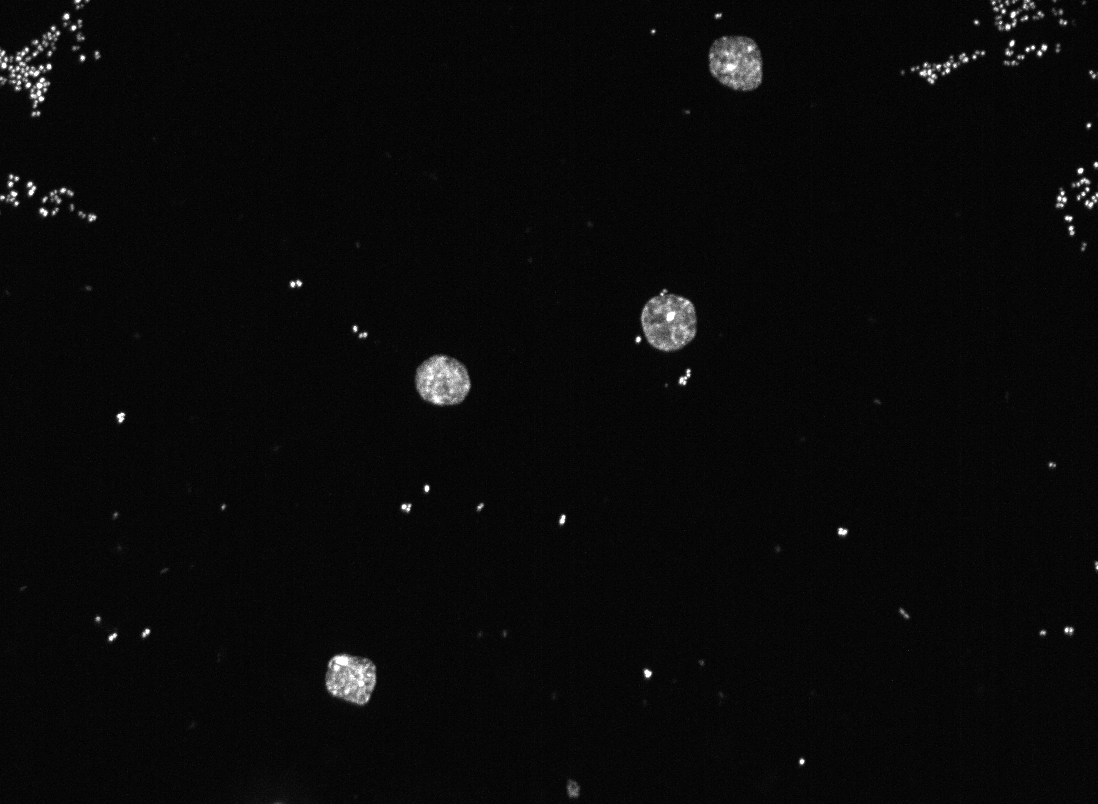

Supplement: Supplementary file 3 — Source data Fig. 1 [file 44318_2025_560_MOESM3_ESM.zip › Figure1/1G/Primary neuron_Control_DIV7_DAPI.tif]

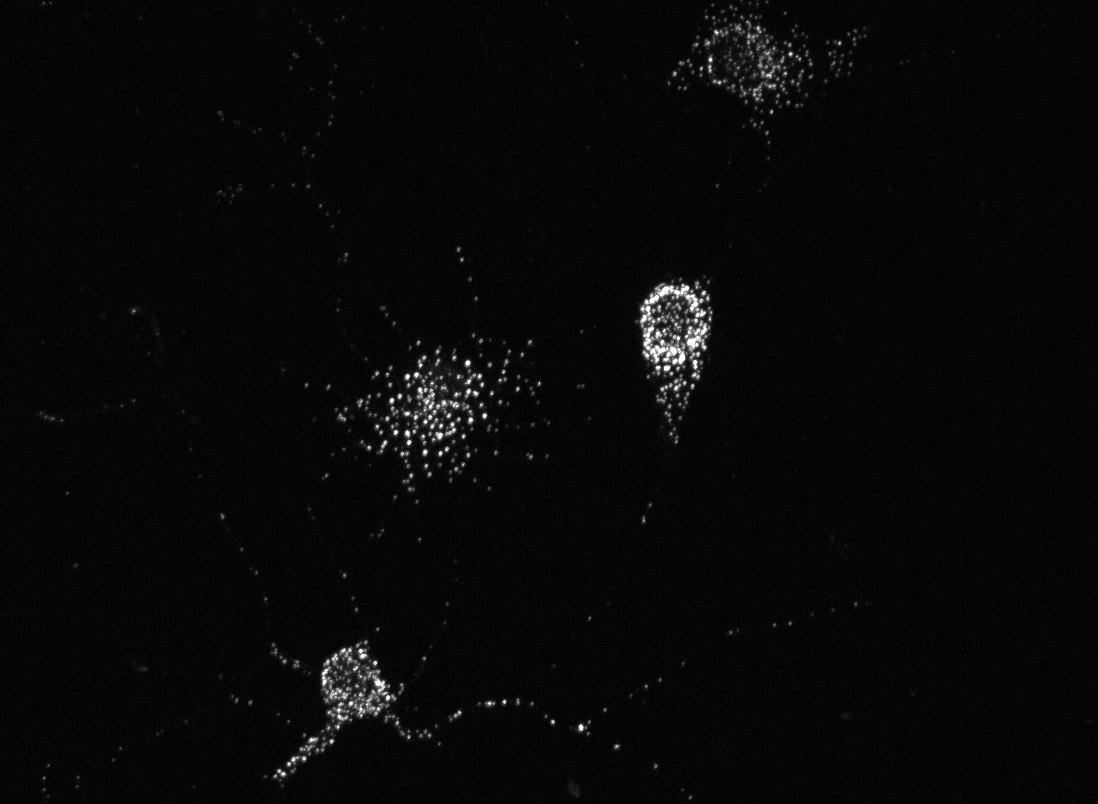

Supplement: Supplementary file 3 — Source data Fig. 1 [file 44318_2025_560_MOESM3_ESM.zip › Figure1/1G/Primary neuron_Control_DIV7_importinB2.tif]

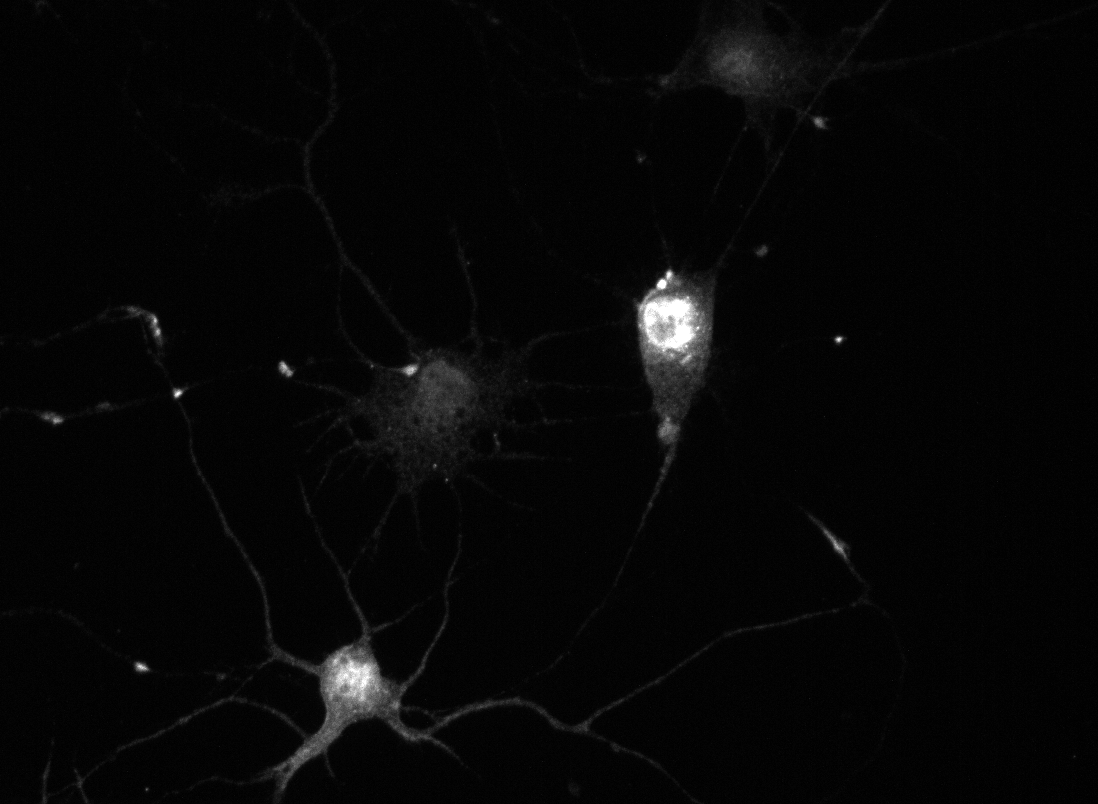

Supplement: Supplementary file 3 — Source data Fig. 1 [file 44318_2025_560_MOESM3_ESM.zip › Figure1/1G/Primary neuron_Control_DIV7_p-PaxillinS119.tif]

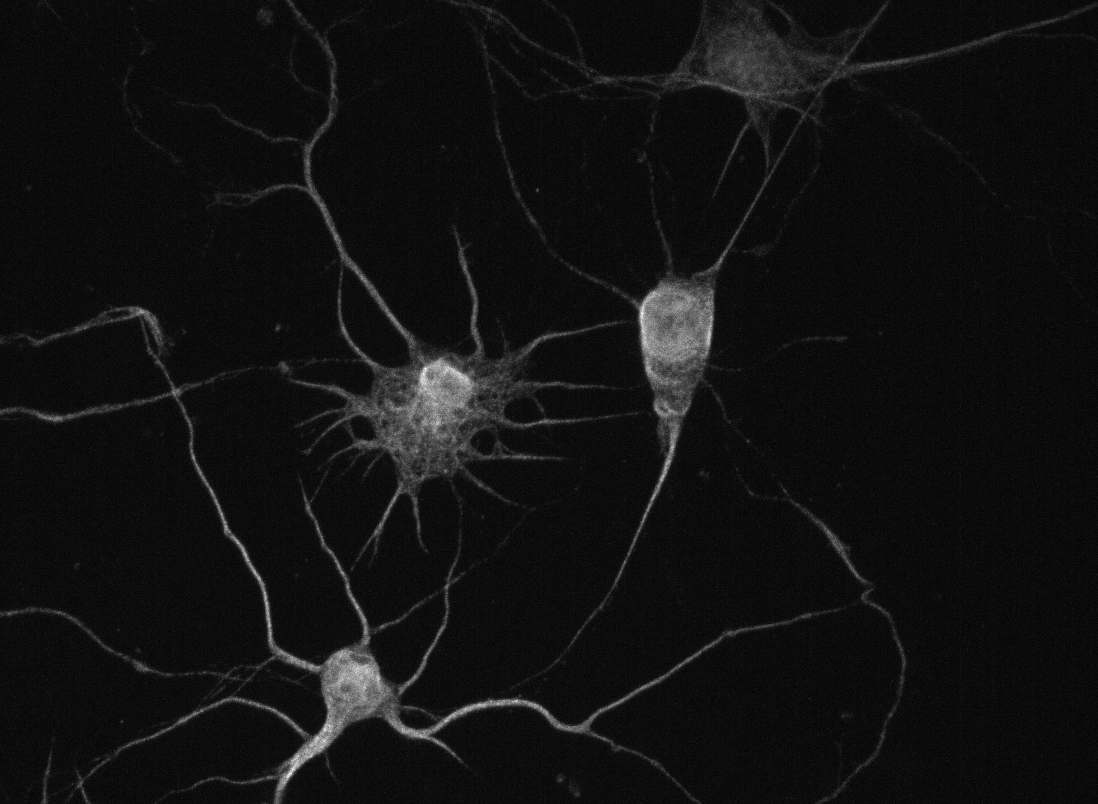

Supplement: Supplementary file 3 — Source data Fig. 1 [file 44318_2025_560_MOESM3_ESM.zip › Figure1/1G/Primary neuron_Control_DIV7_Tuj-1.tif]

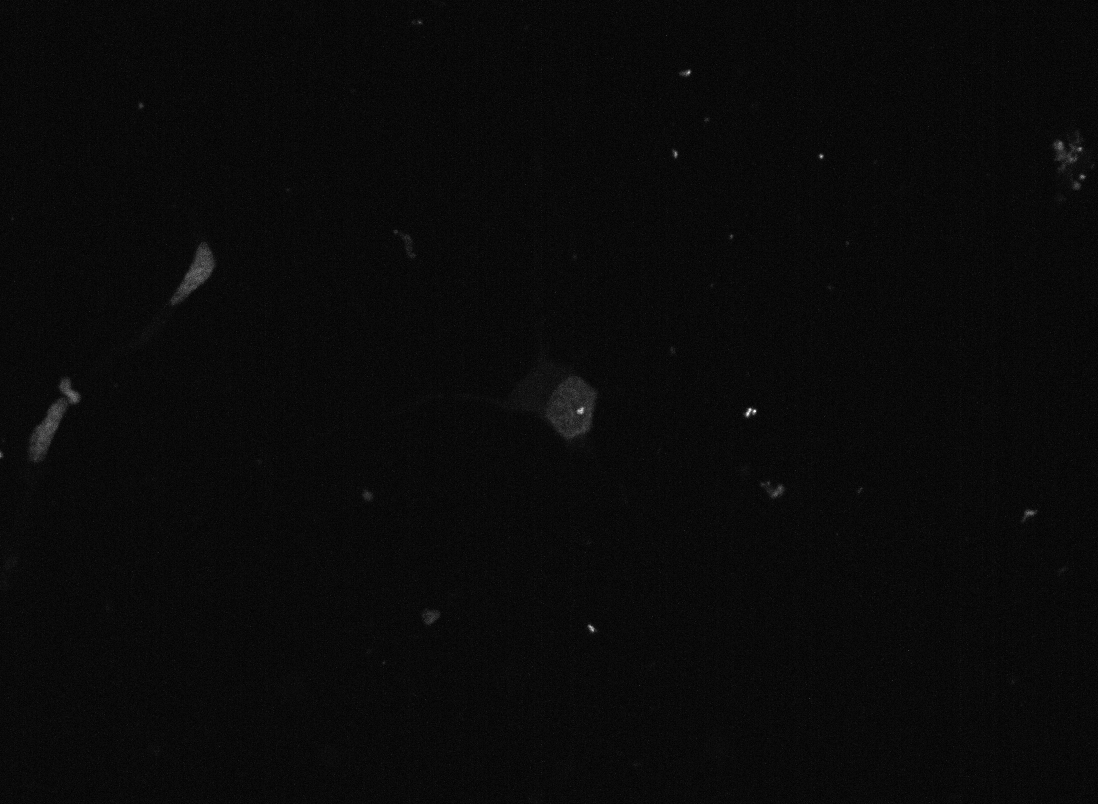

Supplement: Supplementary file 3 — Source data Fig. 1 [file 44318_2025_560_MOESM3_ESM.zip › Figure1/1G/Primary neuron_IPZ_DIV7_DAPI.tif]

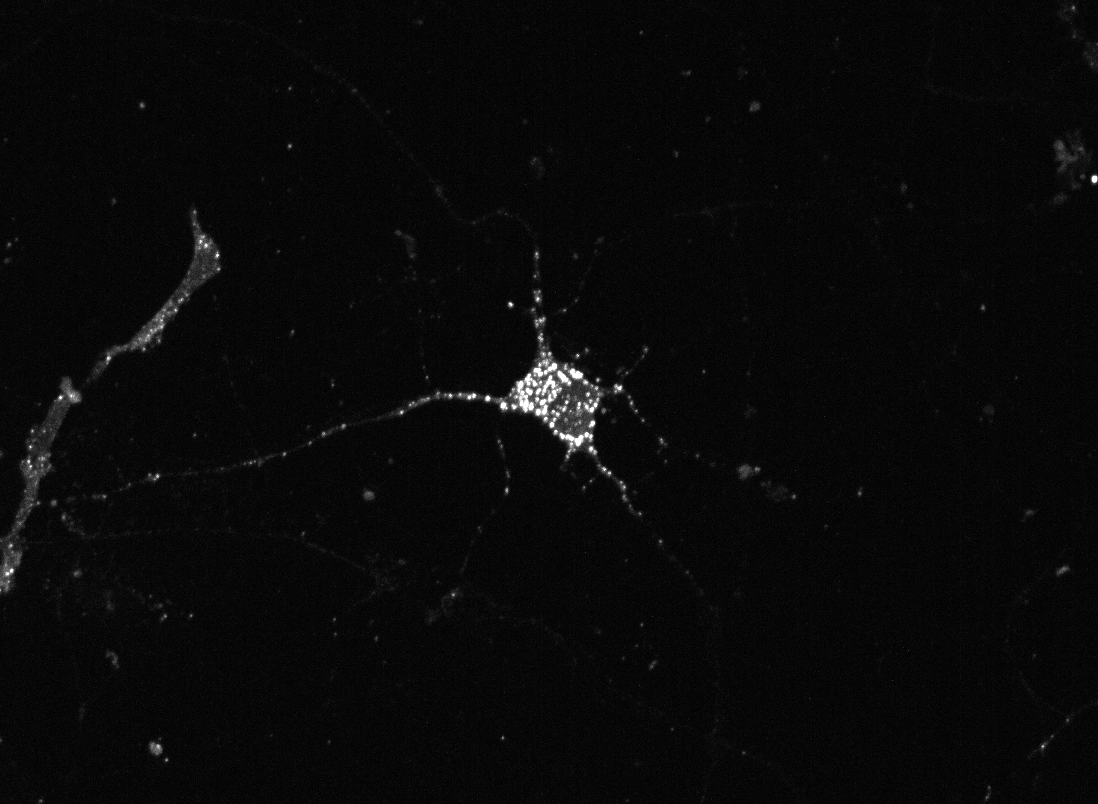

Supplement: Supplementary file 3 — Source data Fig. 1 [file 44318_2025_560_MOESM3_ESM.zip › Figure1/1G/Primary neuron_IPZ_DIV7_importinB2.tif]

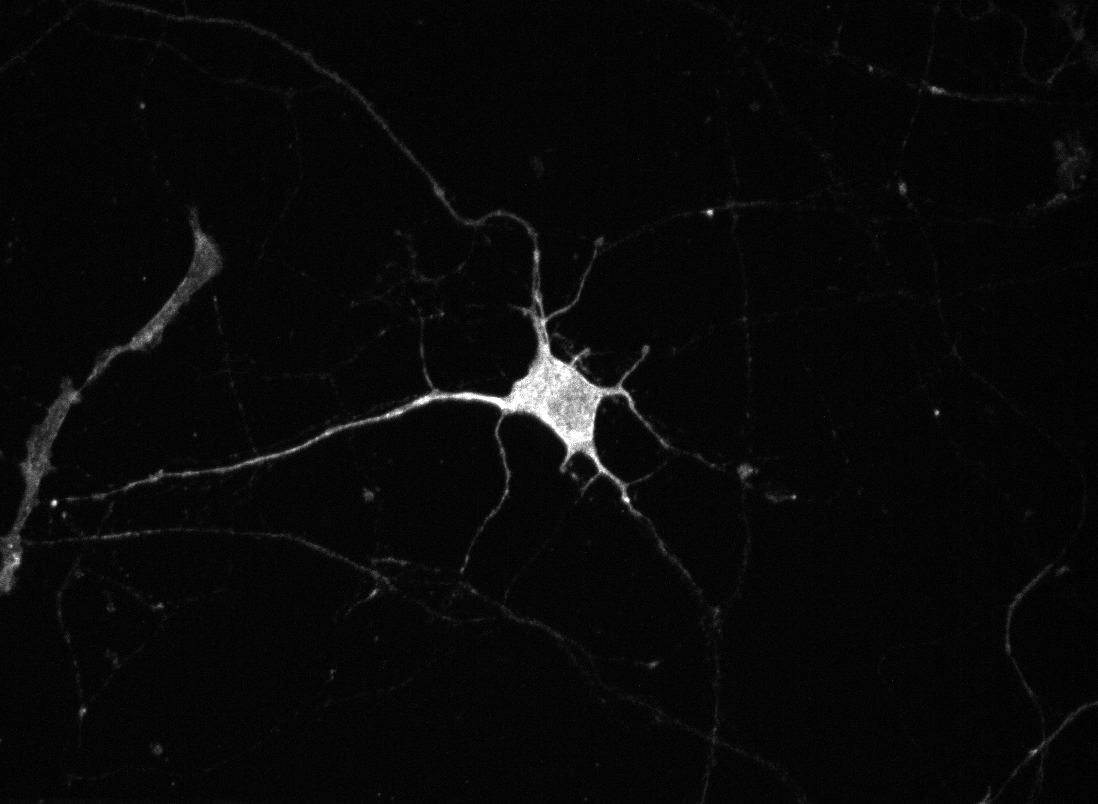

Supplement: Supplementary file 3 — Source data Fig. 1 [file 44318_2025_560_MOESM3_ESM.zip › Figure1/1G/Primary neuron_IPZ_DIV7_p-PaxillinS119.tif]

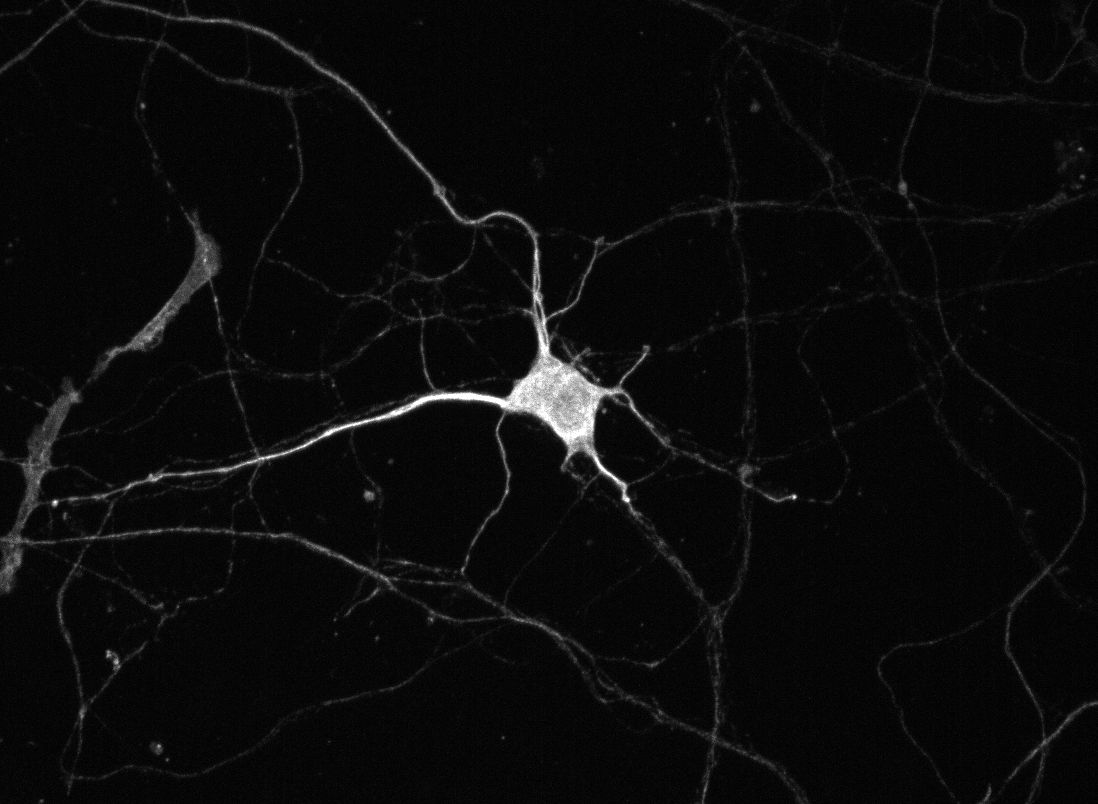

Supplement: Supplementary file 3 — Source data Fig. 1 [file 44318_2025_560_MOESM3_ESM.zip › Figure1/1G/Primary neuron_IPZ_DIV7_Tuj-1.tif]

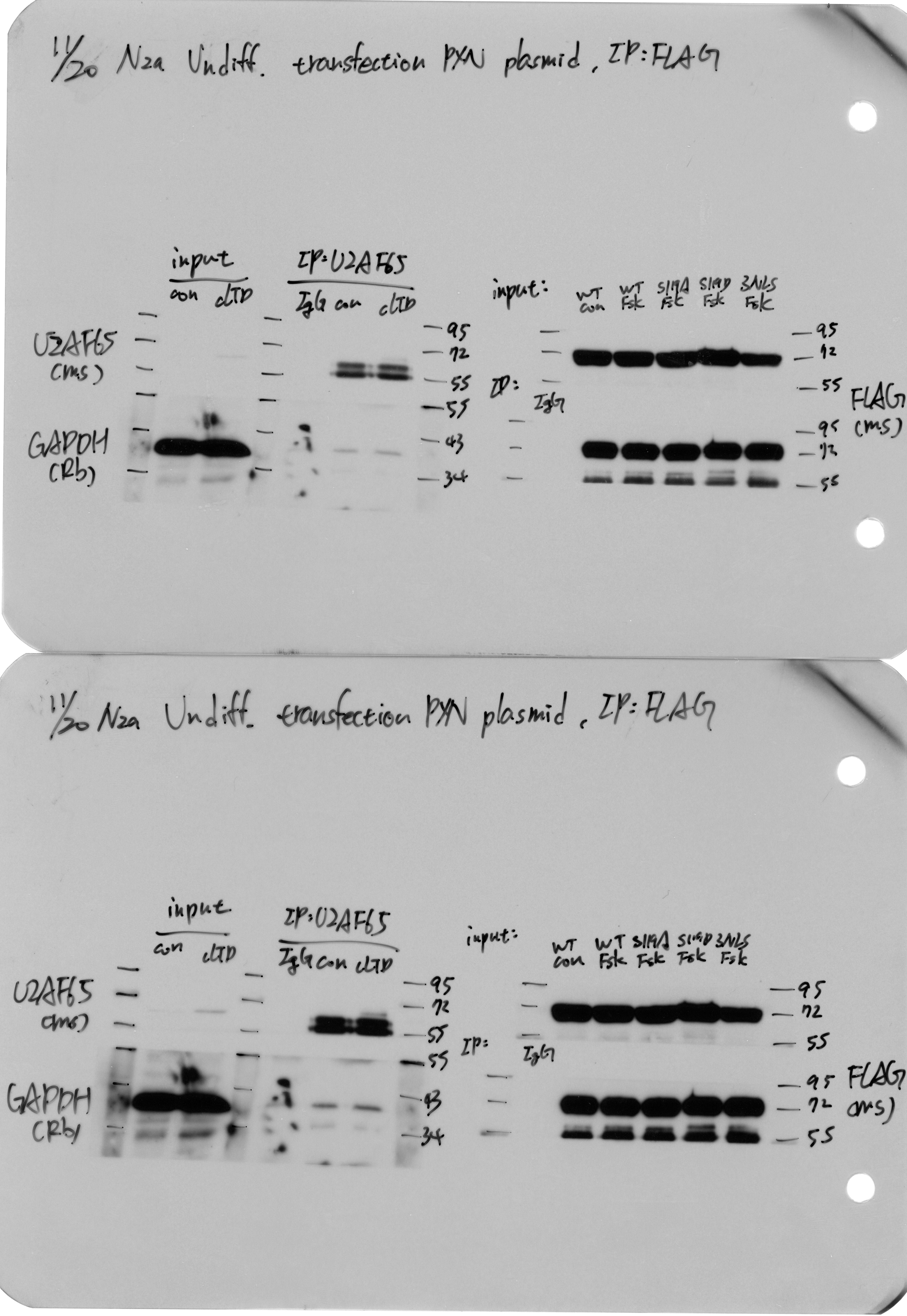

Supplement: Supplementary file 3 — Source data Fig. 1 [file 44318_2025_560_MOESM3_ESM.zip › Figure1/1K/IP-FLAG western_FLAG-1.tif]

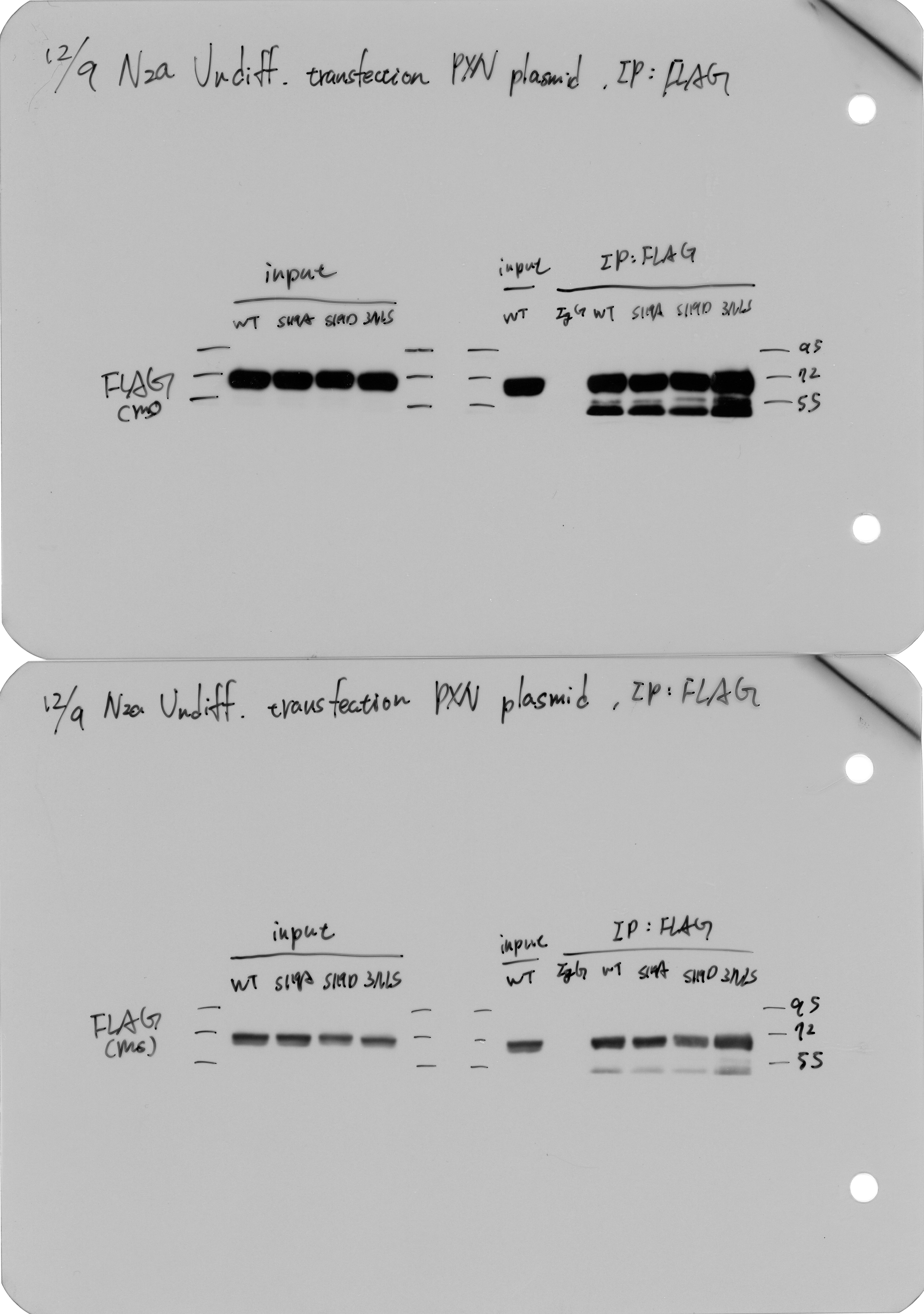

Supplement: Supplementary file 3 — Source data Fig. 1 [file 44318_2025_560_MOESM3_ESM.zip › Figure1/1K/IP-FLAG western_FLAG-2.tif]

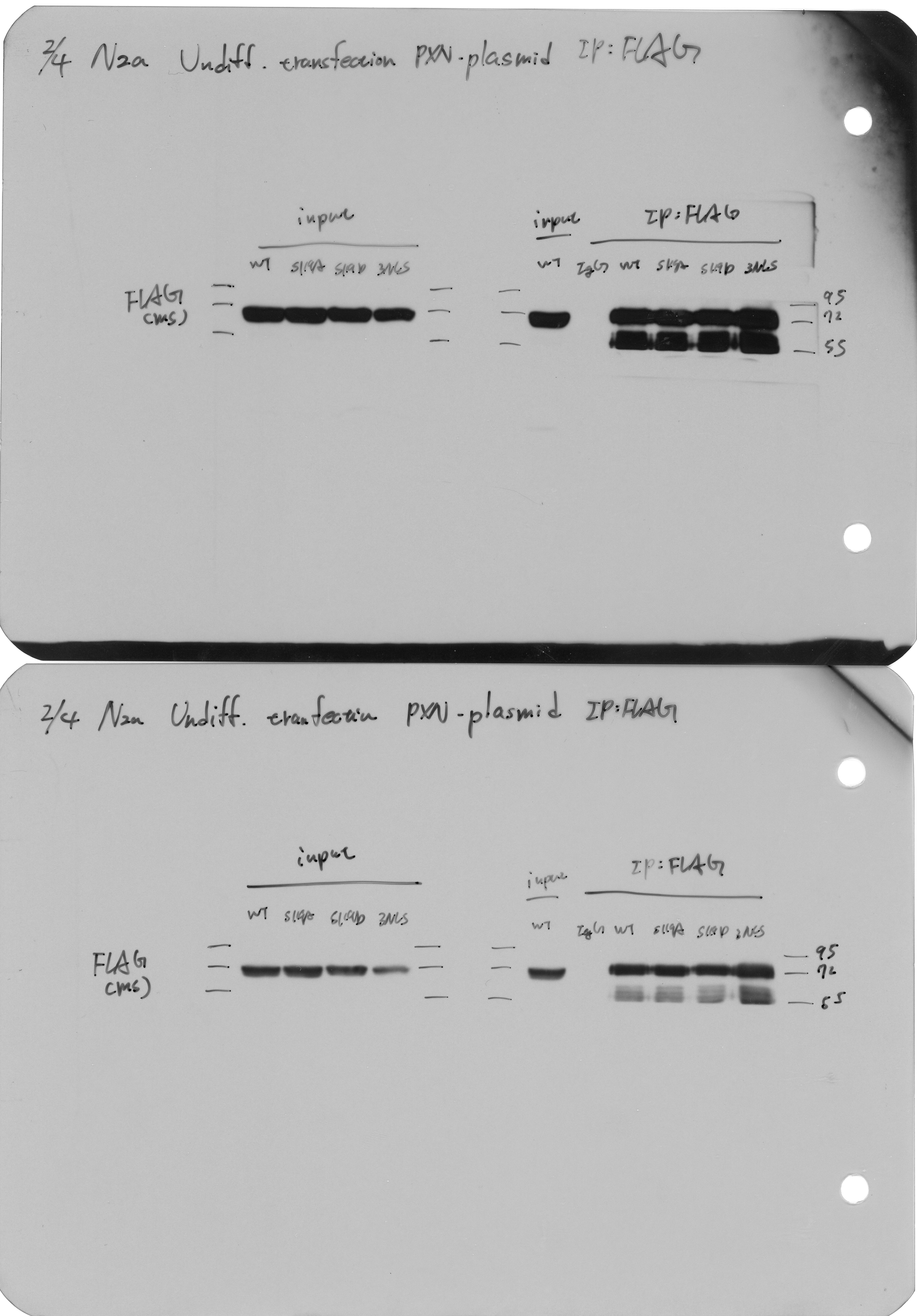

Supplement: Supplementary file 3 — Source data Fig. 1 [file 44318_2025_560_MOESM3_ESM.zip › Figure1/1K/IP-FLAG western_FLAG-3.tif]

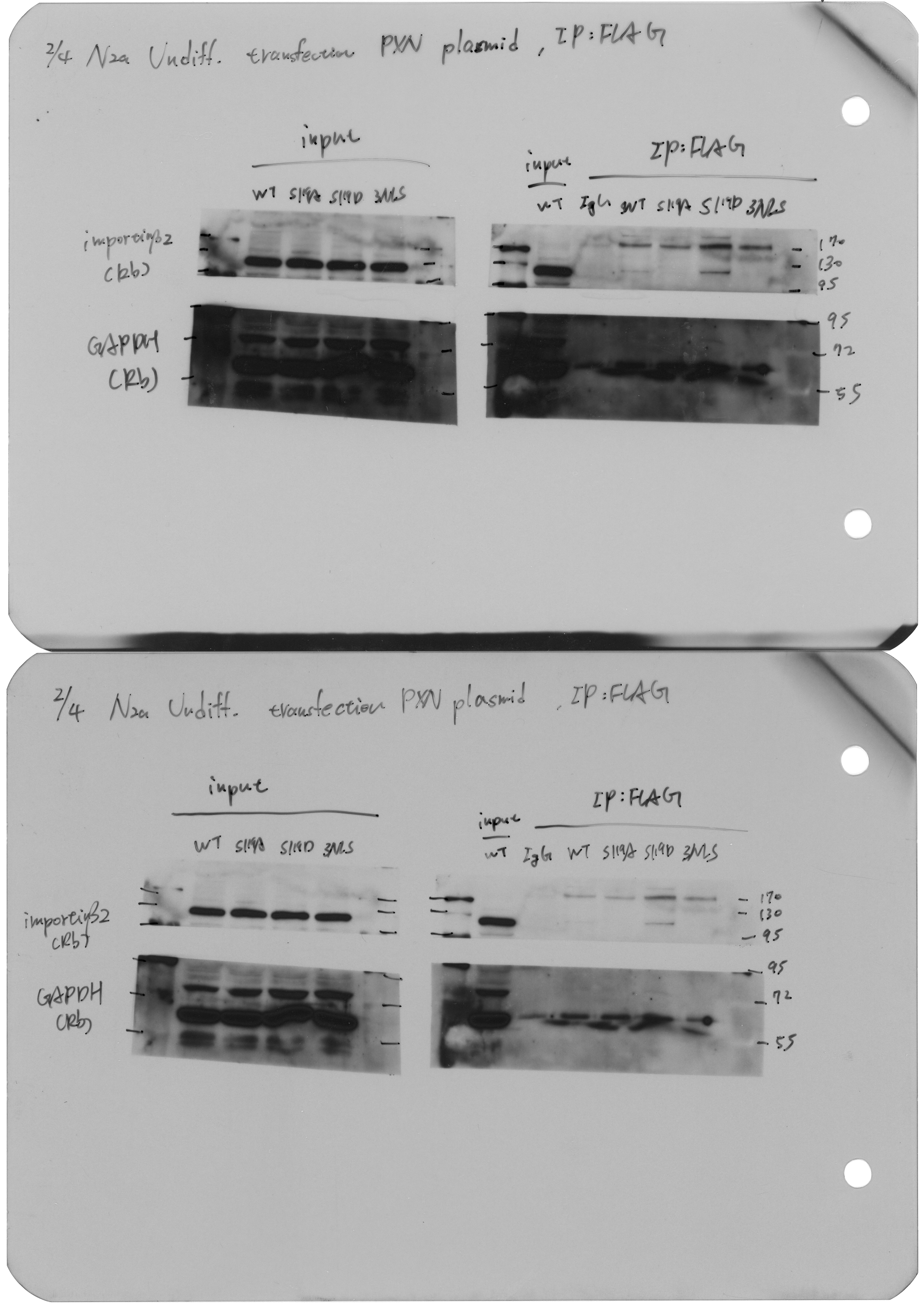

Supplement: Supplementary file 3 — Source data Fig. 1 [file 44318_2025_560_MOESM3_ESM.zip › Figure1/1K/IP-FLAG western_importinB_GAPDH-1.tif]

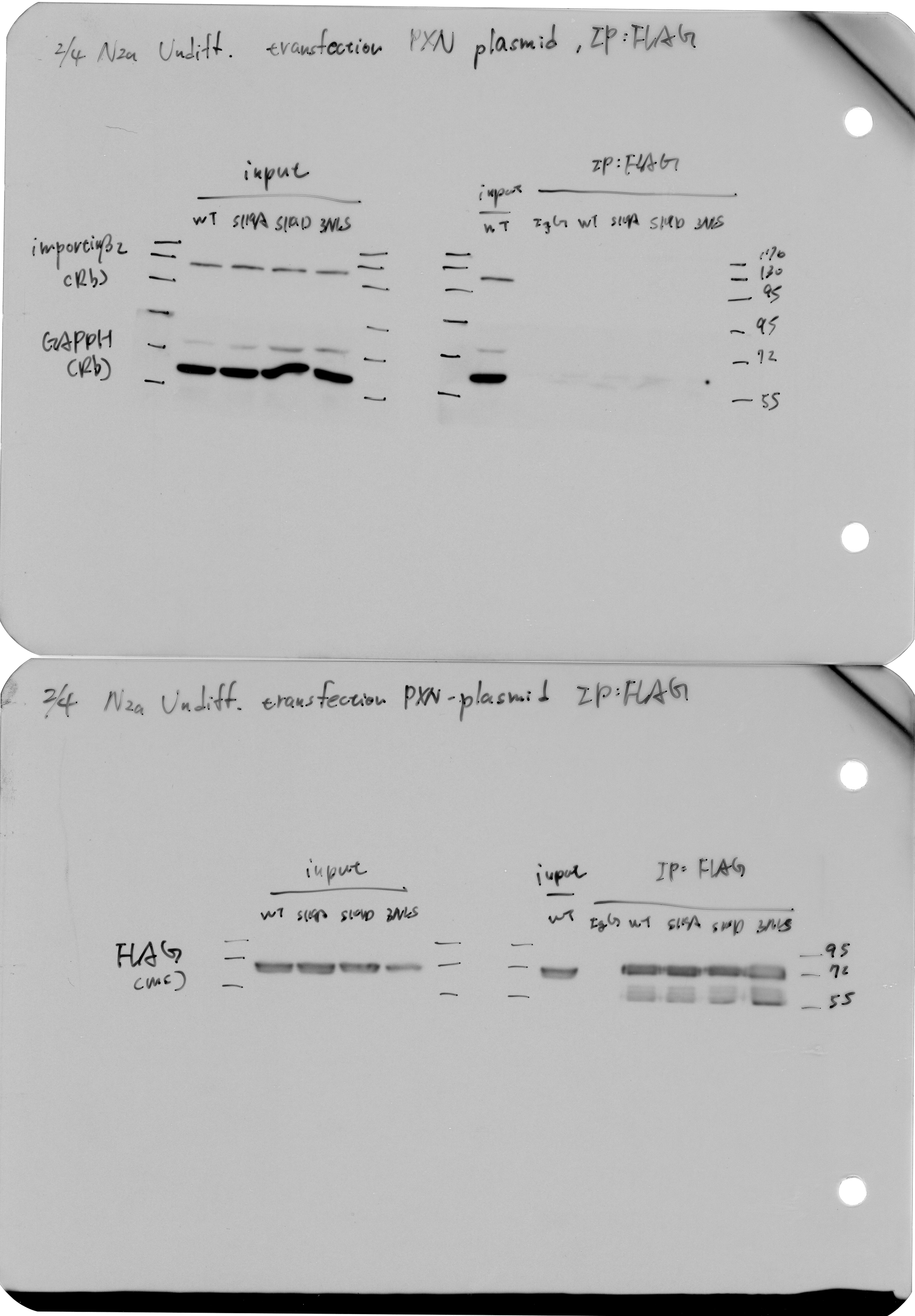

Supplement: Supplementary file 3 — Source data Fig. 1 [file 44318_2025_560_MOESM3_ESM.zip › Figure1/1K/IP-FLAG western_importinB_GAPDH-2.tif]

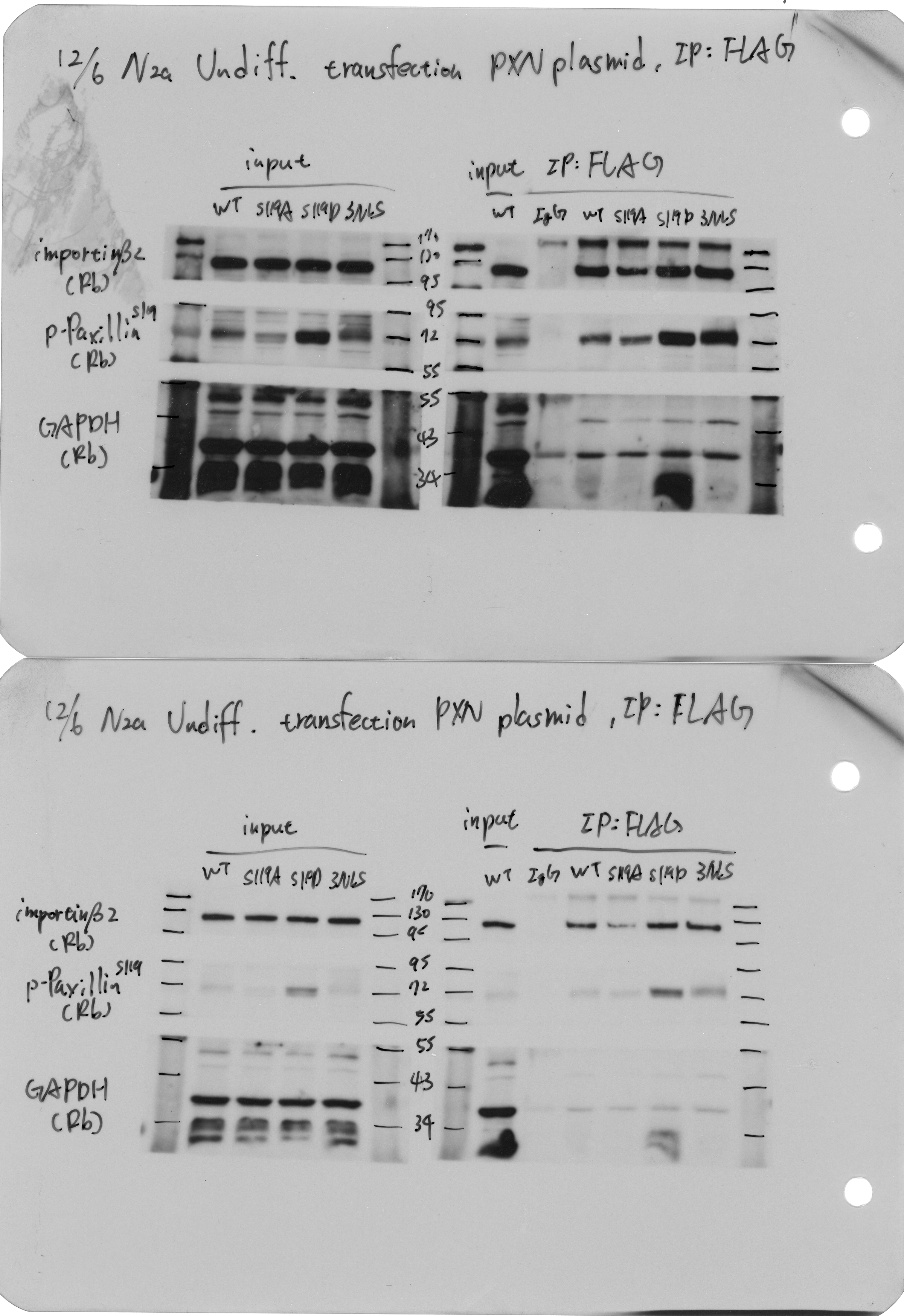

Supplement: Supplementary file 3 — Source data Fig. 1 [file 44318_2025_560_MOESM3_ESM.zip › Figure1/1K/IP-FLAG western_importinB_p-PaxillinS119_GAPDH-1.tif]

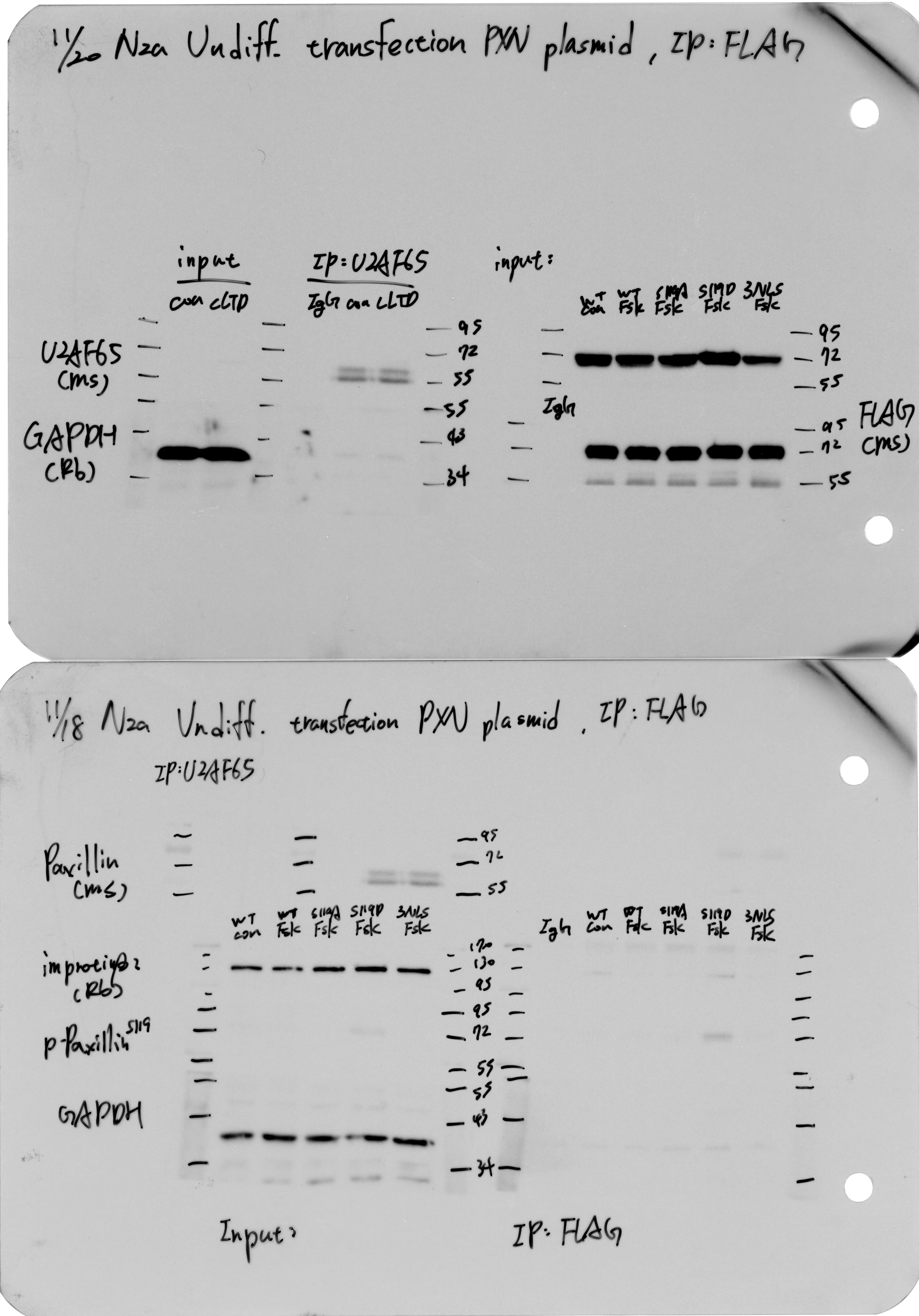

Supplement: Supplementary file 3 — Source data Fig. 1 [file 44318_2025_560_MOESM3_ESM.zip › Figure1/1K/IP-FLAG western_importinB_p-PaxillinS119_GAPDH-2.tif]

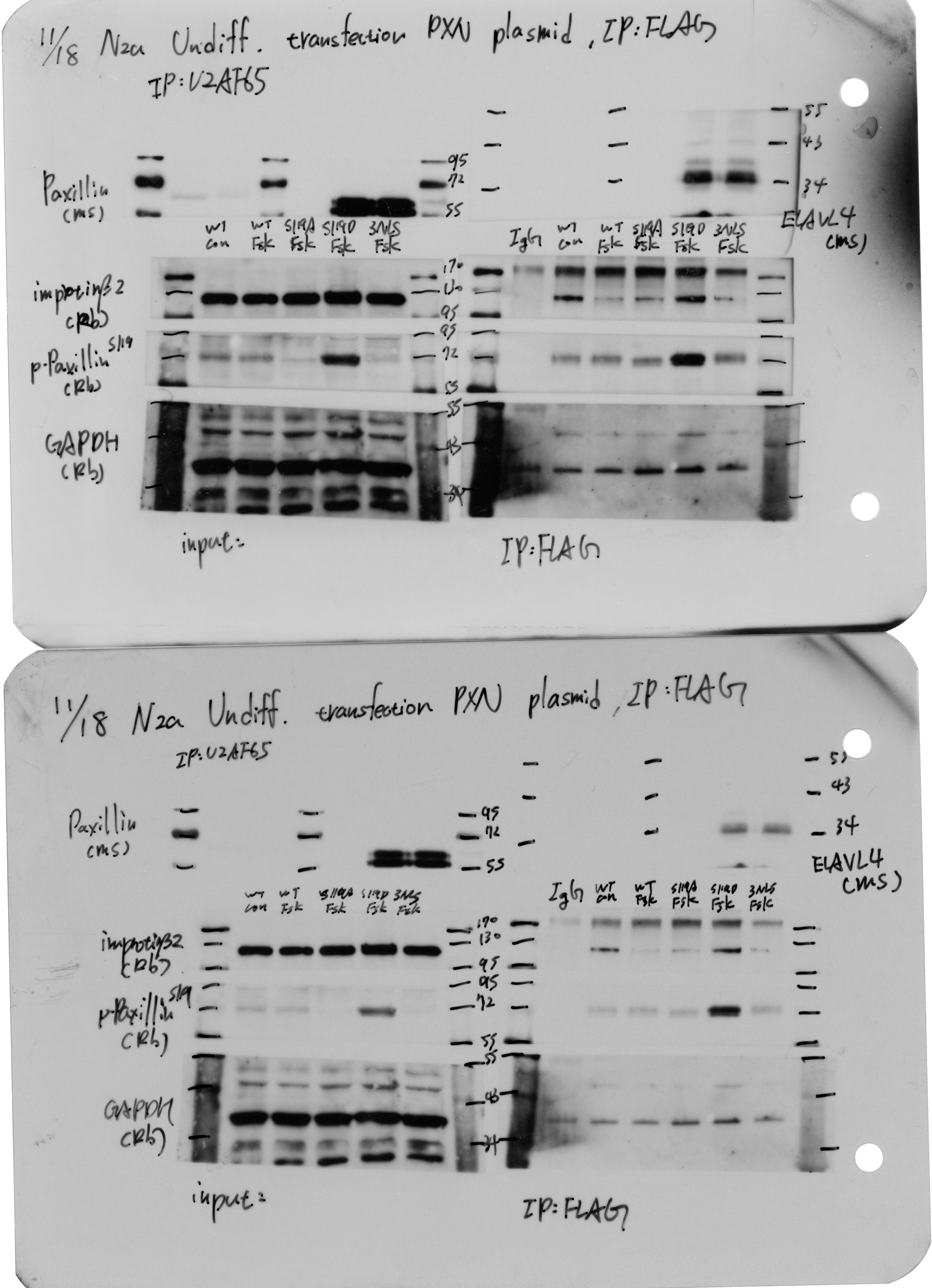

Supplement: Supplementary file 3 — Source data Fig. 1 [file 44318_2025_560_MOESM3_ESM.zip › Figure1/1K/IP-FLAG western_importinB_p-PaxillinS119_GAPDH-3.tif]

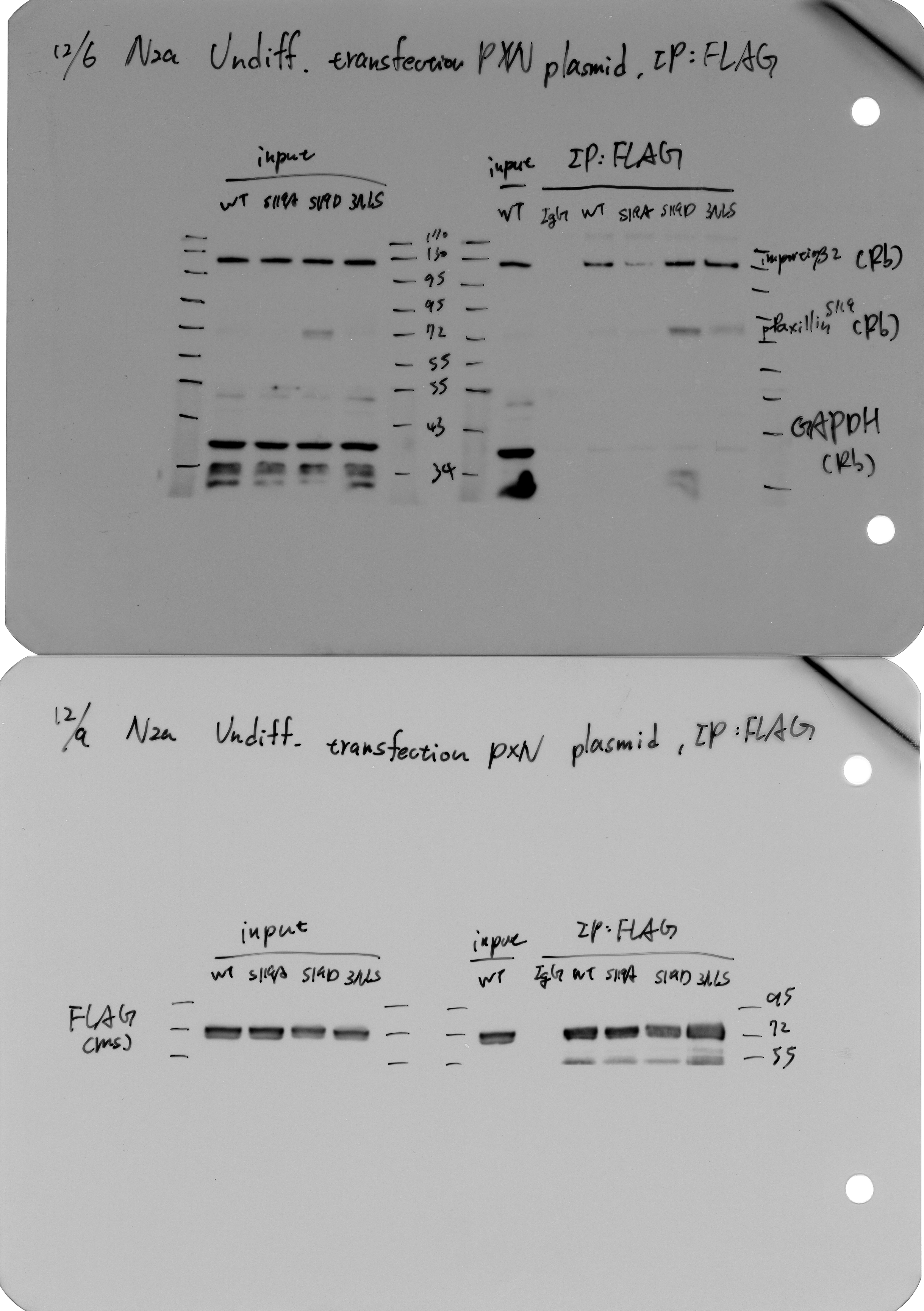

Supplement: Supplementary file 3 — Source data Fig. 1 [file 44318_2025_560_MOESM3_ESM.zip › Figure1/1K/IP-FLAG western_importinB_p-PaxillinS119_GAPDH-4.tif]

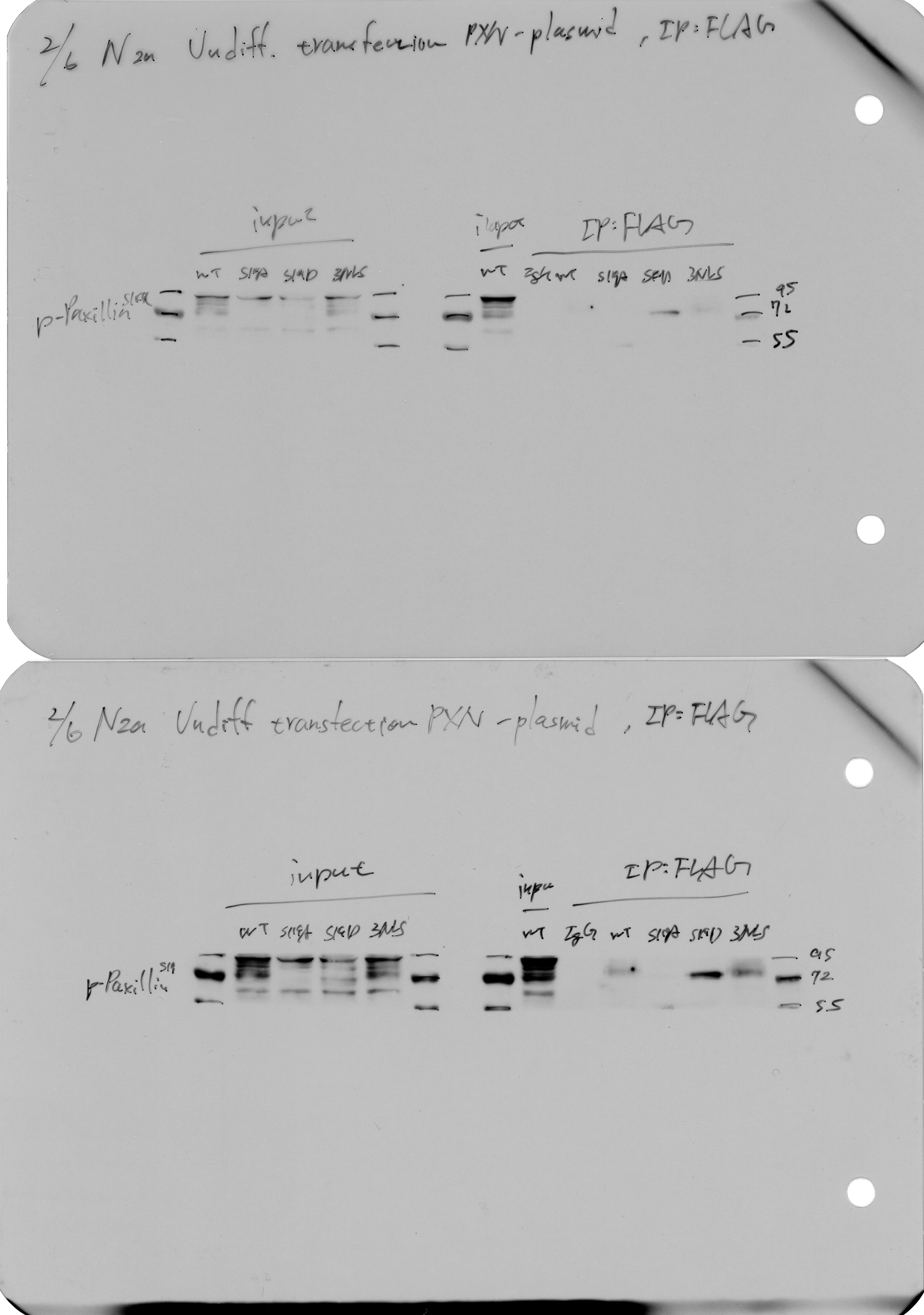

Supplement: Supplementary file 3 — Source data Fig. 1 [file 44318_2025_560_MOESM3_ESM.zip › Figure1/1K/IP-FLAG western_p-PaxillinS119.tif]

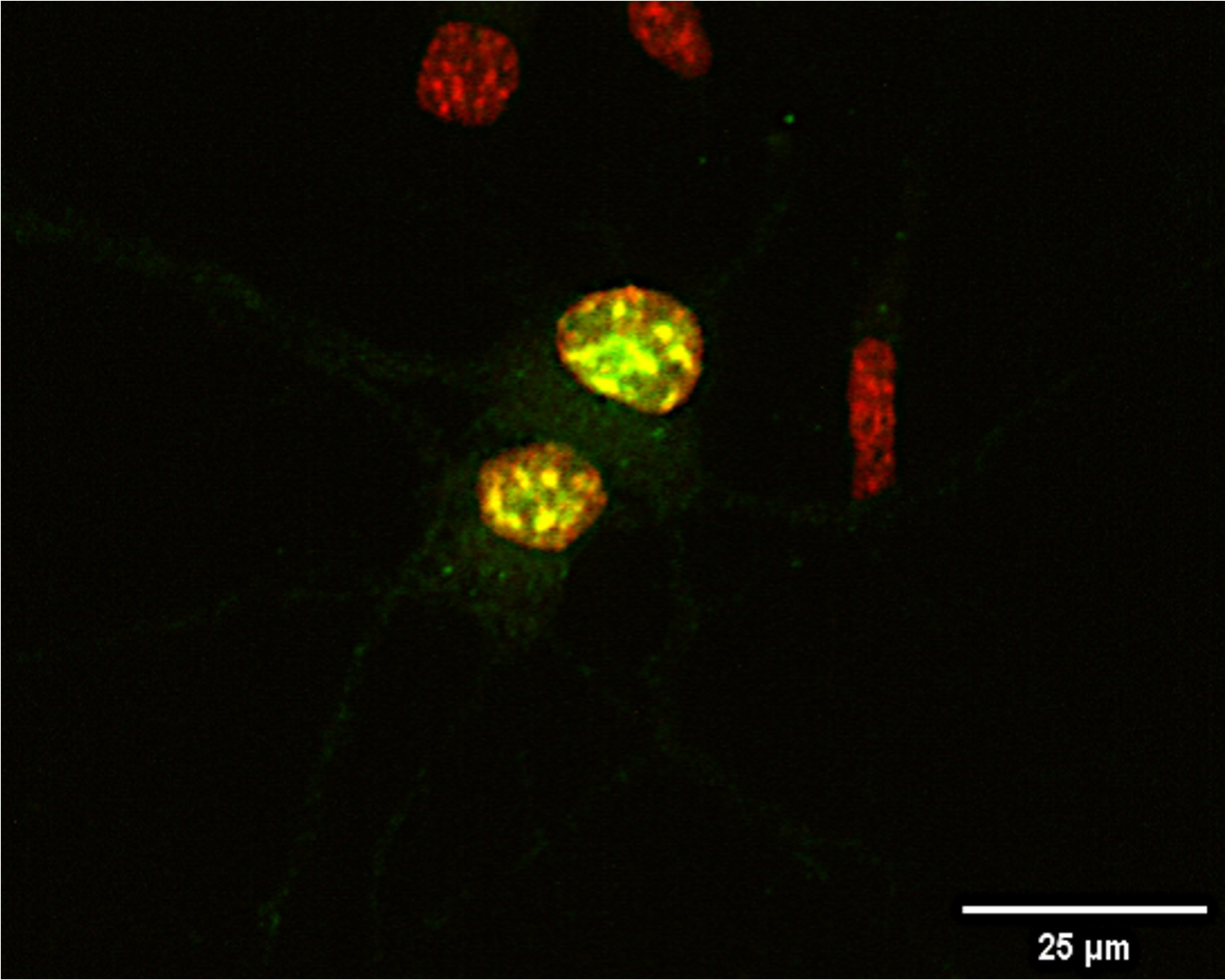

Supplement: Supplementary file 3 — Source data Fig. 1 [file 44318_2025_560_MOESM3_ESM.zip › Figure1/1D/Figure 1D_p-SR_p-PXNS119_merge.tif]

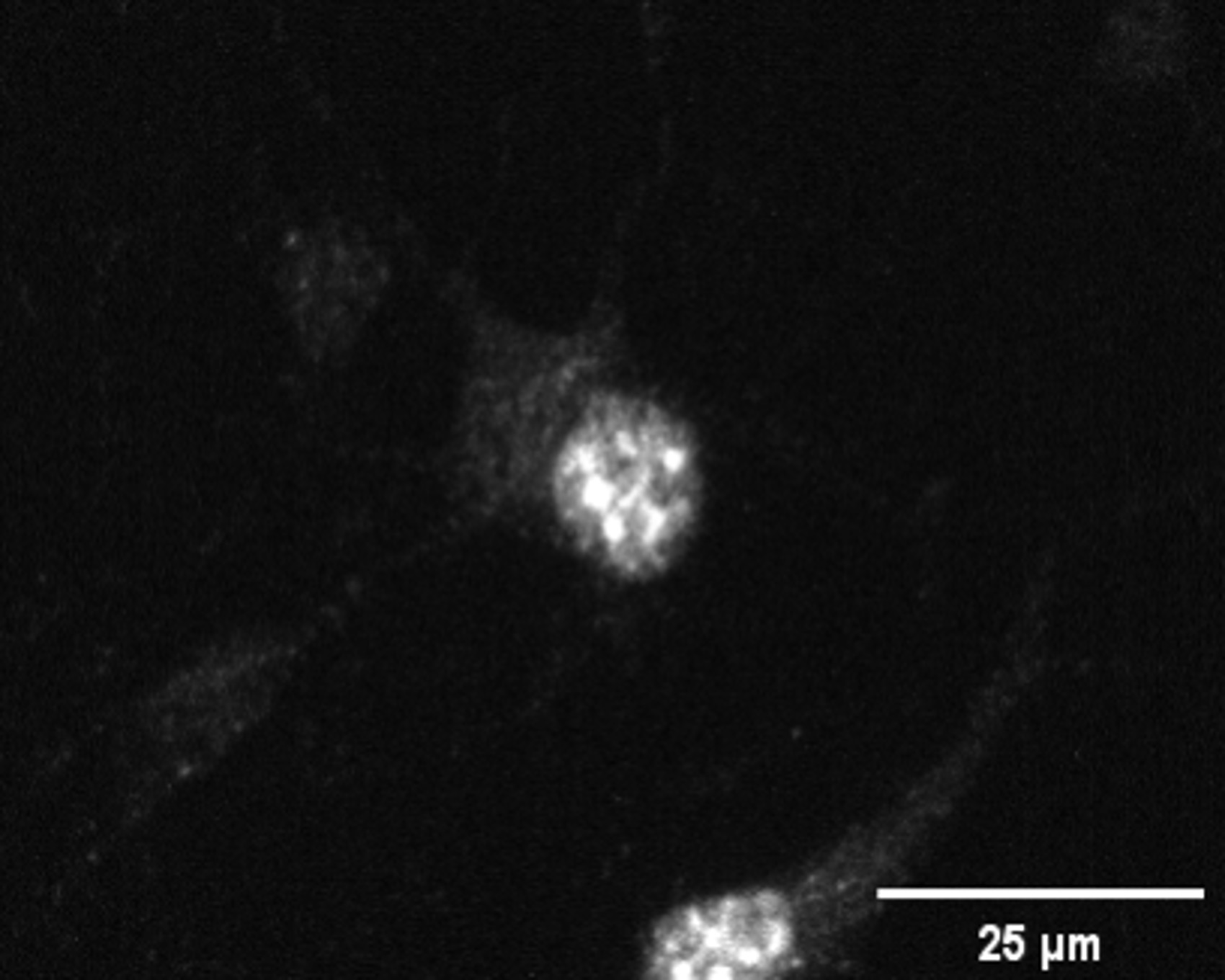

Supplement: Supplementary file 3 — Source data Fig. 1 [file 44318_2025_560_MOESM3_ESM.zip › Figure1/1D/Figure 1D_SC35_p-PXNS119_p-PXNS119.tif]

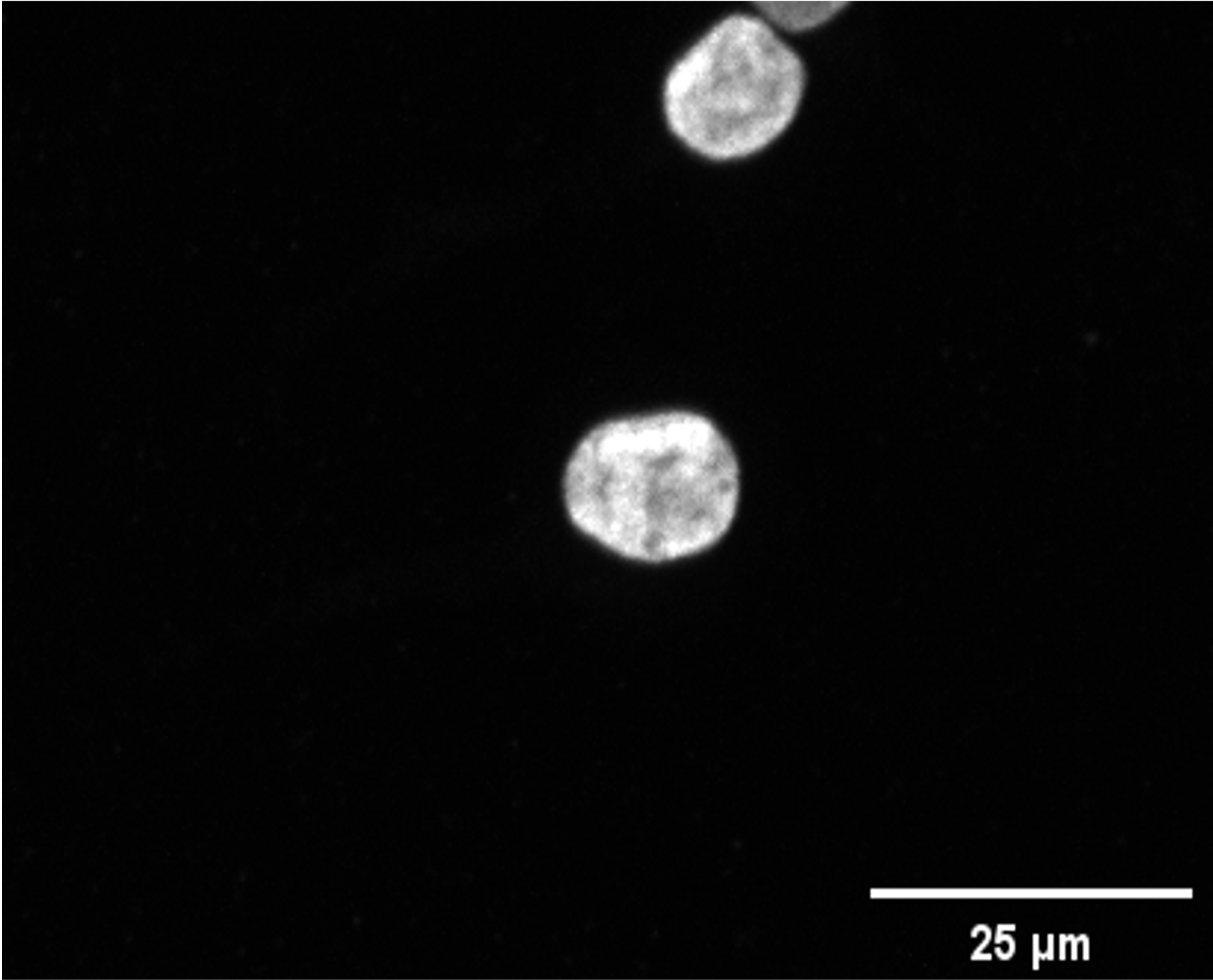

Supplement: Supplementary file 3 — Source data Fig. 1 [file 44318_2025_560_MOESM3_ESM.zip › Figure1/1D/Figure 1D_Histon H3_p-PXNS119_Histon H3.tif]

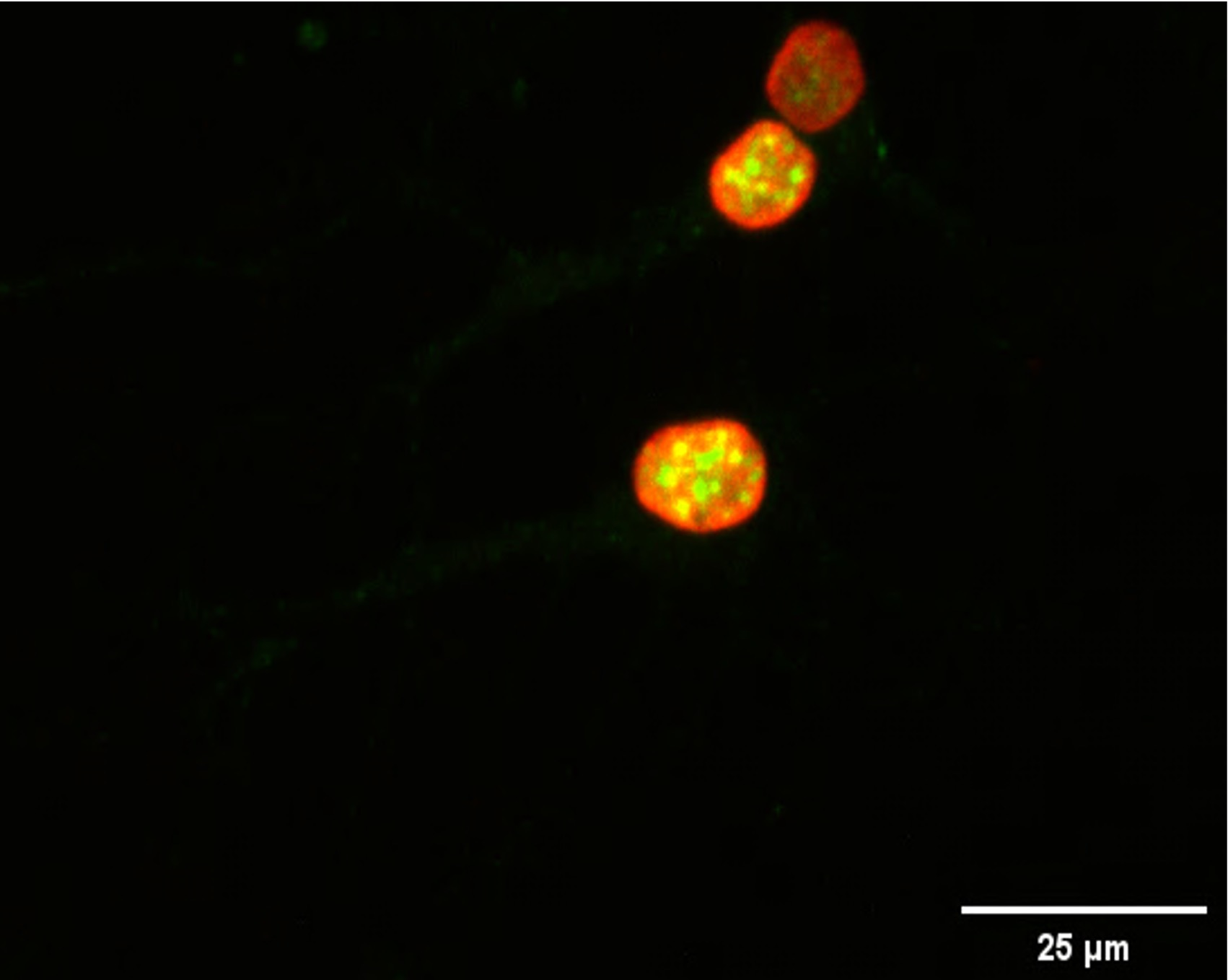

Supplement: Supplementary file 3 — Source data Fig. 1 [file 44318_2025_560_MOESM3_ESM.zip › Figure1/1D/Figure 1D_Histon H3_p-PXNS119_Merge.tif]

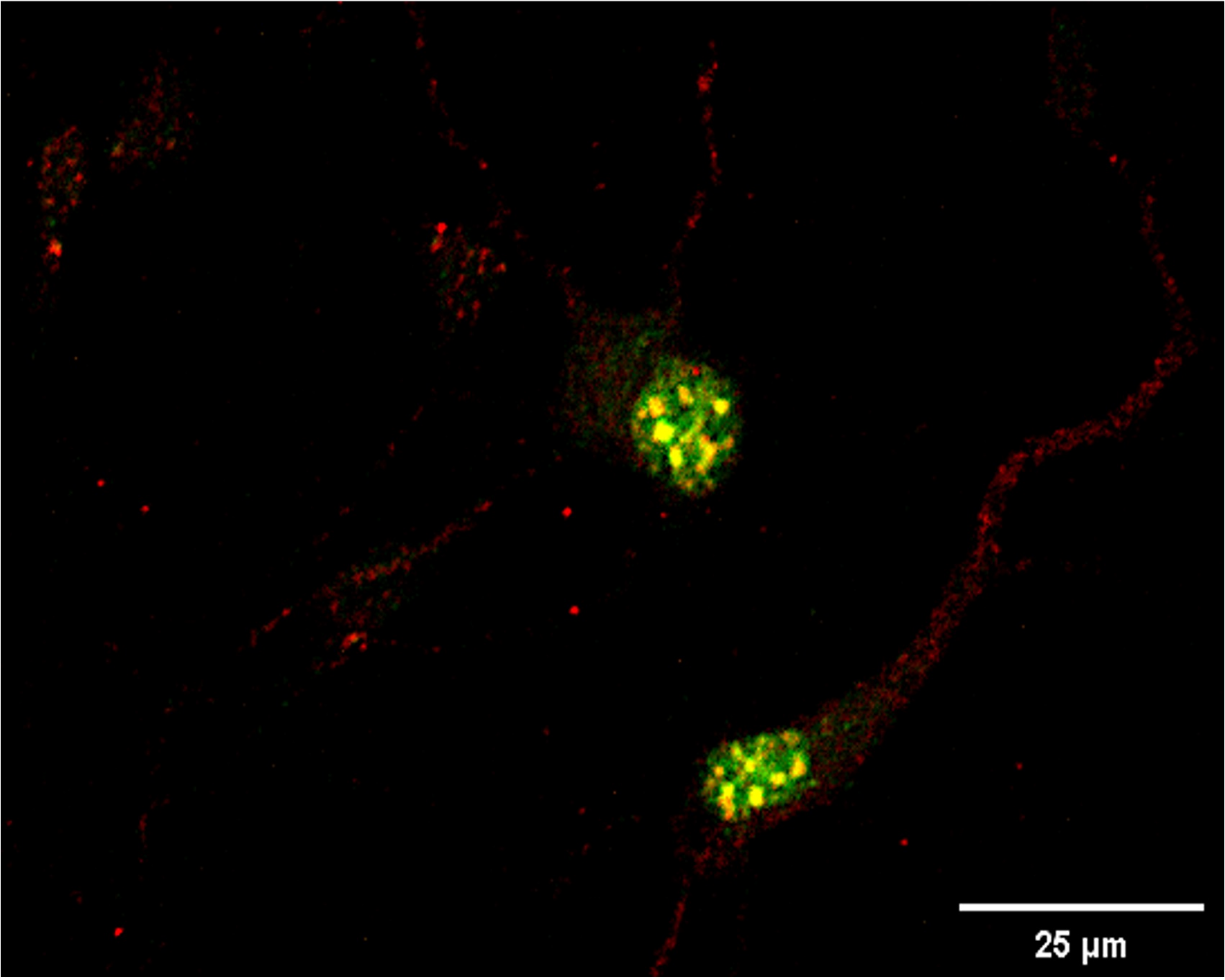

Supplement: Supplementary file 3 — Source data Fig. 1 [file 44318_2025_560_MOESM3_ESM.zip › Figure1/1D/Figure 1D_SC35_p-PXNS119_merge.tif]

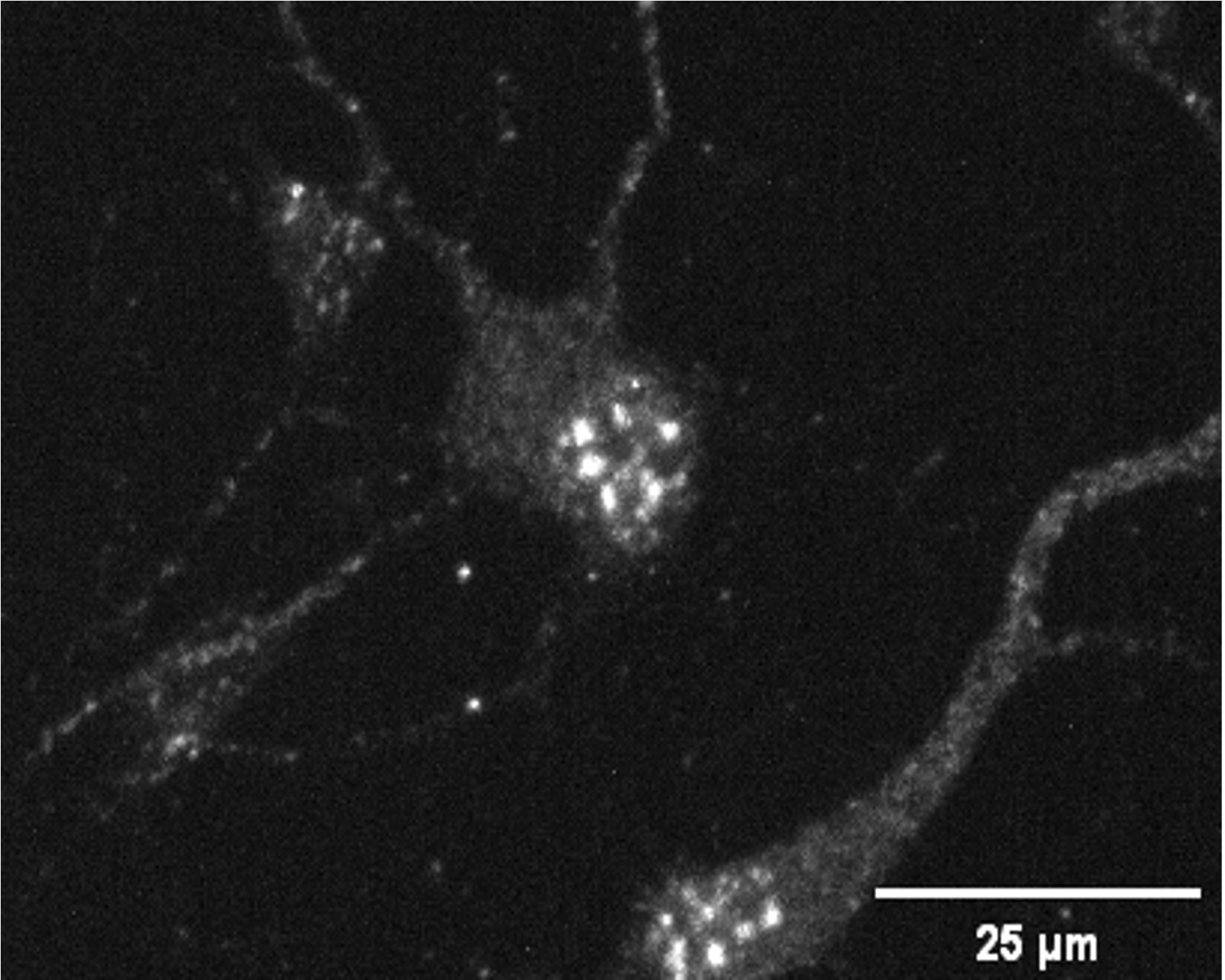

Supplement: Supplementary file 3 — Source data Fig. 1 [file 44318_2025_560_MOESM3_ESM.zip › Figure1/1D/Figure 1D_SC35_p-PXNS119_SC35.tif]

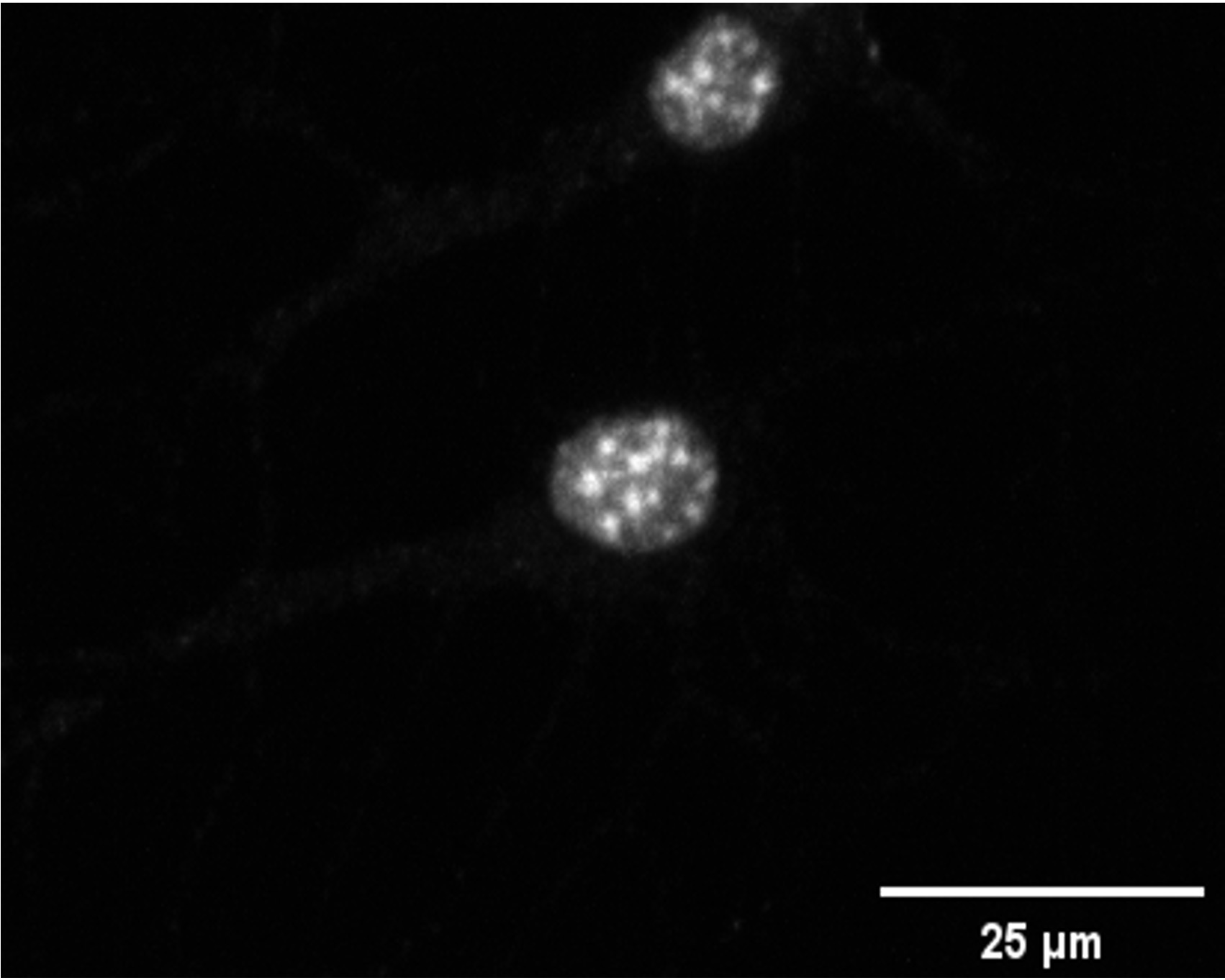

Supplement: Supplementary file 3 — Source data Fig. 1 [file 44318_2025_560_MOESM3_ESM.zip › Figure1/1D/Figure 1D_Histon H3_p-PXNS119_p-PXNS119.tif]

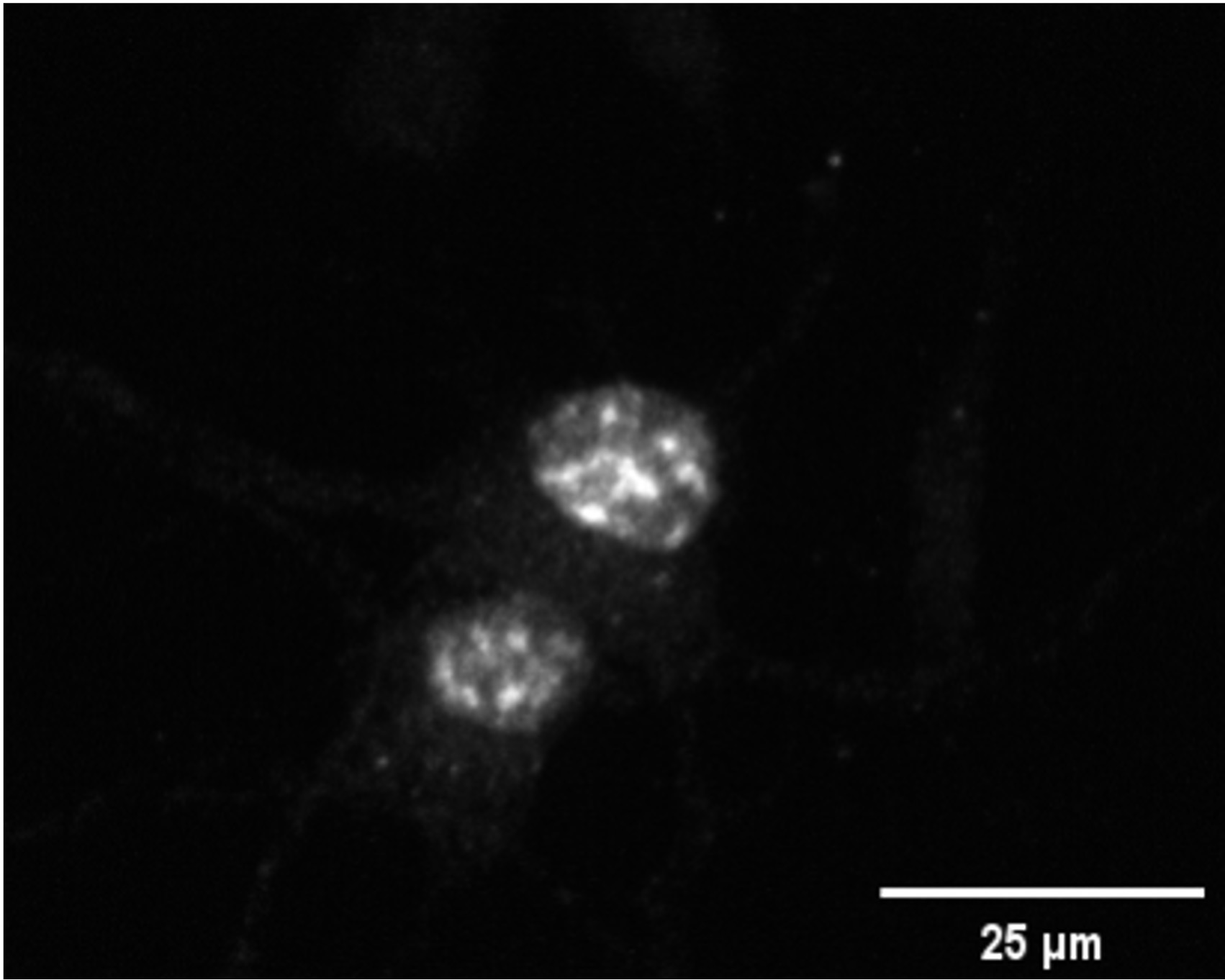

Supplement: Supplementary file 3 — Source data Fig. 1 [file 44318_2025_560_MOESM3_ESM.zip › Figure1/1D/Figure 1D_p-SR_p-PXNS119_p-PXNS119.tif]

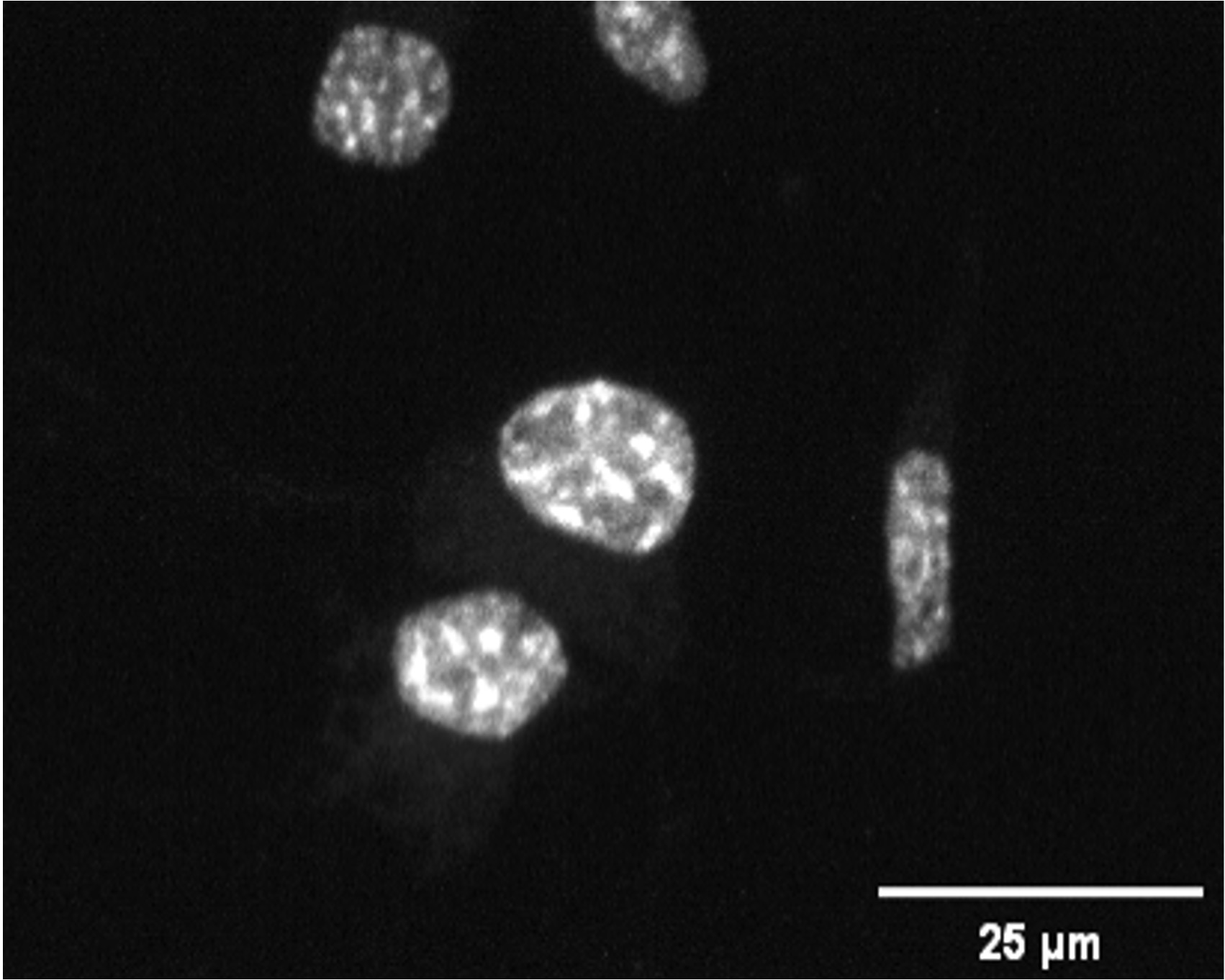

Supplement: Supplementary file 3 — Source data Fig. 1 [file 44318_2025_560_MOESM3_ESM.zip › Figure1/1D/Figure 1D_p-SR_p-PXNS119_p-SR.tif]

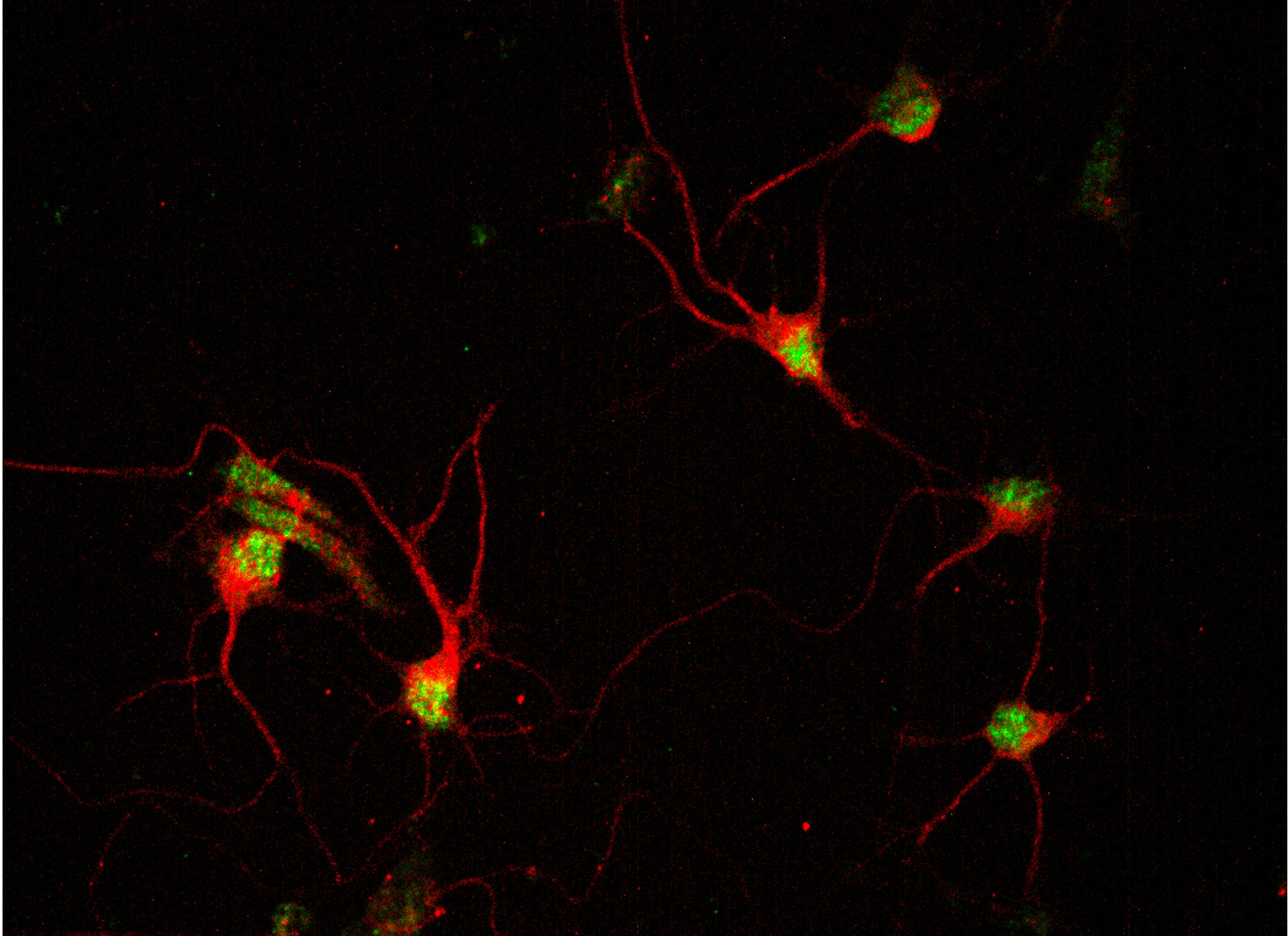

Supplement: Supplementary file 3 — Source data Fig. 1 [file 44318_2025_560_MOESM3_ESM.zip › Figure1/1E/Figure 1E_control_p-SR_p-PXNS119_merge.tif]

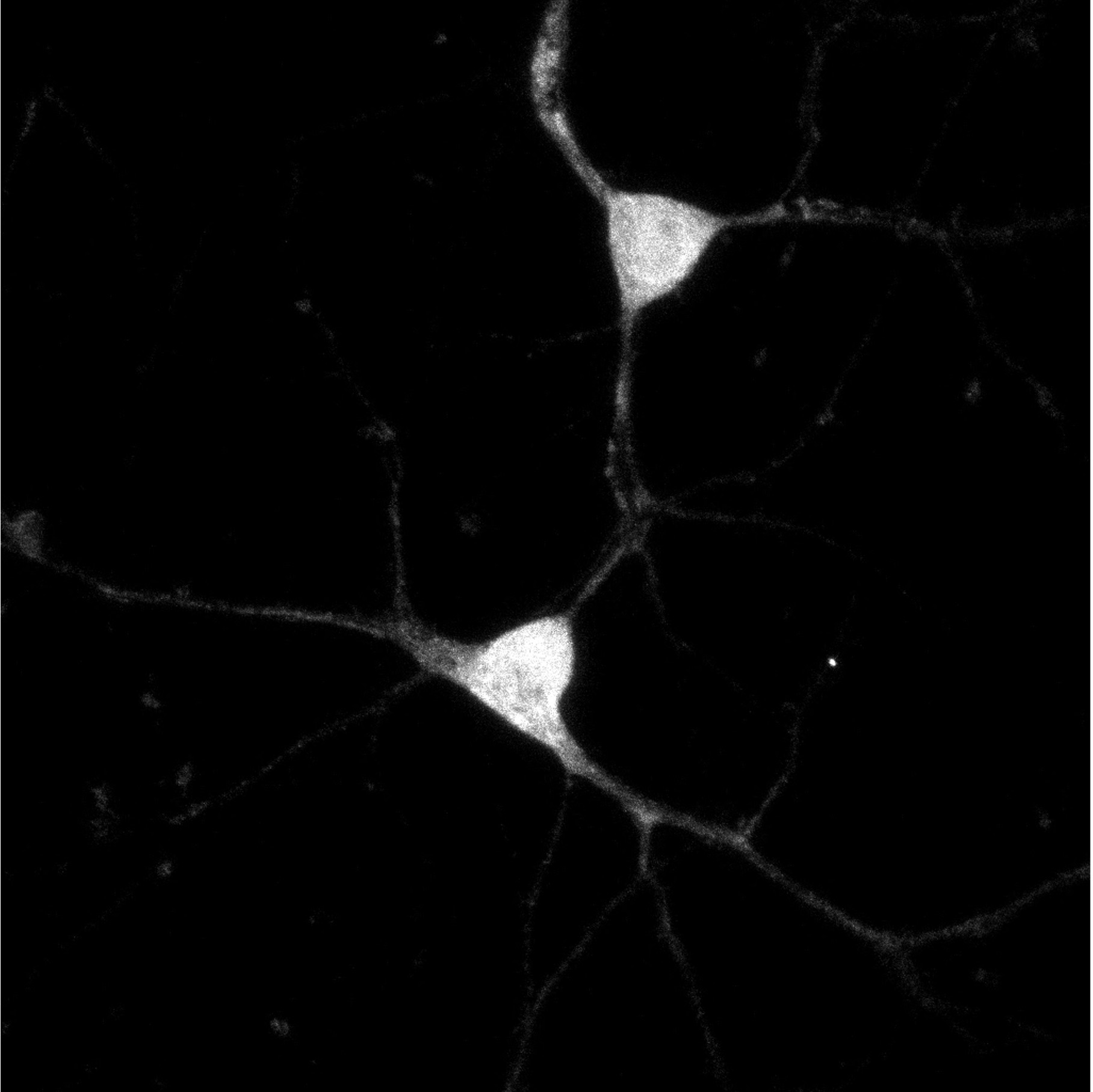

Supplement: Supplementary file 3 — Source data Fig. 1 [file 44318_2025_560_MOESM3_ESM.zip › Figure1/1E/Figure 2C_DMSO_DIV7 neurons_Tuj-1.tif]

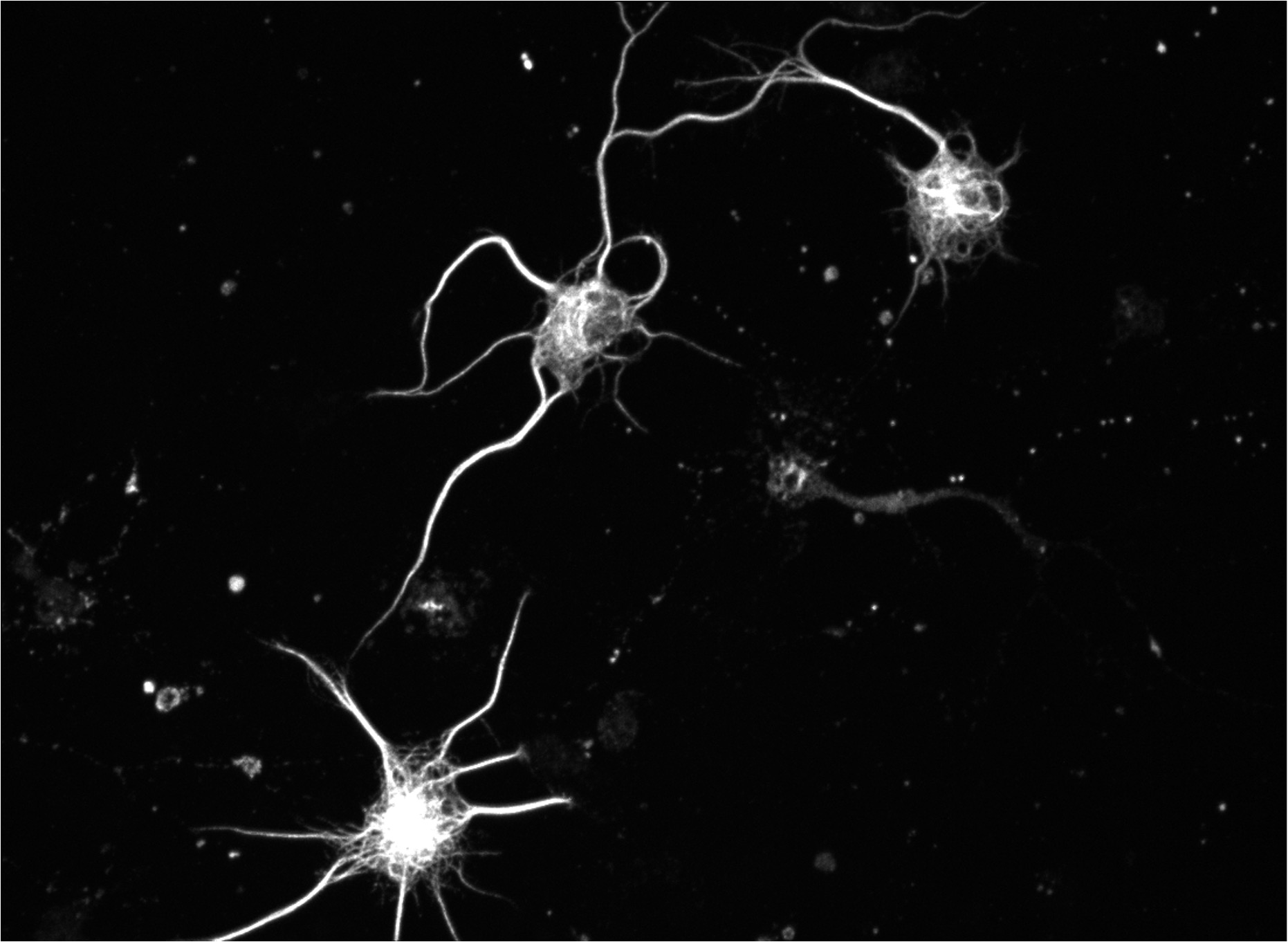

Supplement: Supplementary file 3 — Source data Fig. 1 [file 44318_2025_560_MOESM3_ESM.zip › Figure1/1E/Figure 1E_LMB_p-SR_p-PXNS119_Tuj-1.tif]

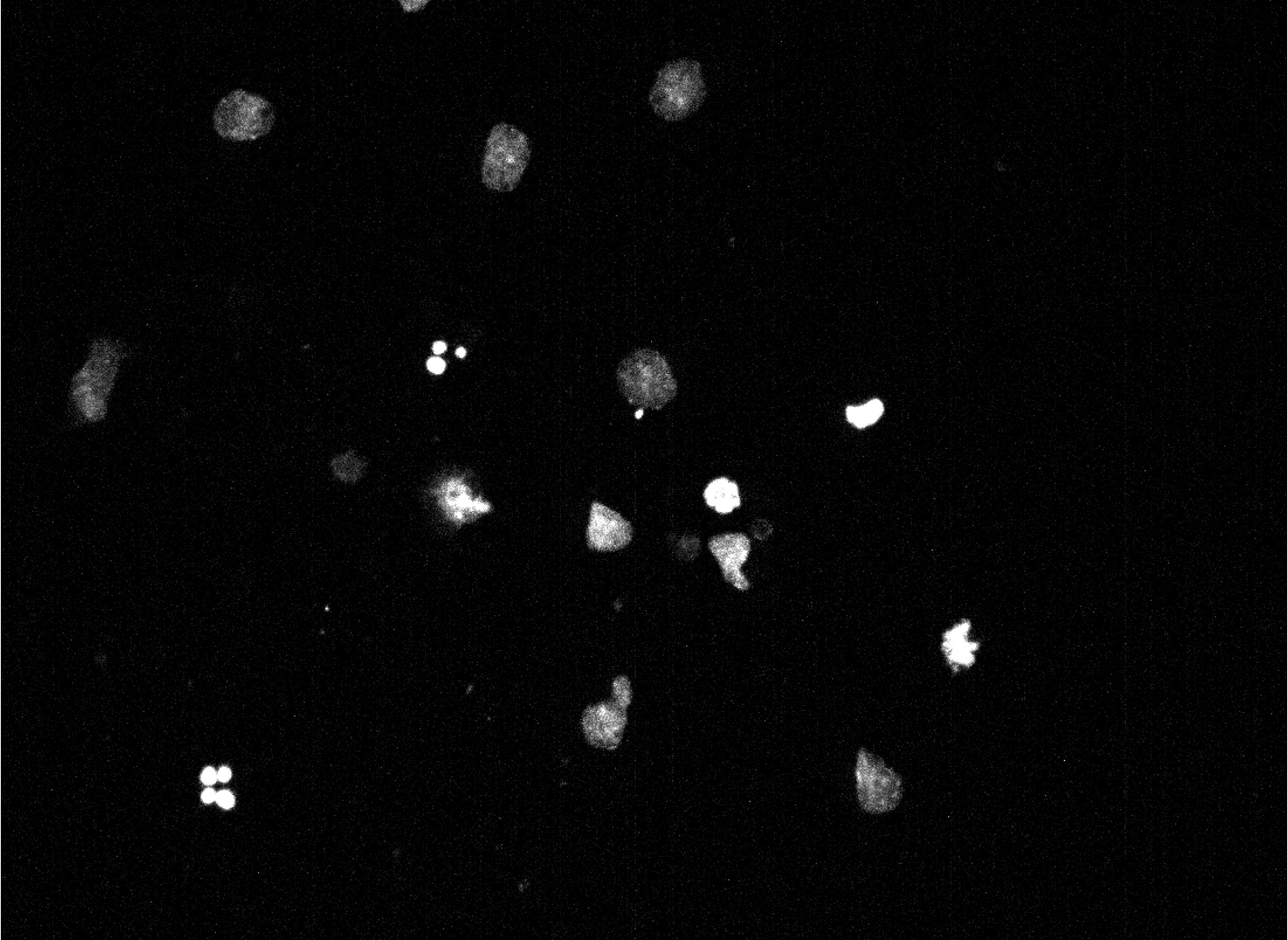

Supplement: Supplementary file 3 — Source data Fig. 1 [file 44318_2025_560_MOESM3_ESM.zip › Figure1/1E/Figure 1E_KPT-330_p-SR_p-PXNS119_DAPI.tif]

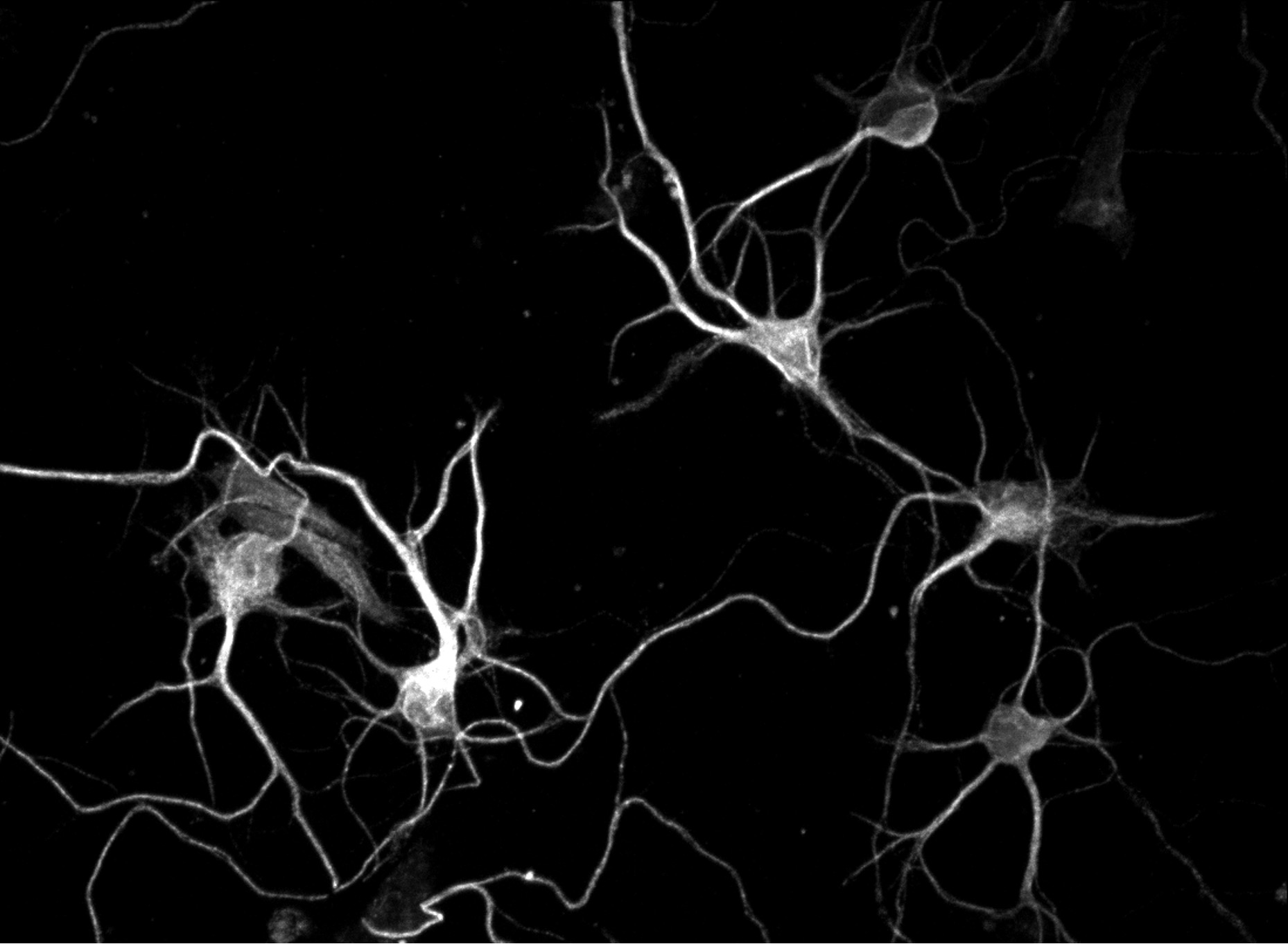

Supplement: Supplementary file 3 — Source data Fig. 1 [file 44318_2025_560_MOESM3_ESM.zip › Figure1/1E/Figure 1E_control_p-SR_p-PXNS119_Tuj-1.tif]

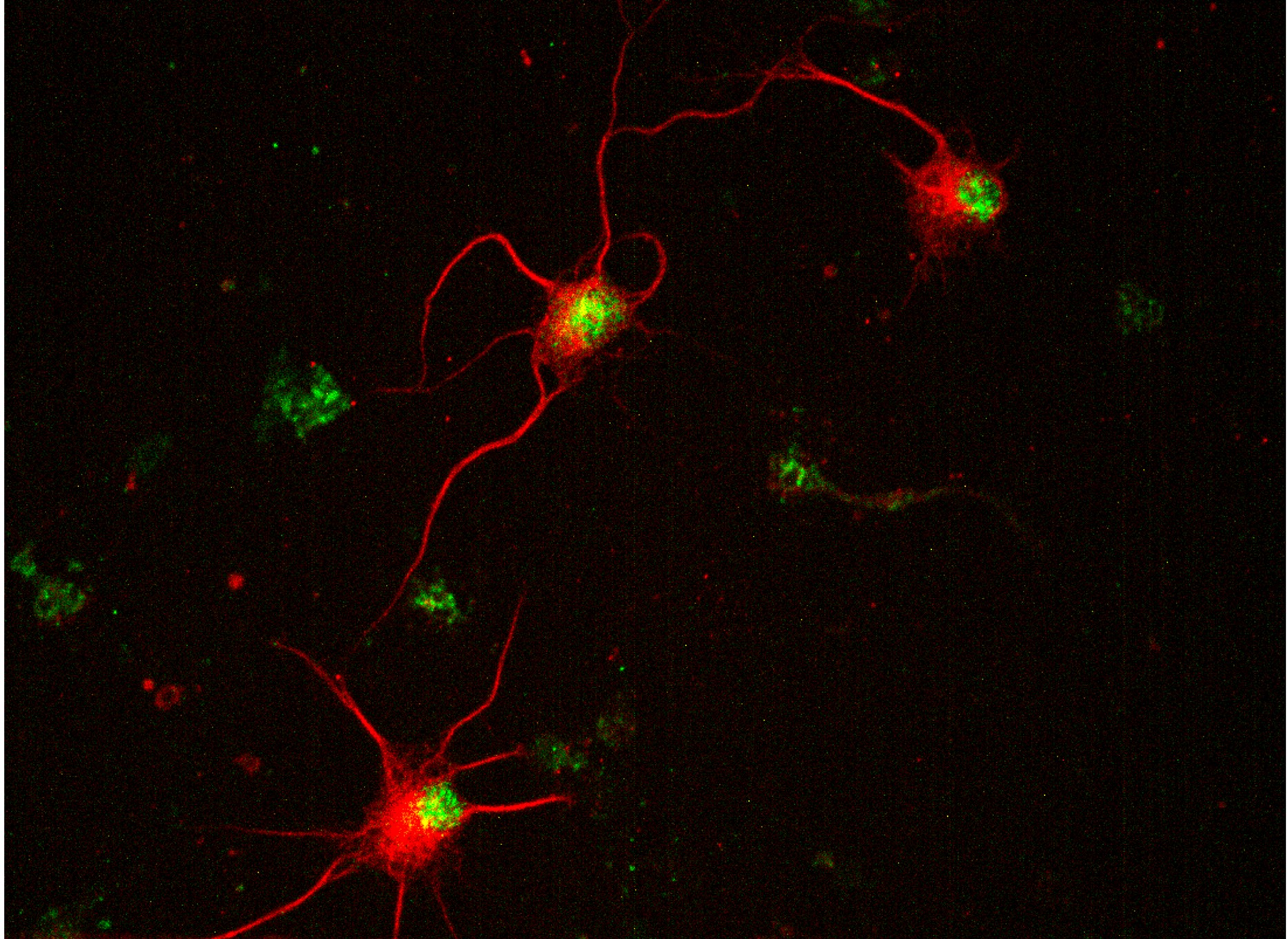

Supplement: Supplementary file 3 — Source data Fig. 1 [file 44318_2025_560_MOESM3_ESM.zip › Figure1/1E/Figure 1E_LMB_p-SR_p-PXNS119_merge.tif]

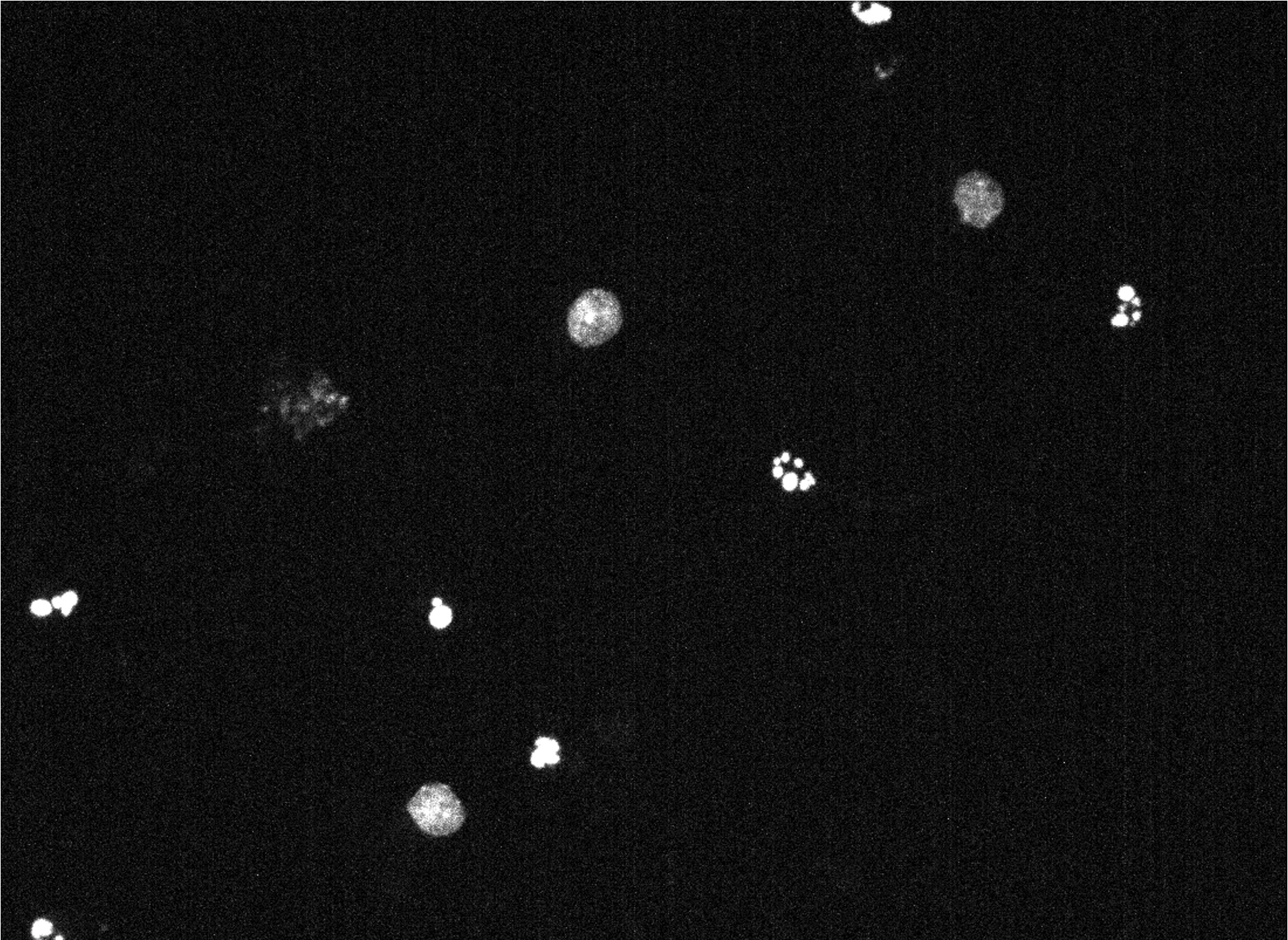

Supplement: Supplementary file 3 — Source data Fig. 1 [file 44318_2025_560_MOESM3_ESM.zip › Figure1/1E/Figure 1E_LMB_p-SR_p-PXNS119_DAPI.tif]

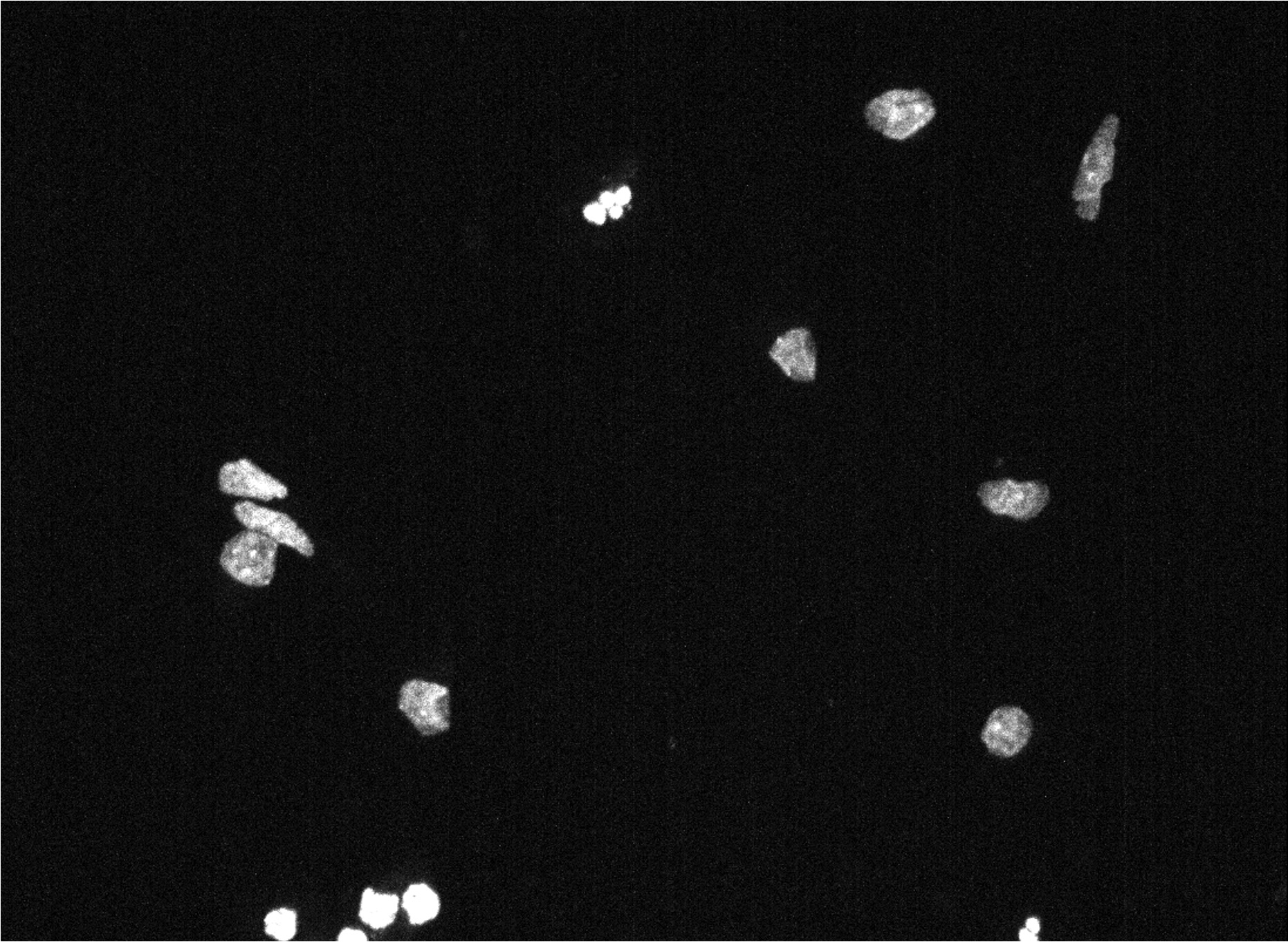

Supplement: Supplementary file 3 — Source data Fig. 1 [file 44318_2025_560_MOESM3_ESM.zip › Figure1/1E/Figure 1E_control_p-SR_p-PXNS119_DAPI.tif]

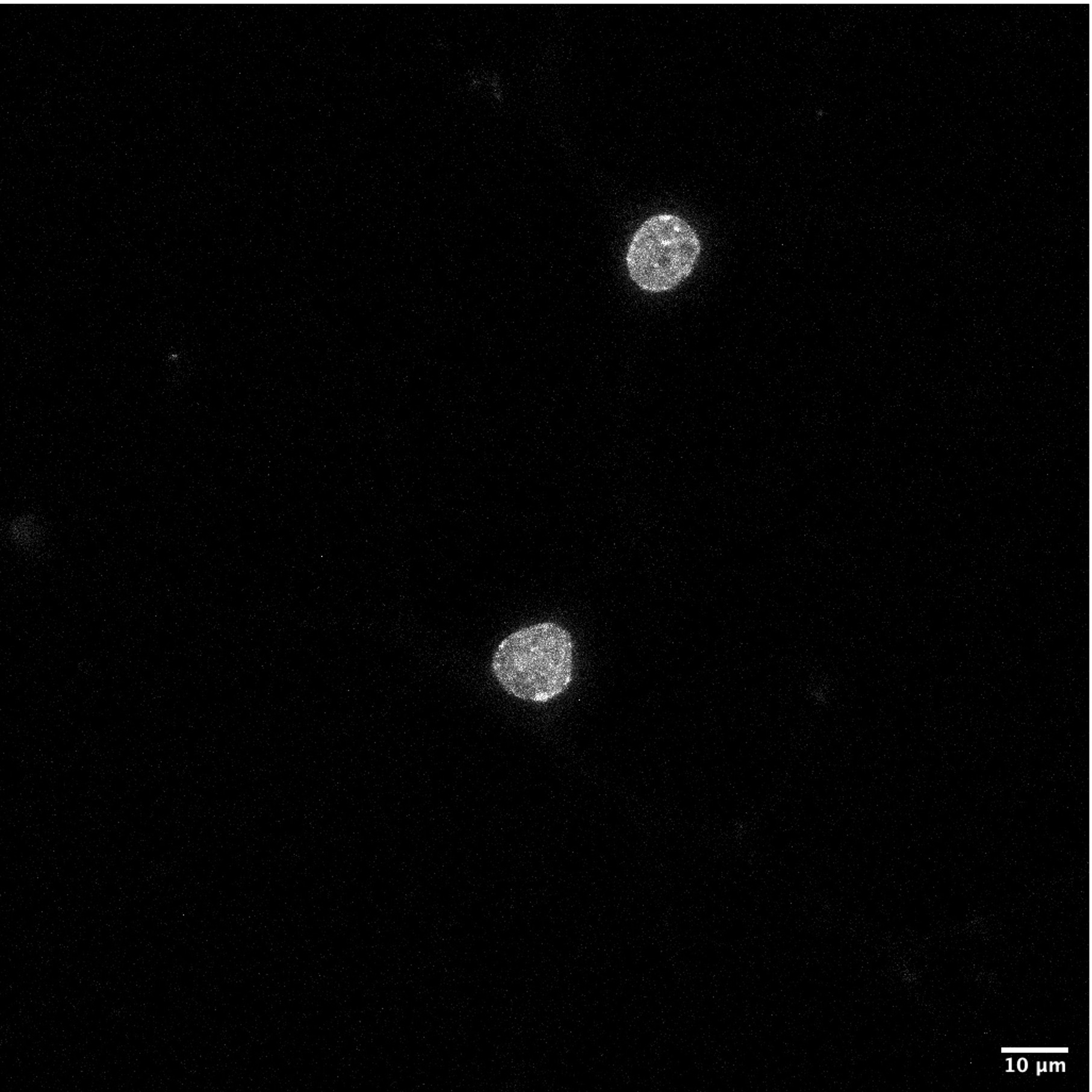

Supplement: Supplementary file 3 — Source data Fig. 1 [file 44318_2025_560_MOESM3_ESM.zip › Figure1/1E/Figure 2C_DMSO_DIV7 neurons_DAPI.tif]

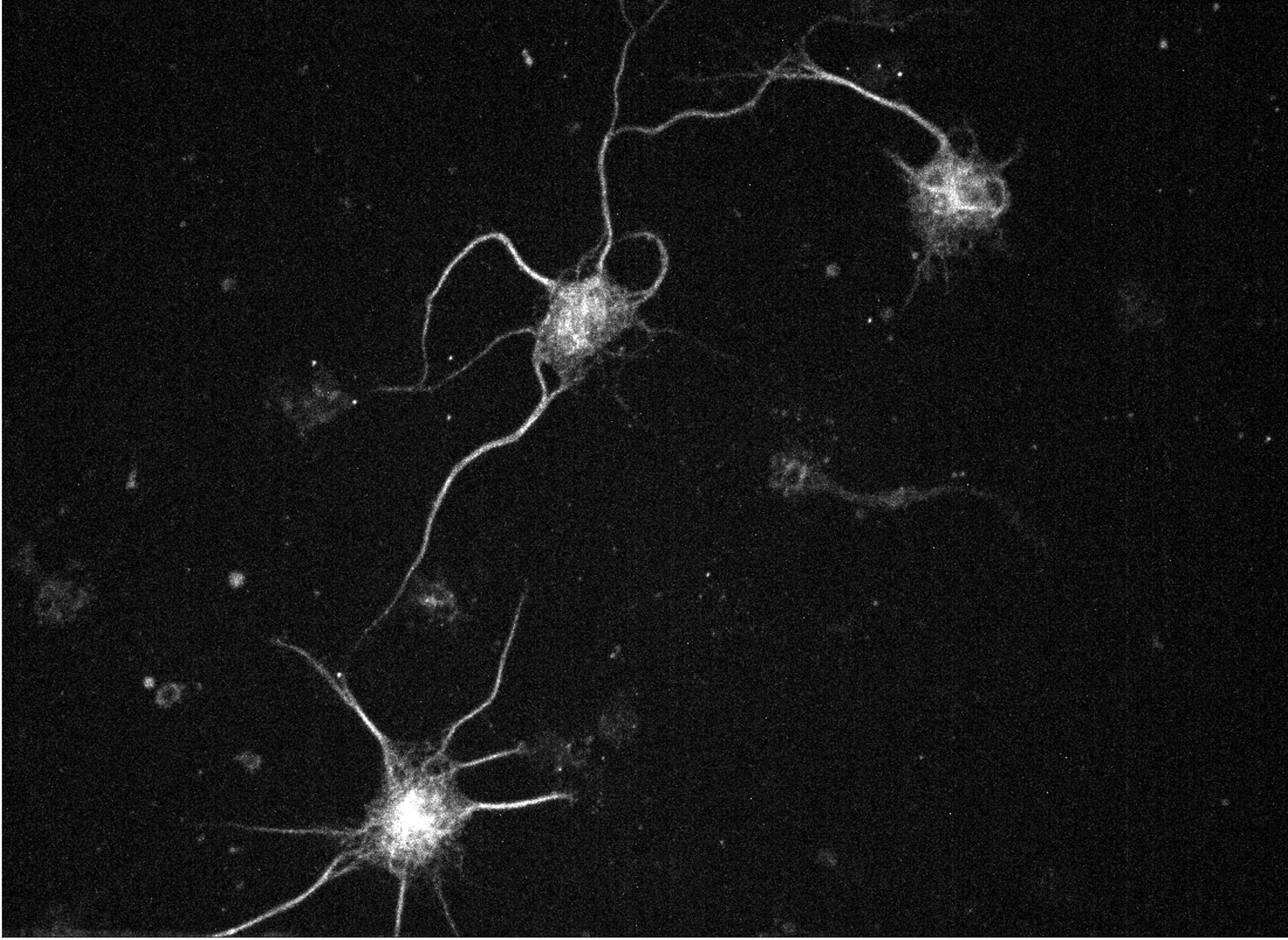

Supplement: Supplementary file 3 — Source data Fig. 1 [file 44318_2025_560_MOESM3_ESM.zip › Figure1/1E/Figure 1E_LMB_p-SR_p-PXNS119_p-PXNS119.tif]

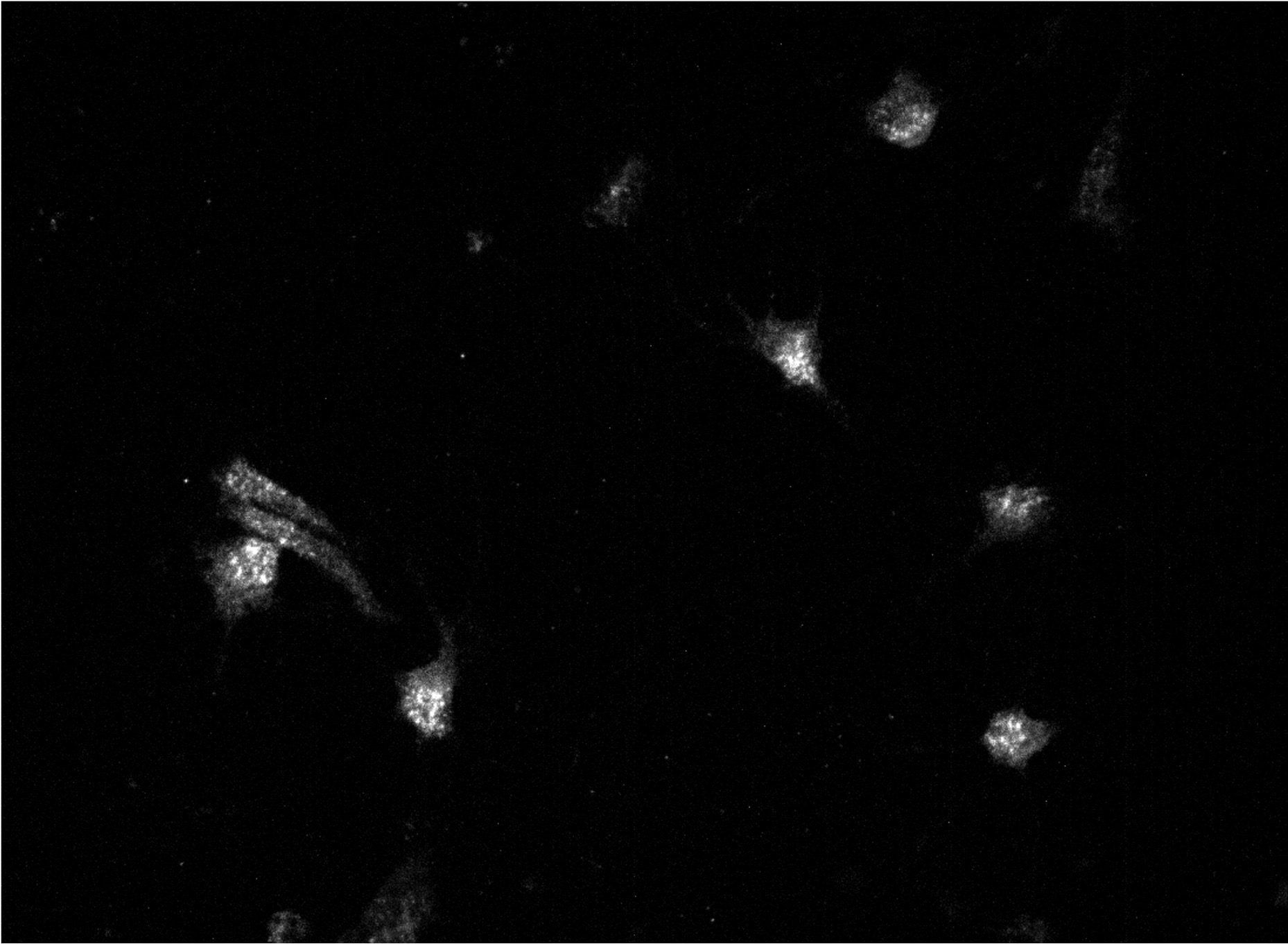

Supplement: Supplementary file 3 — Source data Fig. 1 [file 44318_2025_560_MOESM3_ESM.zip › Figure1/1E/Figure 1E_control_p-SR_p-PXNS119_p-SR.tif]

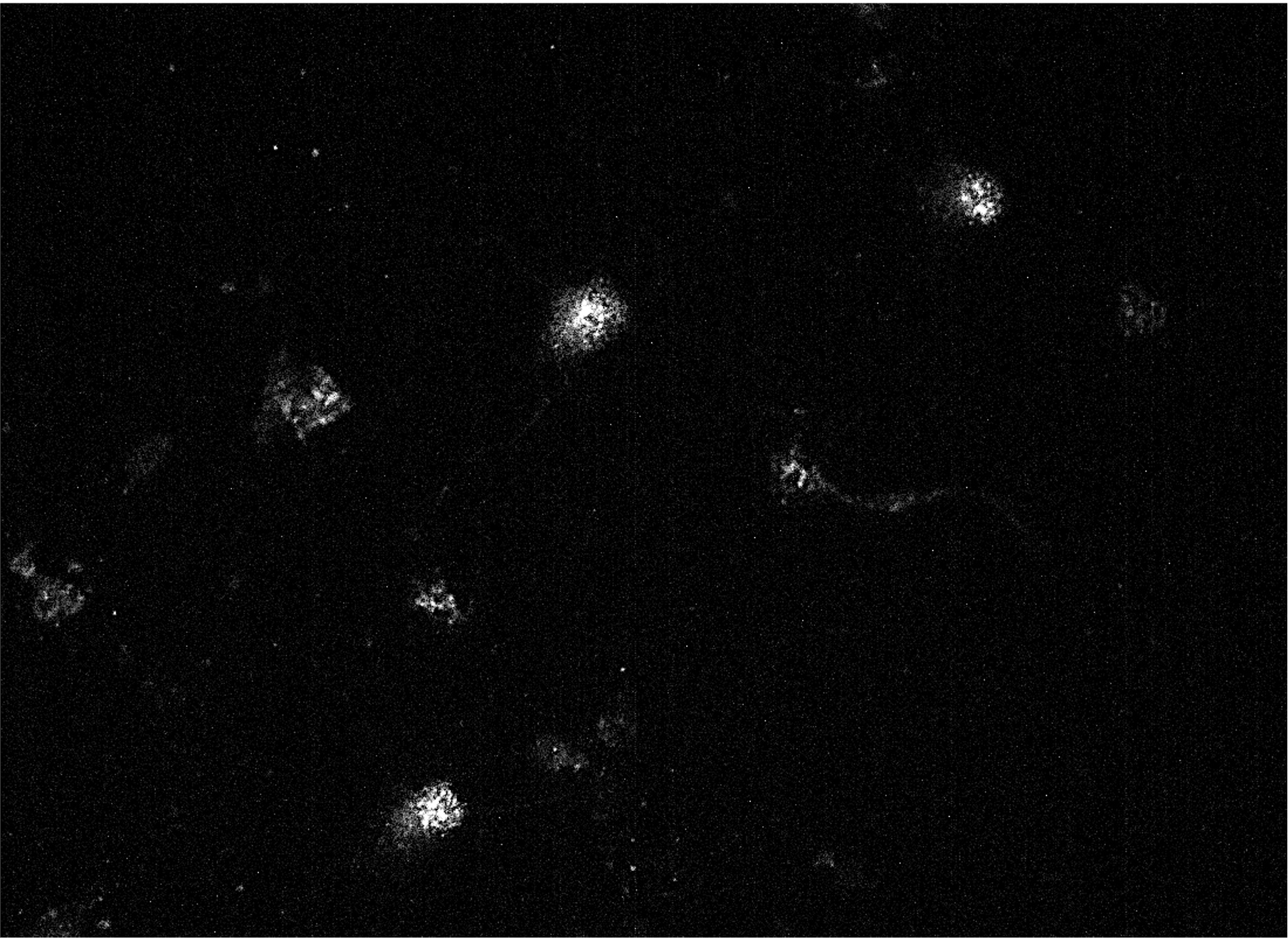

Supplement: Supplementary file 3 — Source data Fig. 1 [file 44318_2025_560_MOESM3_ESM.zip › Figure1/1E/Figure 1E_LMB_p-SR_p-PXNS119_p-SR.tif]

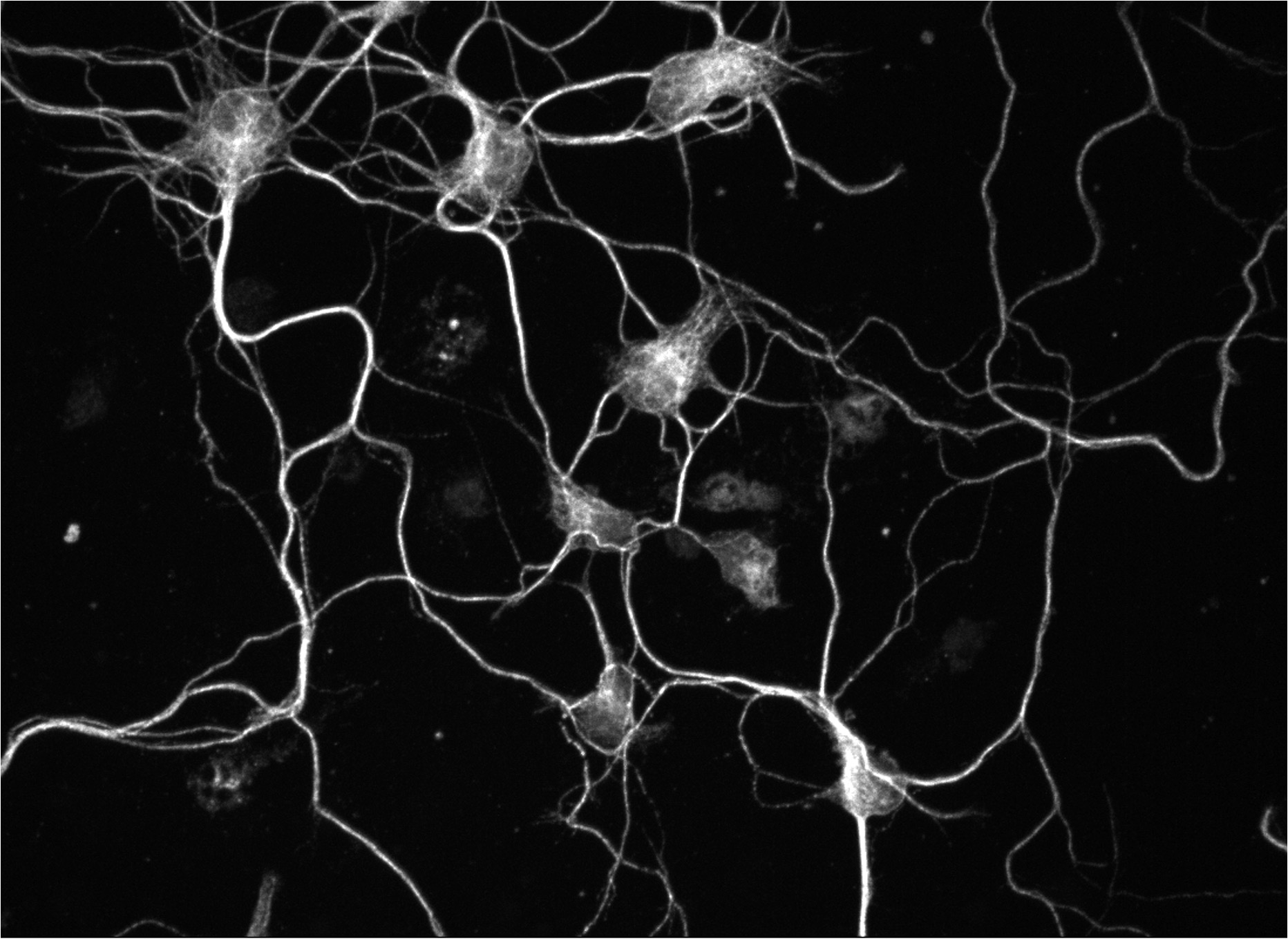

Supplement: Supplementary file 3 — Source data Fig. 1 [file 44318_2025_560_MOESM3_ESM.zip › Figure1/1E/Figure 1E_KPT-330_p-SR_p-PXNS119_Tuj-1.tif]

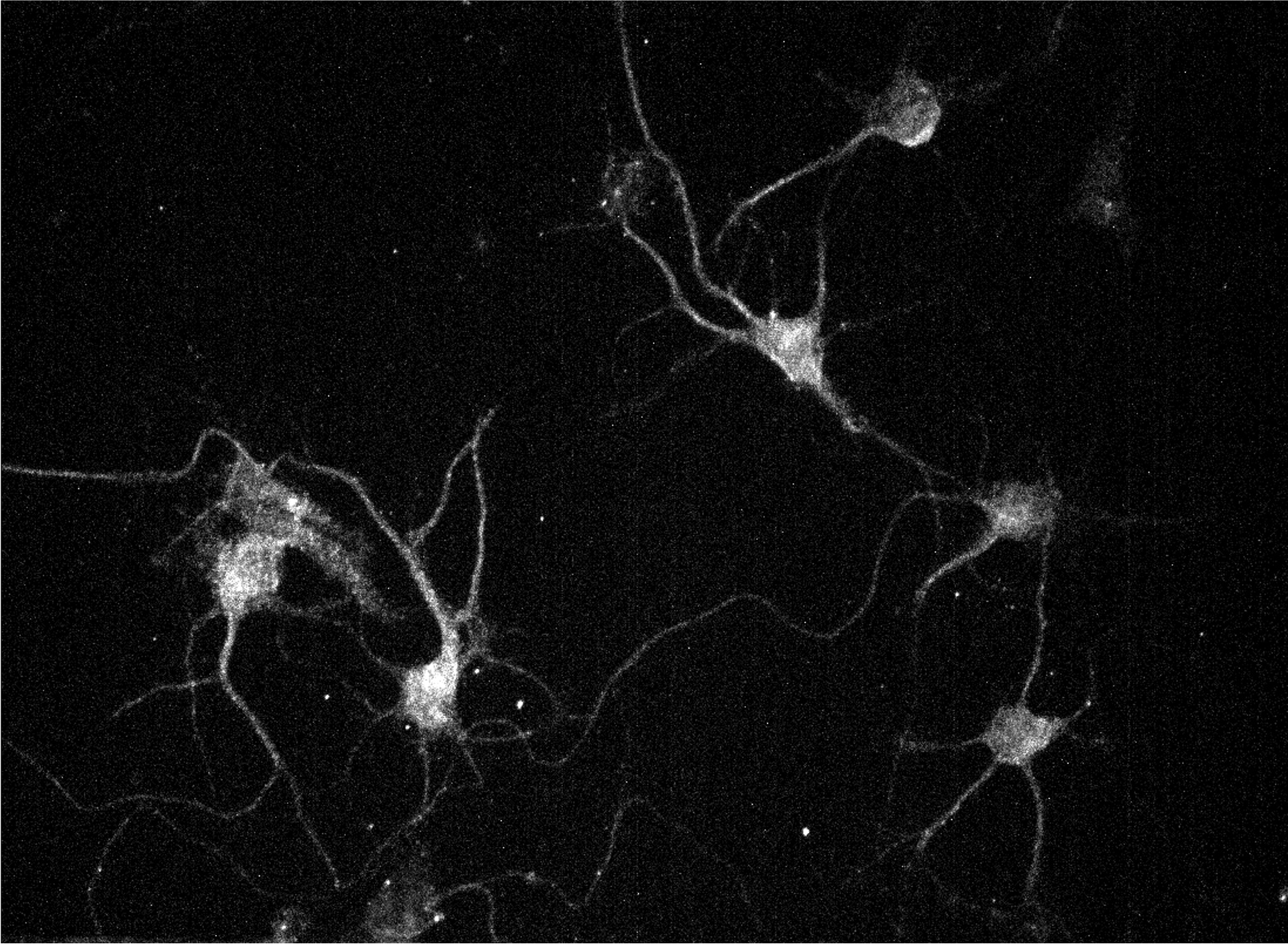

Supplement: Supplementary file 3 — Source data Fig. 1 [file 44318_2025_560_MOESM3_ESM.zip › Figure1/1E/Figure 1E_control_p-SR_p-PXNS119_p-PXNS119.tif]

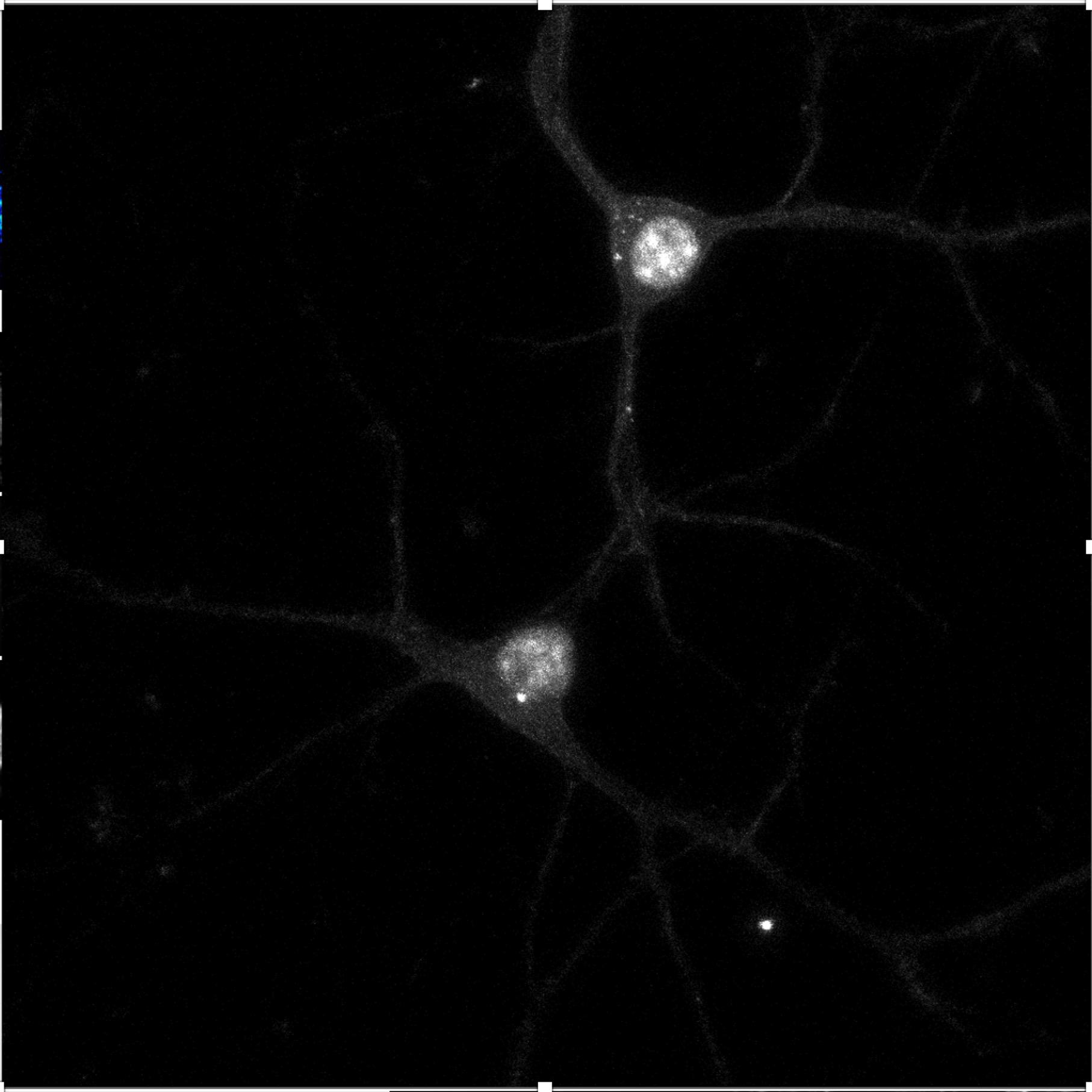

Supplement: Supplementary file 3 — Source data Fig. 1 [file 44318_2025_560_MOESM3_ESM.zip › Figure1/1E/Figure 2C_DMSO_DIV7 neurons_p-PXNS119.tif]

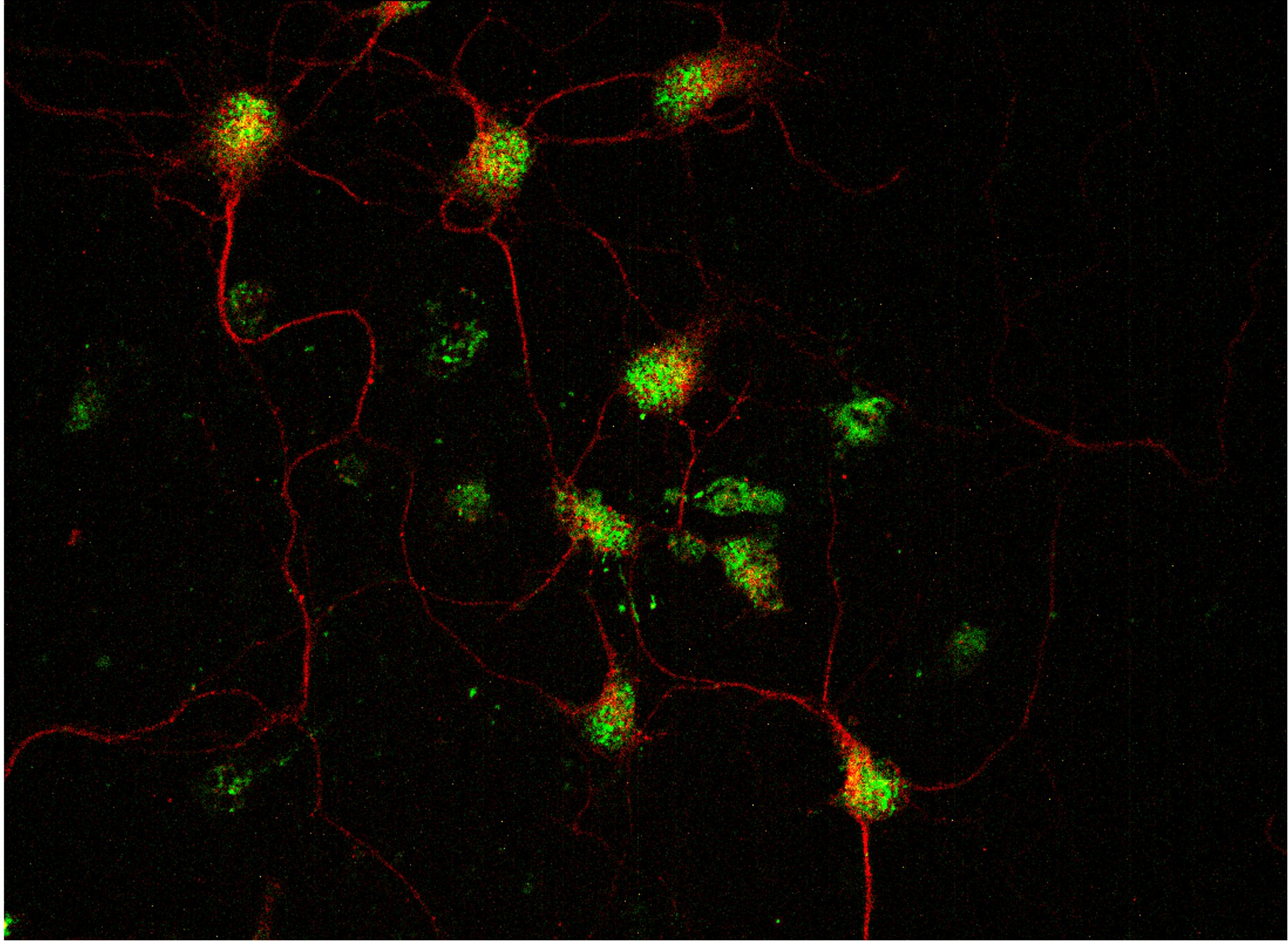

Supplement: Supplementary file 3 — Source data Fig. 1 [file 44318_2025_560_MOESM3_ESM.zip › Figure1/1E/Figure 1E_KPT-330_p-SR_p-PXNS119_merge.tif]

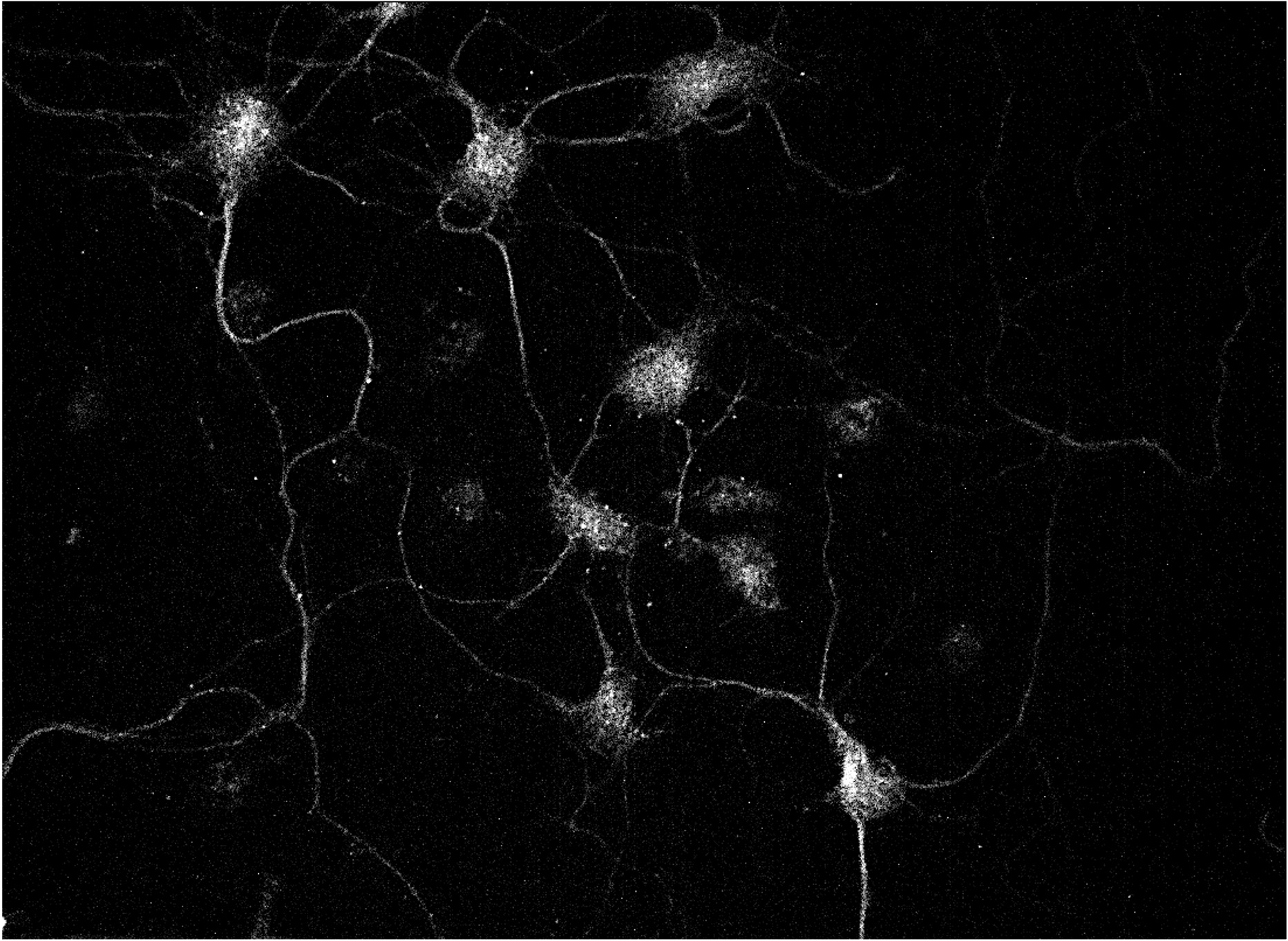

Supplement: Supplementary file 3 — Source data Fig. 1 [file 44318_2025_560_MOESM3_ESM.zip › Figure1/1E/Figure 1E_KPT-330_p-SR_p-PXNS119_p-PXNS119.tif]

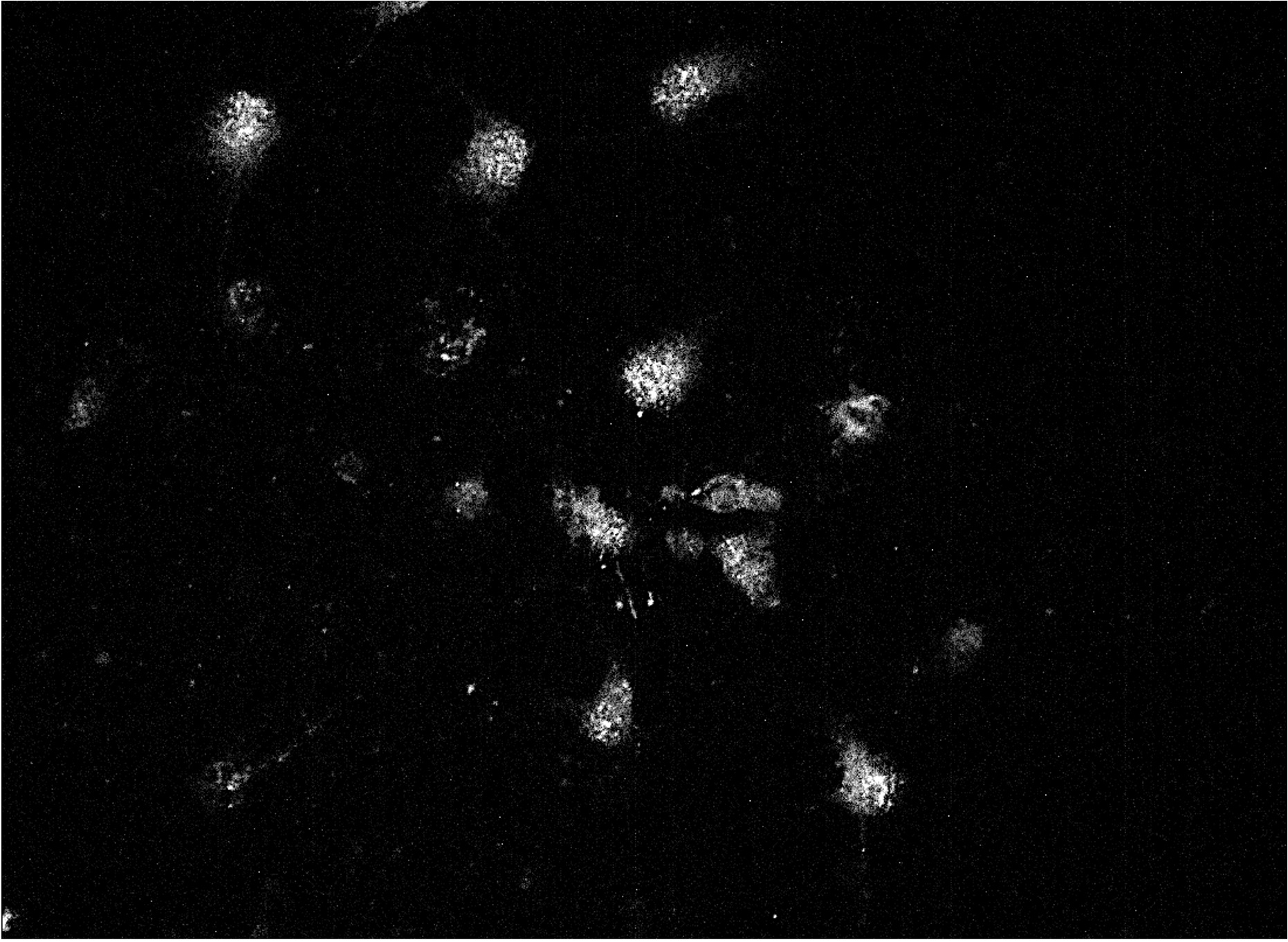

Supplement: Supplementary file 3 — Source data Fig. 1 [file 44318_2025_560_MOESM3_ESM.zip › Figure1/1E/Figure 1E_KPT-330_p-SR_p-PXNS119_p-SR.tif]

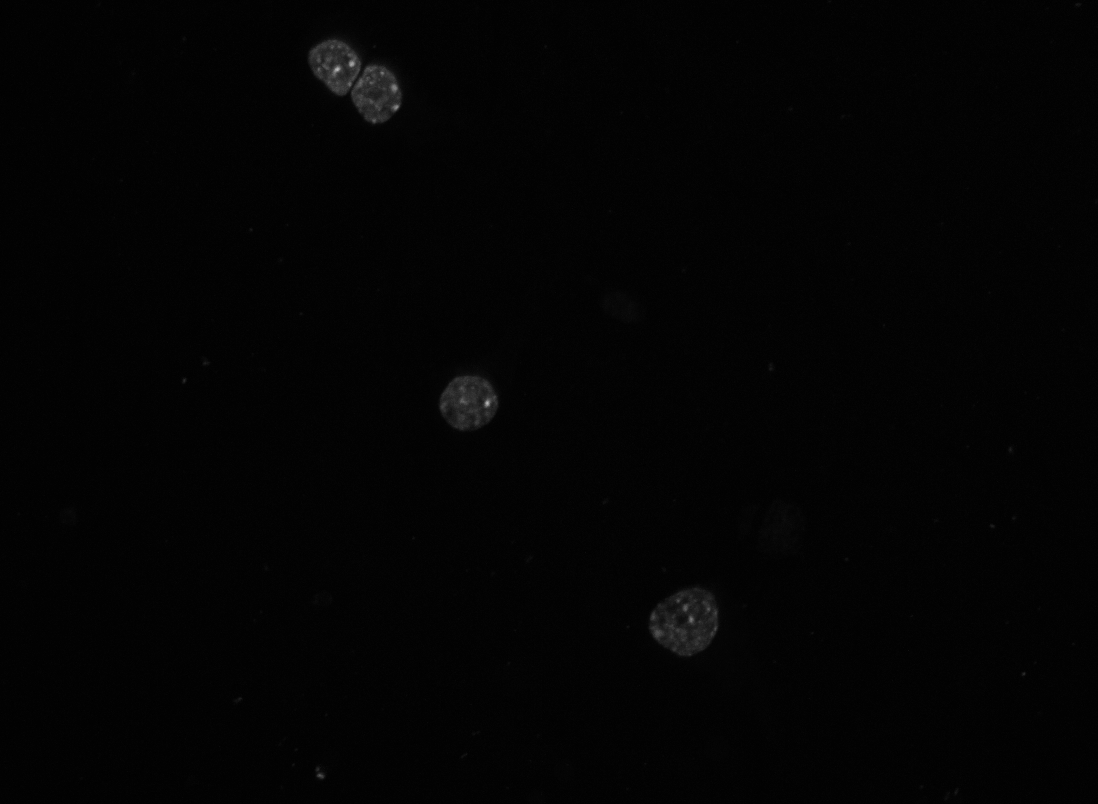

Supplement: Supplementary file 4 — Source data Fig. 2 [file 44318_2025_560_MOESM4_ESM.zip › Figure2/2A/Primary neuron_BDNF_DIV14_DAPI.tif]

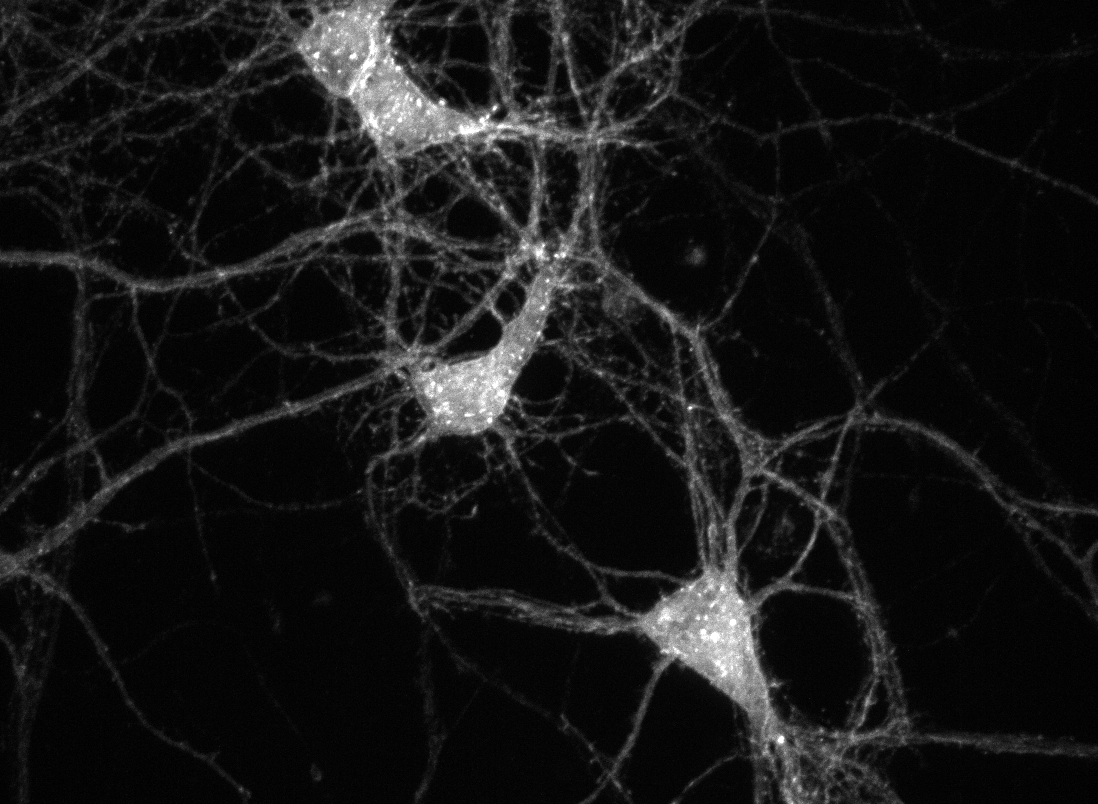

Supplement: Supplementary file 4 — Source data Fig. 2 [file 44318_2025_560_MOESM4_ESM.zip › Figure2/2A/Primary neuron_BDNF_DIV14_p-PaxillinS119.tif]

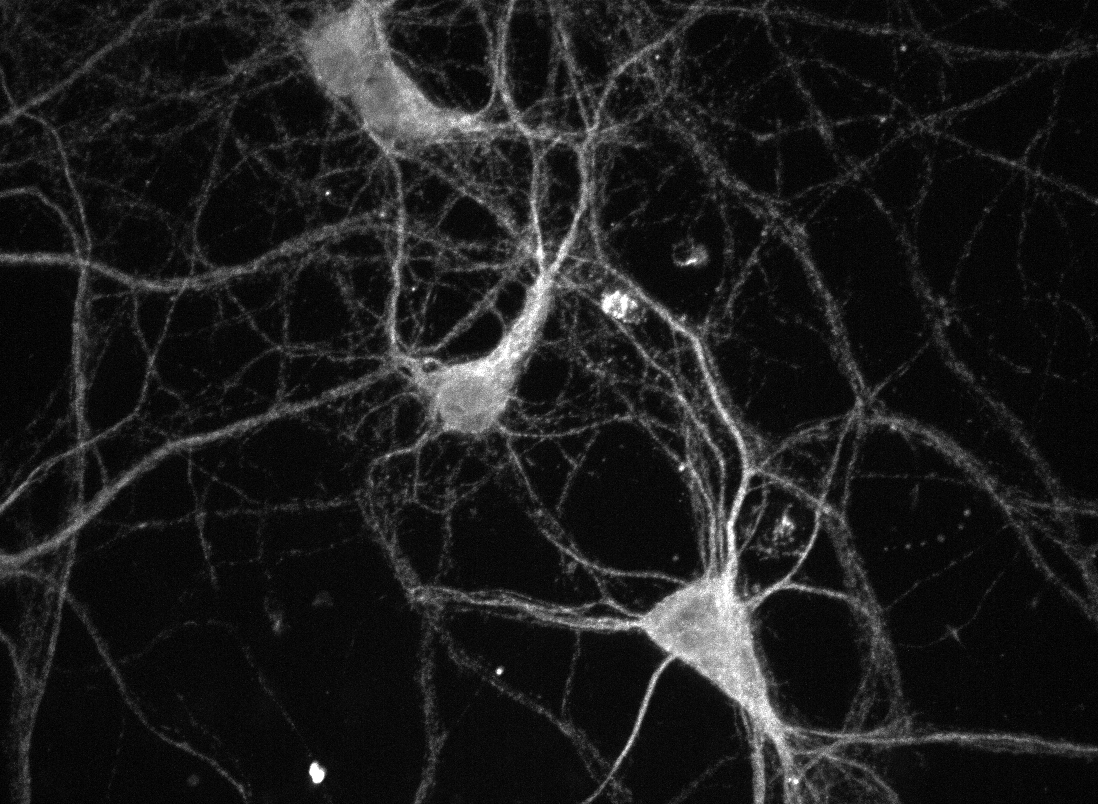

Supplement: Supplementary file 4 — Source data Fig. 2 [file 44318_2025_560_MOESM4_ESM.zip › Figure2/2A/Primary neuron_BDNF_DIV14_Tuj-1.tif]

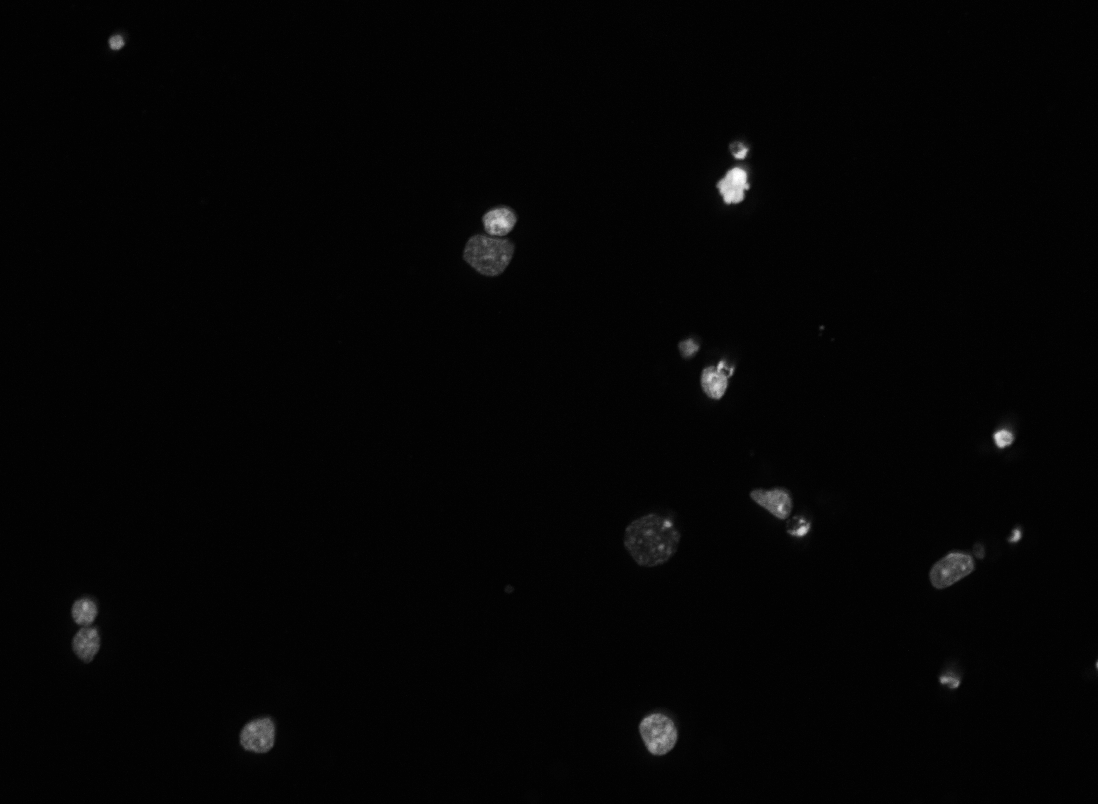

Supplement: Supplementary file 4 — Source data Fig. 2 [file 44318_2025_560_MOESM4_ESM.zip › Figure2/2A/Primary neuron_Con_DIV14_DAPI.tif]

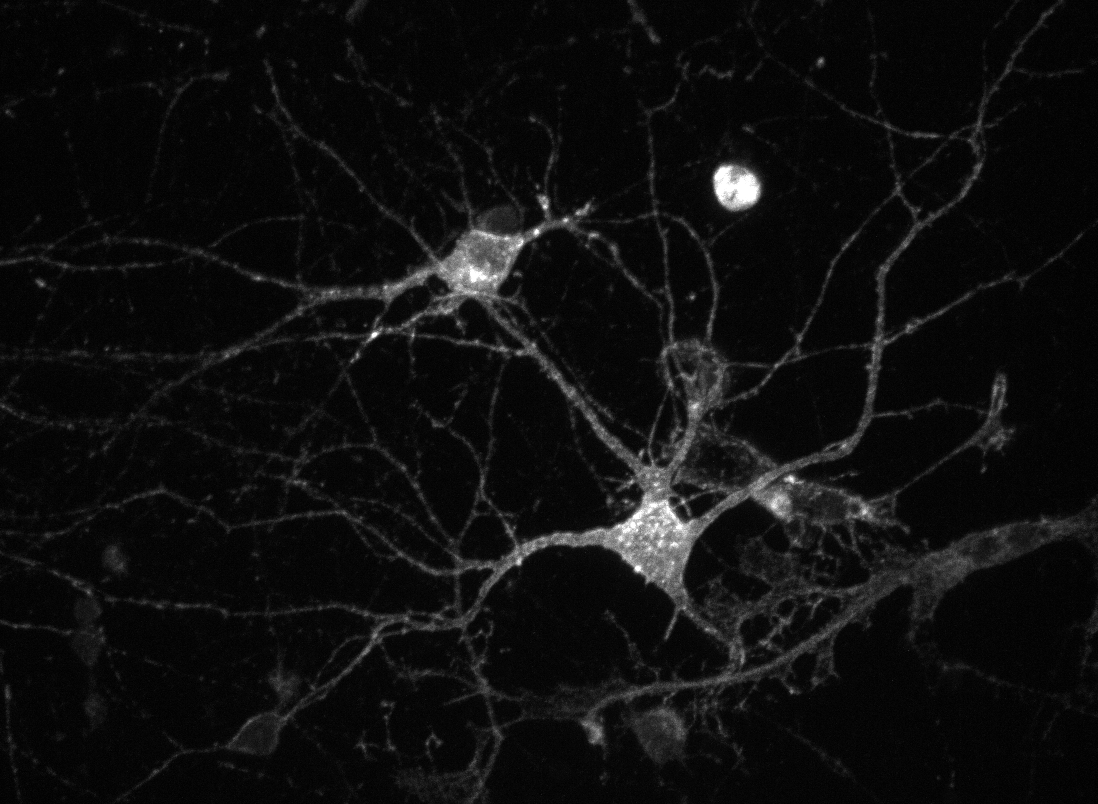

Supplement: Supplementary file 4 — Source data Fig. 2 [file 44318_2025_560_MOESM4_ESM.zip › Figure2/2A/Primary neuron_Con_DIV14_p-PaxillinS119.tif]

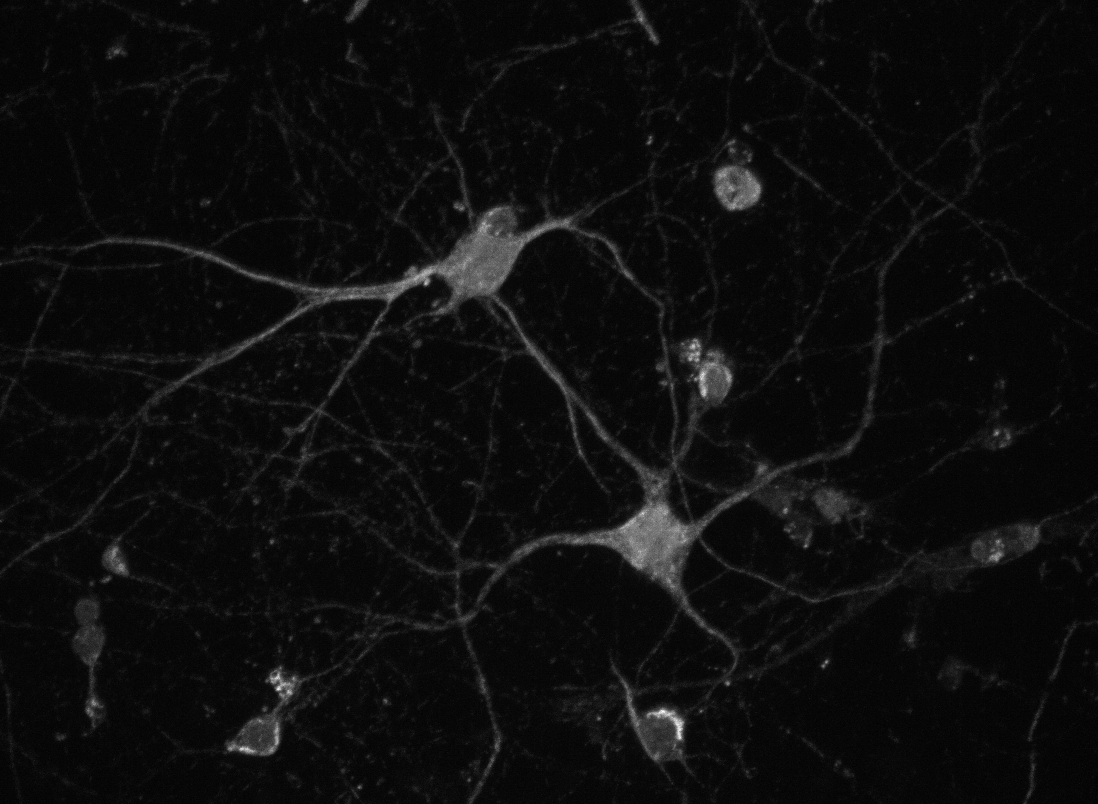

Supplement: Supplementary file 4 — Source data Fig. 2 [file 44318_2025_560_MOESM4_ESM.zip › Figure2/2A/Primary neuron_Con_DIV14_Tuj-1.tif]

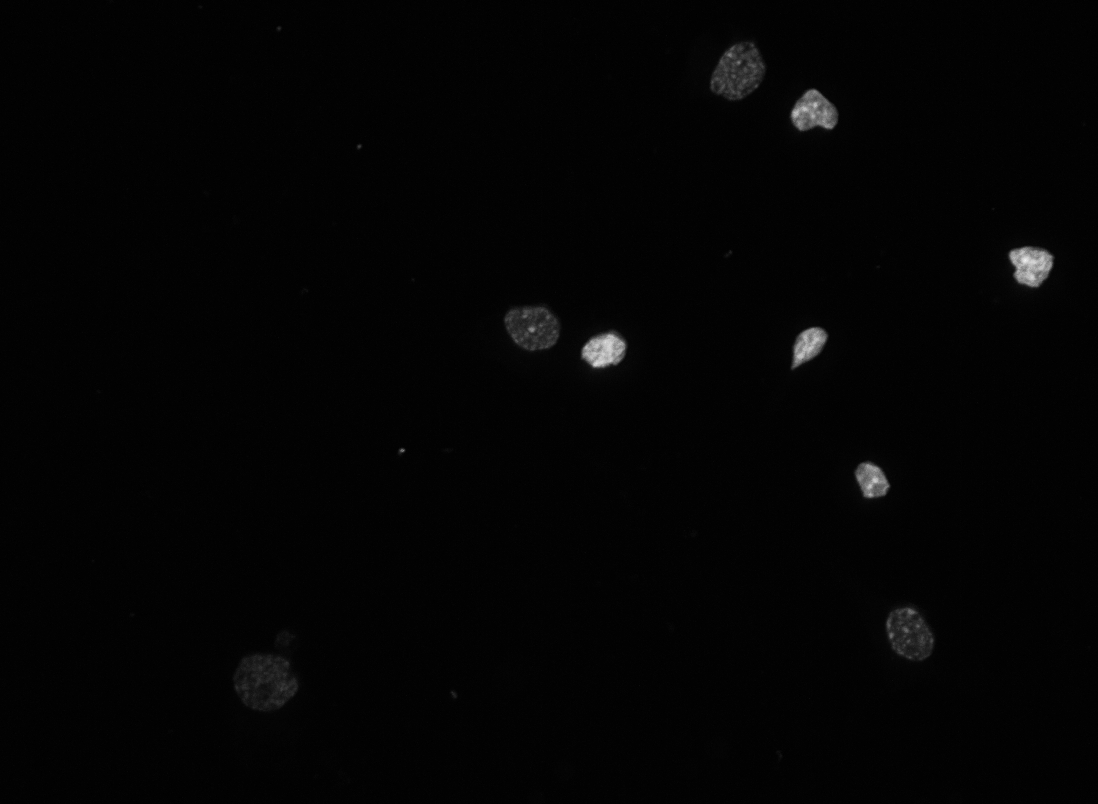

Supplement: Supplementary file 4 — Source data Fig. 2 [file 44318_2025_560_MOESM4_ESM.zip › Figure2/2A/Primary neuron_Fsk_DIV14_DAPI.tif]

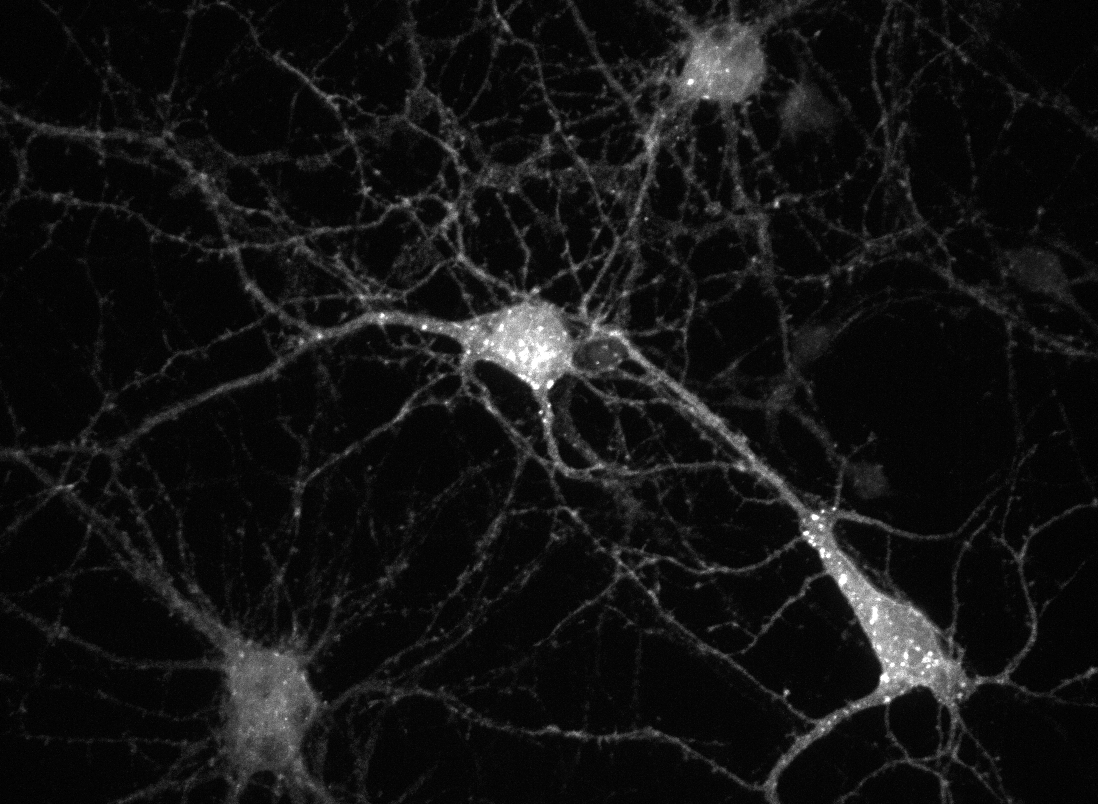

Supplement: Supplementary file 4 — Source data Fig. 2 [file 44318_2025_560_MOESM4_ESM.zip › Figure2/2A/Primary neuron_Fsk_DIV14_p-PaxillinS119.tif]

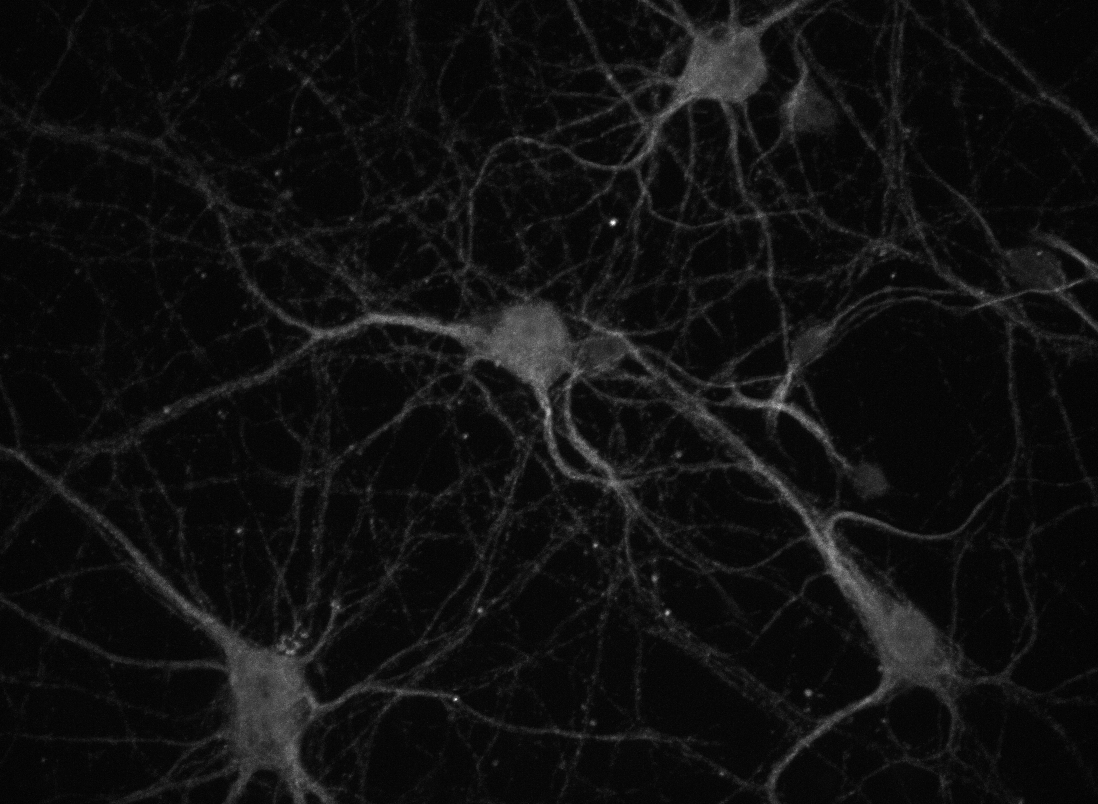

Supplement: Supplementary file 4 — Source data Fig. 2 [file 44318_2025_560_MOESM4_ESM.zip › Figure2/2A/Primary neuron_Fsk_DIV14_Tuj-1.tif]

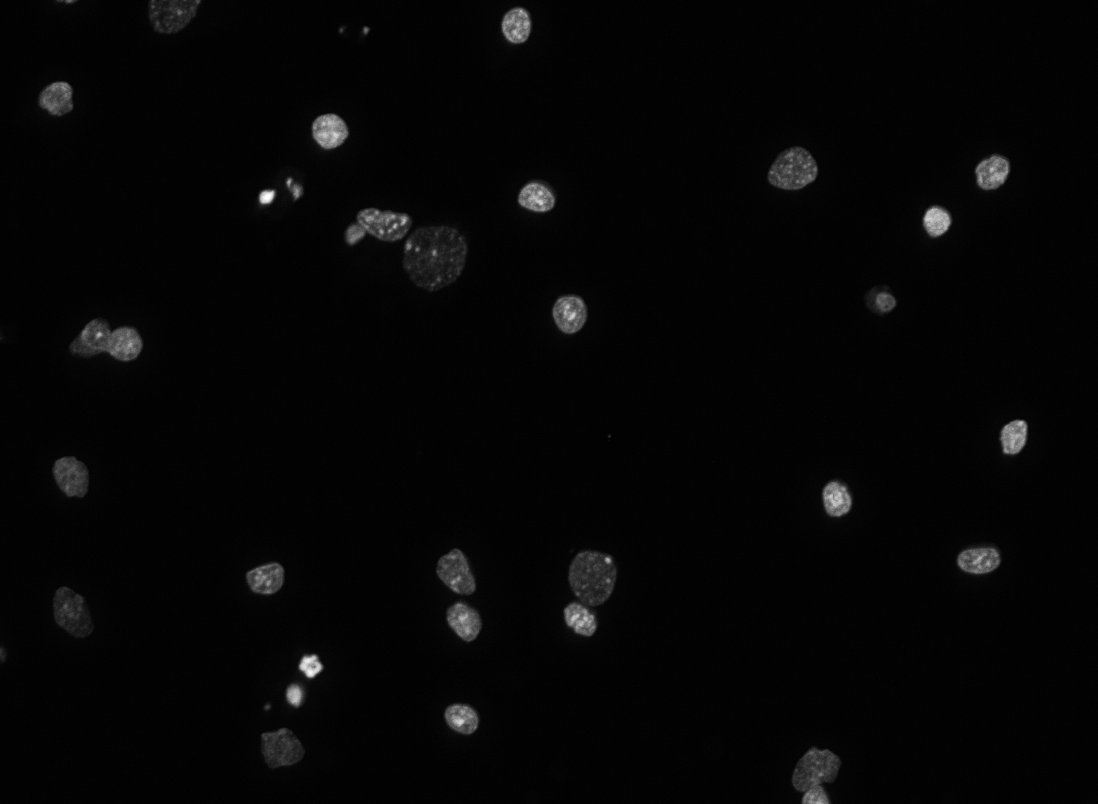

Supplement: Supplementary file 4 — Source data Fig. 2 [file 44318_2025_560_MOESM4_ESM.zip › Figure2/2A/Primary neuron_NMDA_DIV14_DAPI.tif]

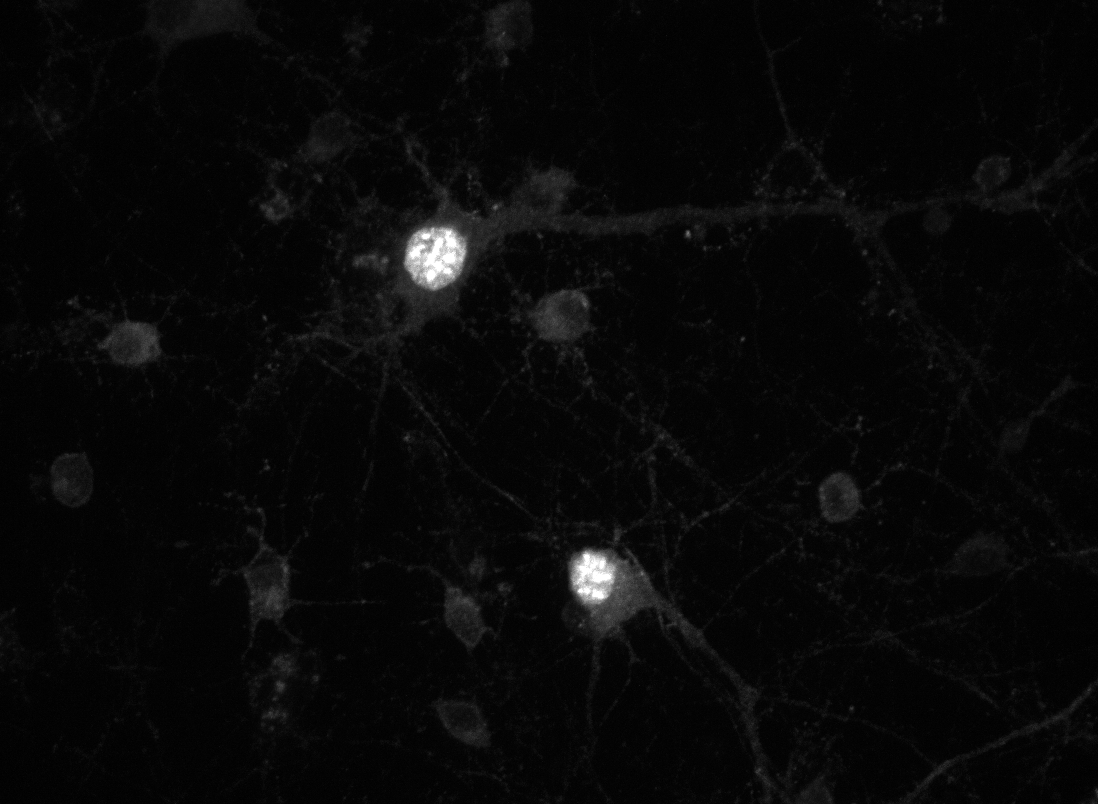

Supplement: Supplementary file 4 — Source data Fig. 2 [file 44318_2025_560_MOESM4_ESM.zip › Figure2/2A/Primary neuron_NMDA_DIV14_p-PaxillinS119.tif]

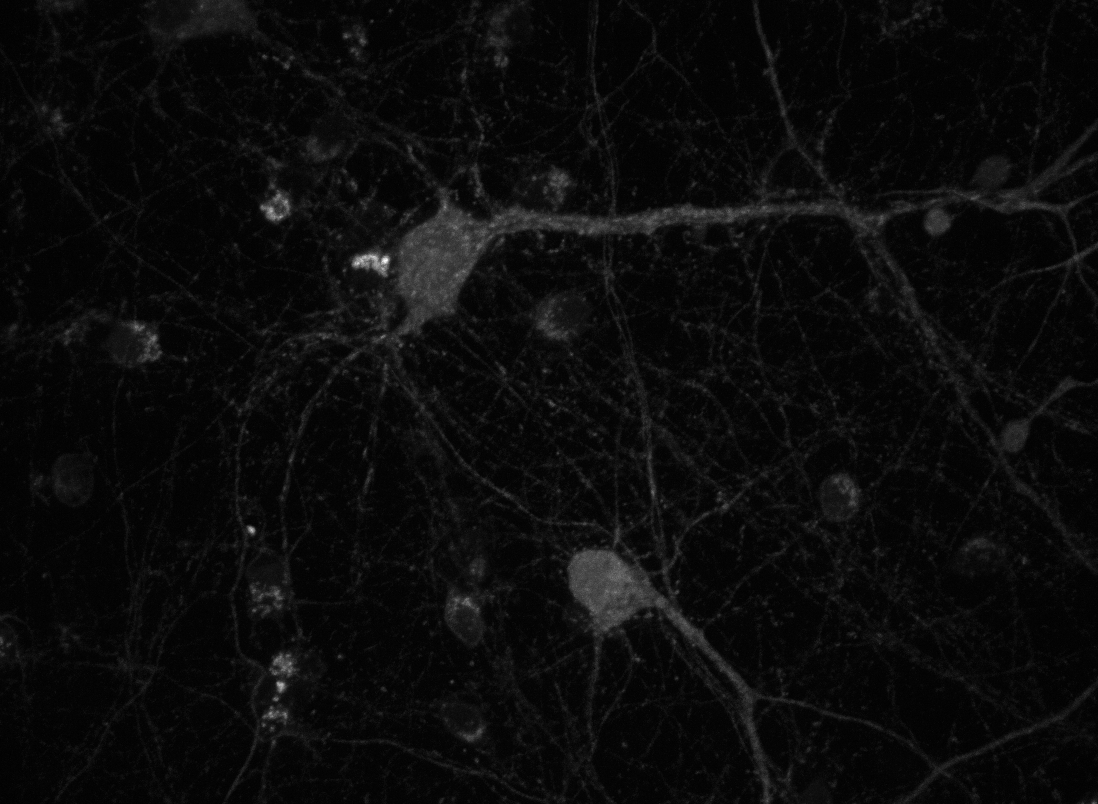

Supplement: Supplementary file 4 — Source data Fig. 2 [file 44318_2025_560_MOESM4_ESM.zip › Figure2/2A/Primary neuron_NMDA_DIV14_Tuj-1.tif]

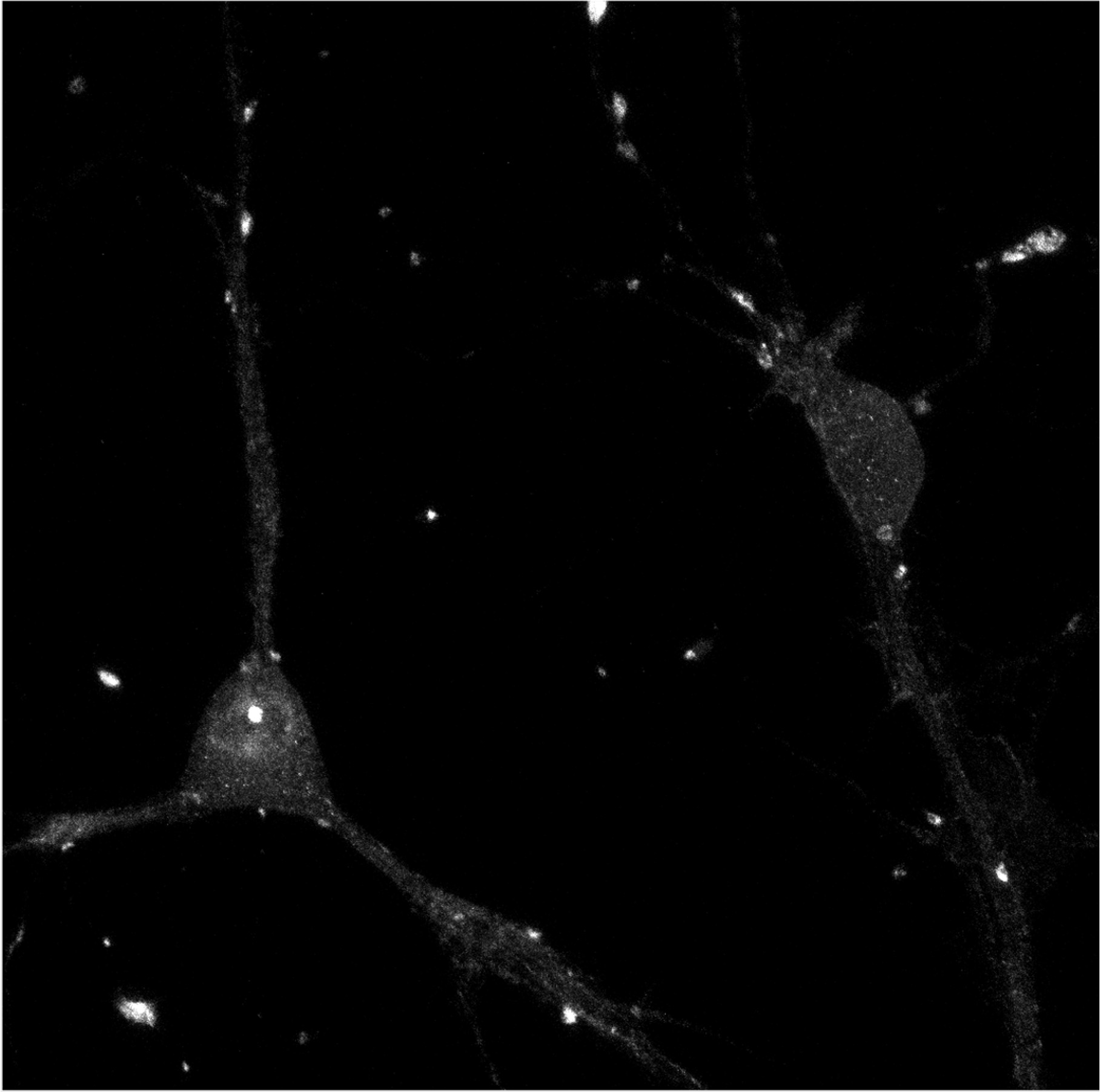

Supplement: Supplementary file 4 — Source data Fig. 2 [file 44318_2025_560_MOESM4_ESM.zip › Figure2/2C/Figure 2C_K252a_DIV7 neurons_p-PXNS119.tif]

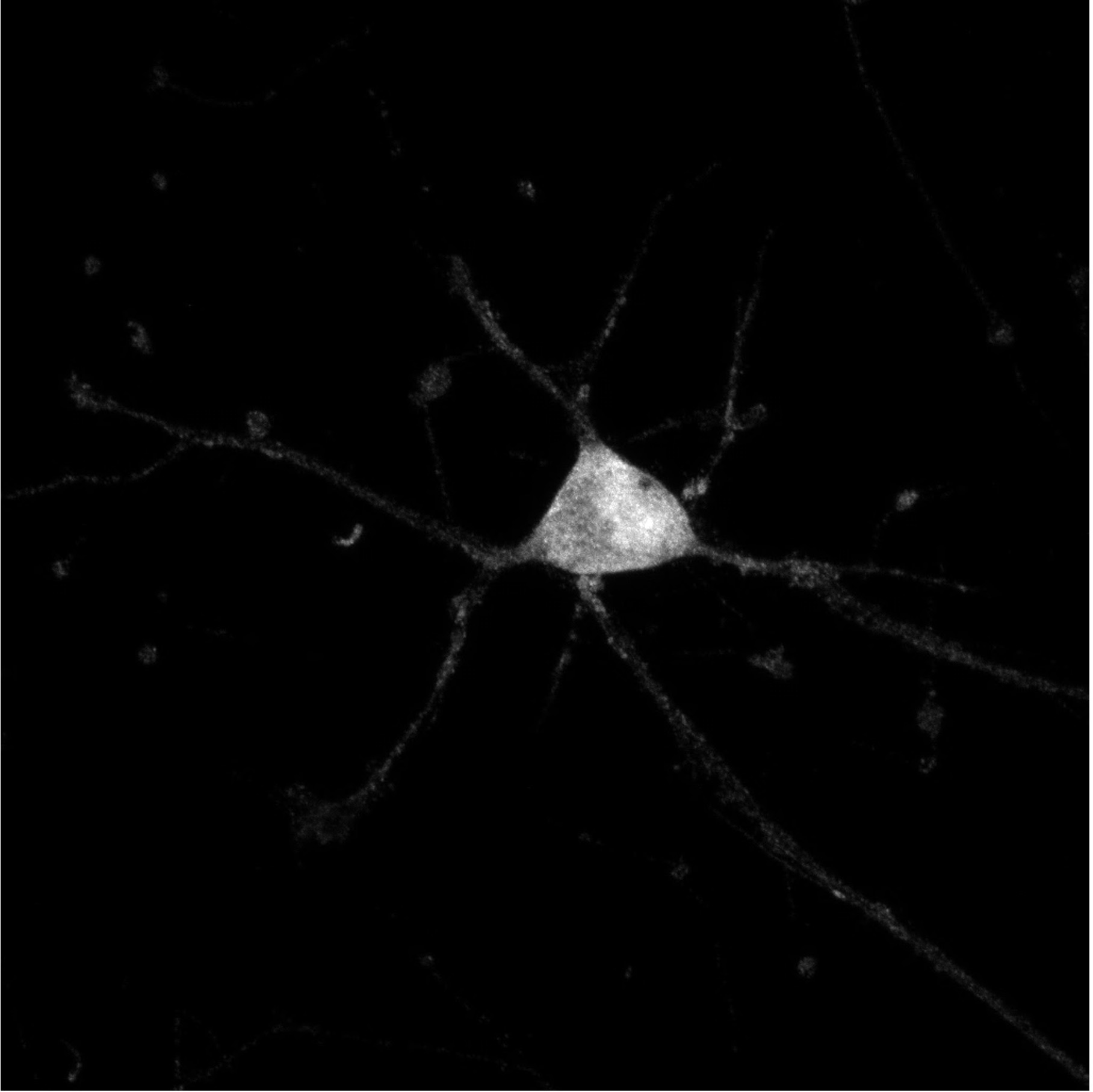

Supplement: Supplementary file 4 — Source data Fig. 2 [file 44318_2025_560_MOESM4_ESM.zip › Figure2/2C/Figure 2C_Purval_DIV7 neurons_Tuj-1.tif]

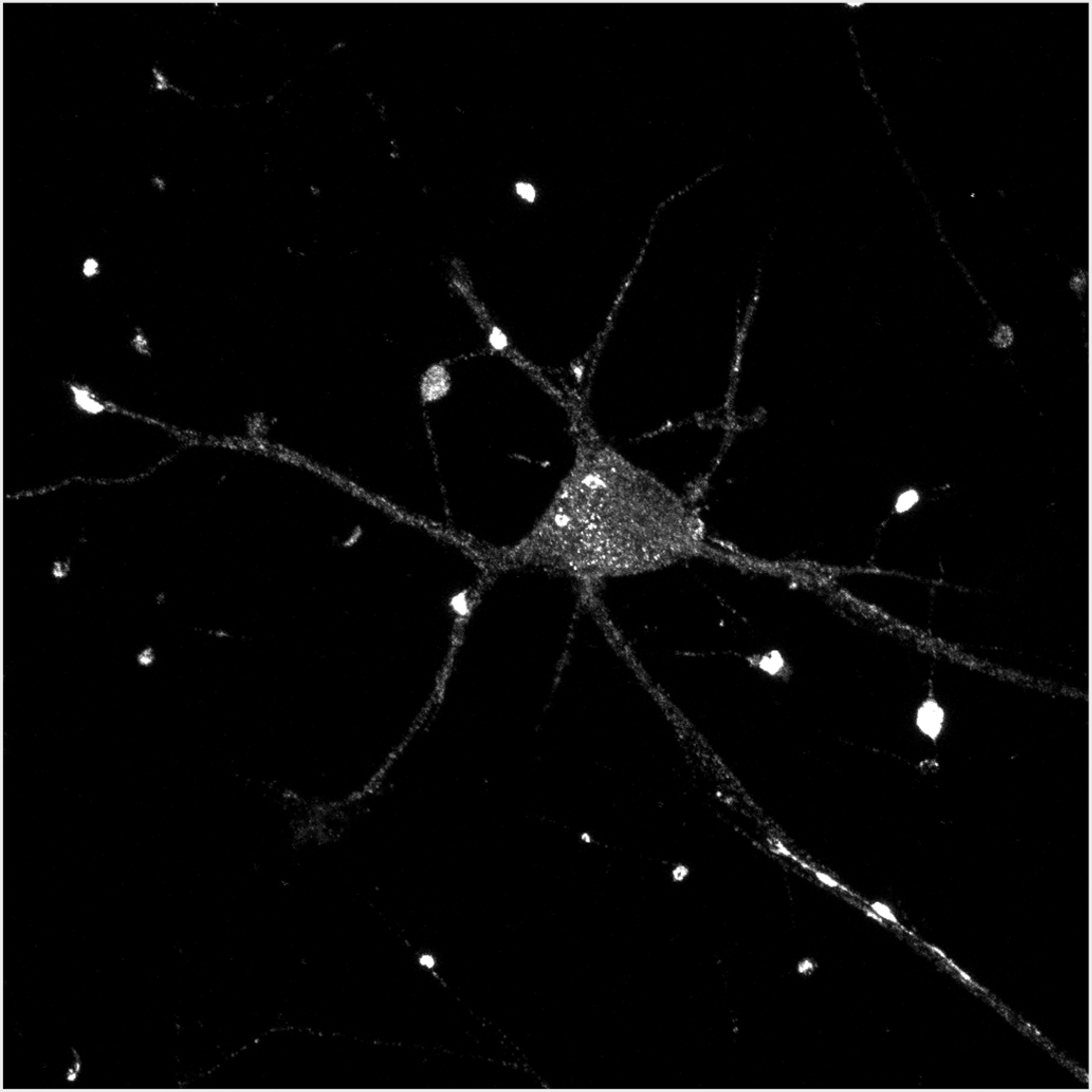

Supplement: Supplementary file 4 — Source data Fig. 2 [file 44318_2025_560_MOESM4_ESM.zip › Figure2/2C/Figure 2C_Purval_DIV7 neurons_p-PXNS119.tif]

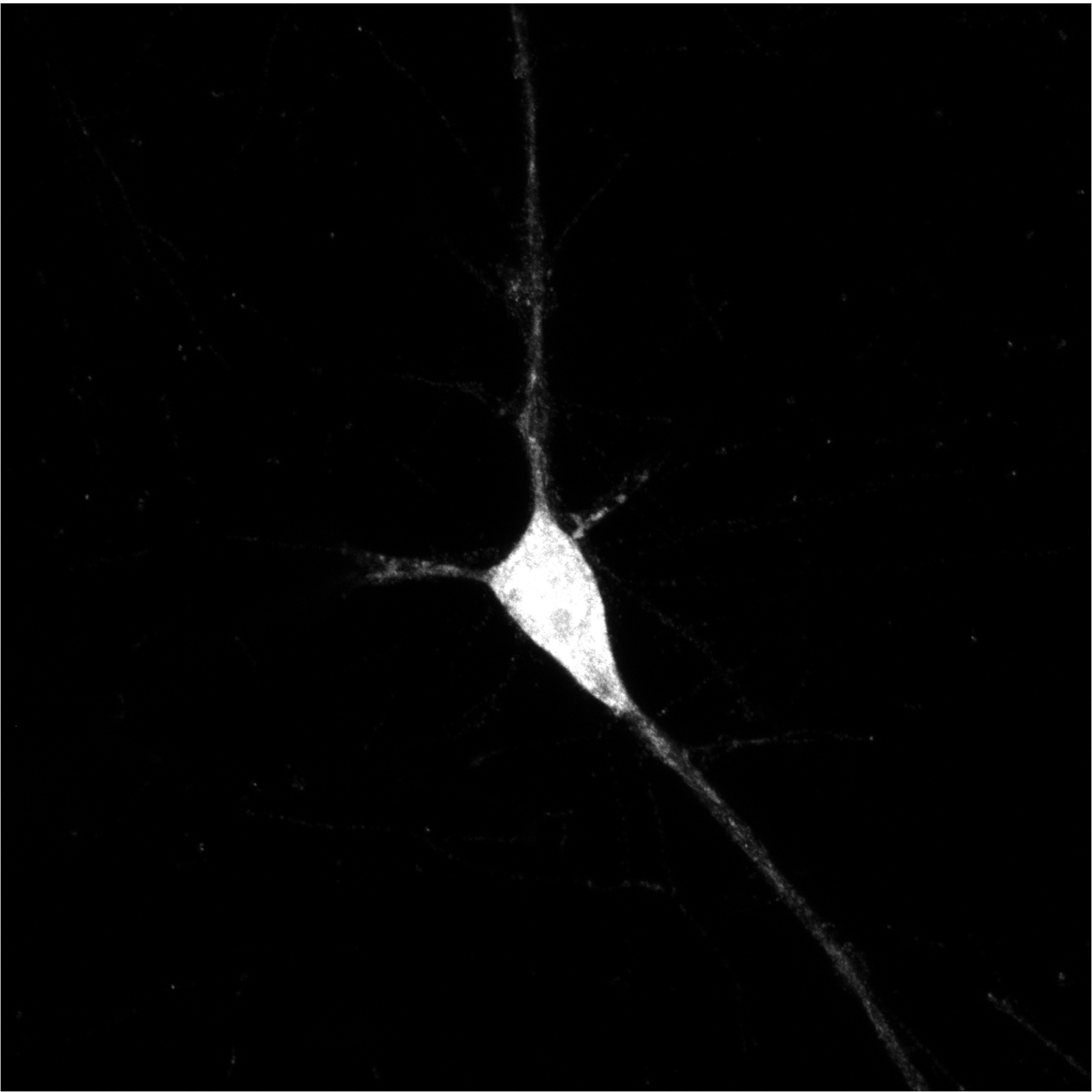

Supplement: Supplementary file 4 — Source data Fig. 2 [file 44318_2025_560_MOESM4_ESM.zip › Figure2/2C/Figure 2C_Rosocovitine_DIV7 neurons_Tuj-1.tif]

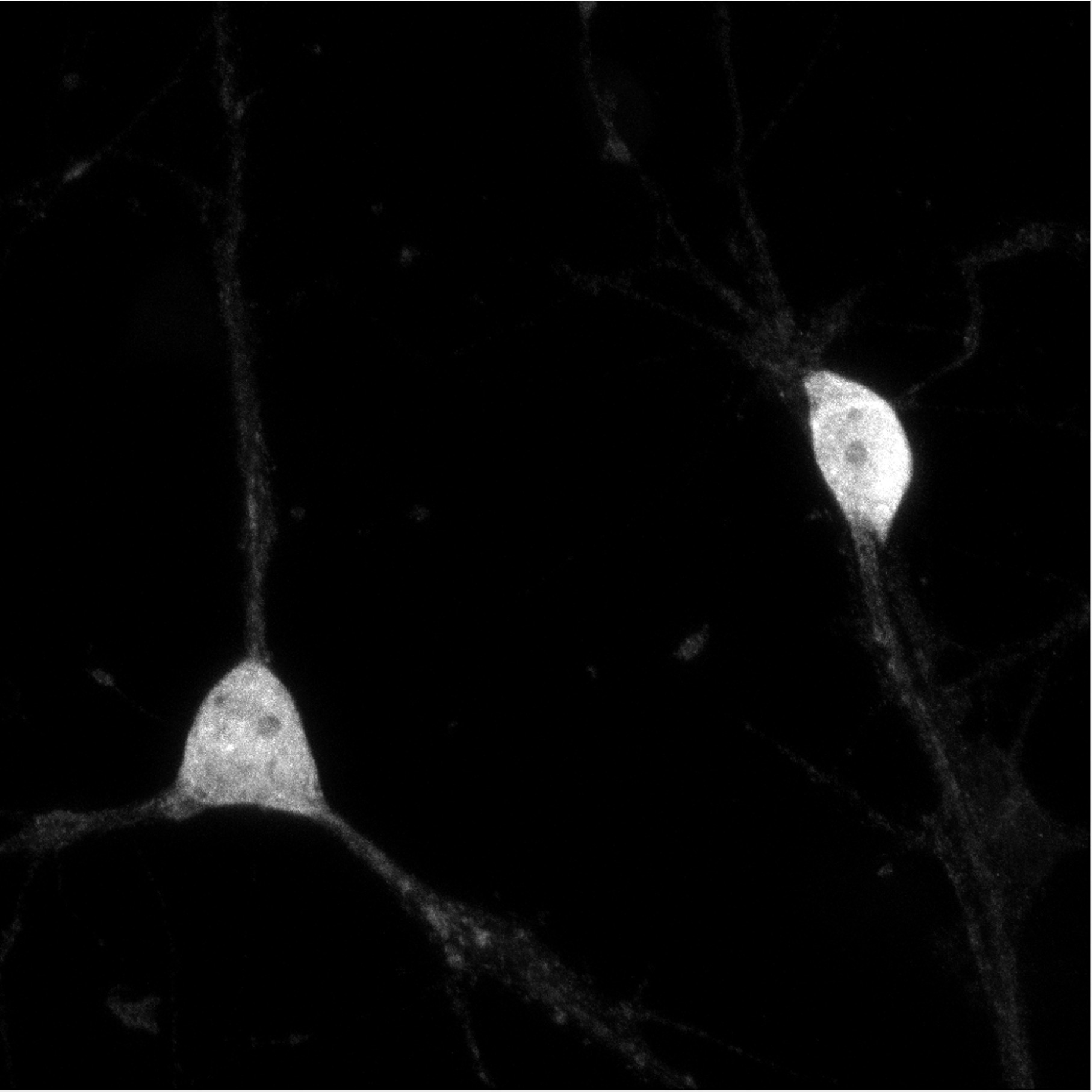

Supplement: Supplementary file 4 — Source data Fig. 2 [file 44318_2025_560_MOESM4_ESM.zip › Figure2/2C/Figure 2C_K252a_DIV7 neurons_Tuj-1.tif]

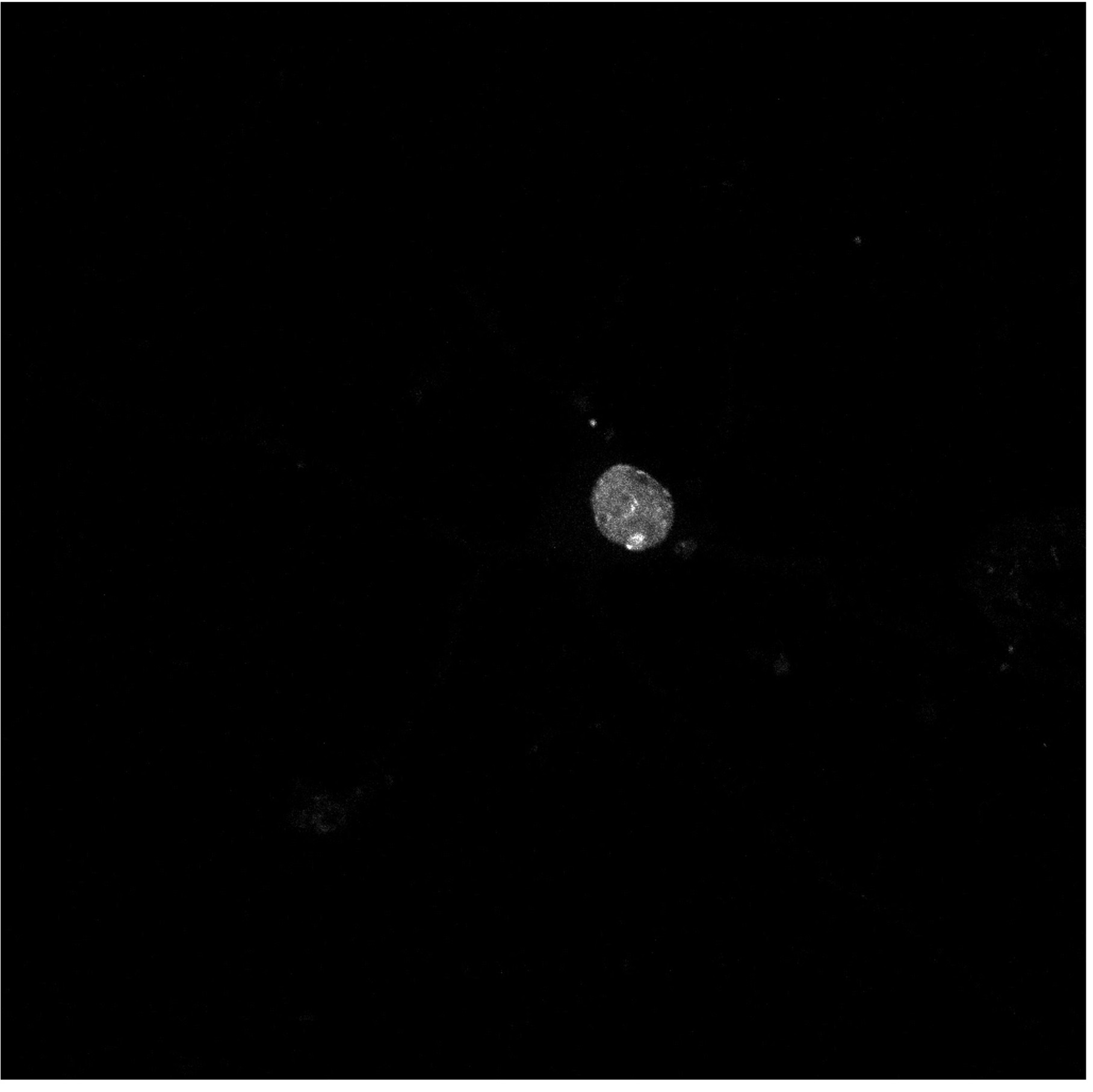

Supplement: Supplementary file 4 — Source data Fig. 2 [file 44318_2025_560_MOESM4_ESM.zip › Figure2/2C/Figure 2C_Purval_DIV7 neurons_DAPI.tif]

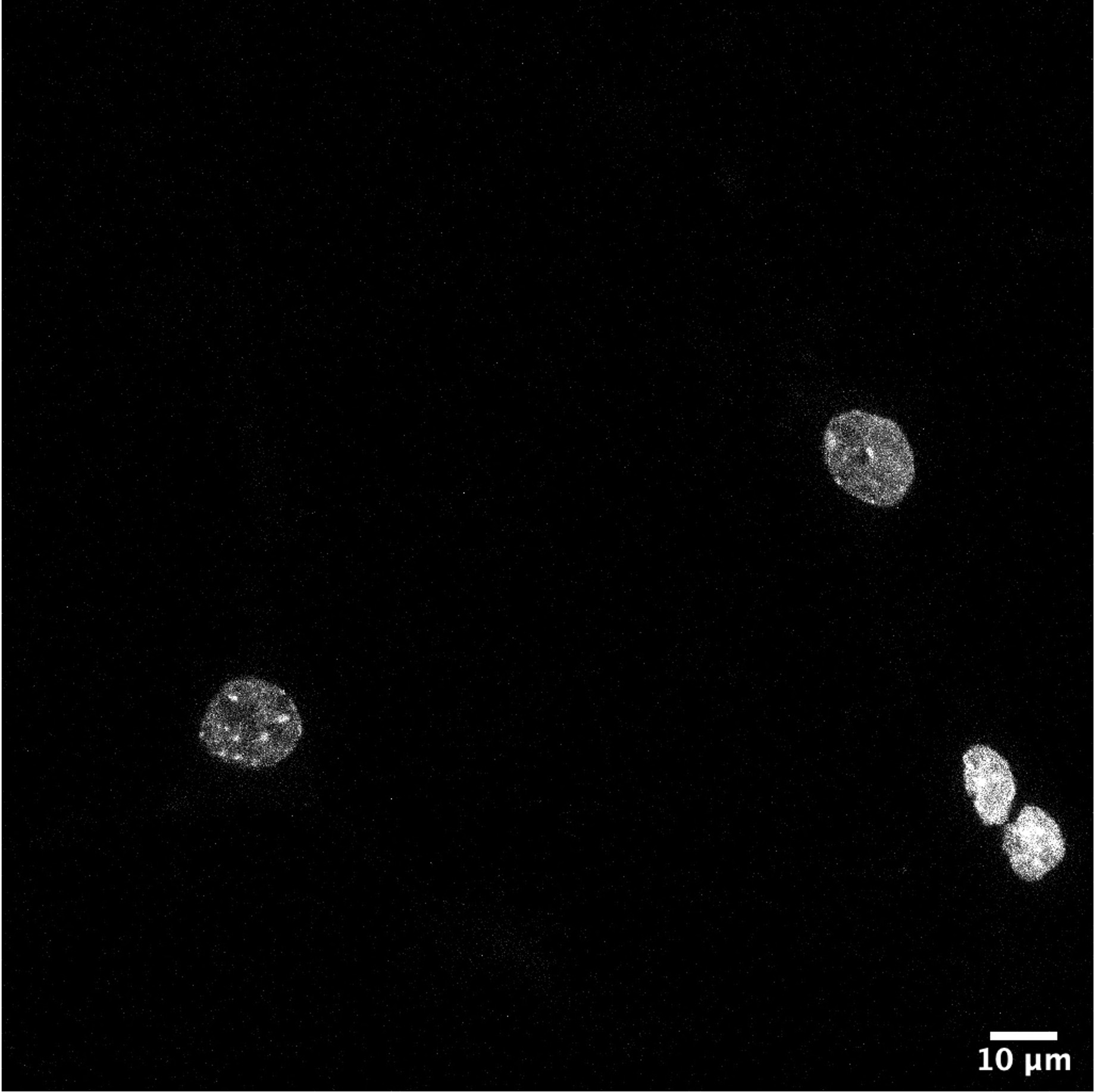

Supplement: Supplementary file 4 — Source data Fig. 2 [file 44318_2025_560_MOESM4_ESM.zip › Figure2/2C/Figure 2C_K252a_DIV7 neurons_DAPI.tif]

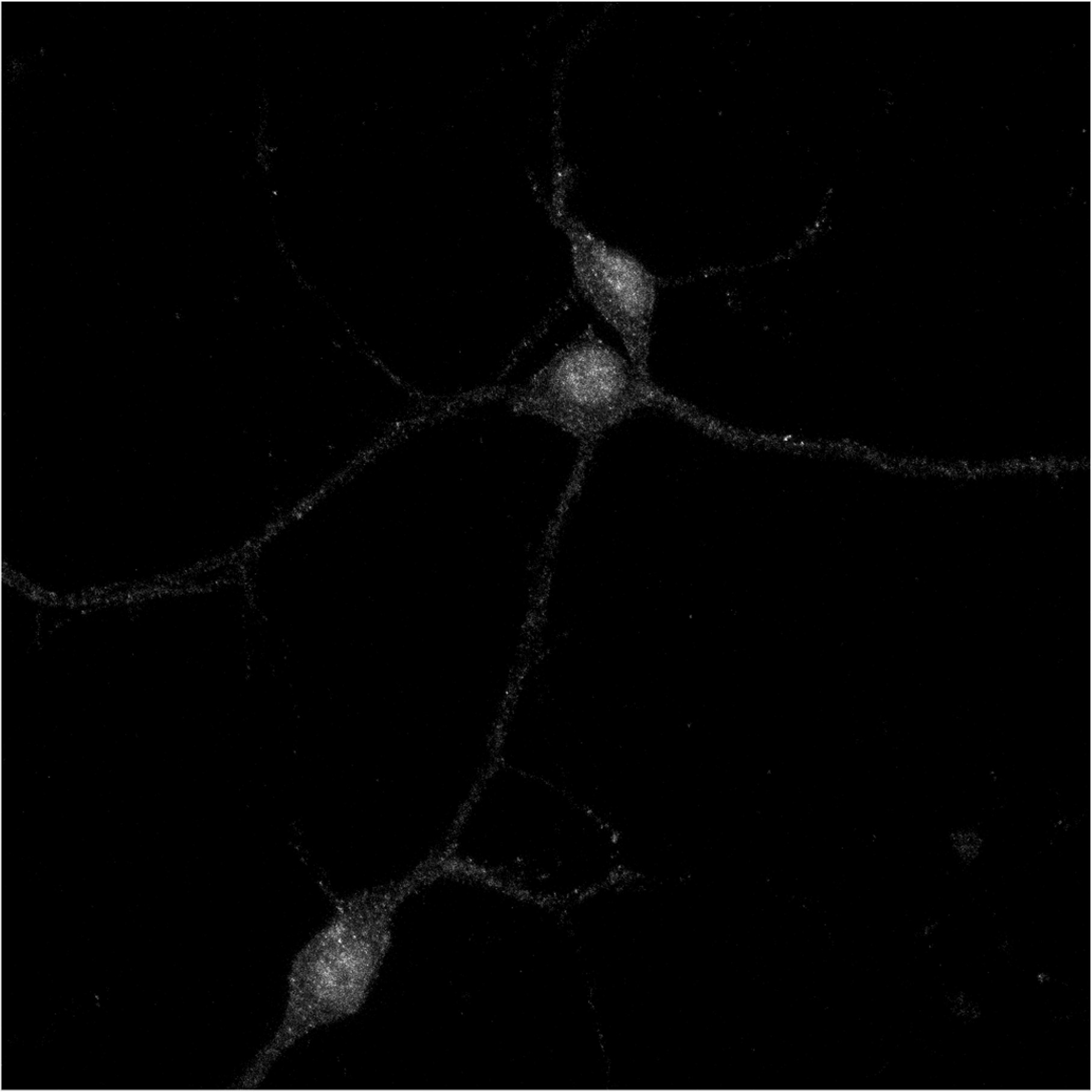

Supplement: Supplementary file 4 — Source data Fig. 2 [file 44318_2025_560_MOESM4_ESM.zip › Figure2/2C/Figure 2C_KT5720_DIV7 neurons_p-PXNS119.tif]

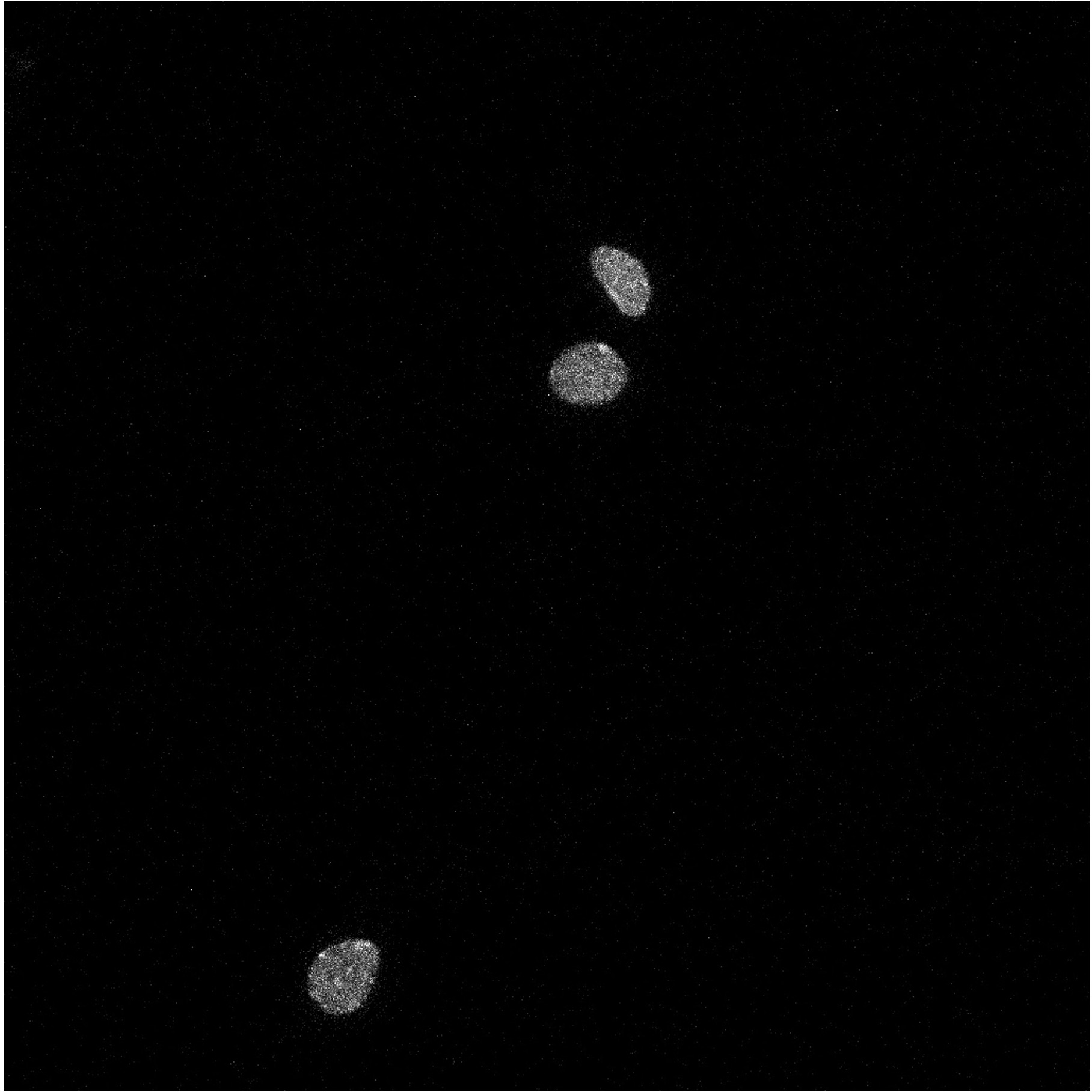

Supplement: Supplementary file 4 — Source data Fig. 2 [file 44318_2025_560_MOESM4_ESM.zip › Figure2/2C/Figure 2C_KT5720_DIV7 neurons_DAPI.tif]

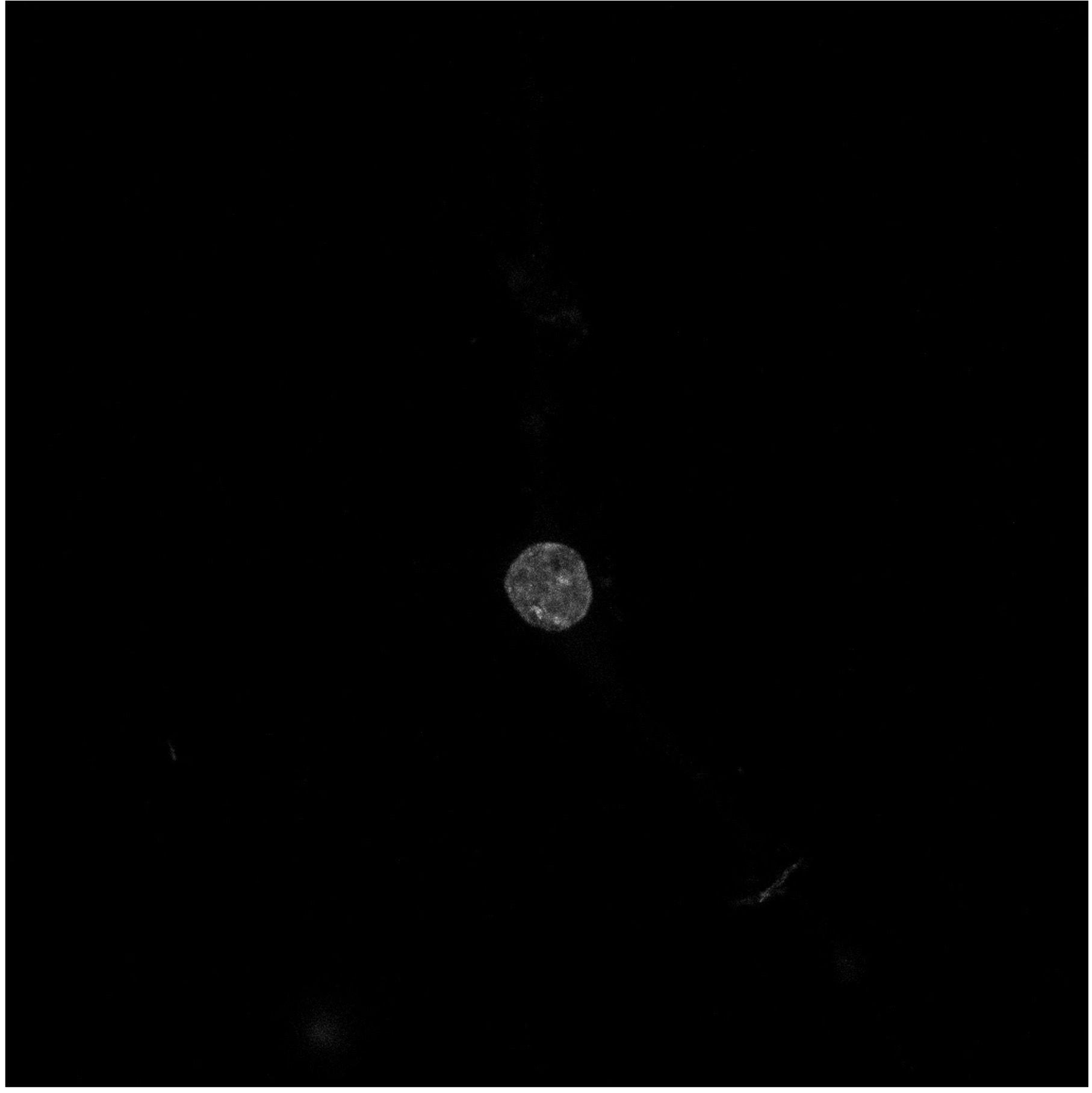

Supplement: Supplementary file 4 — Source data Fig. 2 [file 44318_2025_560_MOESM4_ESM.zip › Figure2/2C/Figure 2C_Rosocovitine_DIV7 neurons_DAPI.tif]

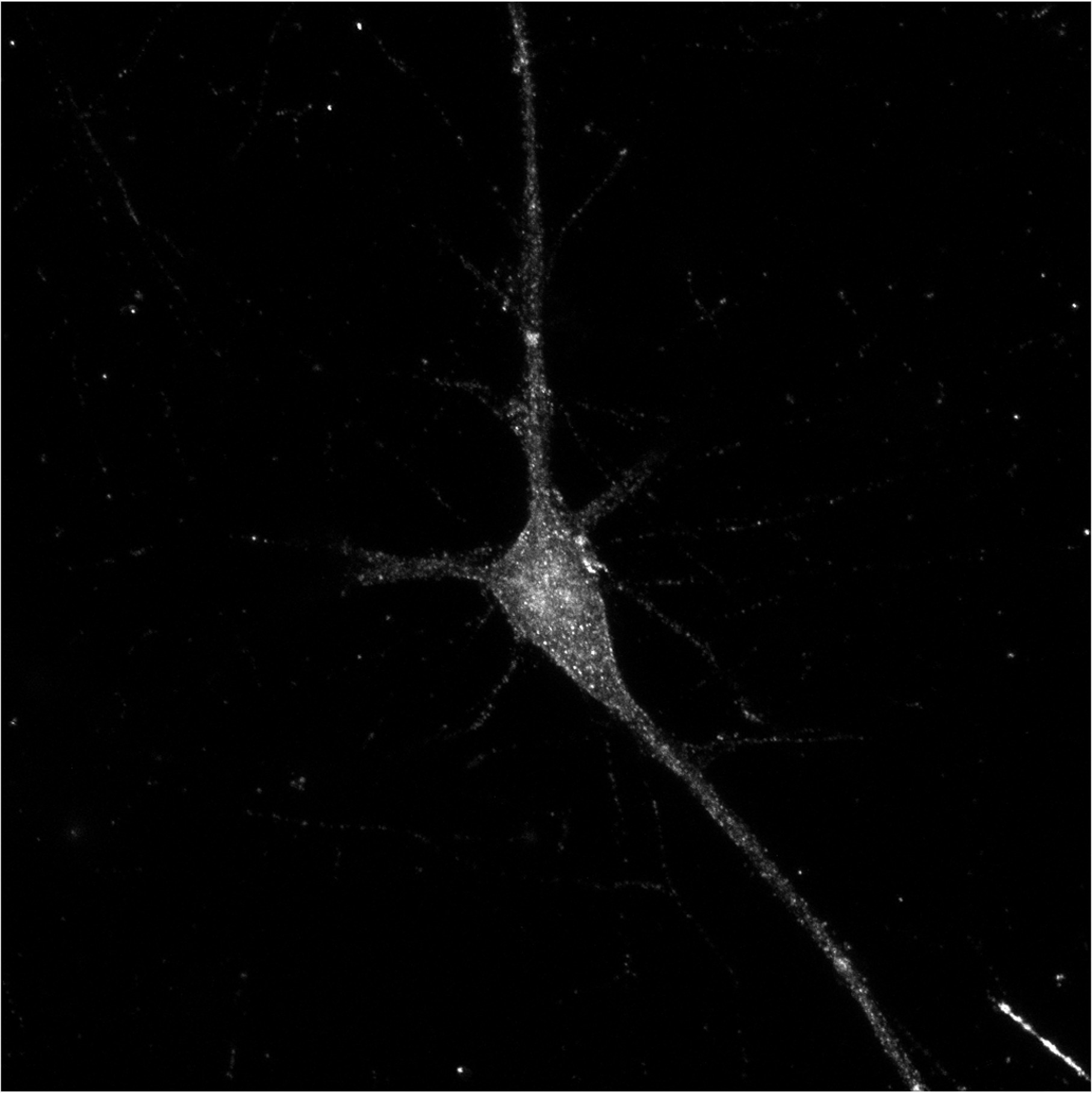

Supplement: Supplementary file 4 — Source data Fig. 2 [file 44318_2025_560_MOESM4_ESM.zip › Figure2/2C/Figure 2C_Rosocovitine_DIV7 neurons_p-PXNS119.tif]

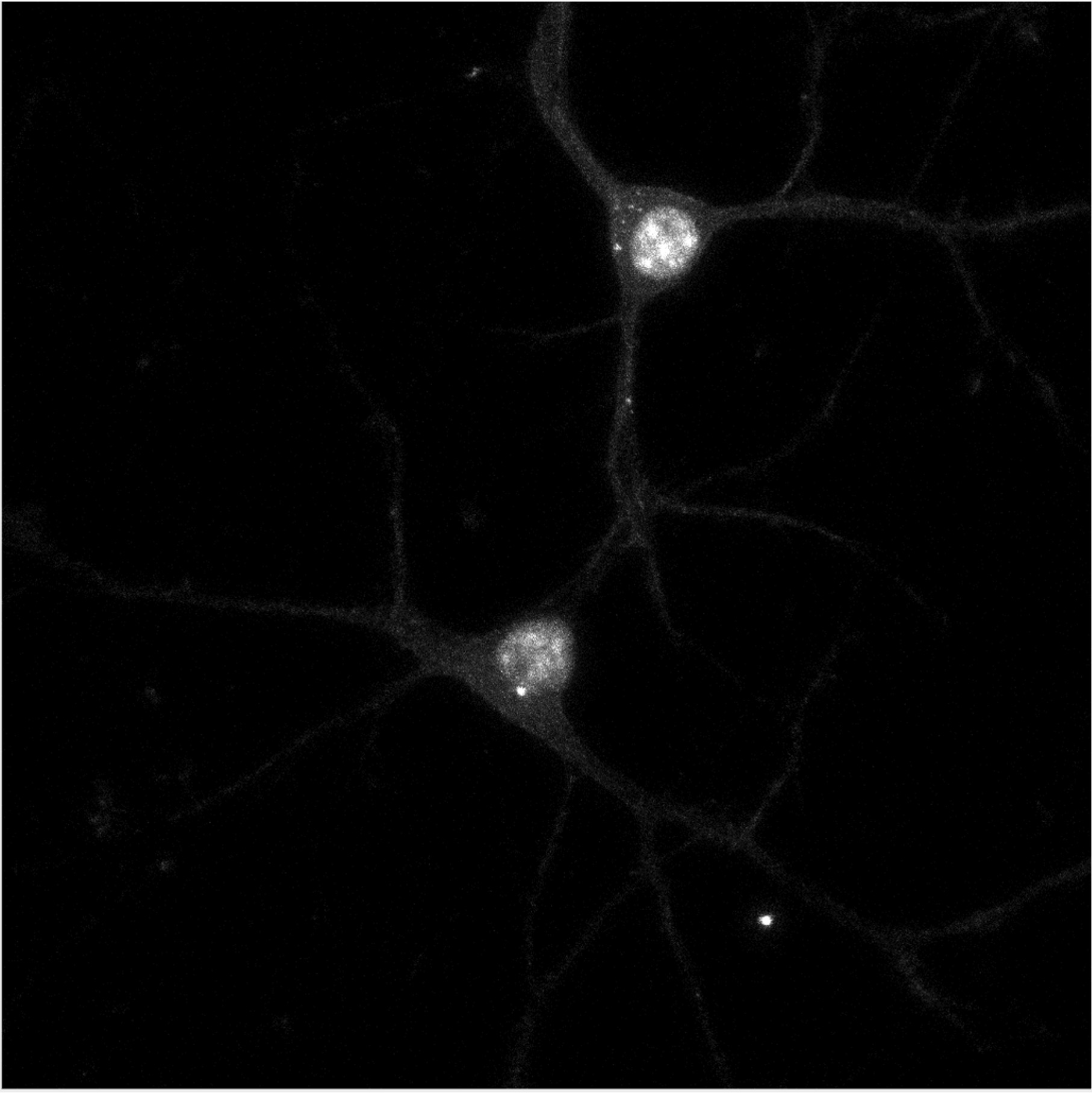

Supplement: Supplementary file 4 — Source data Fig. 2 [file 44318_2025_560_MOESM4_ESM.zip › Figure2/2C/Figure 2C_DMSO_DIV7 neurons_p-PXNS119-1.tif]

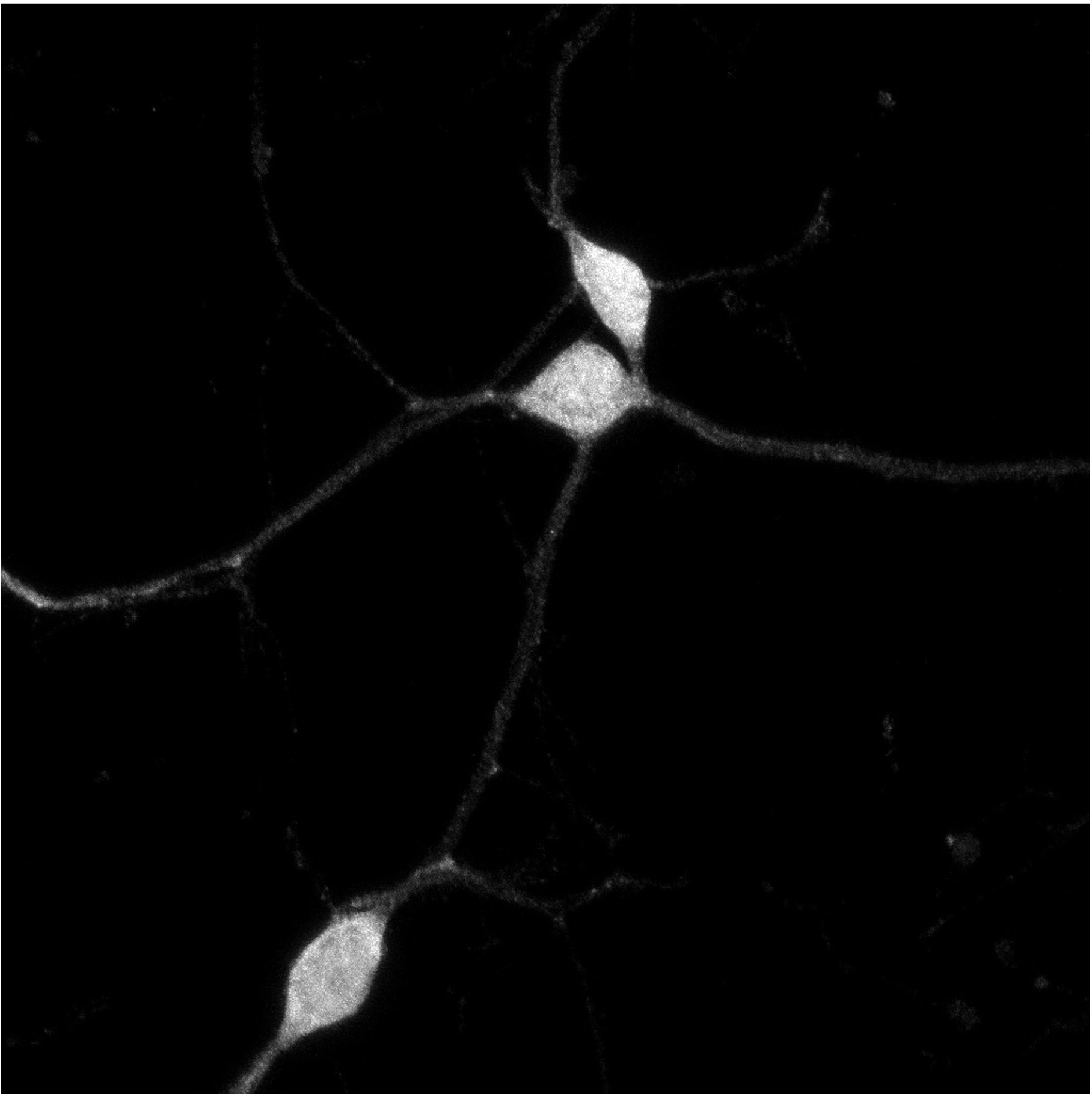

Supplement: Supplementary file 4 — Source data Fig. 2 [file 44318_2025_560_MOESM4_ESM.zip › Figure2/2C/Figure 2C_KT5720_DIV7 neurons_Tuj-1.tif]

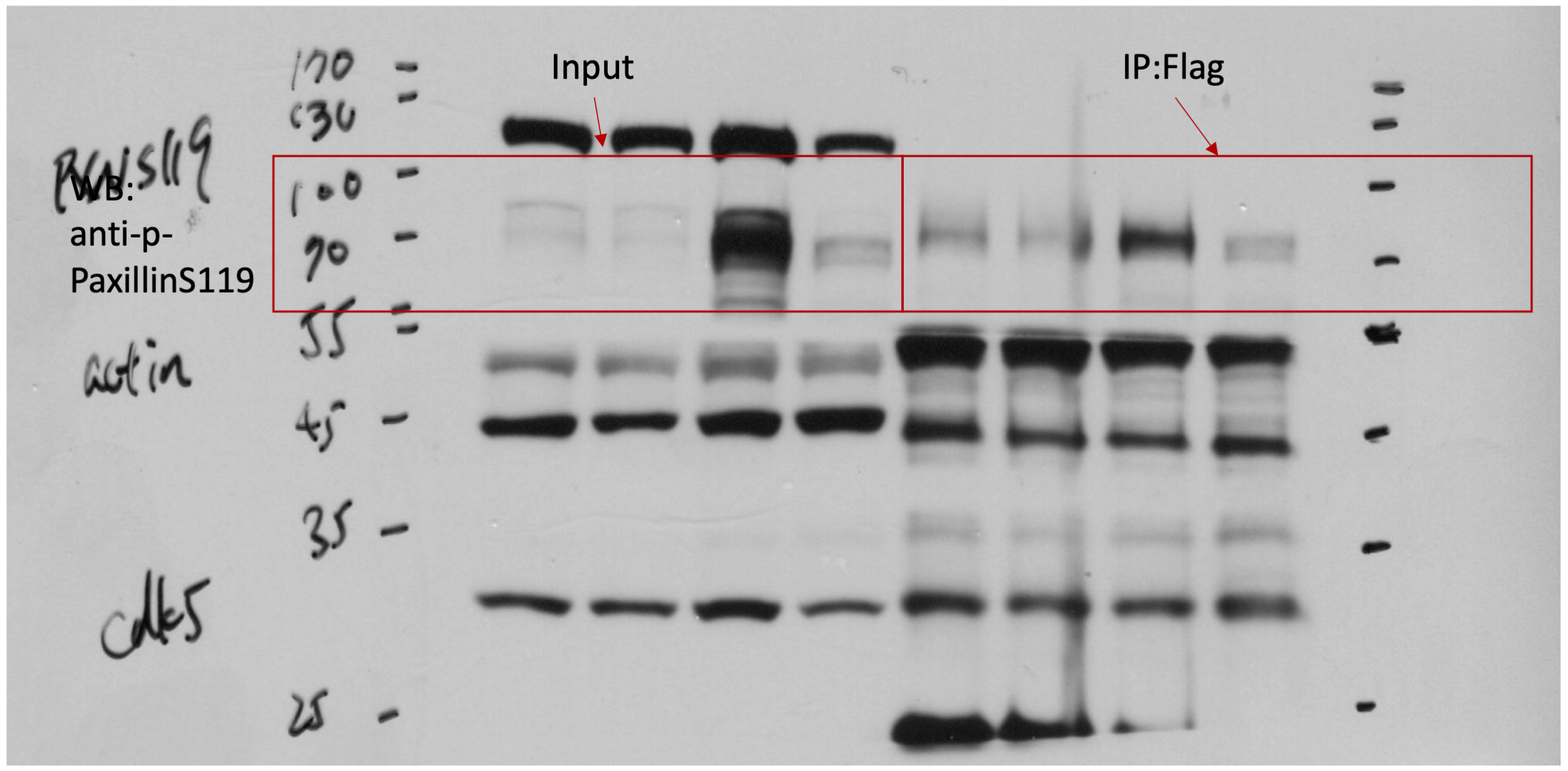

Supplement: Supplementary file 4 — Source data Fig. 2 [file 44318_2025_560_MOESM4_ESM.zip › Figure2/2E/Revision_Figure 2E_DMSO_BDNF_K252a_WB with anti-p-PXNS119_input and Flag-IPed blots.tiff]

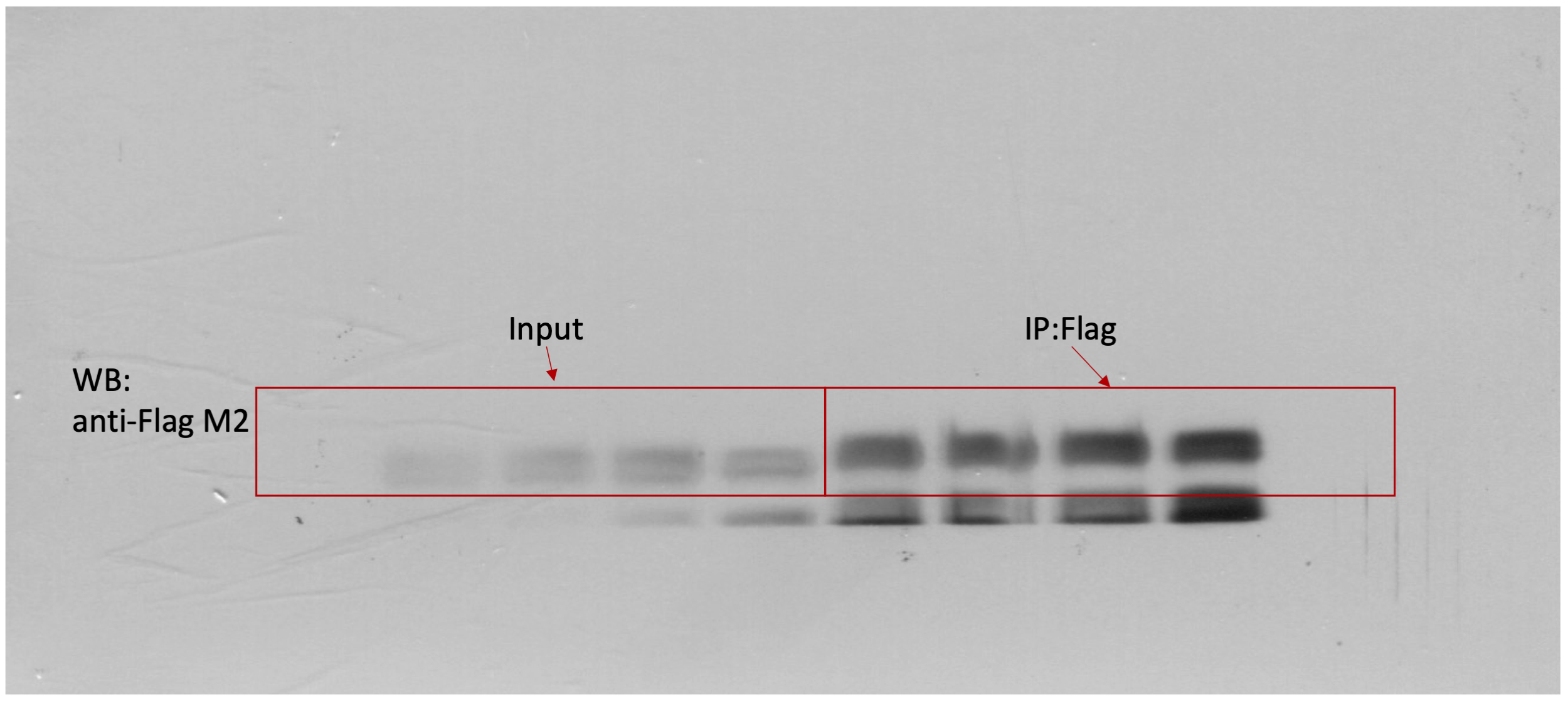

Supplement: Supplementary file 4 — Source data Fig. 2 [file 44318_2025_560_MOESM4_ESM.zip › Figure2/2E/Revision_Figure 2E_DMSO_BDNF_K252a_WB with Flag M2_input and Flag-IPed for p-PXN S119 blots .tiff]

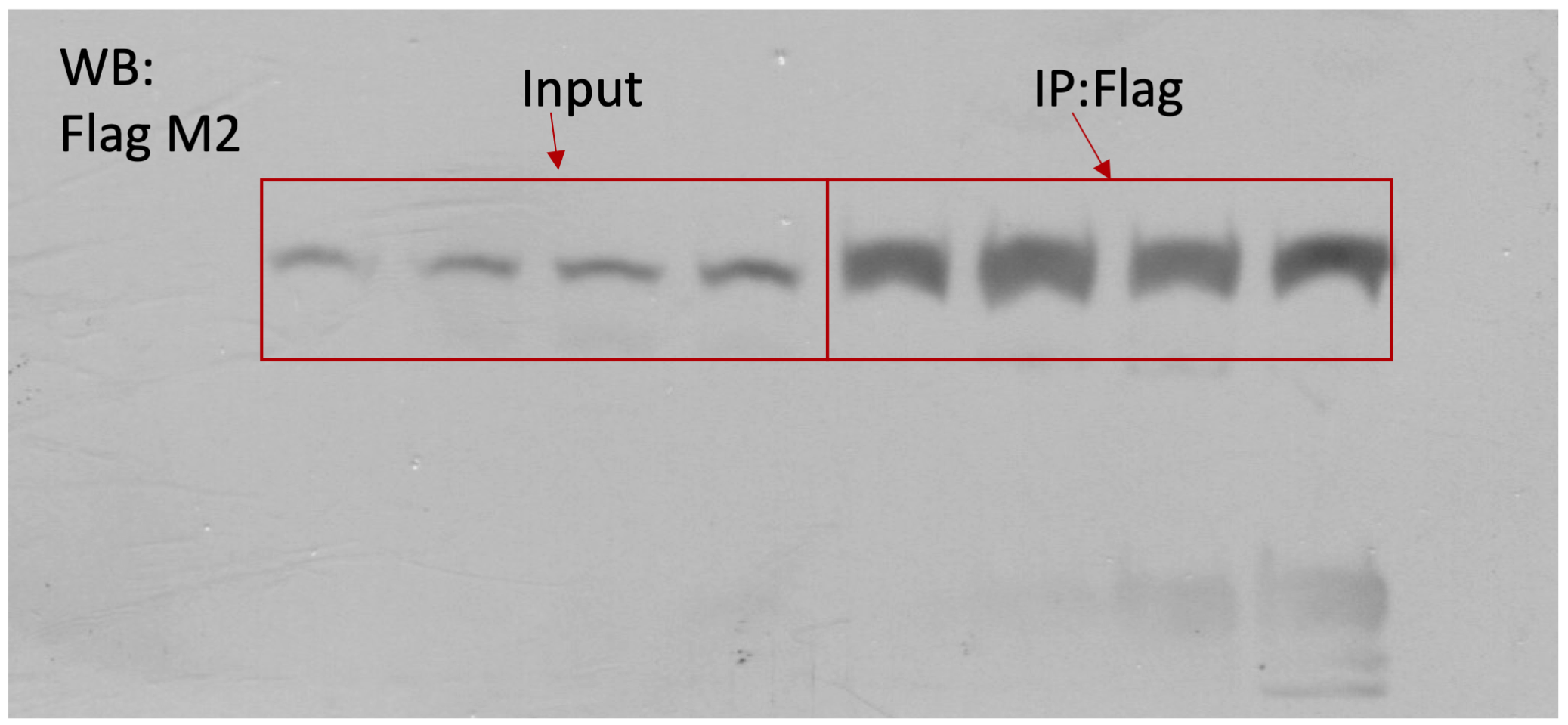

Supplement: Supplementary file 4 — Source data Fig. 2 [file 44318_2025_560_MOESM4_ESM.zip › Figure2/2E/Revision_Figure 2E_DMSO_Frk_KT5720_WB with Flag M2_input and Flag-IPed for p-PXN Y118 blots .tiff]

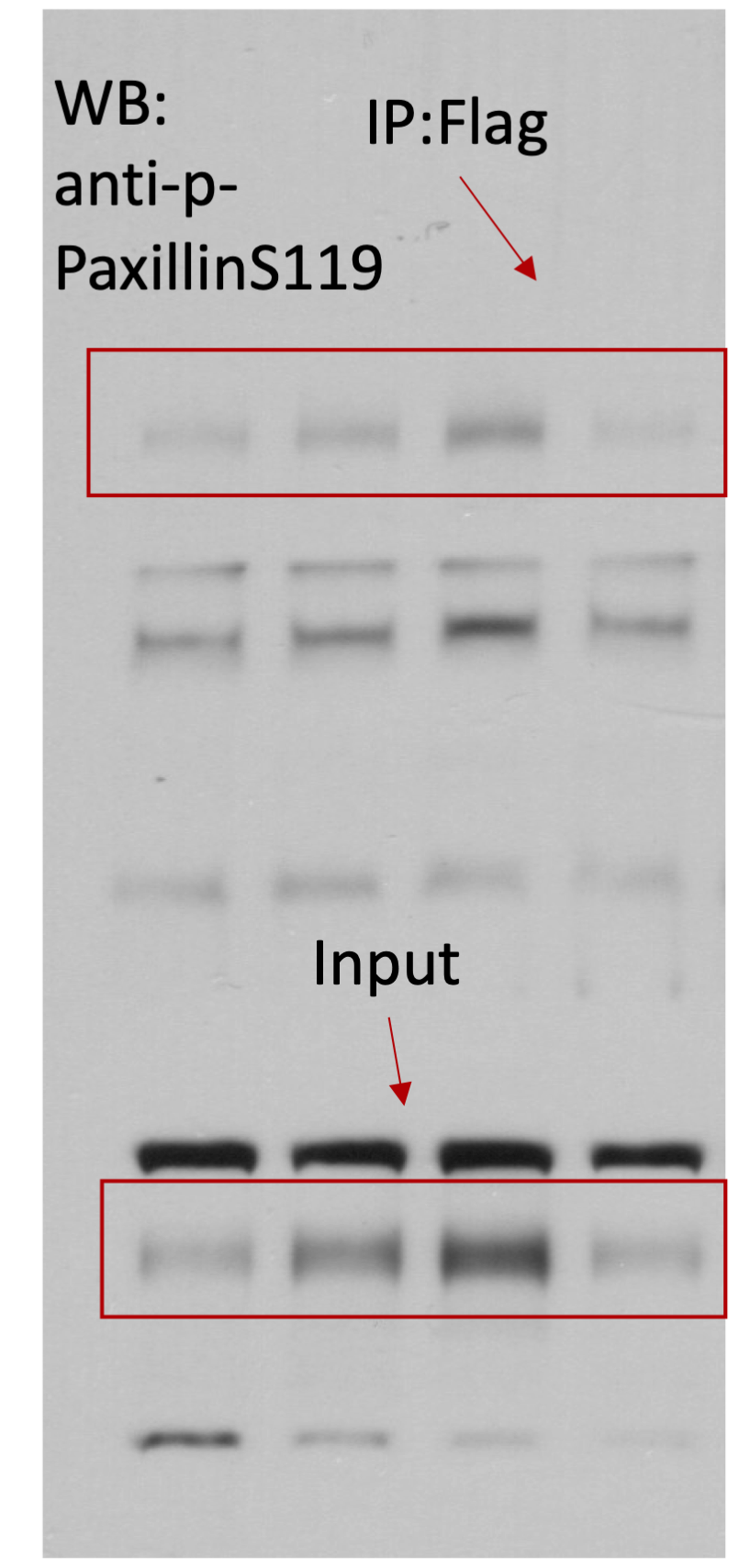

Supplement: Supplementary file 4 — Source data Fig. 2 [file 44318_2025_560_MOESM4_ESM.zip › Figure2/2E/Revision_Figure 2E_DMSO_Frk_KT5720_WB with anti-p-PXNS119_input and Flag-IPed blots.tiff]

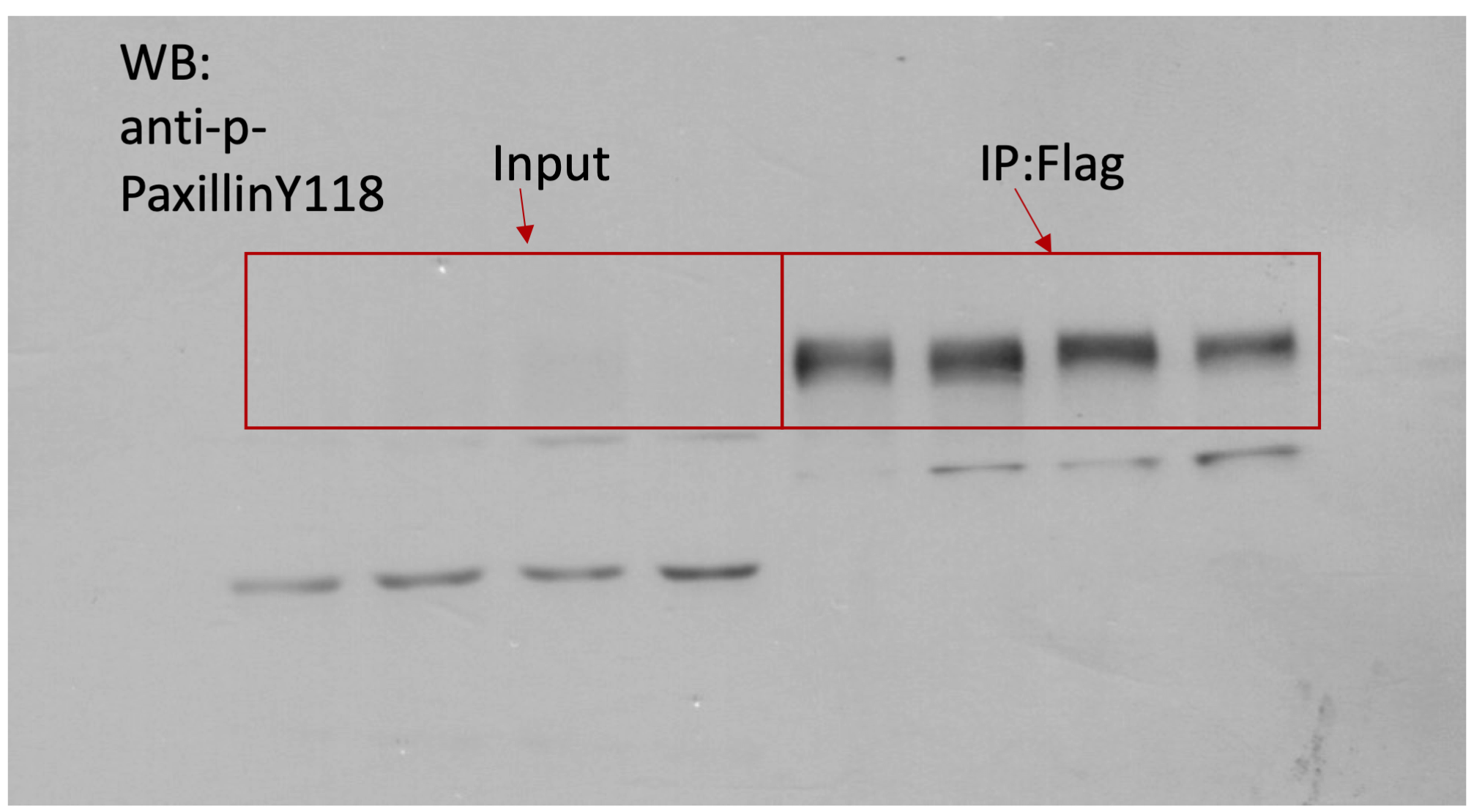

Supplement: Supplementary file 4 — Source data Fig. 2 [file 44318_2025_560_MOESM4_ESM.zip › Figure2/2E/Revision_Figure 2E_DMSO_Frk_KT5720_WB with anti-p-PXNY118_input and Flag-IPed blots.tiff]

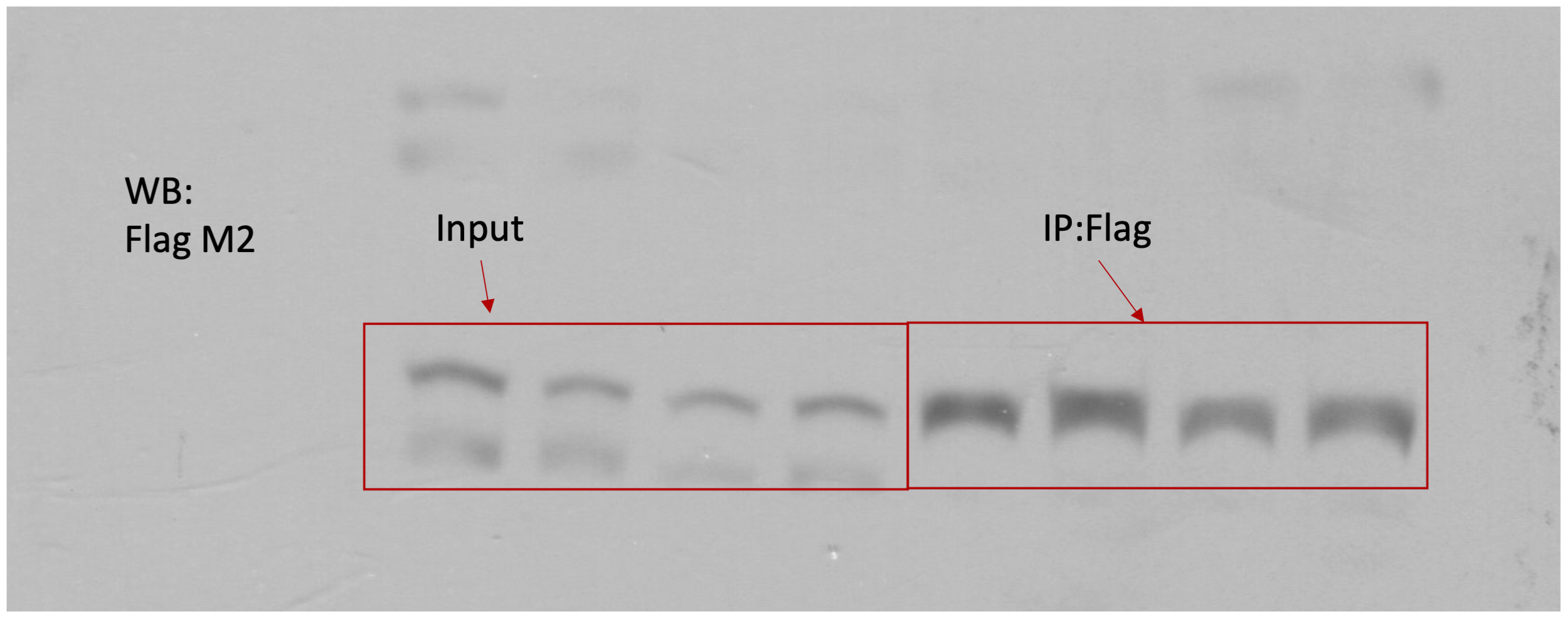

Supplement: Supplementary file 4 — Source data Fig. 2 [file 44318_2025_560_MOESM4_ESM.zip › Figure2/2E/Revision_Figure 2E_DMSO_BDNF_K252a_WB with Flag M2_input and Flag-IPed for p-PXN Y118 blots .tiff]

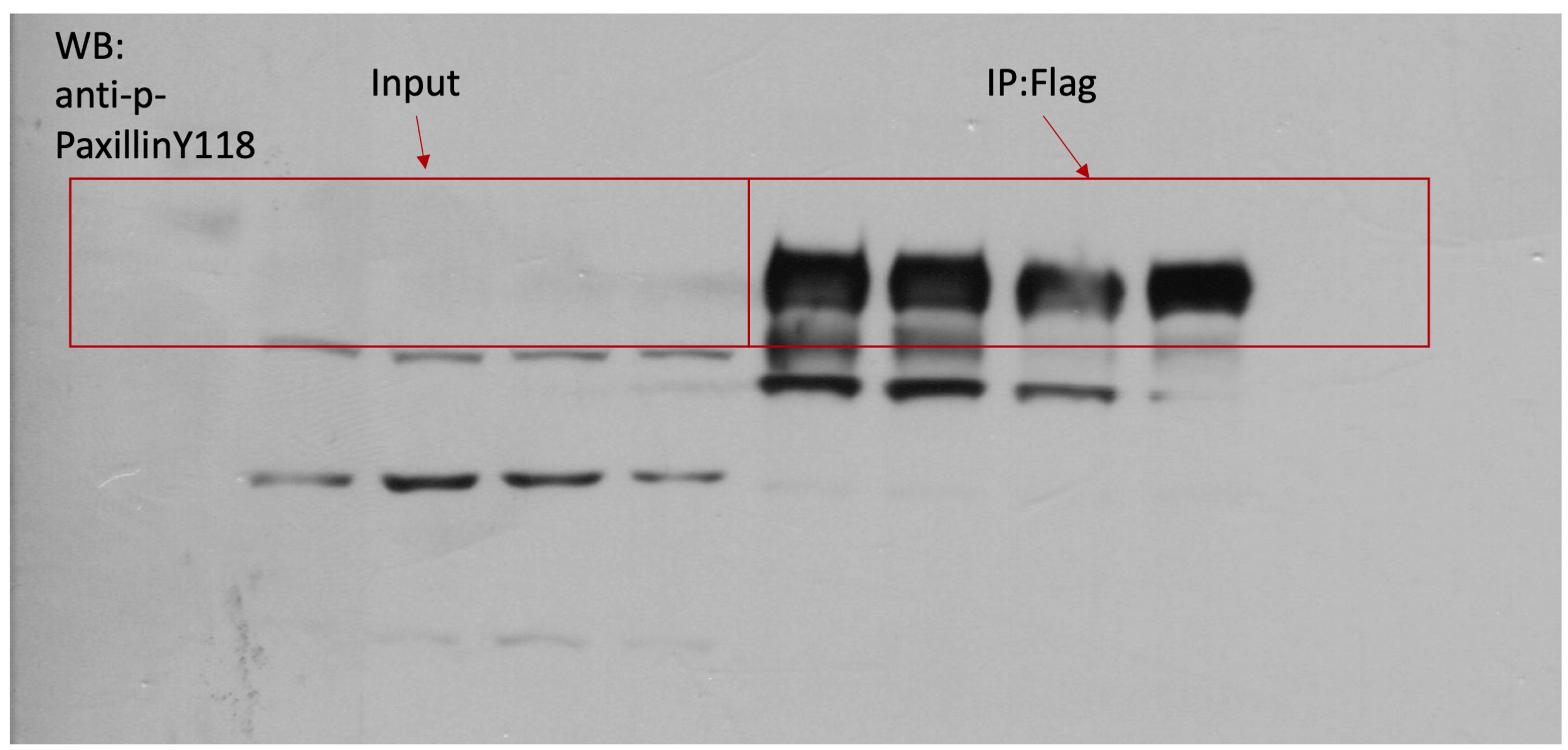

Supplement: Supplementary file 4 — Source data Fig. 2 [file 44318_2025_560_MOESM4_ESM.zip › Figure2/2E/Revision_Figure 2E_DMSO_BDNF_K252a_WB with anti-p-PXNY118_input and Flag-IPed blots.tiff]

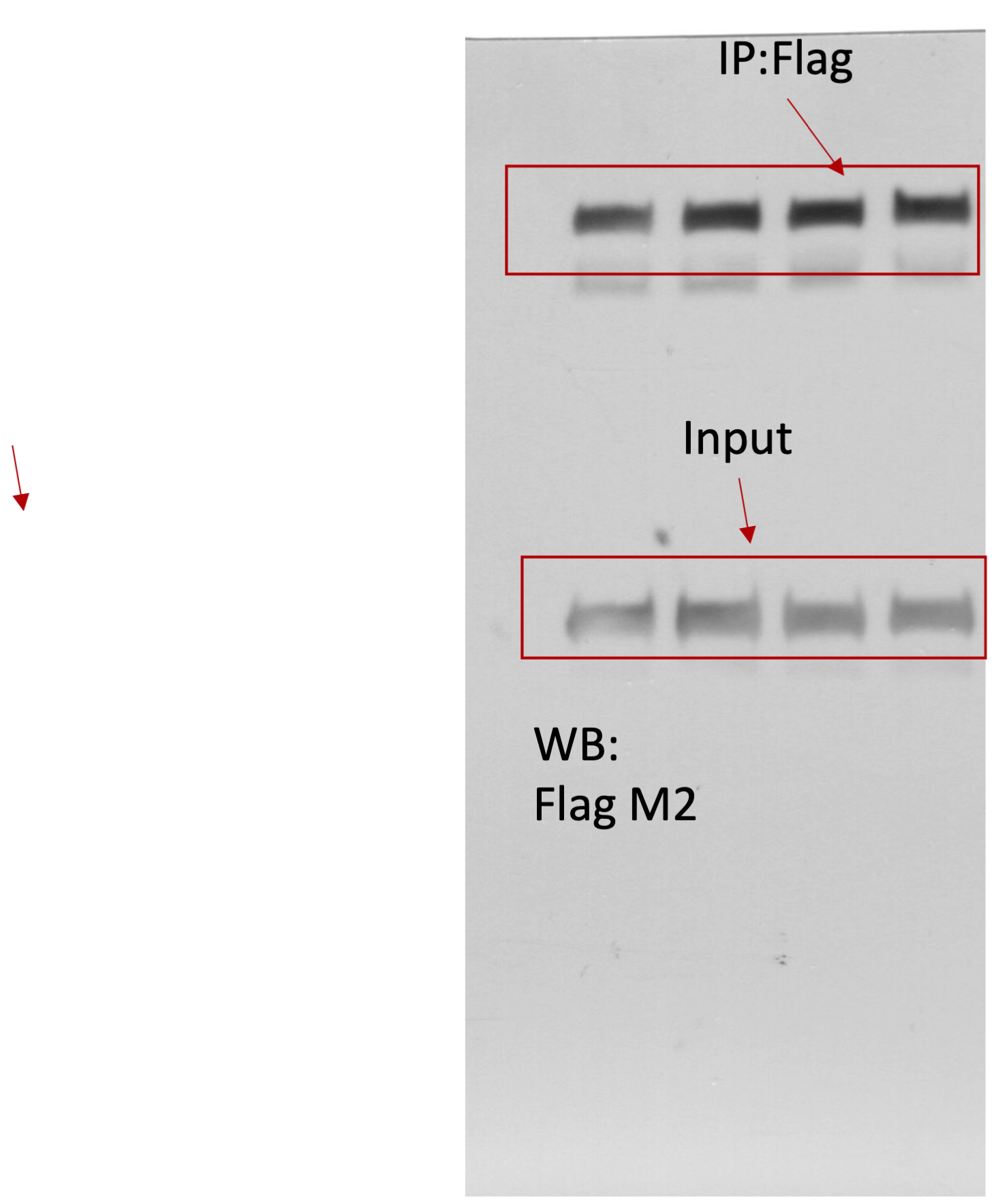

Supplement: Supplementary file 4 — Source data Fig. 2 [file 44318_2025_560_MOESM4_ESM.zip › Figure2/2E/Revision_Figure 2E_DMSO_Frk_KT5720_WB with Flag M2_input and Flag-IPed for p-PXN S119 blots .tiff]

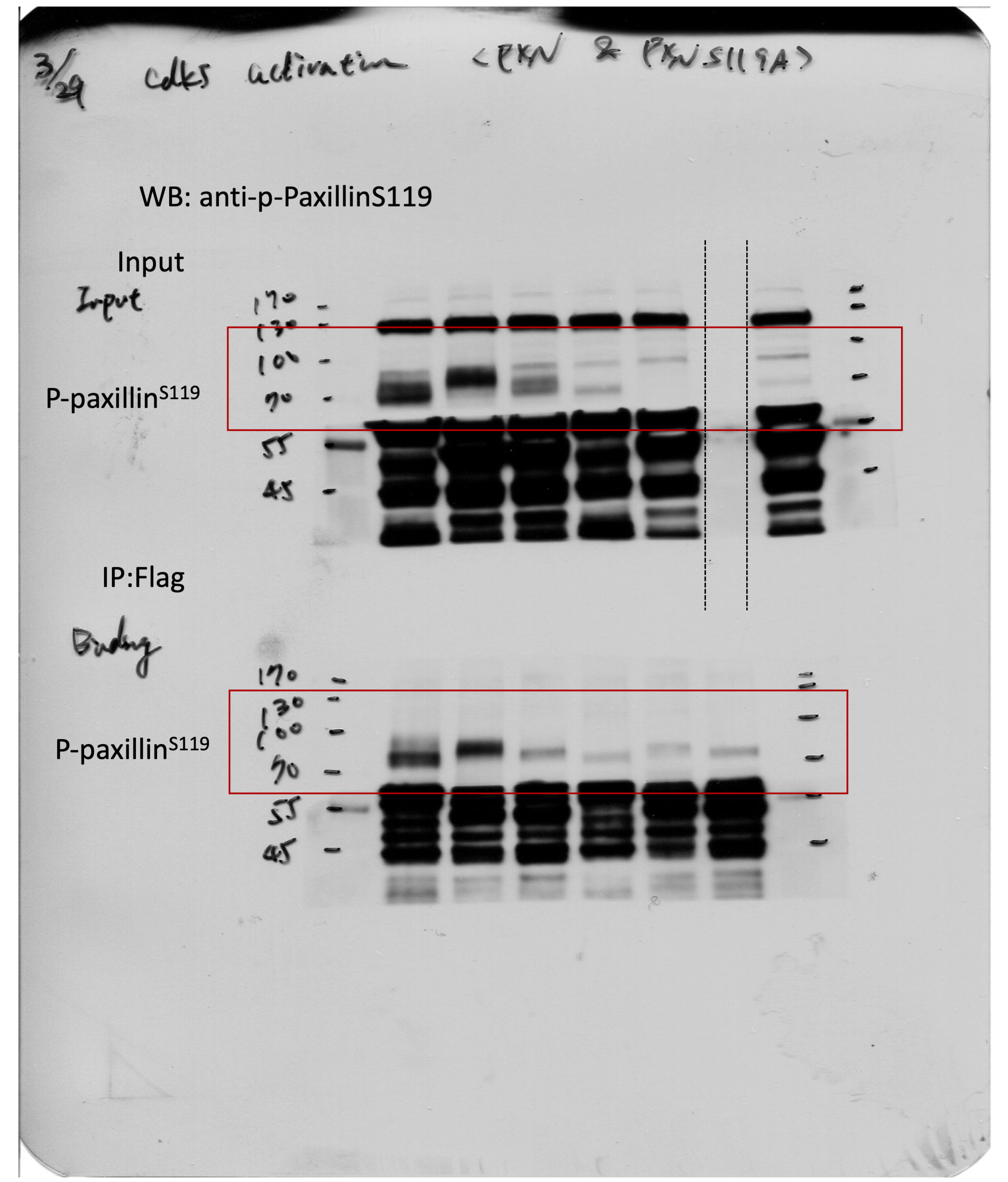

Supplement: Supplementary file 4 — Source data Fig. 2 [file 44318_2025_560_MOESM4_ESM.zip › Figure2/2G/Revision_Figure 2G_WB with anti-p-PXN S119_input and Flag-IPed blots.tiff]

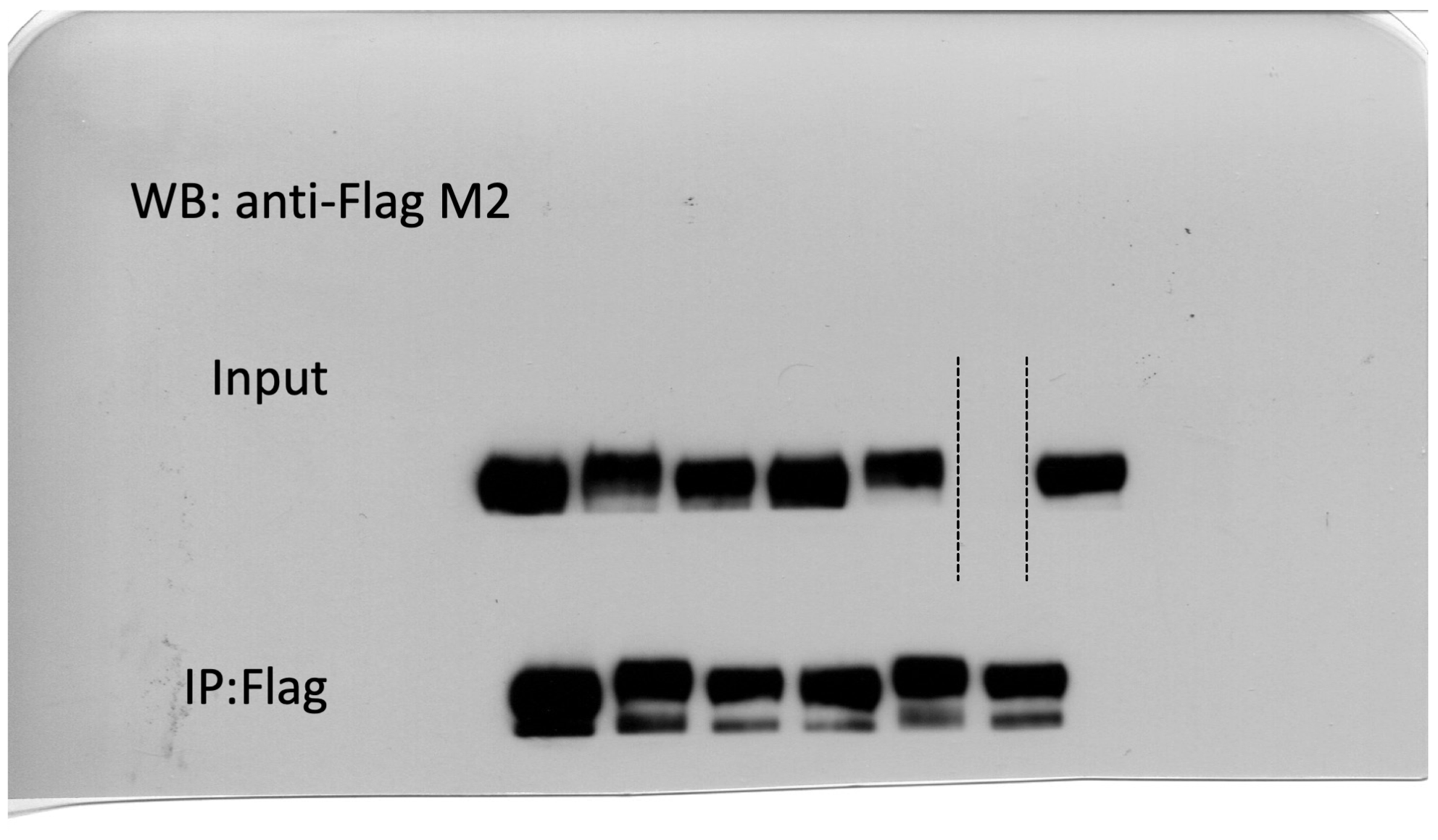

Supplement: Supplementary file 4 — Source data Fig. 2 [file 44318_2025_560_MOESM4_ESM.zip › Figure2/2G/Revision_Figure 2G_WB with Flag M2_input and Flag-IPed blots.tiff]
