# Supplementary figures and images for: Nuclear paxillin functions as a molecular switch for alternative splicing in neurons during a critical period of brain development (part 2 of 2)
Source: EMBO J. 2025 Sep 9;44(21):5965–92. doi: 10.1038/s44318-025-00560-8 (PMC12583701; doi:10.1038/s44318-025-00560-8)

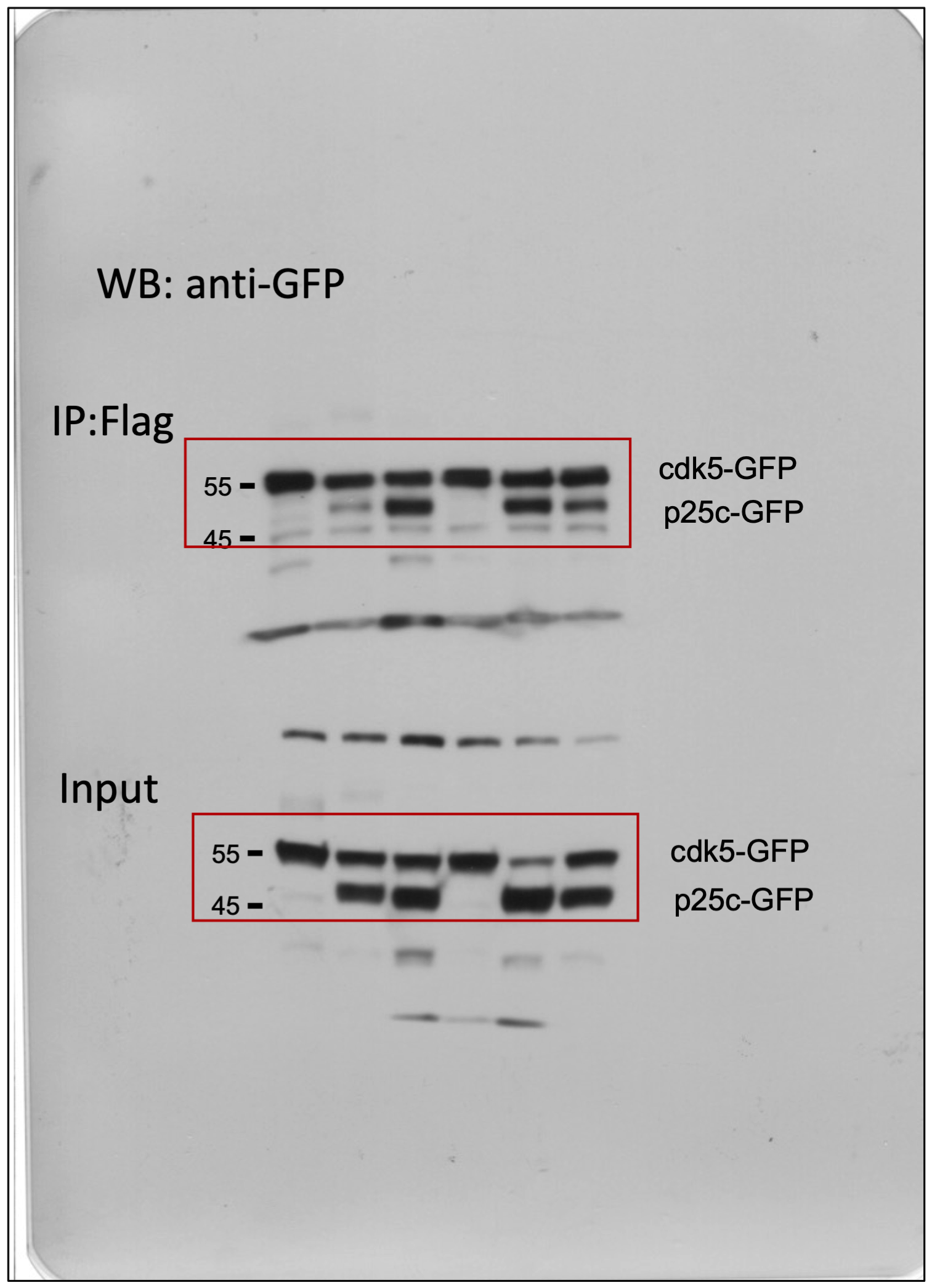

Supplement: Supplementary file 4 — Source data Fig. 2 [file 44318_2025_560_MOESM4_ESM.zip › Figure2/2G/Revision_Figure 2G_WB with anti-GFP_inpur and Flad-IPed blots.tiff]

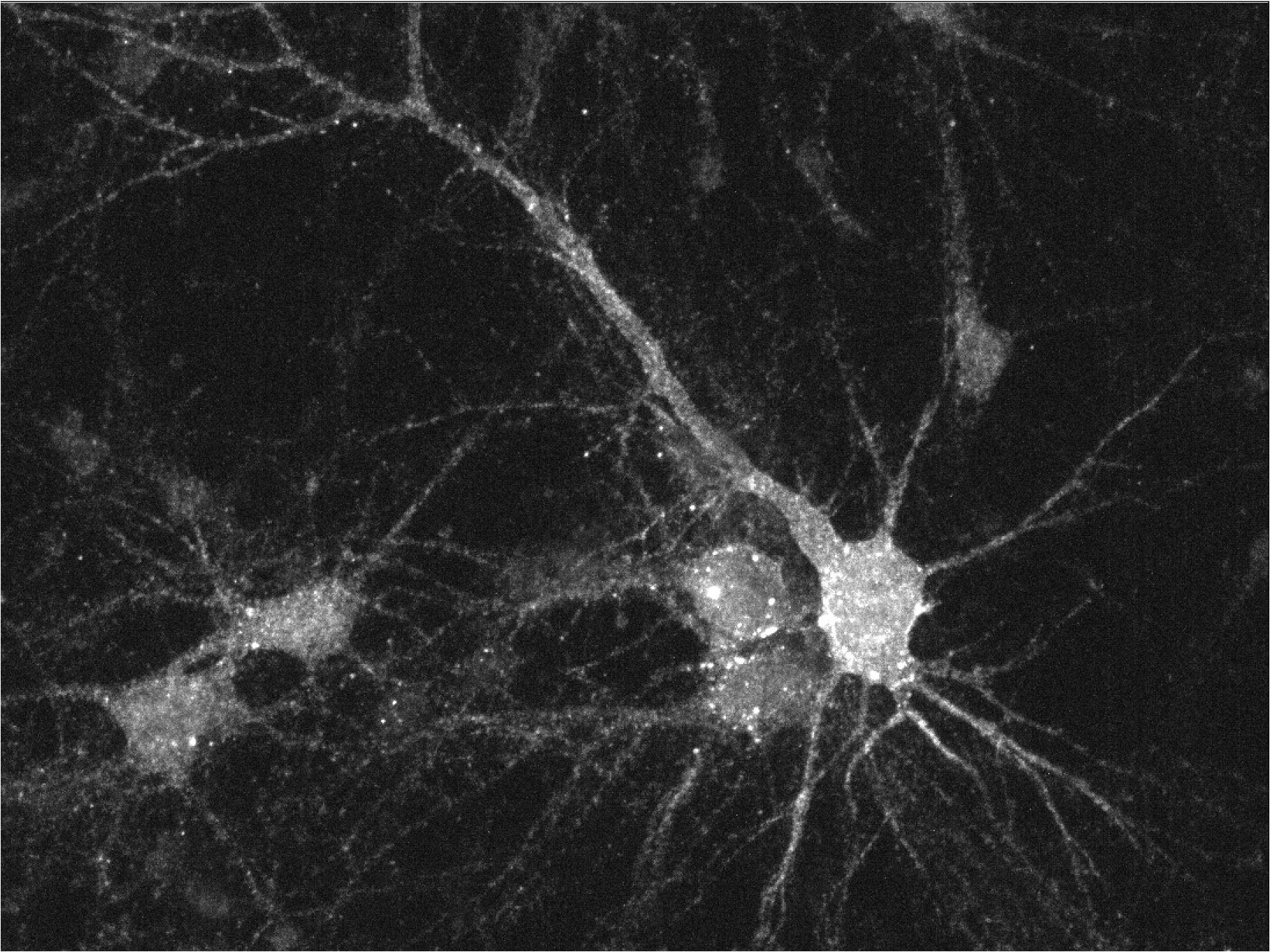

Supplement: Supplementary file 4 — Source data Fig. 2 [file 44318_2025_560_MOESM4_ESM.zip › Figure2/2I/Figure2I_NMDA_PurvalA_DIV14_p-PXNS119.tif]

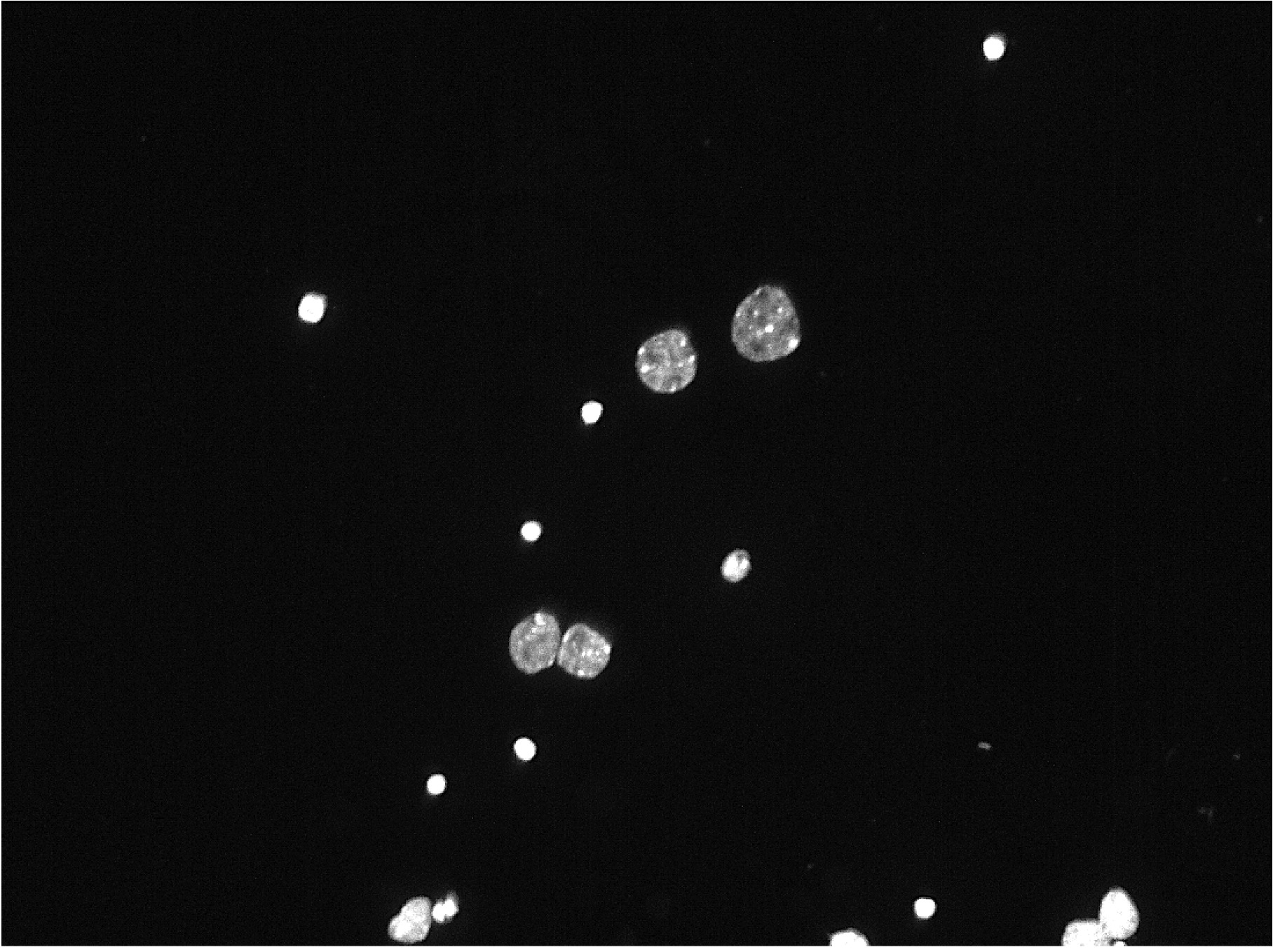

Supplement: Supplementary file 4 — Source data Fig. 2 [file 44318_2025_560_MOESM4_ESM.zip › Figure2/2I/Figure2I_Regular medium_DIV14_DAPI.tif]

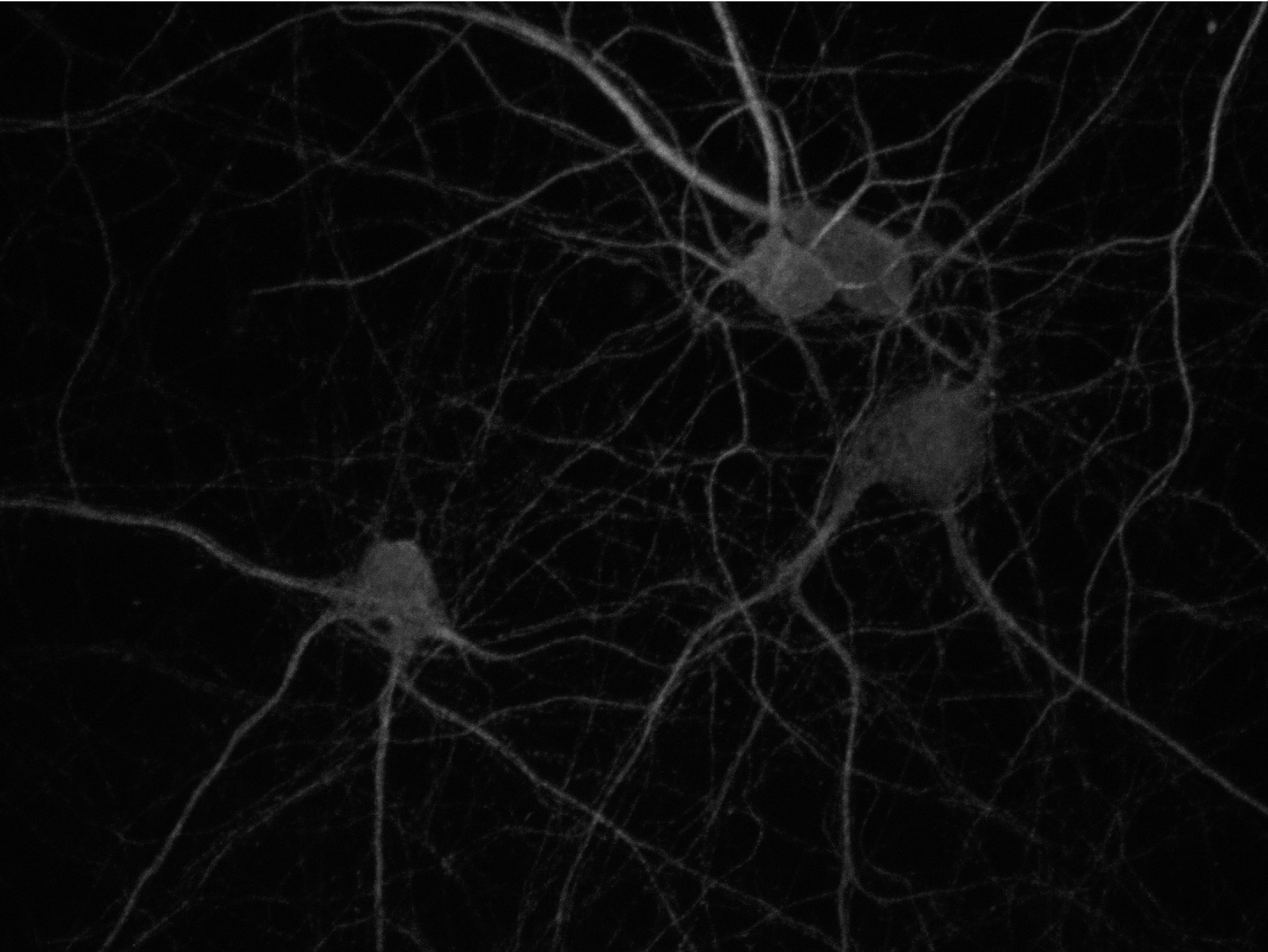

Supplement: Supplementary file 4 — Source data Fig. 2 [file 44318_2025_560_MOESM4_ESM.zip › Figure2/2I/Figure2I_NMDA_DIV14_Tuj-1.tif]

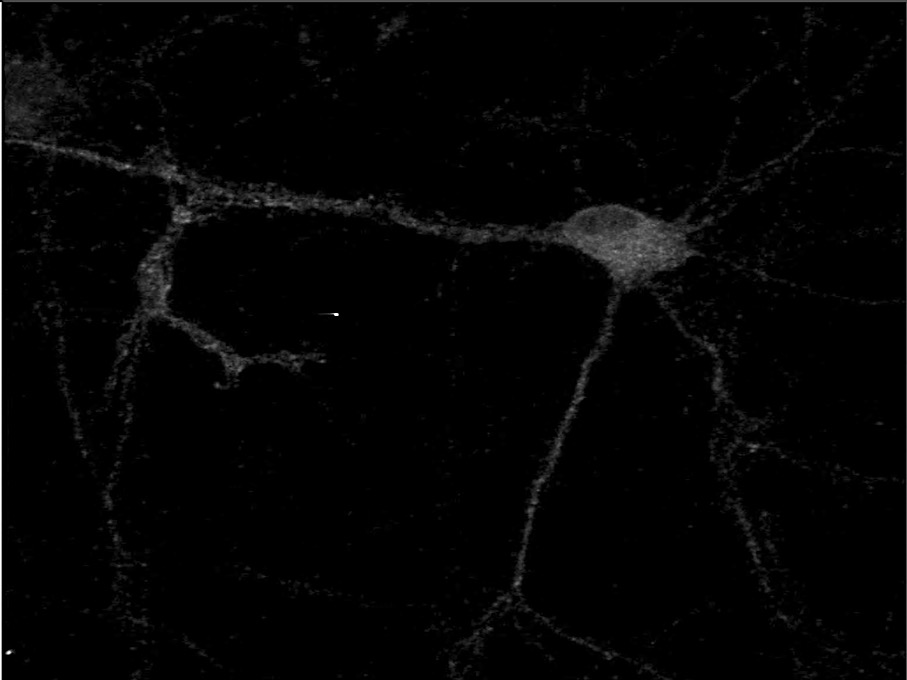

Supplement: Supplementary file 4 — Source data Fig. 2 [file 44318_2025_560_MOESM4_ESM.zip › Figure2/2I/Figure2I_NMDA_CNQX_DIV14_p-PXNS119.tif]

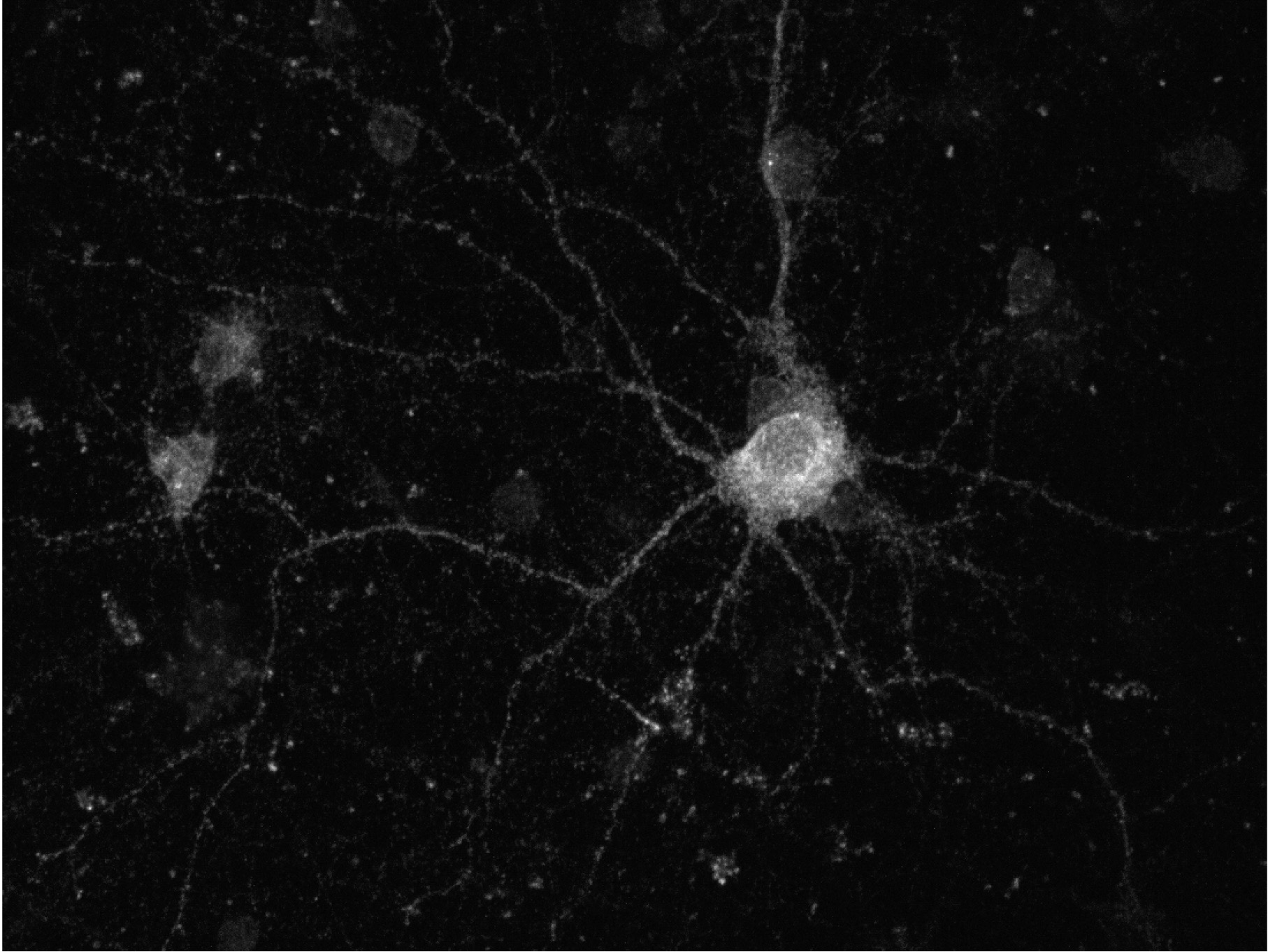

Supplement: Supplementary file 4 — Source data Fig. 2 [file 44318_2025_560_MOESM4_ESM.zip › Figure2/2I/Figure2I_NMDA_IPZ_DIV14_p-PXNS119.tif]

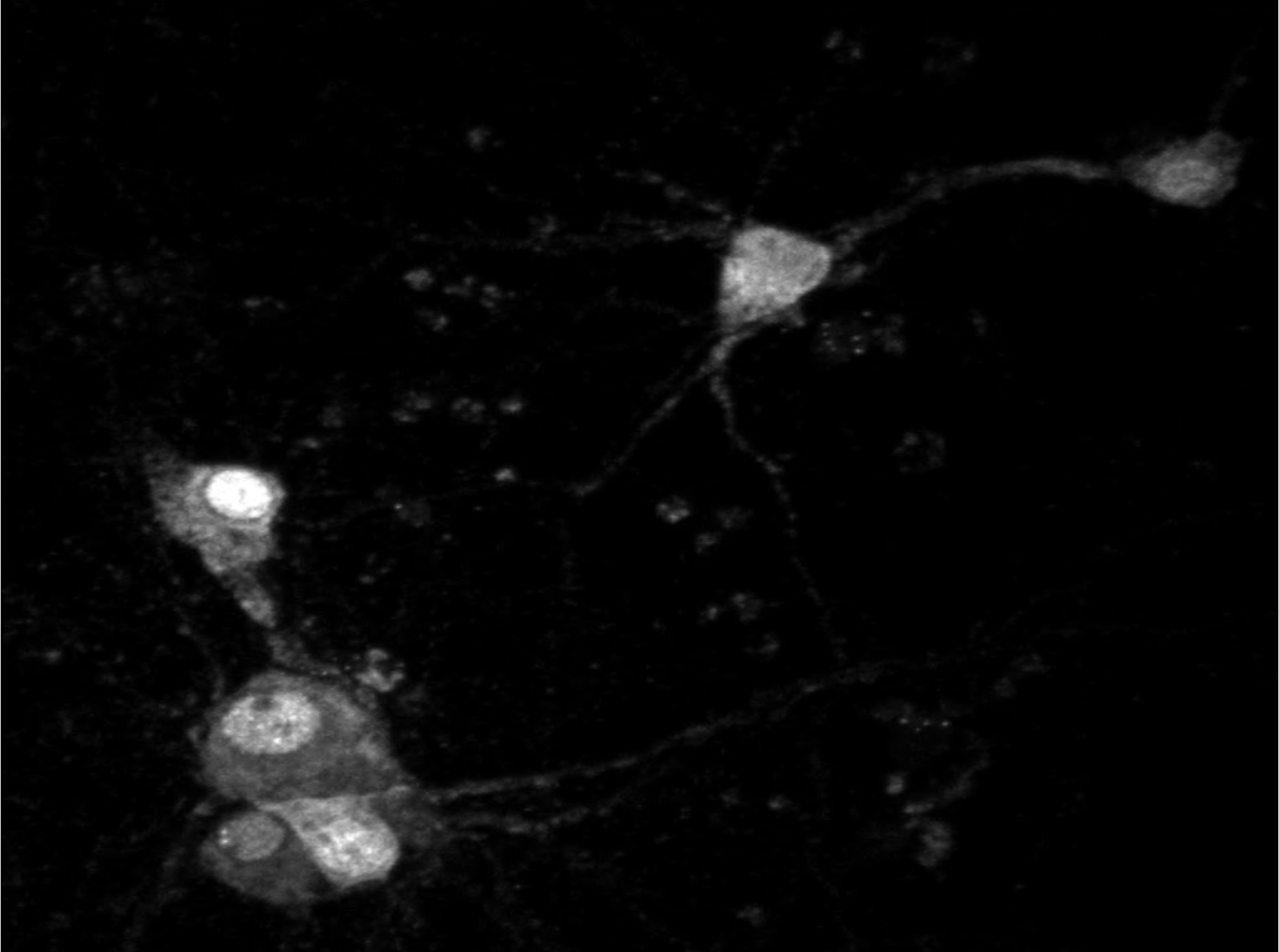

Supplement: Supplementary file 4 — Source data Fig. 2 [file 44318_2025_560_MOESM4_ESM.zip › Figure2/2I/Figure2I_NMDA_APV_DIV14_Tuj-1.tif]

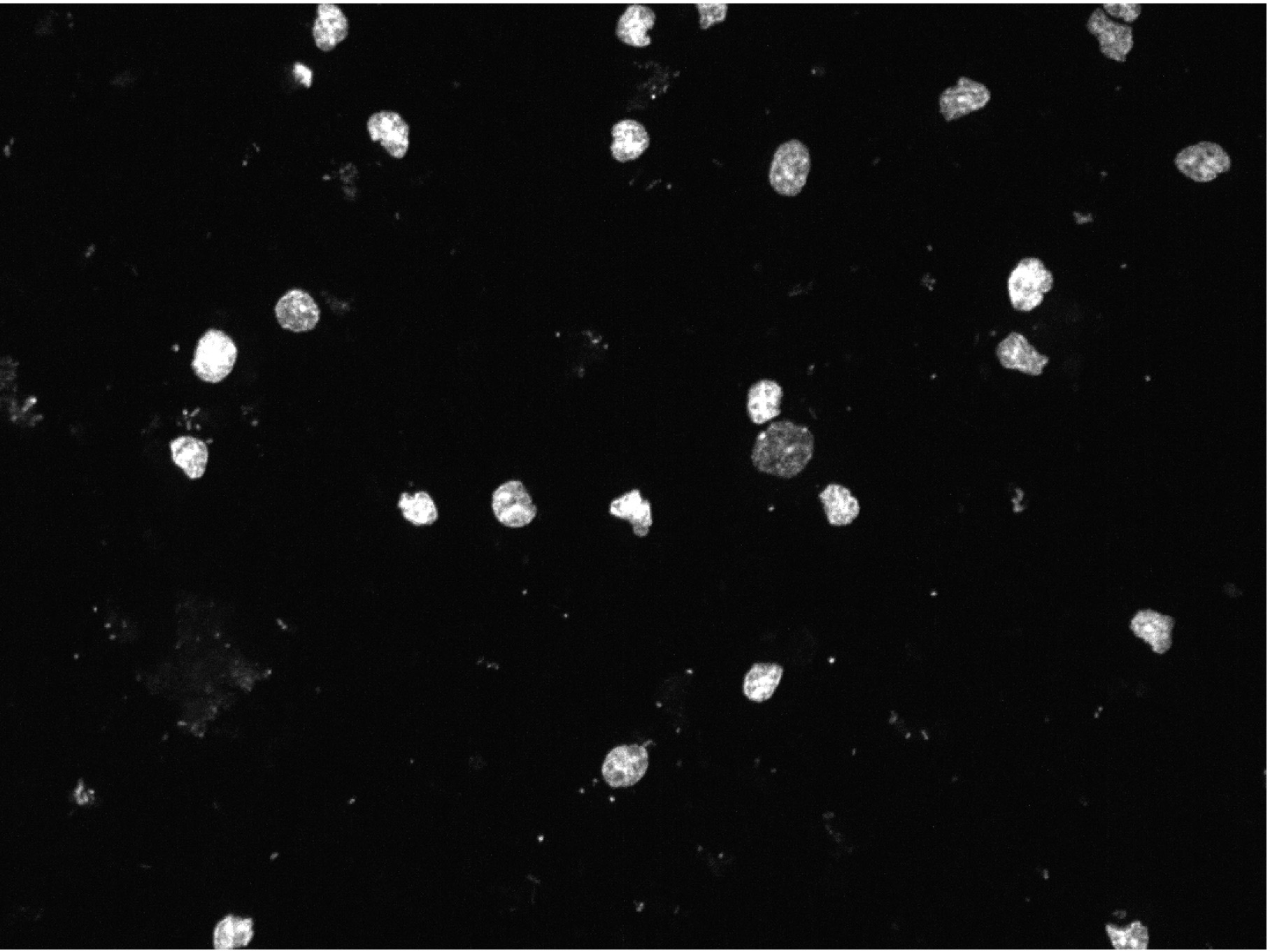

Supplement: Supplementary file 4 — Source data Fig. 2 [file 44318_2025_560_MOESM4_ESM.zip › Figure2/2I/Figure2I_NMDA_IPZ_DIV14_DAPI.tif]

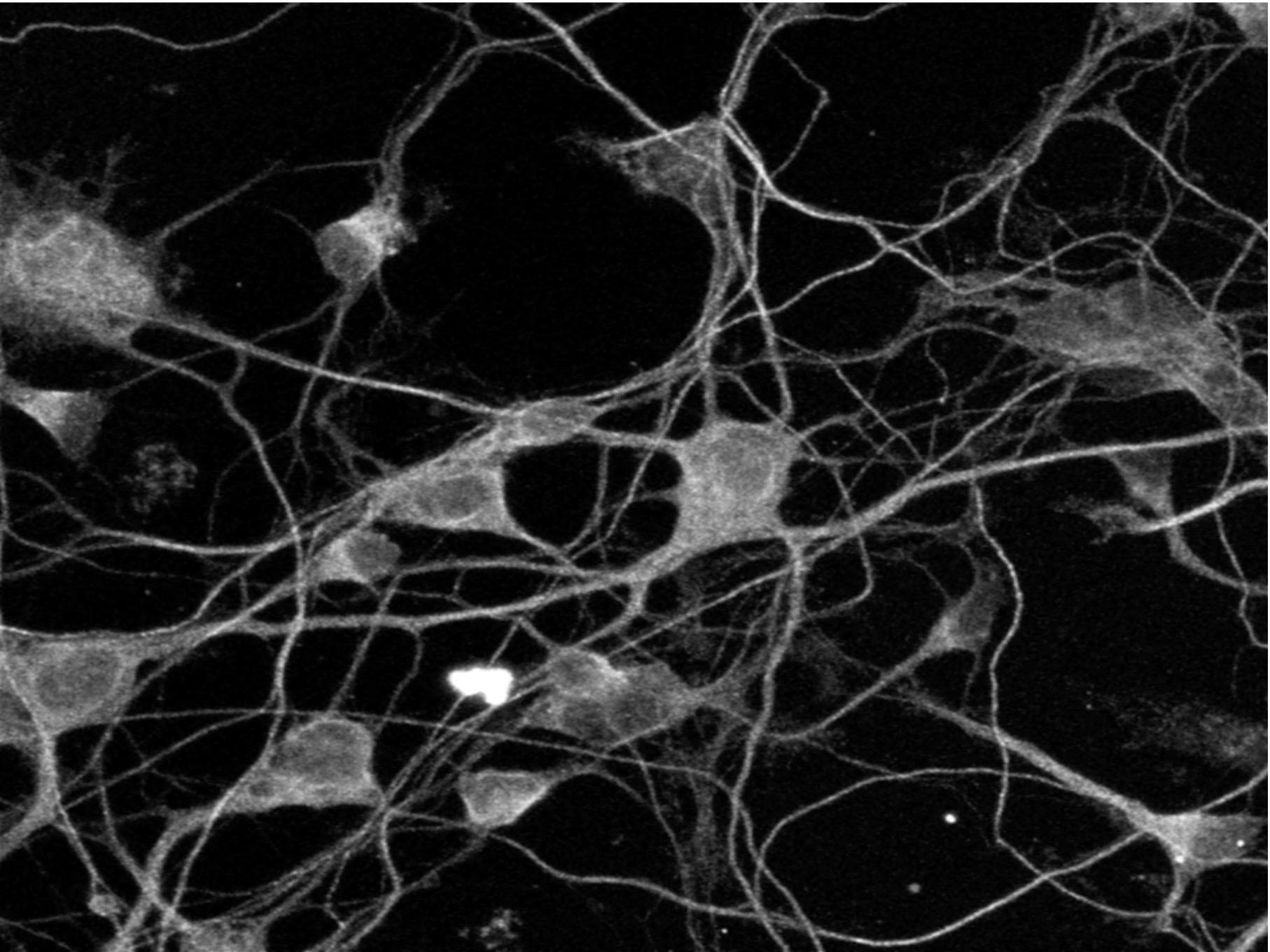

Supplement: Supplementary file 4 — Source data Fig. 2 [file 44318_2025_560_MOESM4_ESM.zip › Figure2/2I/Figure2I_Regular medium_DIV7_Tuj-1.tif]

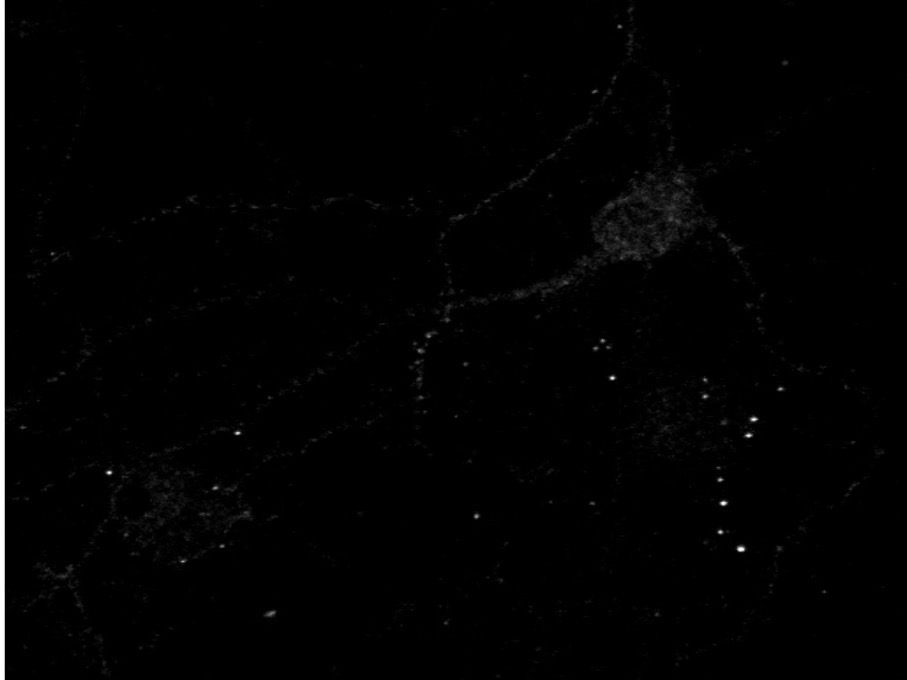

Supplement: Supplementary file 4 — Source data Fig. 2 [file 44318_2025_560_MOESM4_ESM.zip › Figure2/2I/Figure2I_NMDA_KN62_DIV14_p-PXNS119.tif]

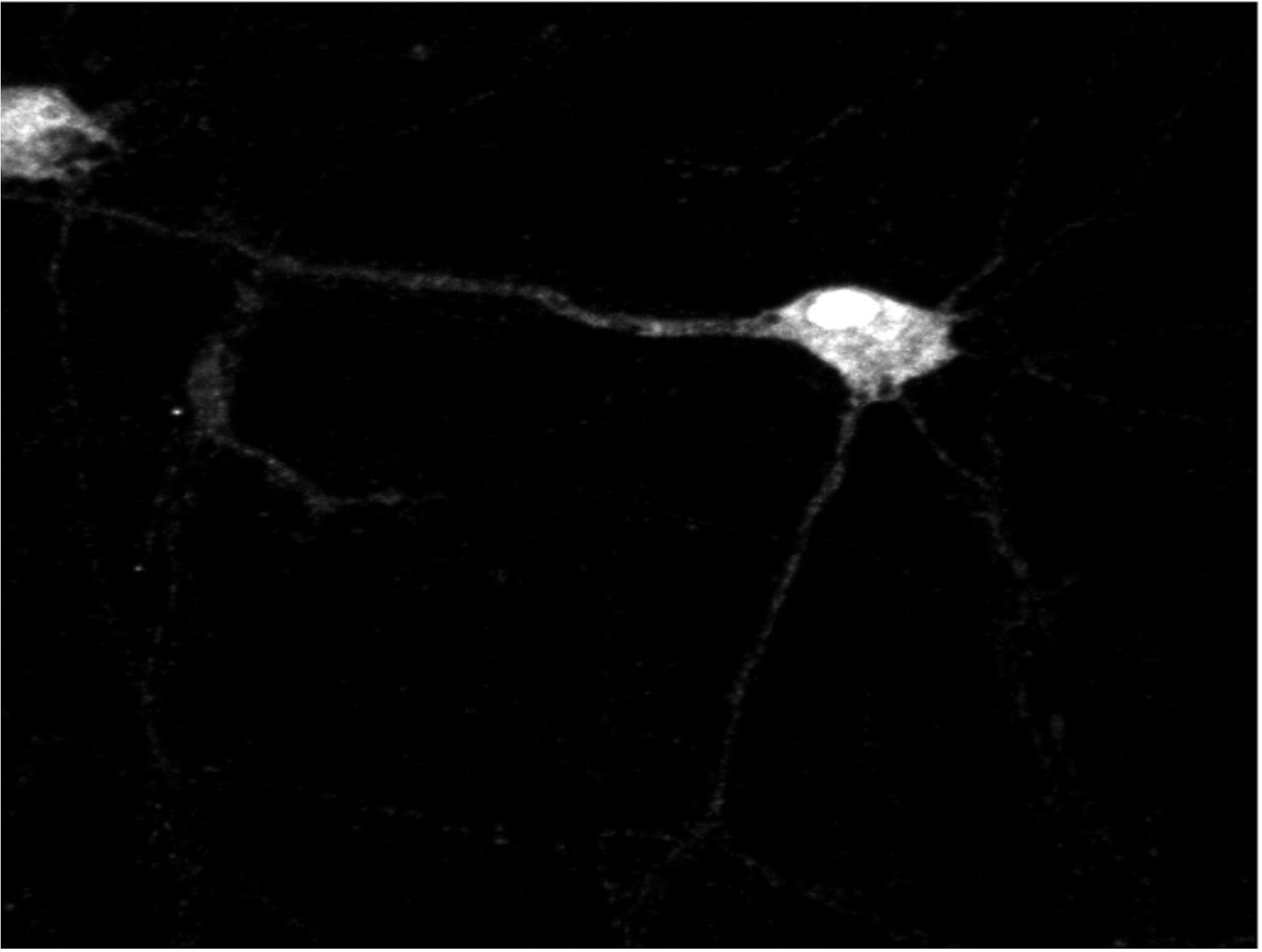

Supplement: Supplementary file 4 — Source data Fig. 2 [file 44318_2025_560_MOESM4_ESM.zip › Figure2/2I/Figure2I_NMDA_CNQX_DIV14_Tuj-1.tif]

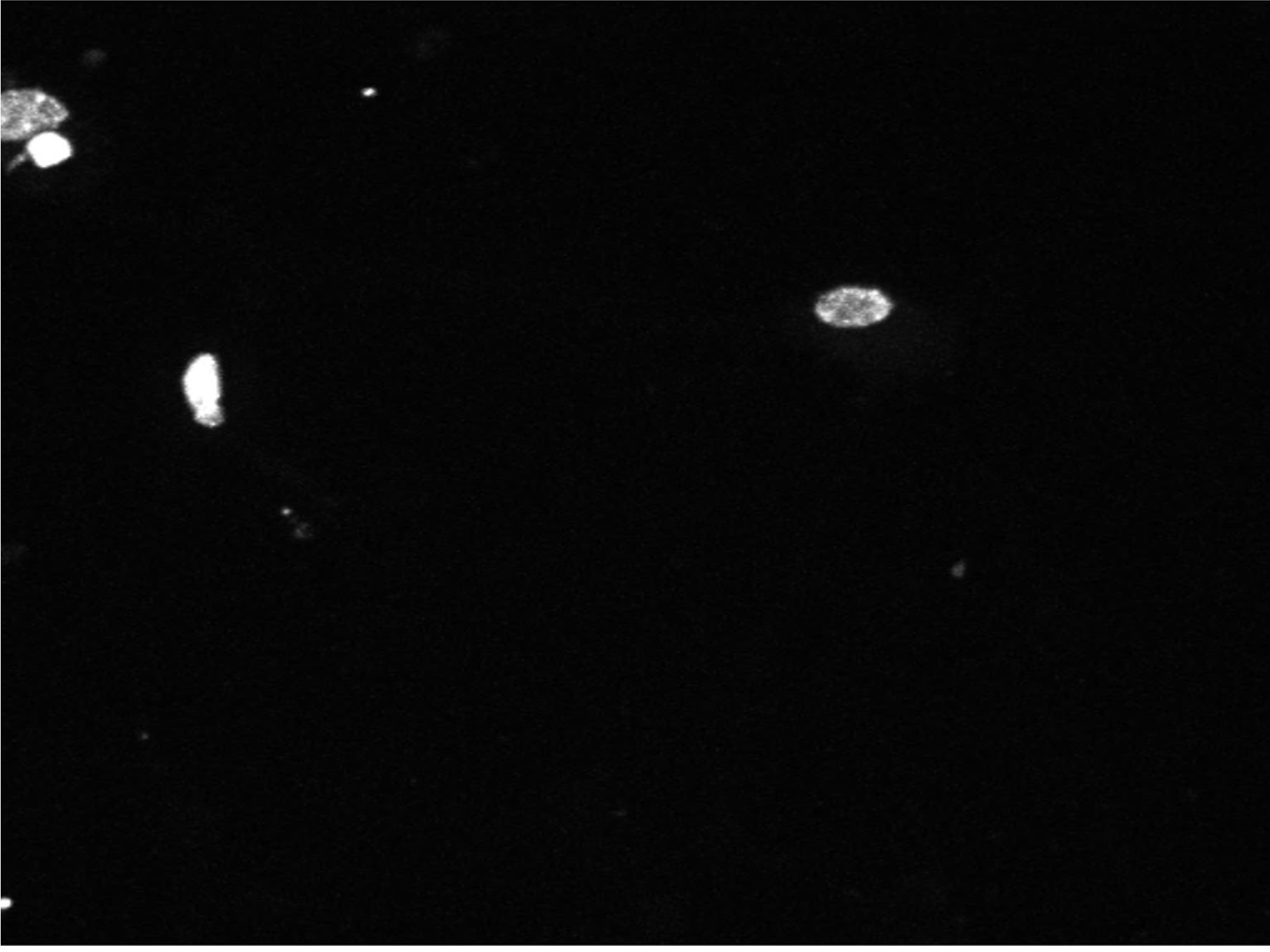

Supplement: Supplementary file 4 — Source data Fig. 2 [file 44318_2025_560_MOESM4_ESM.zip › Figure2/2I/Figure2I_NMDA_CNQX_DIV14_DAPI.tif]

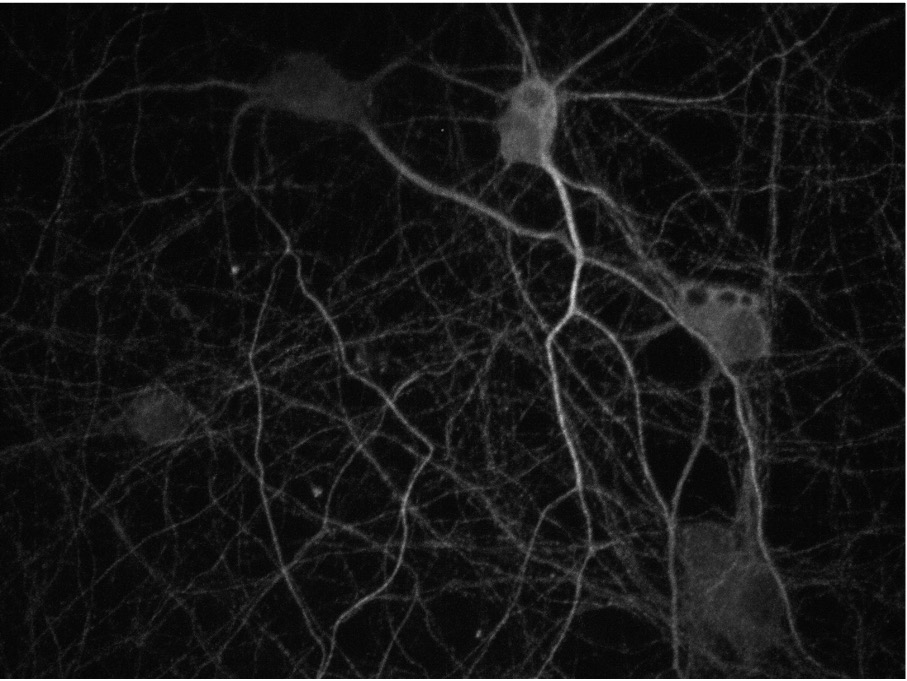

Supplement: Supplementary file 4 — Source data Fig. 2 [file 44318_2025_560_MOESM4_ESM.zip › Figure2/2I/Figure2I_NMDA_KT5720_DIV14_Tuj-1.tif]

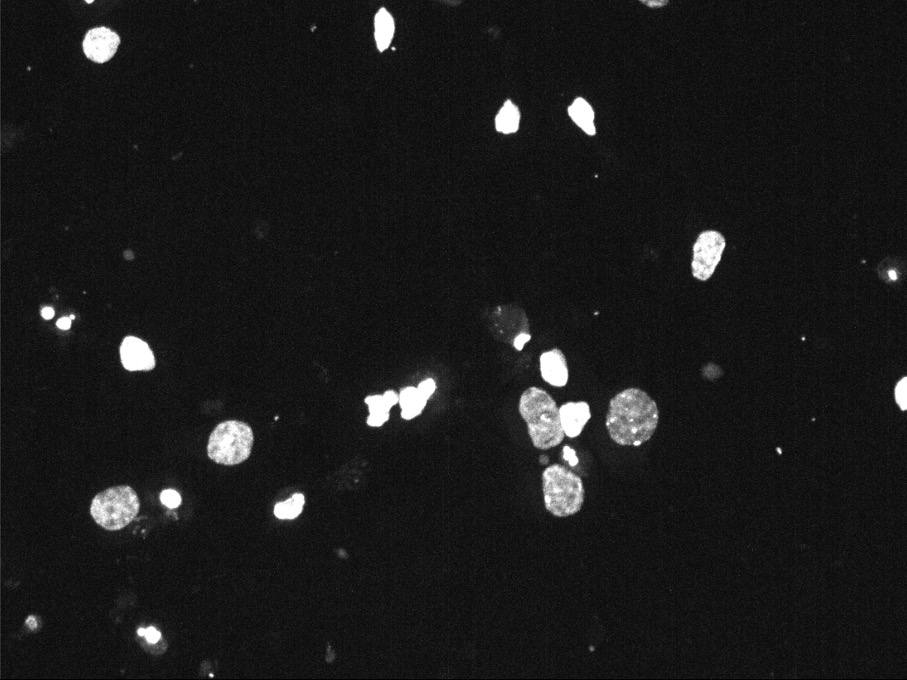

Supplement: Supplementary file 4 — Source data Fig. 2 [file 44318_2025_560_MOESM4_ESM.zip › Figure2/2I/Figure2I_NMDA_PurvalA_DIV14_DAPI.tif]

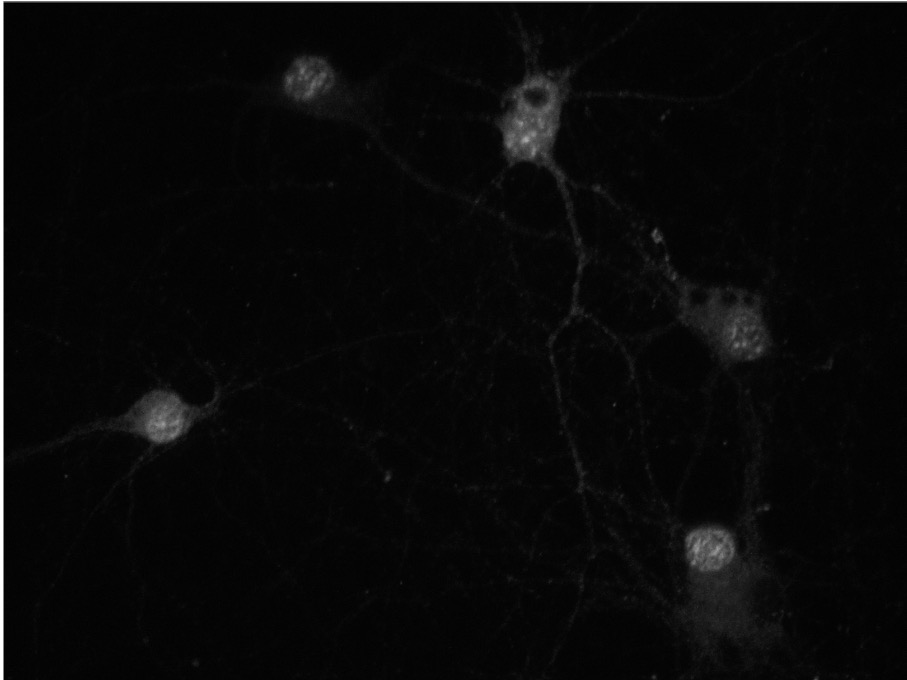

Supplement: Supplementary file 4 — Source data Fig. 2 [file 44318_2025_560_MOESM4_ESM.zip › Figure2/2I/Figure2I_NMDA_KT5720_DIV14_p-PXNS119.tif]

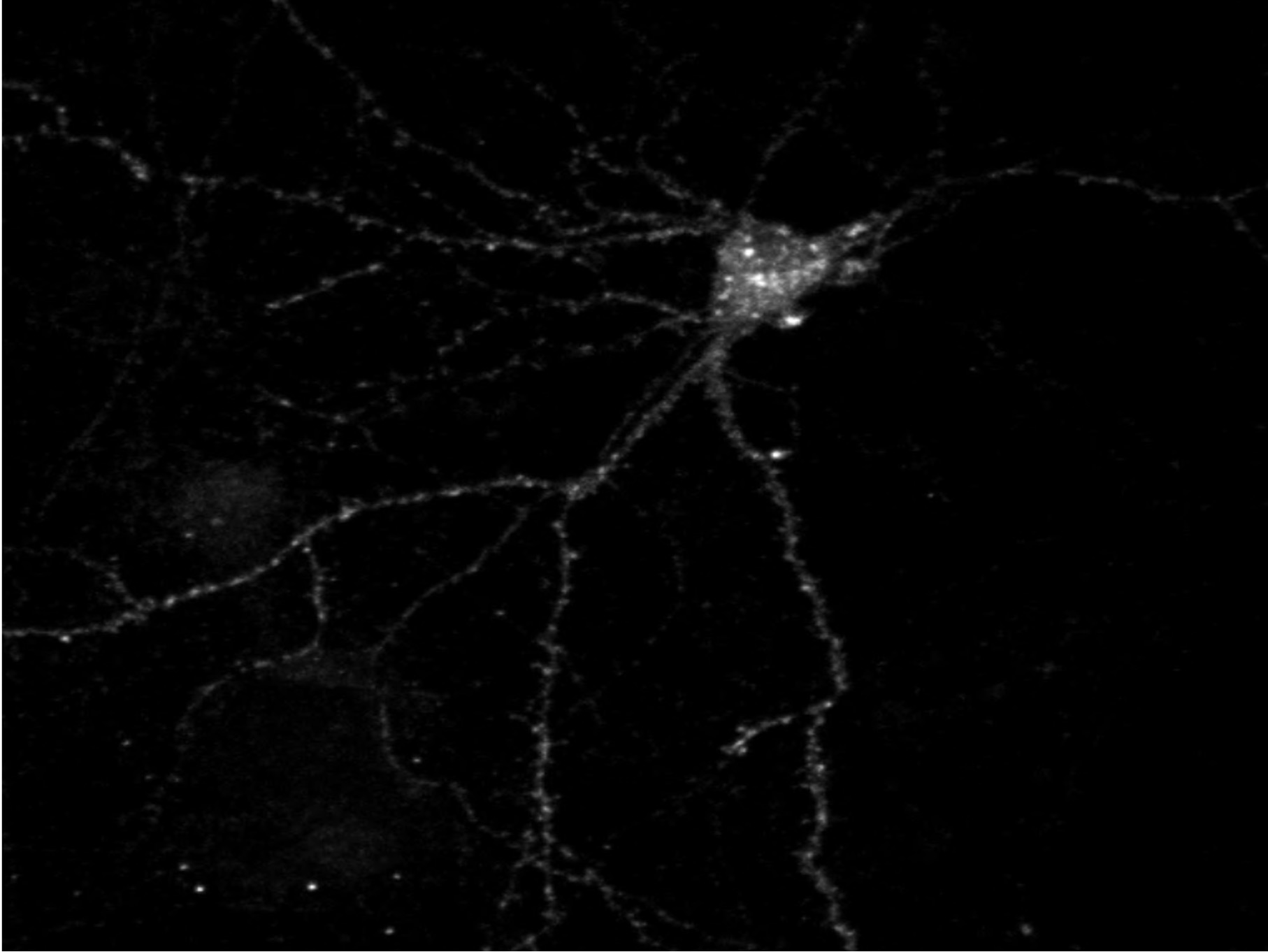

Supplement: Supplementary file 4 — Source data Fig. 2 [file 44318_2025_560_MOESM4_ESM.zip › Figure2/2I/Figure2I_NMDA_APV_DIV14_p-PXNS119.tif]

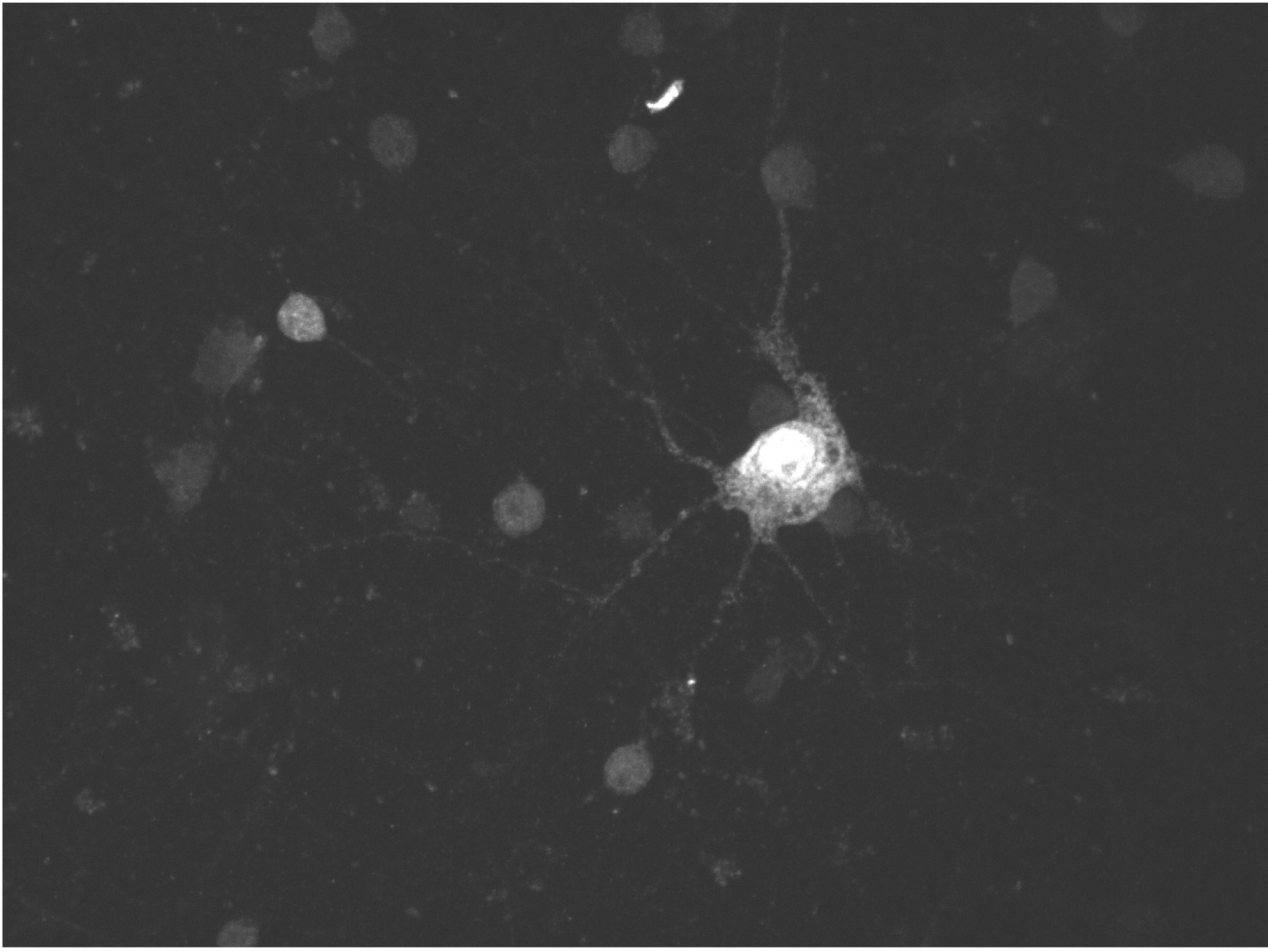

Supplement: Supplementary file 4 — Source data Fig. 2 [file 44318_2025_560_MOESM4_ESM.zip › Figure2/2I/Figure2I_NMDA_IPZ_DIV14_Tuj-1.tif]

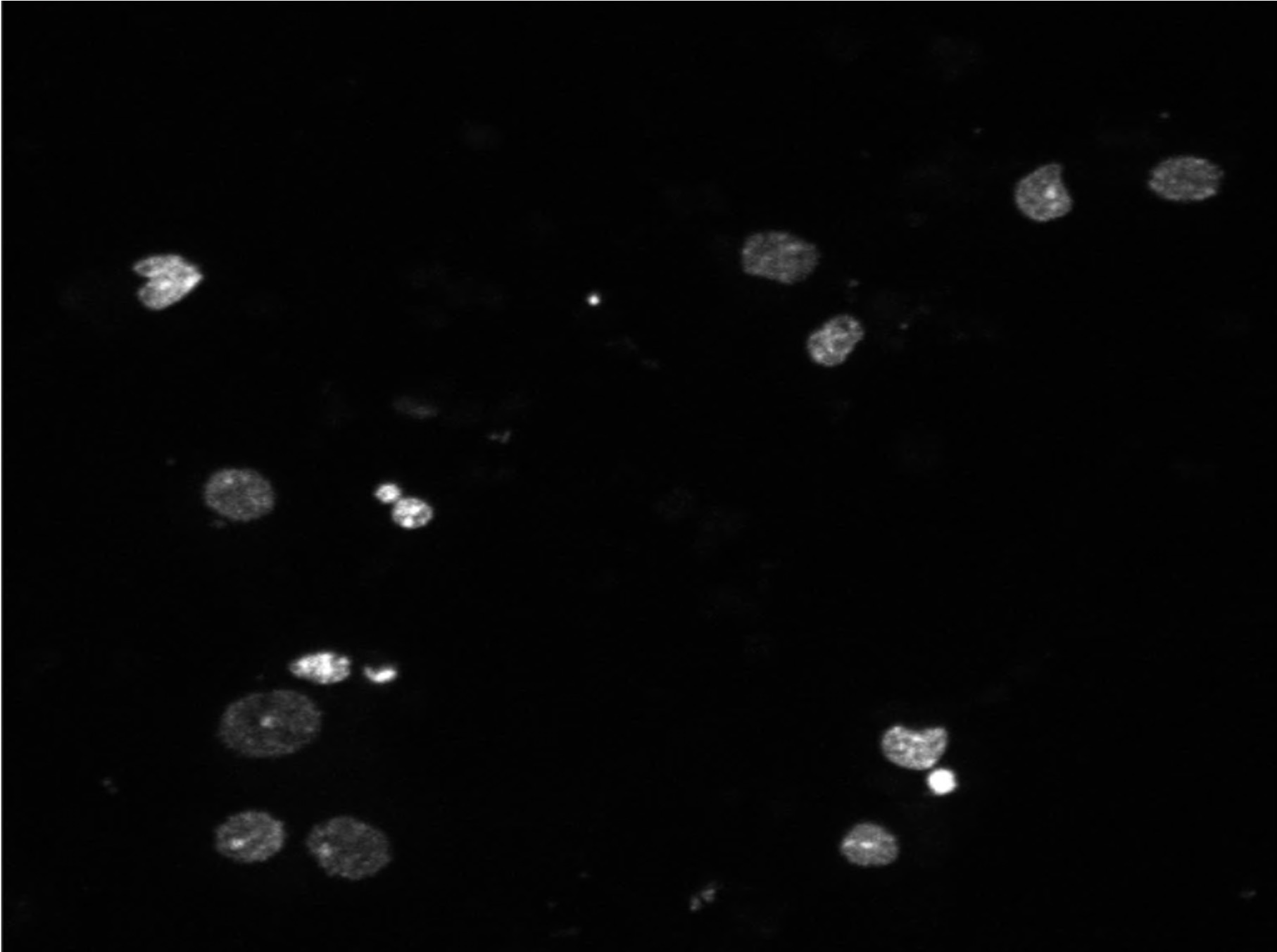

Supplement: Supplementary file 4 — Source data Fig. 2 [file 44318_2025_560_MOESM4_ESM.zip › Figure2/2I/Figure2I_NMDA_APV_DIV14_DAPI.tif]

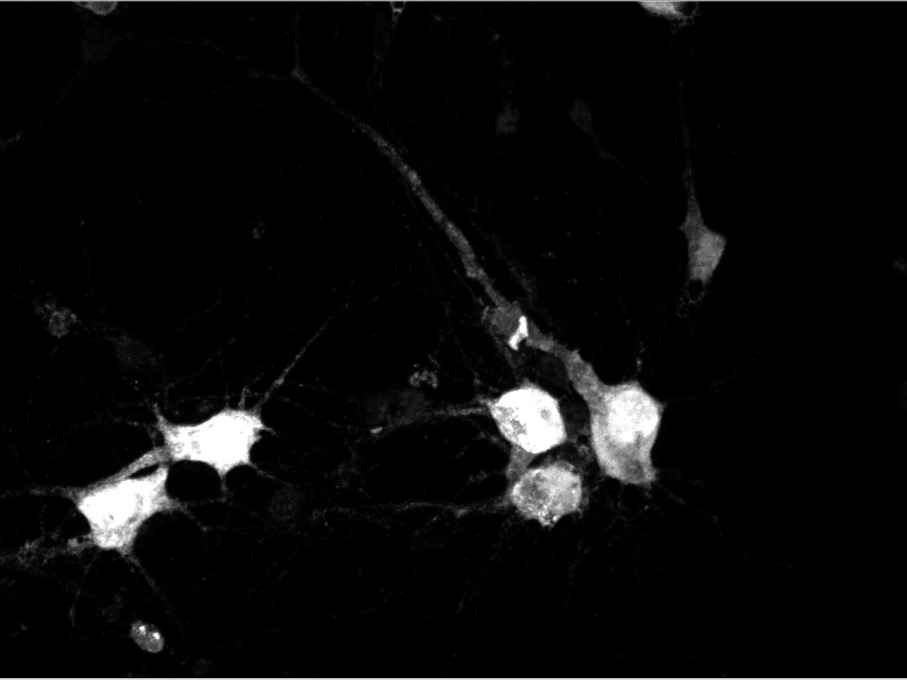

Supplement: Supplementary file 4 — Source data Fig. 2 [file 44318_2025_560_MOESM4_ESM.zip › Figure2/2I/Figure2I_NMDA_PurvalA_DIV14_Tuj-1.tif]

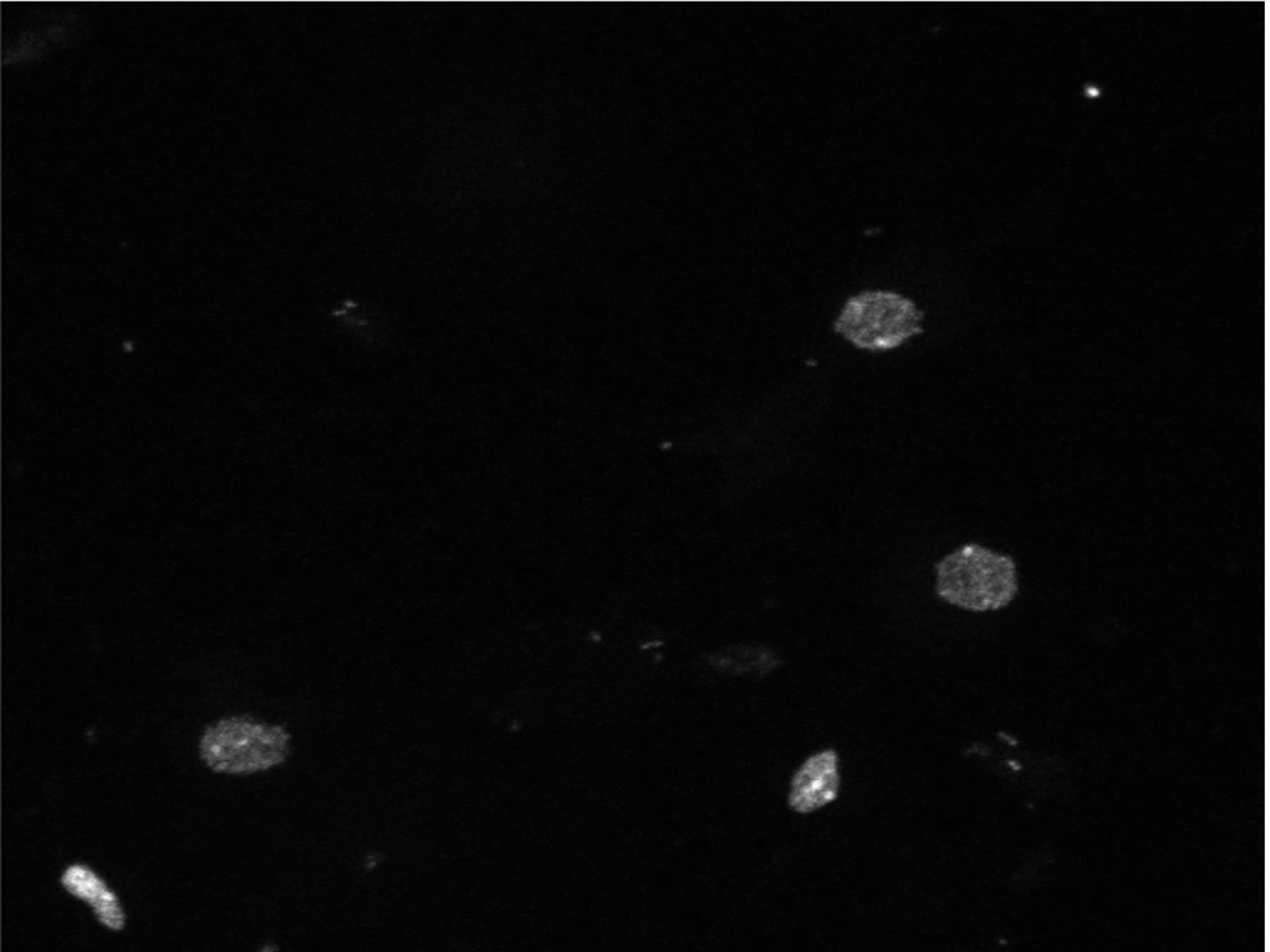

Supplement: Supplementary file 4 — Source data Fig. 2 [file 44318_2025_560_MOESM4_ESM.zip › Figure2/2I/Figure2I_NMDA_KN62_DIV14_DAPI.tif]

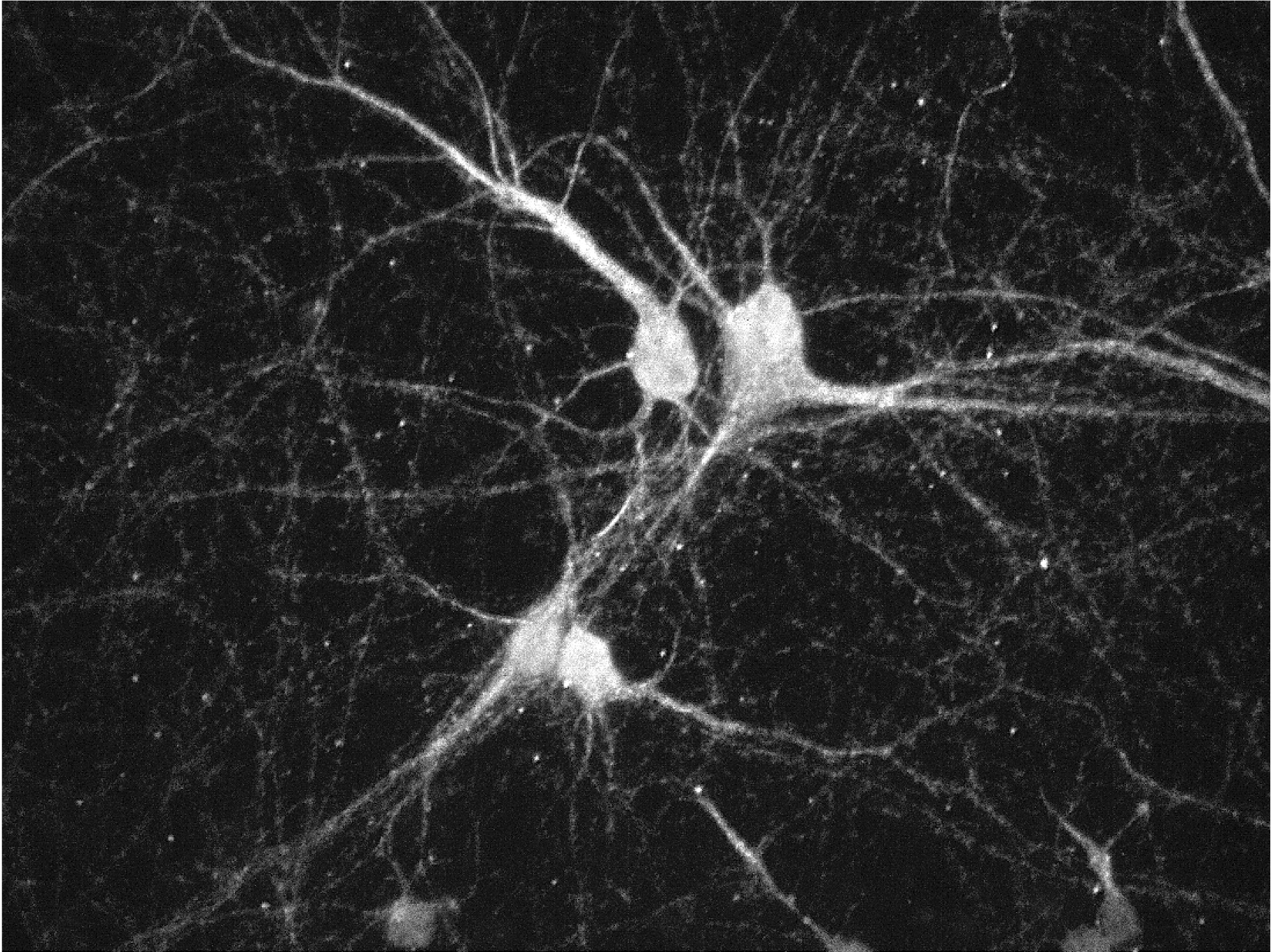

Supplement: Supplementary file 4 — Source data Fig. 2 [file 44318_2025_560_MOESM4_ESM.zip › Figure2/2I/Figure2I_Regular medium_DIV14_Tuj-1.tif]

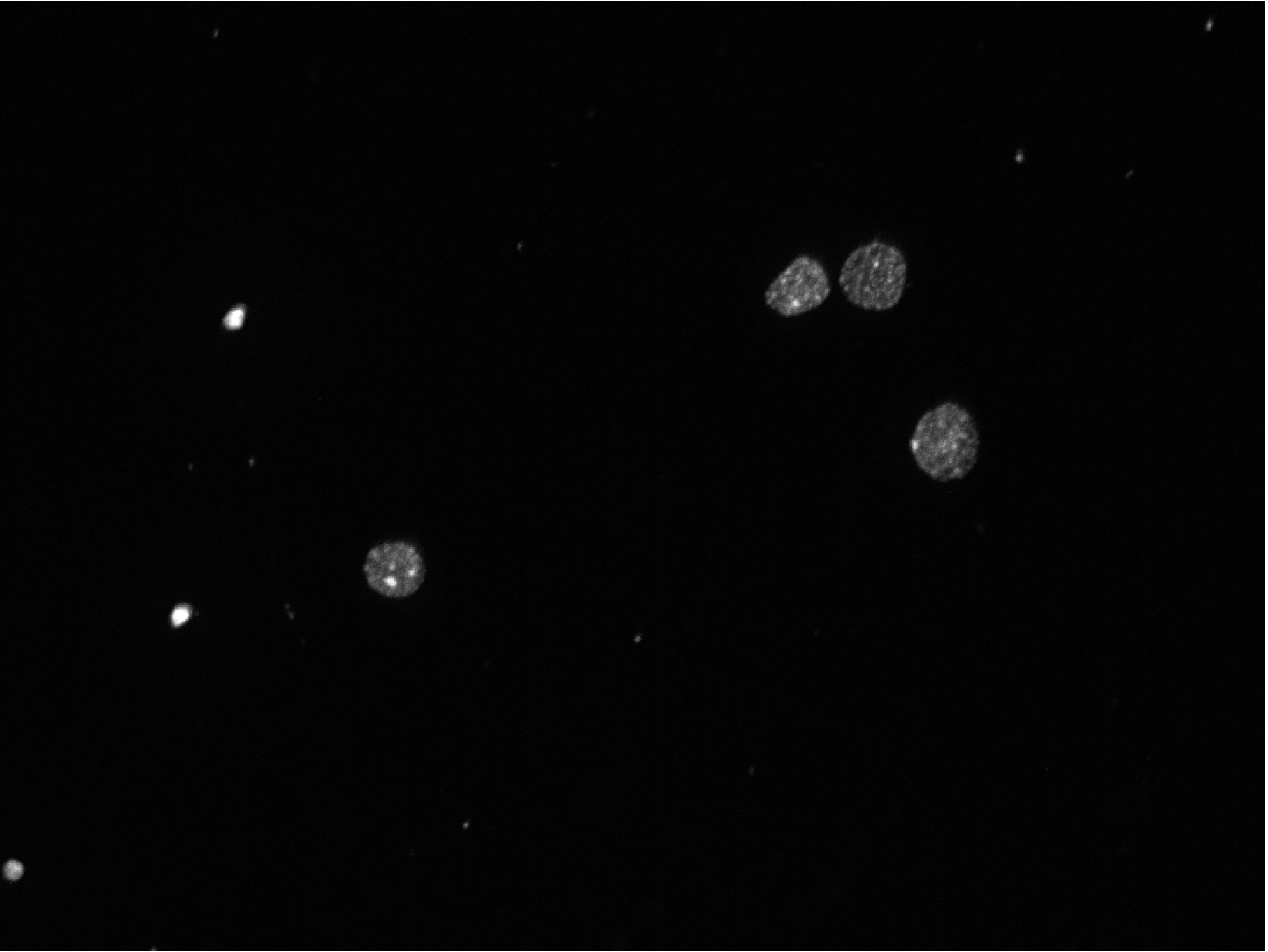

Supplement: Supplementary file 4 — Source data Fig. 2 [file 44318_2025_560_MOESM4_ESM.zip › Figure2/2I/Figure2I_NMDA_DIV14_DAPI.tif]

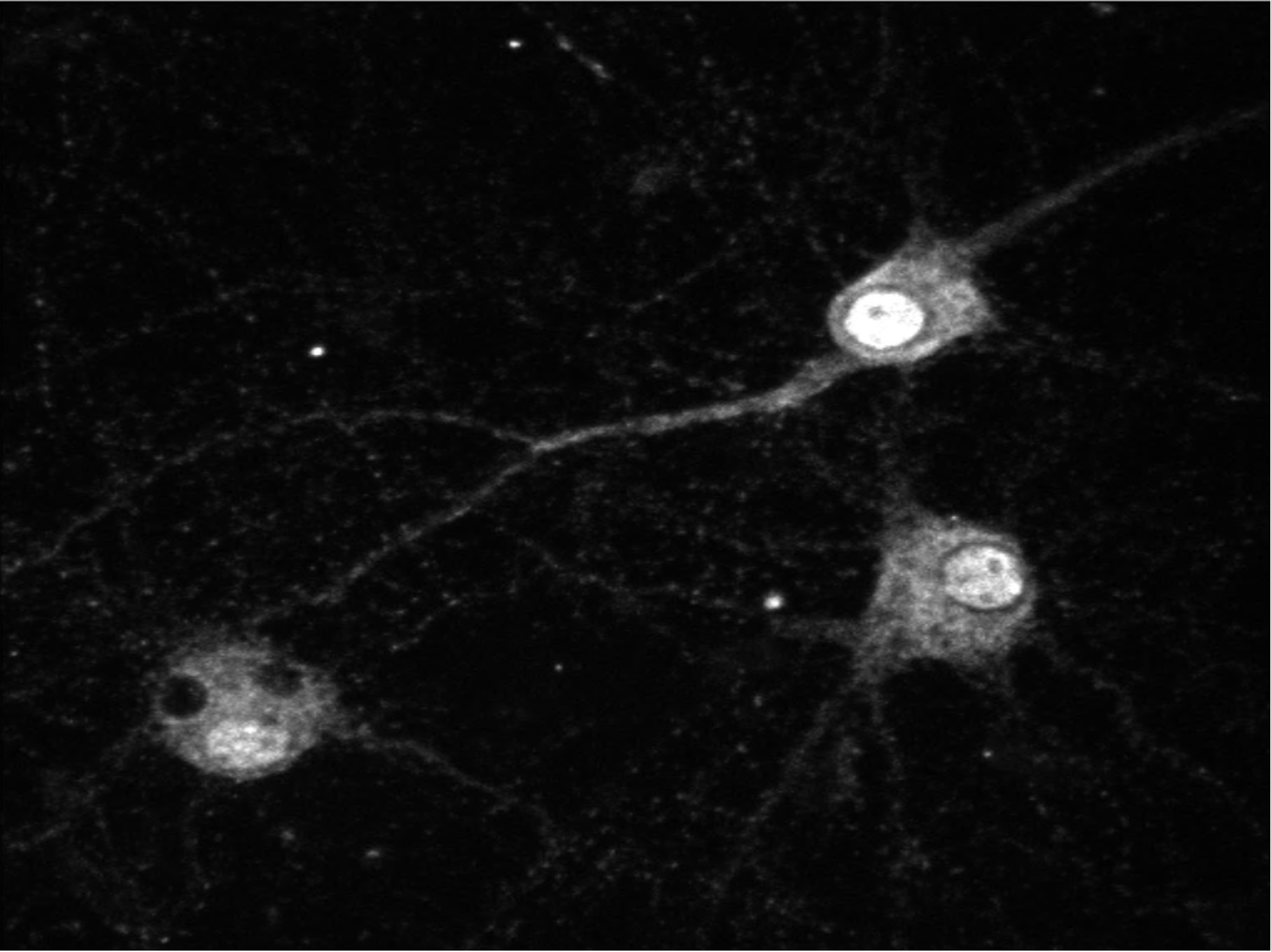

Supplement: Supplementary file 4 — Source data Fig. 2 [file 44318_2025_560_MOESM4_ESM.zip › Figure2/2I/Figure2I_NMDA_KN62_DIV14_Tuj-1.tif]

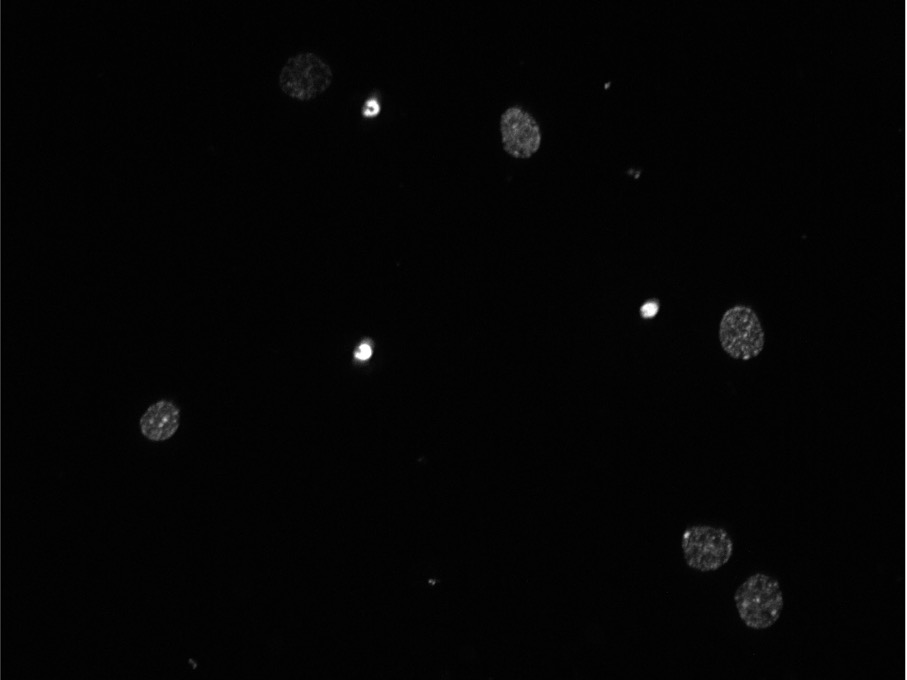

Supplement: Supplementary file 4 — Source data Fig. 2 [file 44318_2025_560_MOESM4_ESM.zip › Figure2/2I/Figure2I_NMDA_KT5720_DIV14_DAPI.tif]

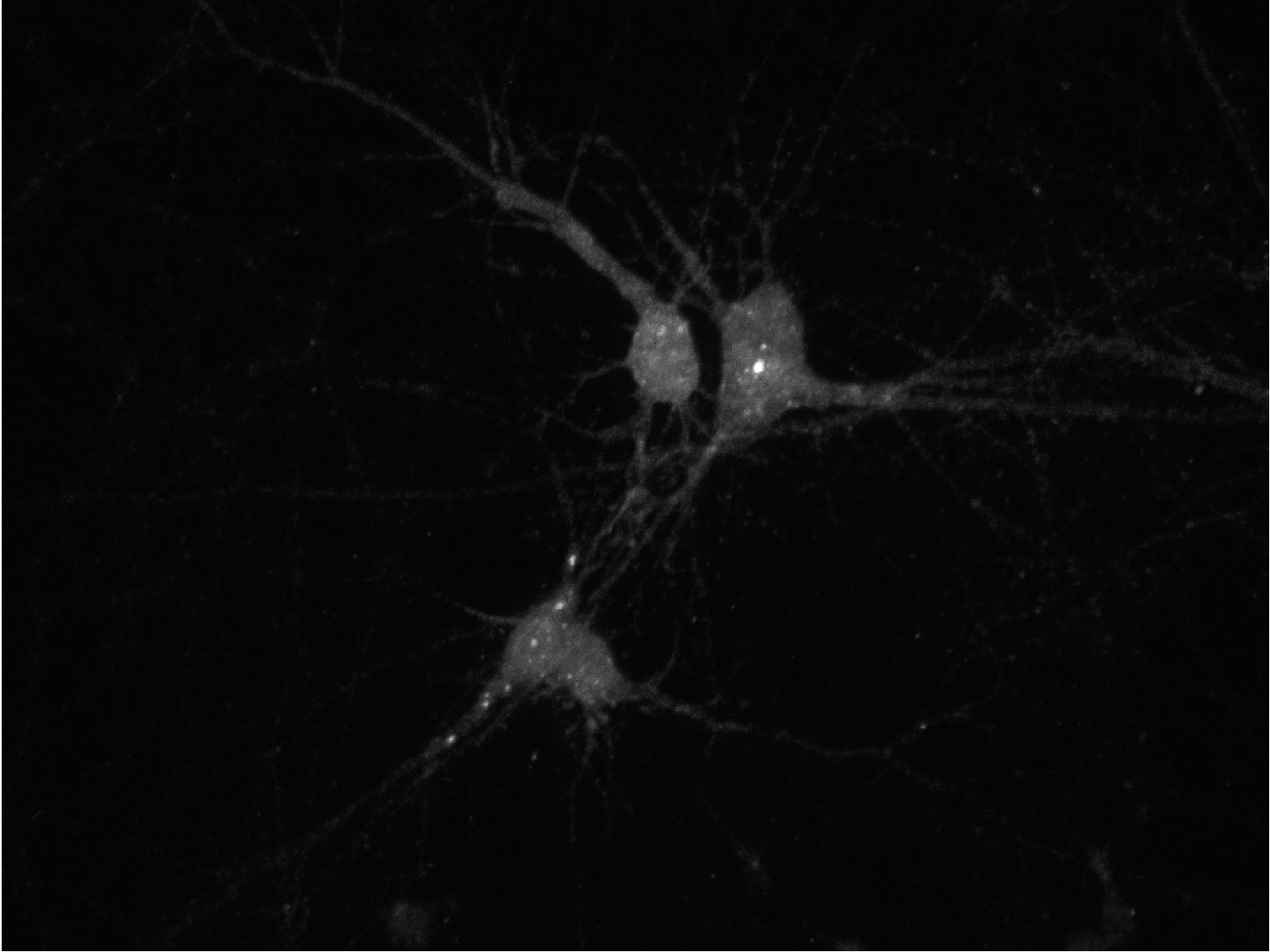

Supplement: Supplementary file 4 — Source data Fig. 2 [file 44318_2025_560_MOESM4_ESM.zip › Figure2/2I/Figure2I_Regular medium_DIV14_p-PXNS119.tif]

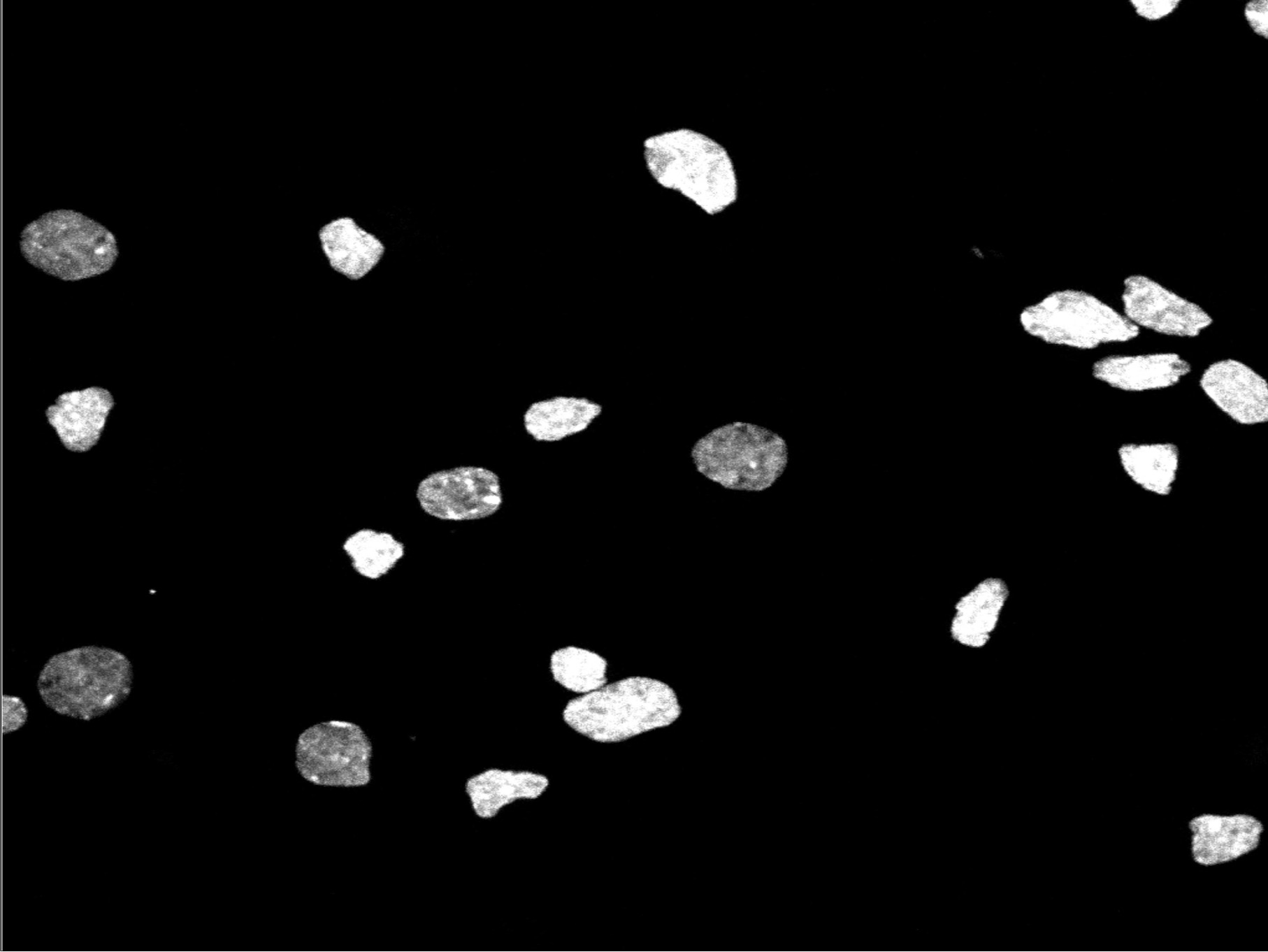

Supplement: Supplementary file 4 — Source data Fig. 2 [file 44318_2025_560_MOESM4_ESM.zip › Figure2/2I/Figure2I_Regular medium_DIV7_DAPI.tif]

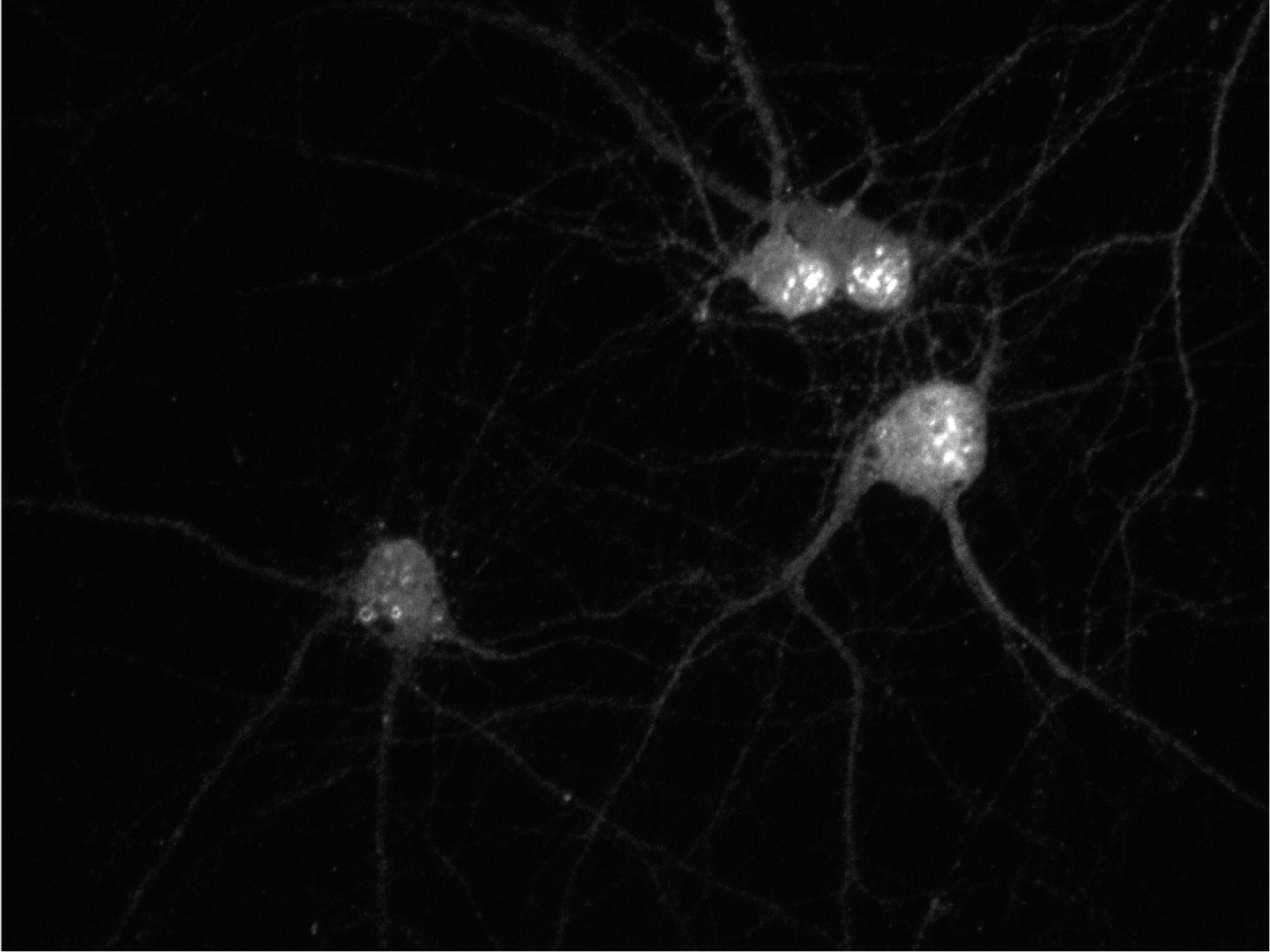

Supplement: Supplementary file 4 — Source data Fig. 2 [file 44318_2025_560_MOESM4_ESM.zip › Figure2/2I/Figure2I_NMDA_DIV14_p-PXNS119.tif]

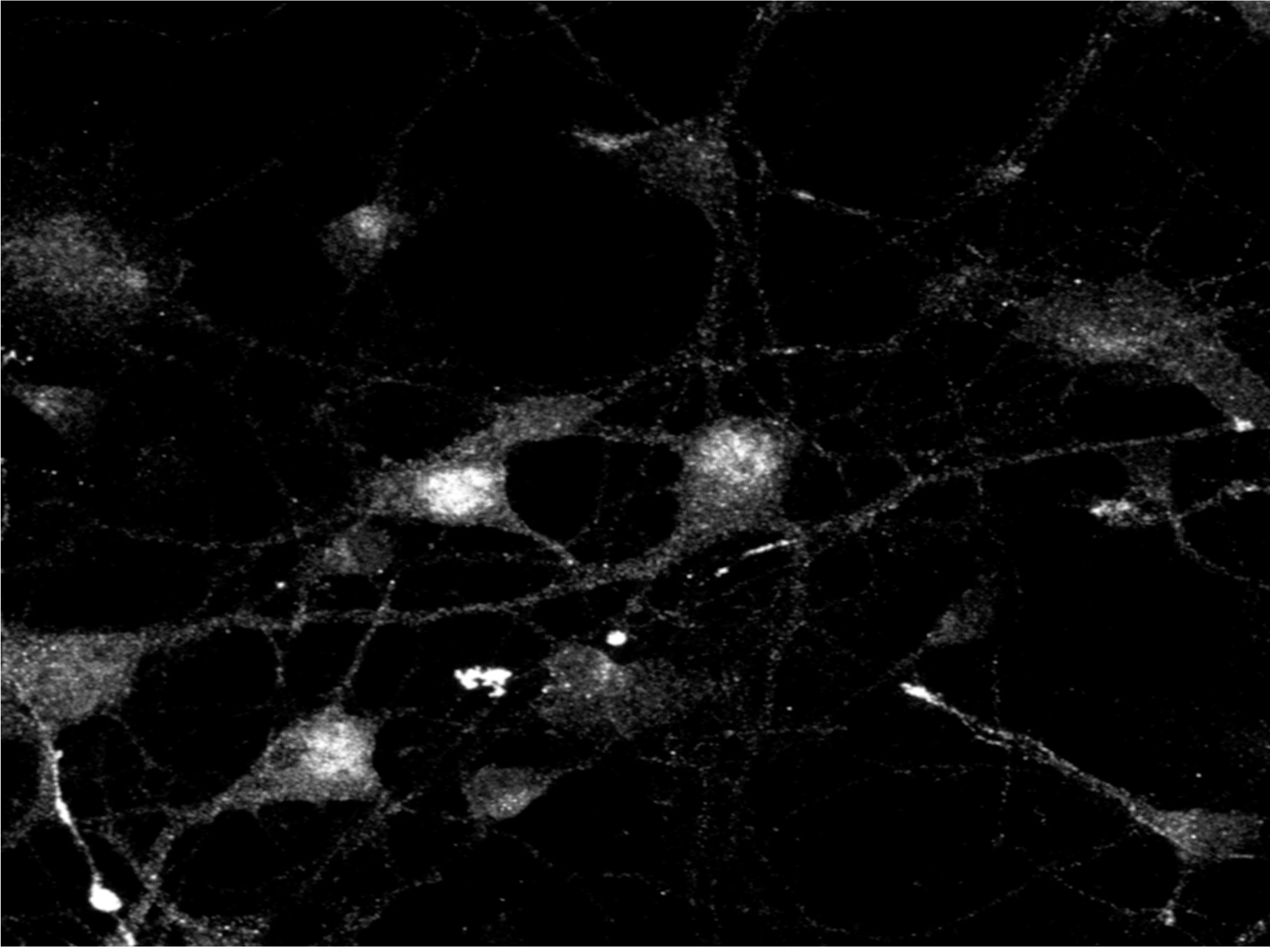

Supplement: Supplementary file 4 — Source data Fig. 2 [file 44318_2025_560_MOESM4_ESM.zip › Figure2/2I/Figure2I_Regular medium_DIV7_p-PXNS119.tif]

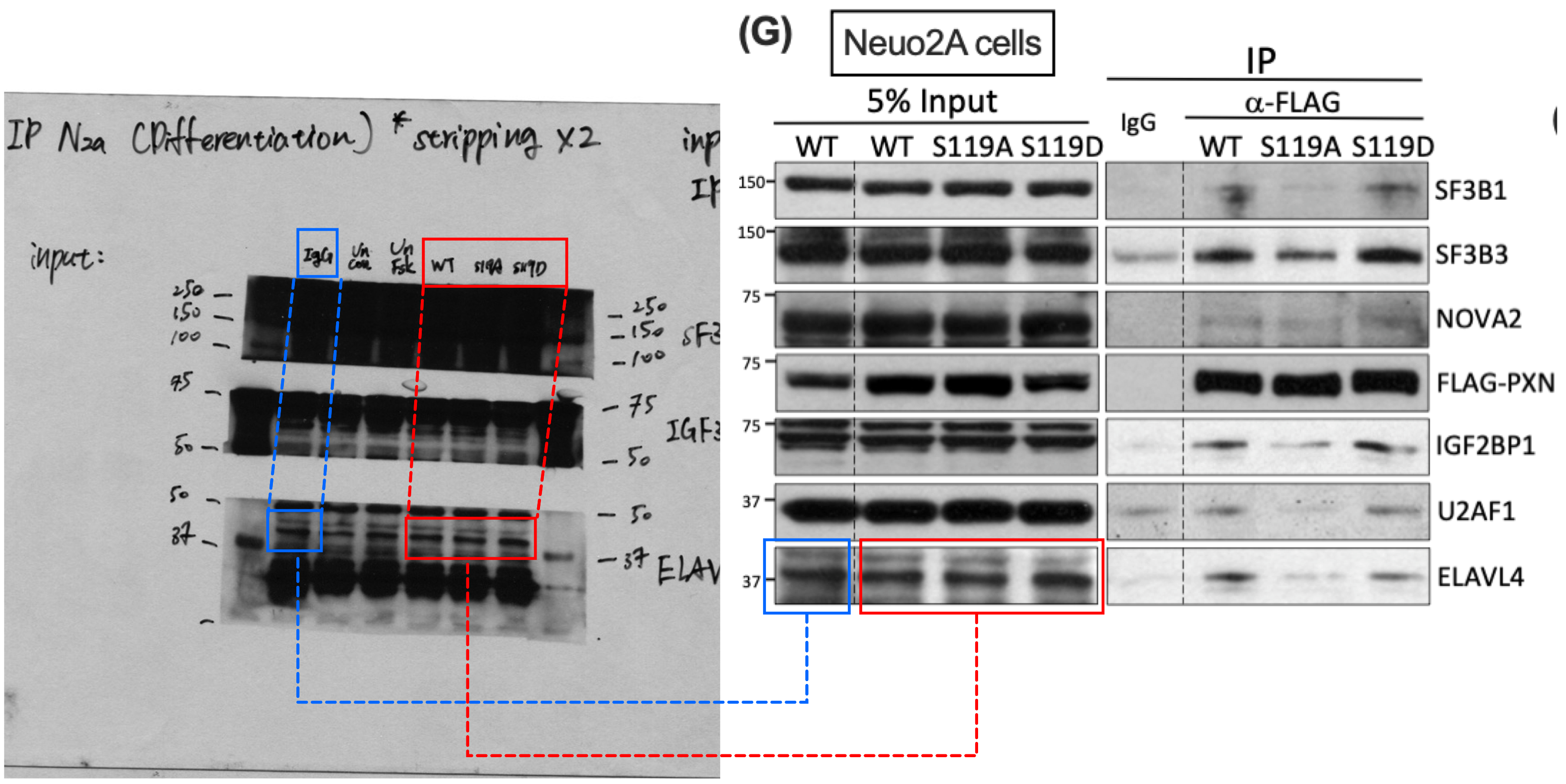

Supplement: Supplementary file 5 — Source data Fig. 4 [file 44318_2025_560_MOESM5_ESM.zip › Figure4/source data_Figure 4G (ELAV4_input)_final.tiff]

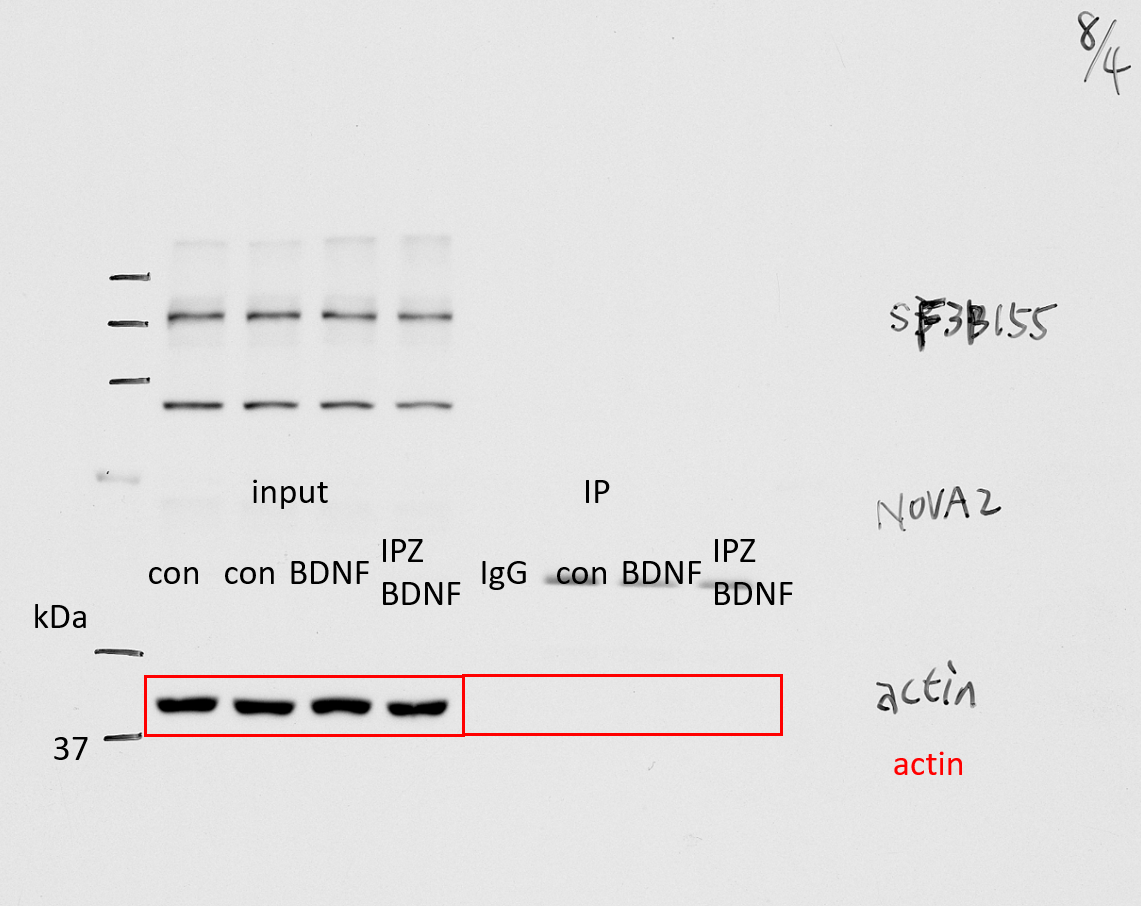

Supplement: Supplementary file 5 — Source data Fig. 4 [file 44318_2025_560_MOESM5_ESM.zip › Figure4/4C/Primary neuron_DIV7_IP-PXN_western_actin_input,IP.tif]

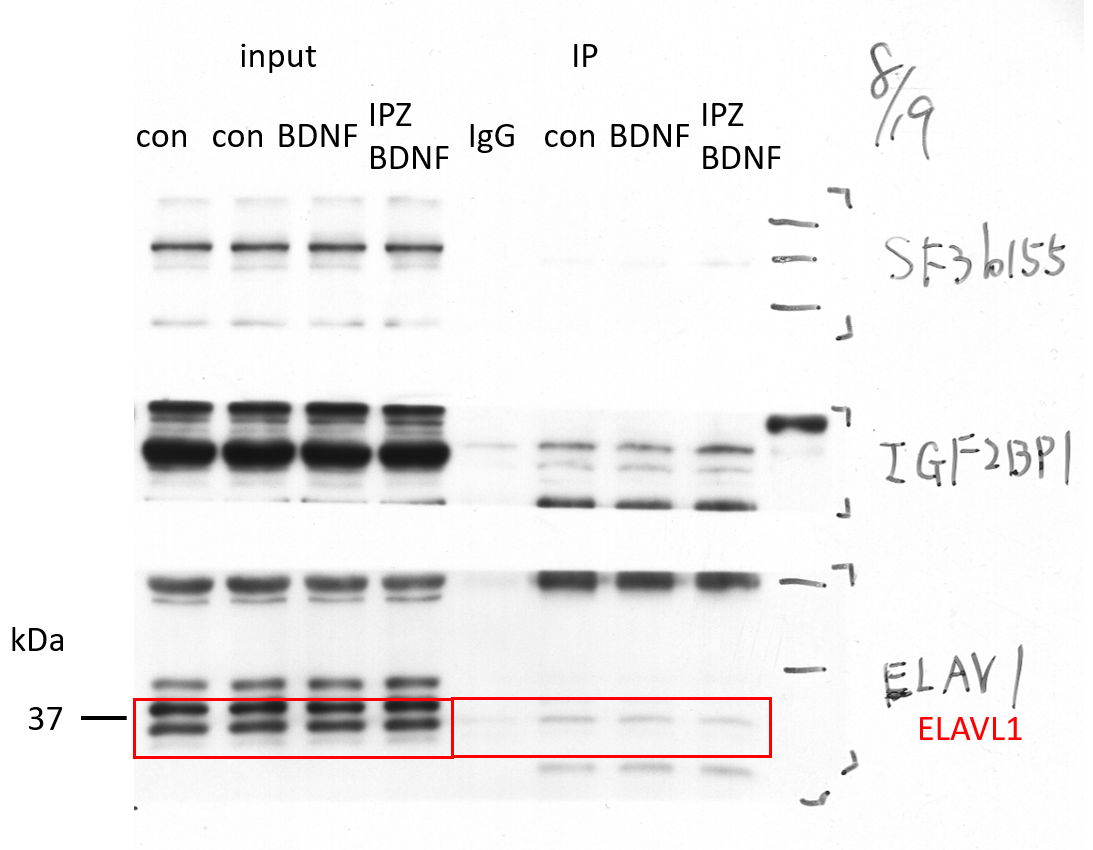

Supplement: Supplementary file 5 — Source data Fig. 4 [file 44318_2025_560_MOESM5_ESM.zip › Figure4/4C/Primary neuron_DIV7_IP-PXN_western_ELAVL1_input,IP.tif]

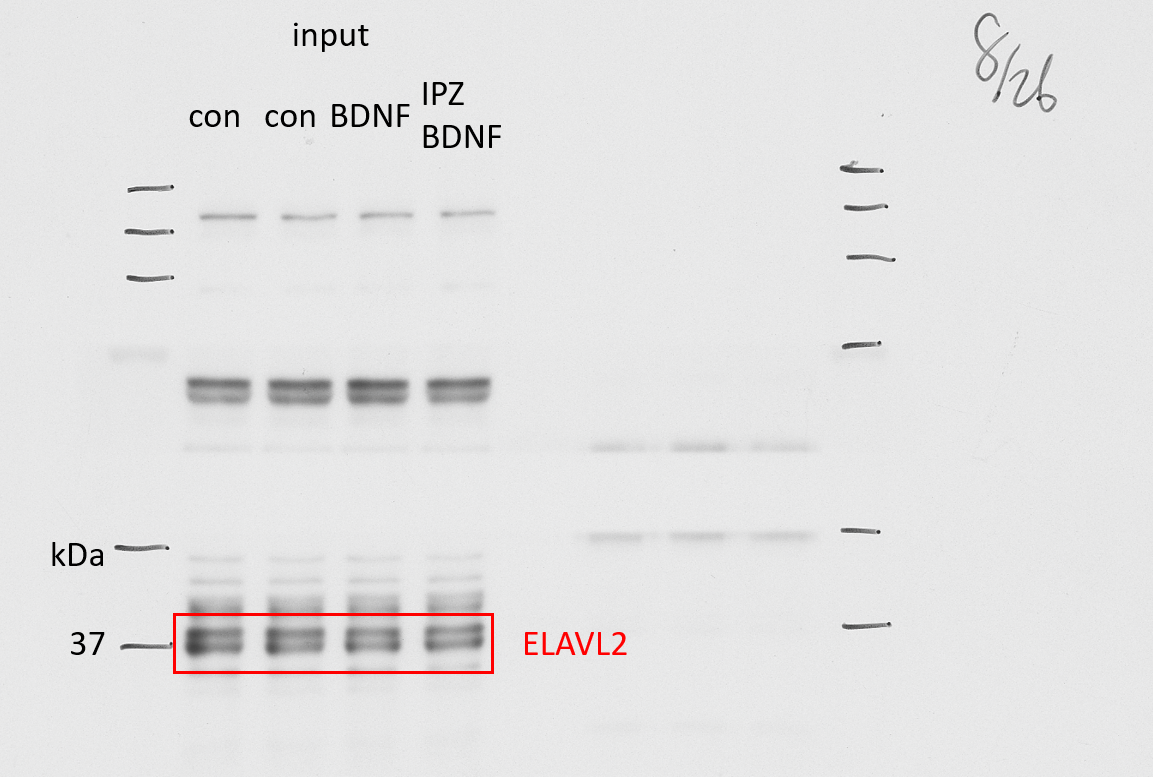

Supplement: Supplementary file 5 — Source data Fig. 4 [file 44318_2025_560_MOESM5_ESM.zip › Figure4/4C/Primary neuron_DIV7_IP-PXN_western_ELAVL2_input.tif]

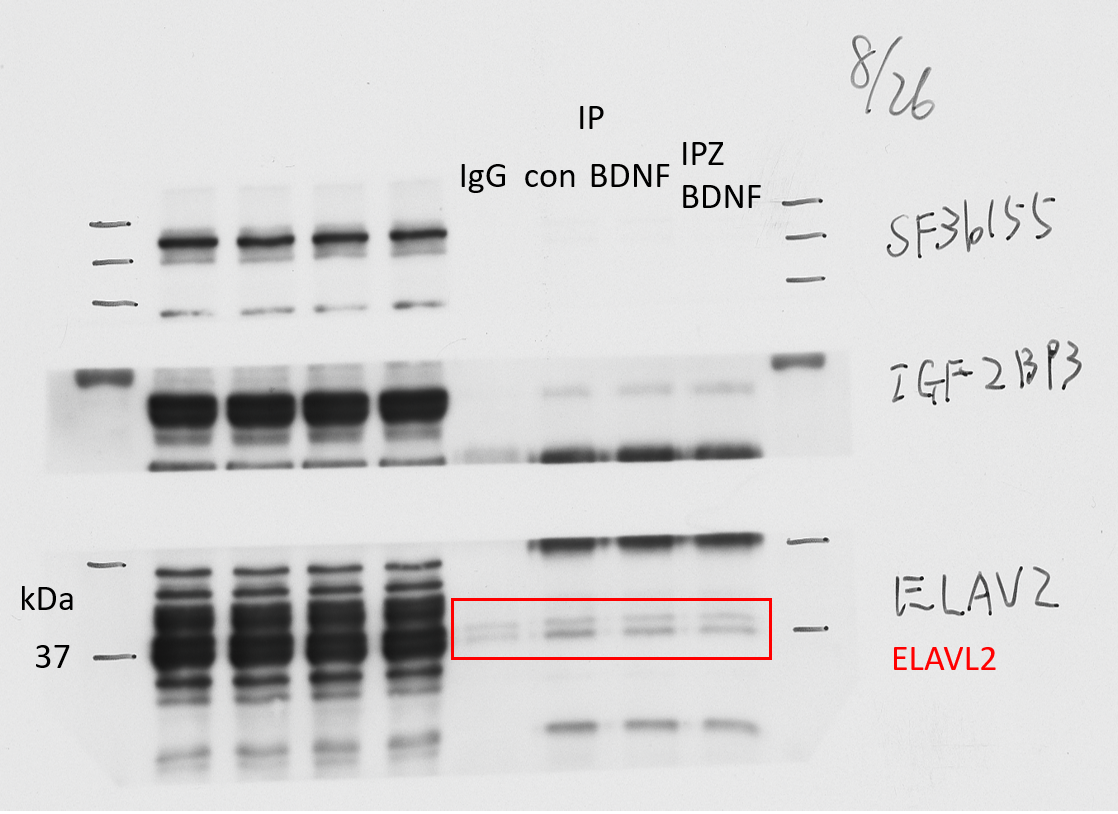

Supplement: Supplementary file 5 — Source data Fig. 4 [file 44318_2025_560_MOESM5_ESM.zip › Figure4/4C/Primary neuron_DIV7_IP-PXN_western_ELAVL2_IP.tif]

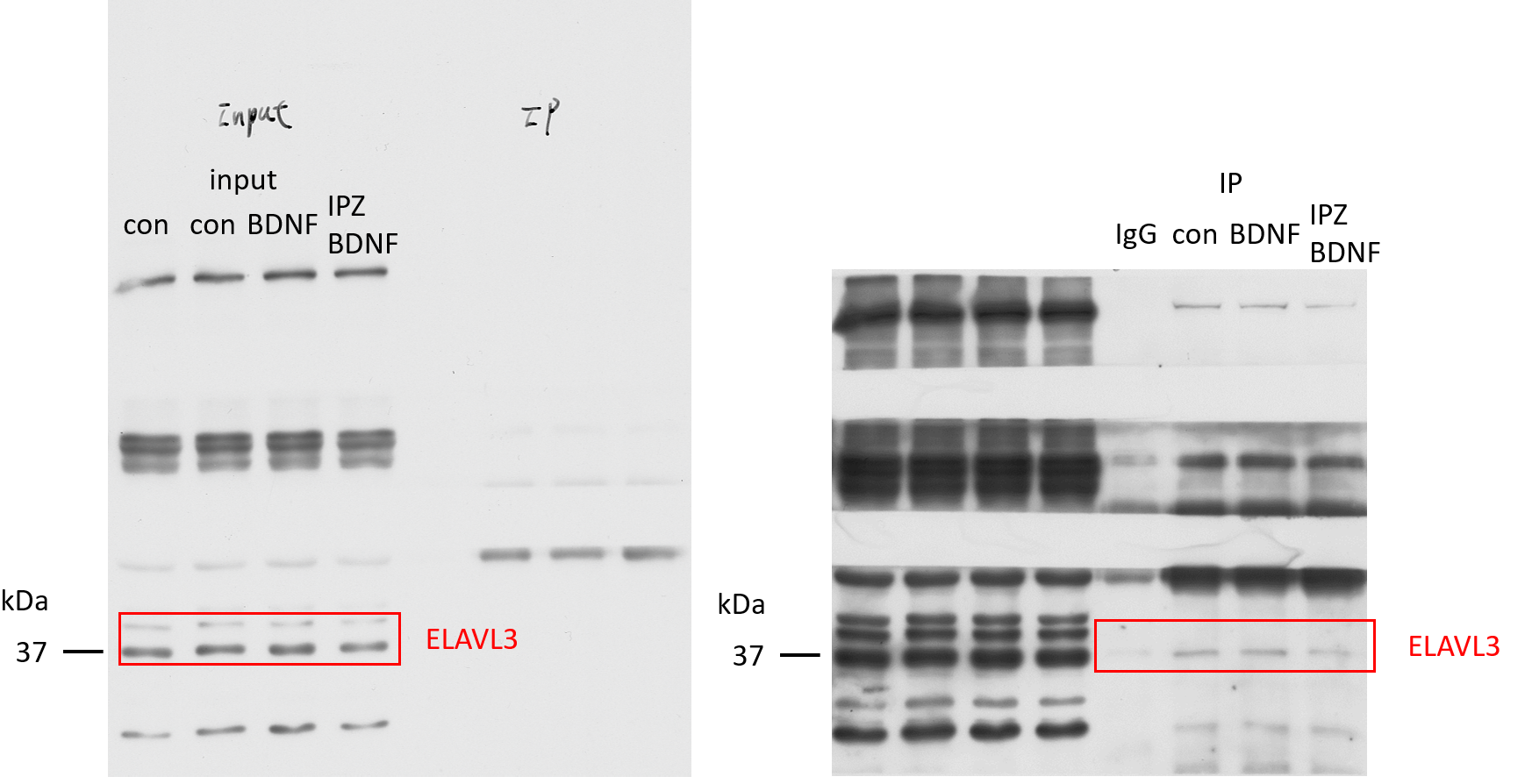

Supplement: Supplementary file 5 — Source data Fig. 4 [file 44318_2025_560_MOESM5_ESM.zip › Figure4/4C/Primary neuron_DIV7_IP-PXN_western_ELAVL3_input,IP.tif]

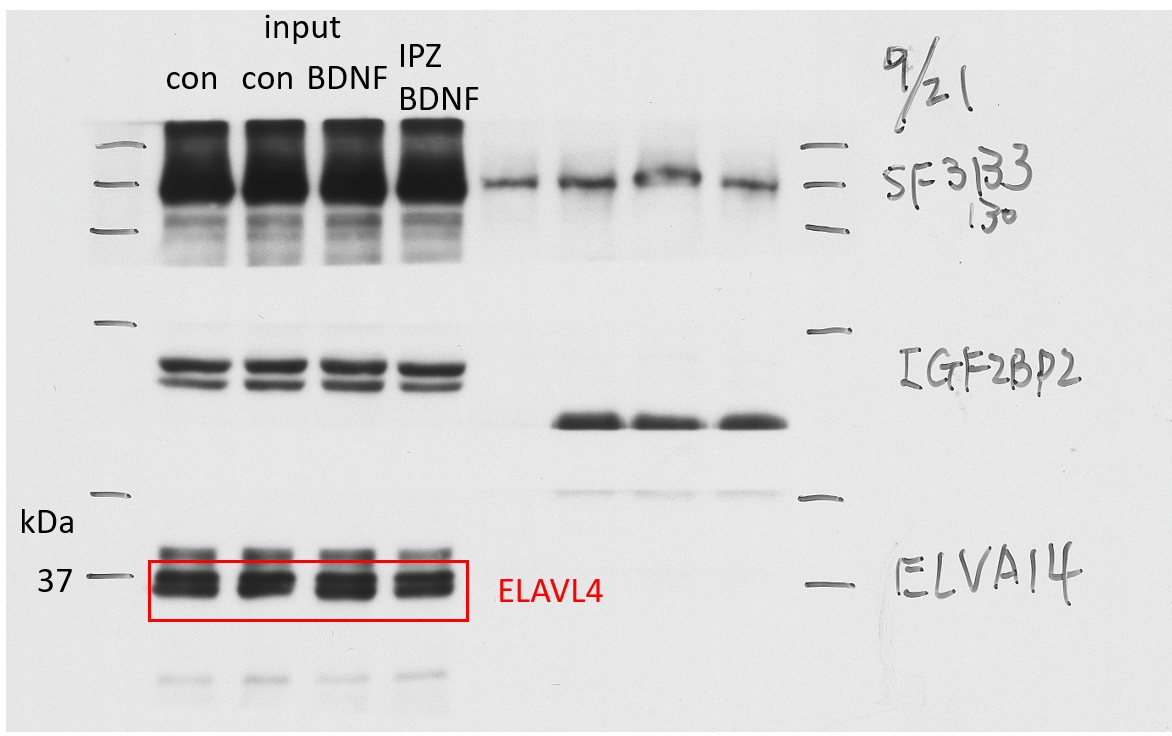

Supplement: Supplementary file 5 — Source data Fig. 4 [file 44318_2025_560_MOESM5_ESM.zip › Figure4/4C/Primary neuron_DIV7_IP-PXN_western_ELAVL4_input.tif]

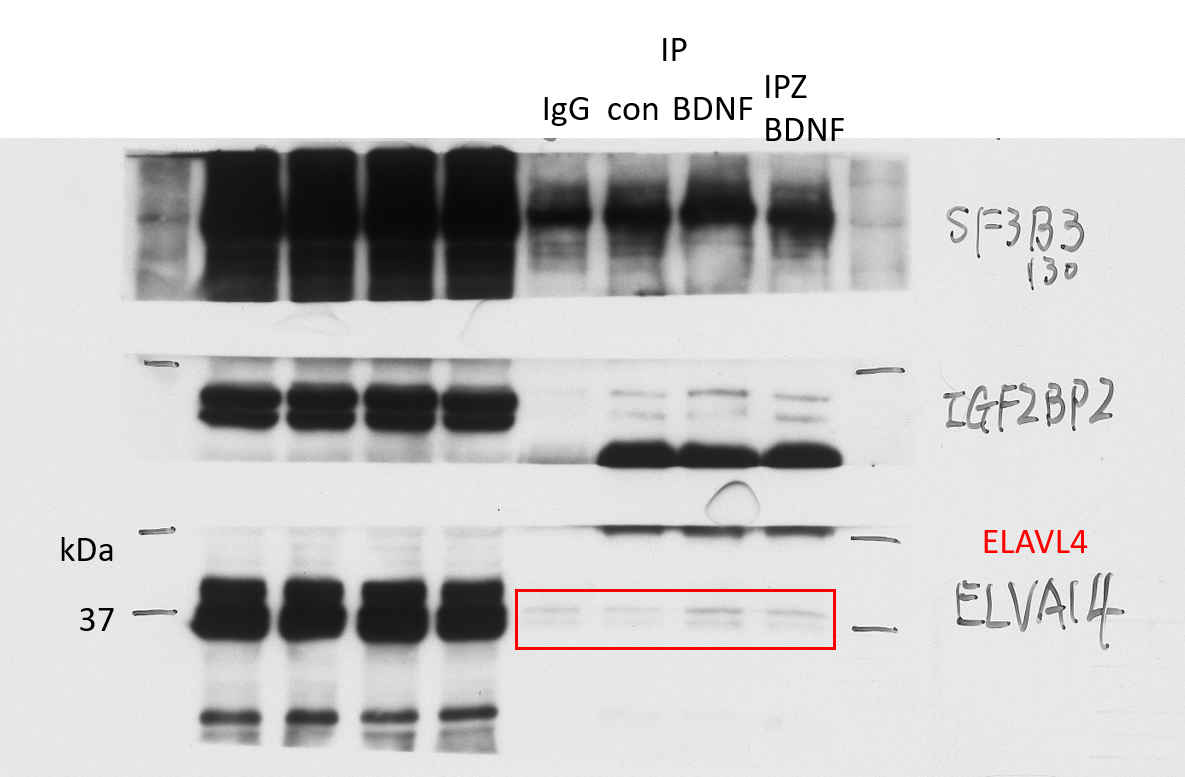

Supplement: Supplementary file 5 — Source data Fig. 4 [file 44318_2025_560_MOESM5_ESM.zip › Figure4/4C/Primary neuron_DIV7_IP-PXN_western_ELAVL4_IP.tif]

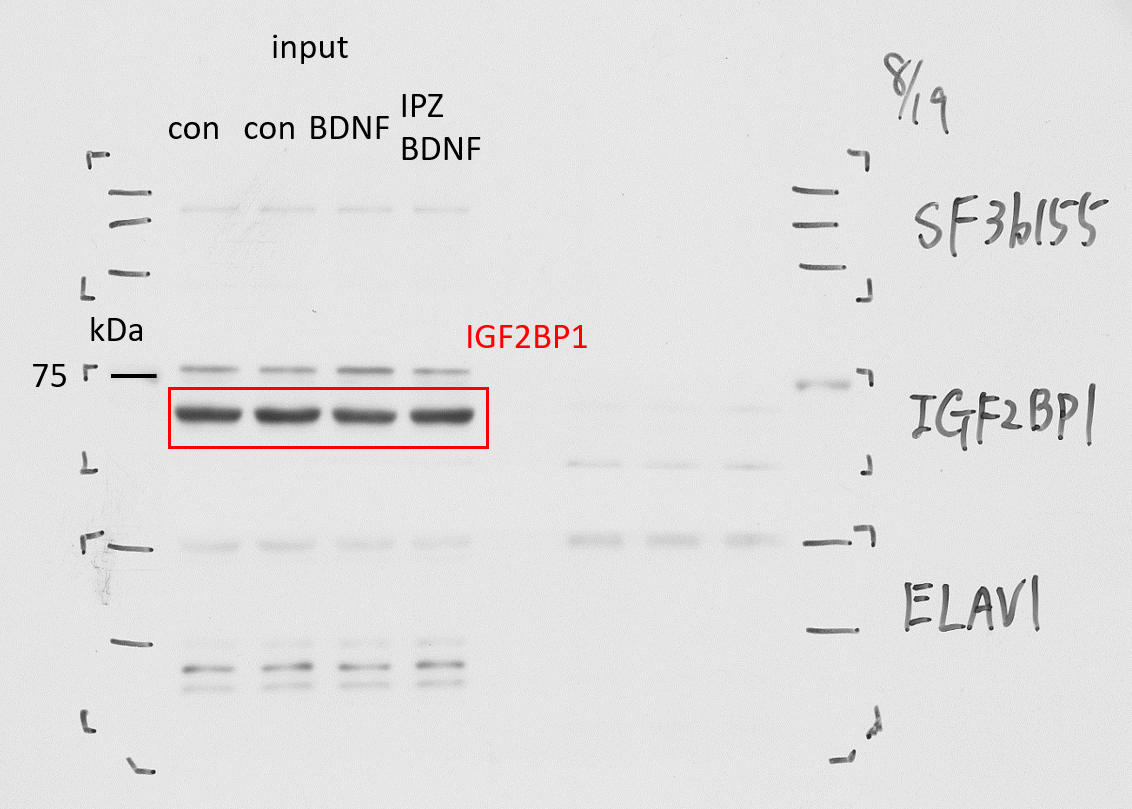

Supplement: Supplementary file 5 — Source data Fig. 4 [file 44318_2025_560_MOESM5_ESM.zip › Figure4/4C/Primary neuron_DIV7_IP-PXN_western_IGF2BP1_input.tif]

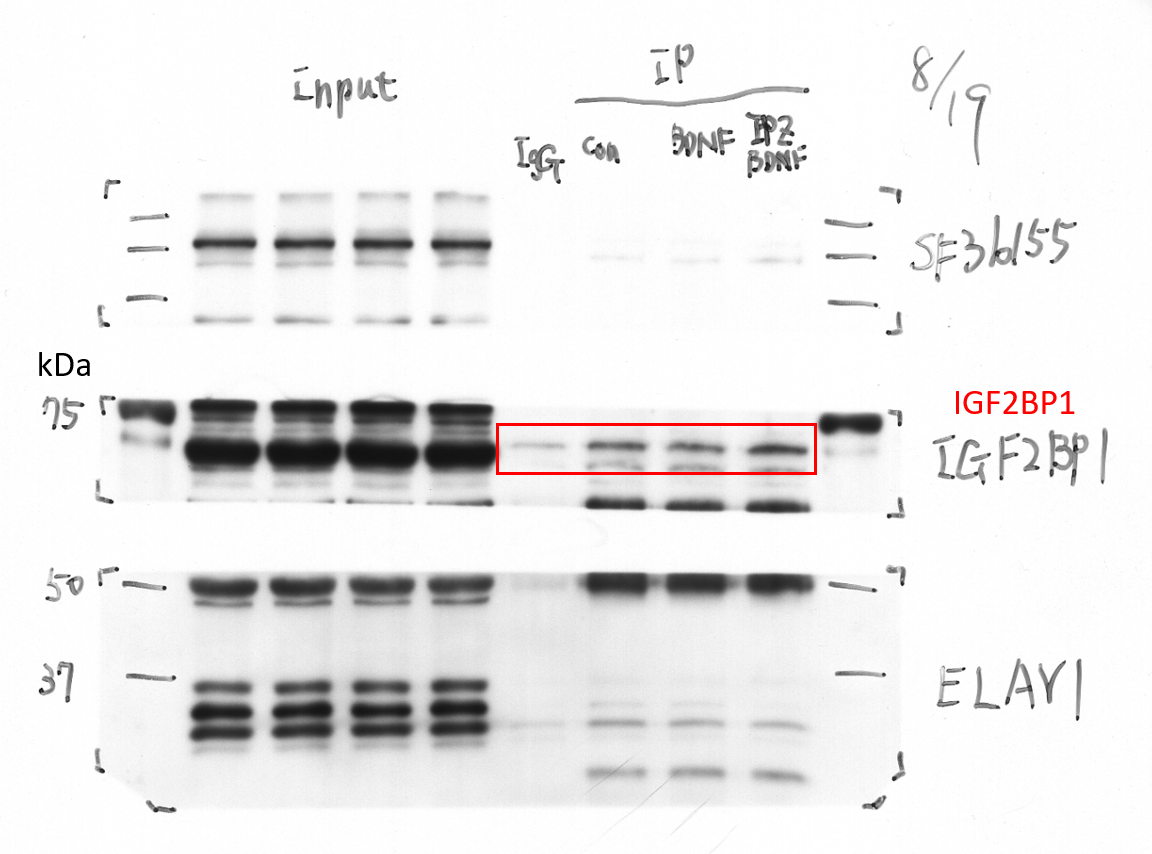

Supplement: Supplementary file 5 — Source data Fig. 4 [file 44318_2025_560_MOESM5_ESM.zip › Figure4/4C/Primary neuron_DIV7_IP-PXN_western_IGF2BP1_IP.tif]

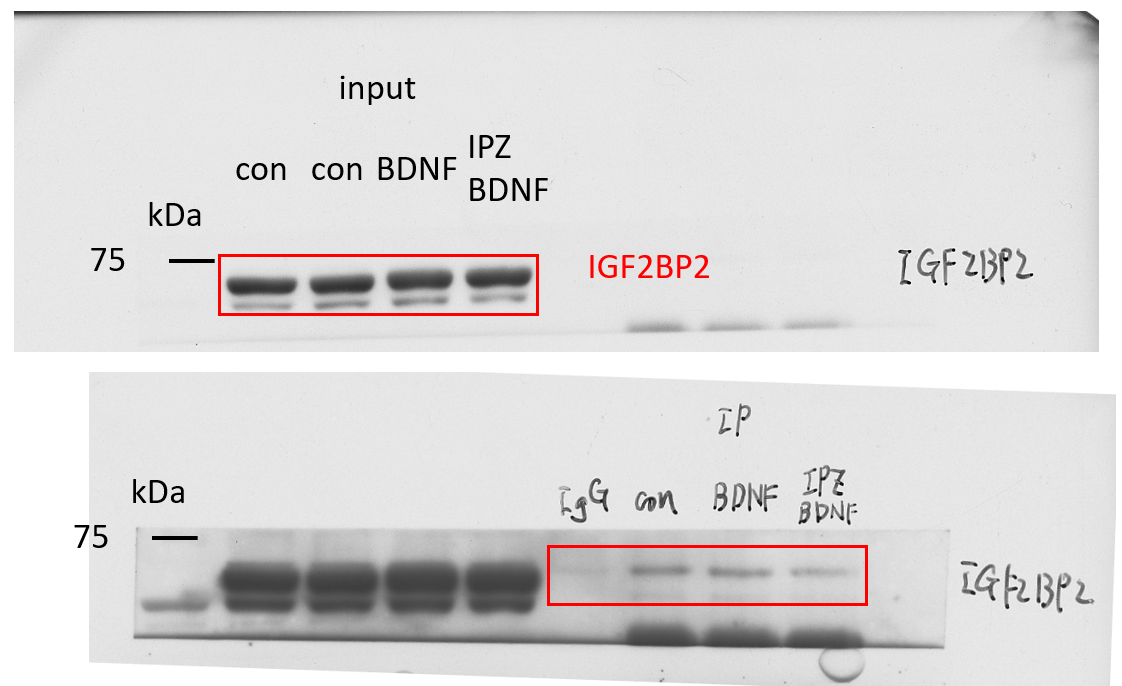

Supplement: Supplementary file 5 — Source data Fig. 4 [file 44318_2025_560_MOESM5_ESM.zip › Figure4/4C/Primary neuron_DIV7_IP-PXN_western_IGF2BP2_input,IP.tif]

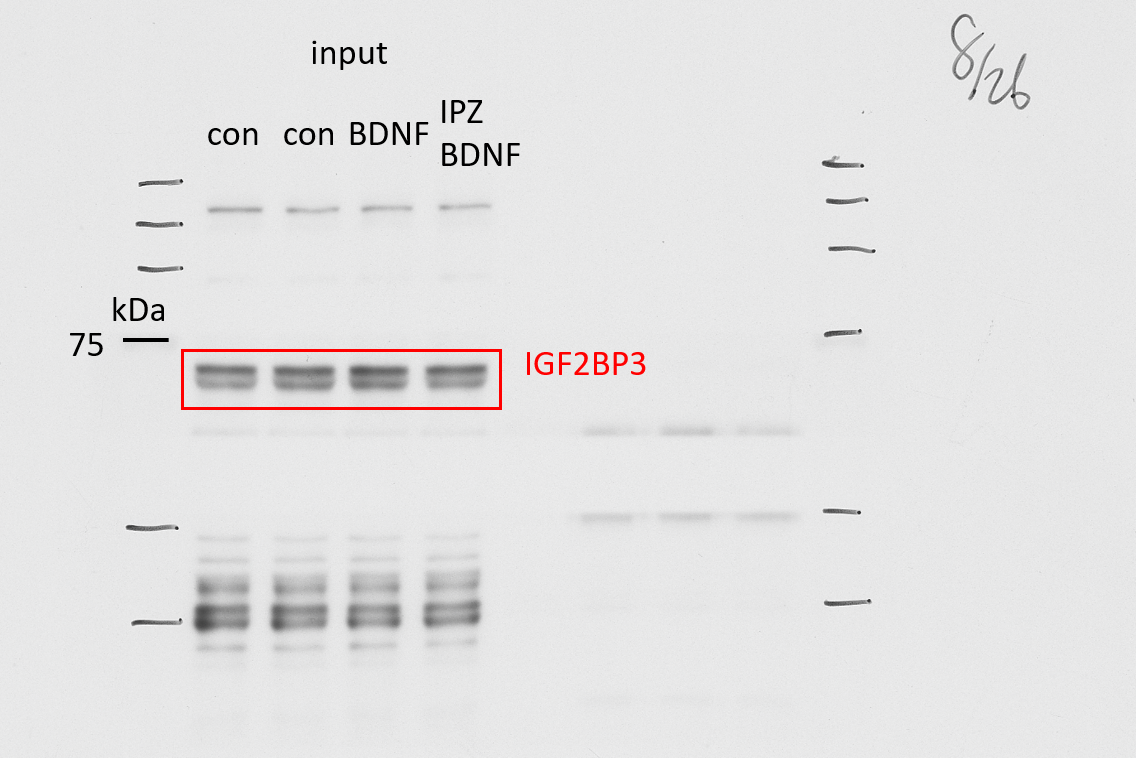

Supplement: Supplementary file 5 — Source data Fig. 4 [file 44318_2025_560_MOESM5_ESM.zip › Figure4/4C/Primary neuron_DIV7_IP-PXN_western_IGF2BP3_input.tif]

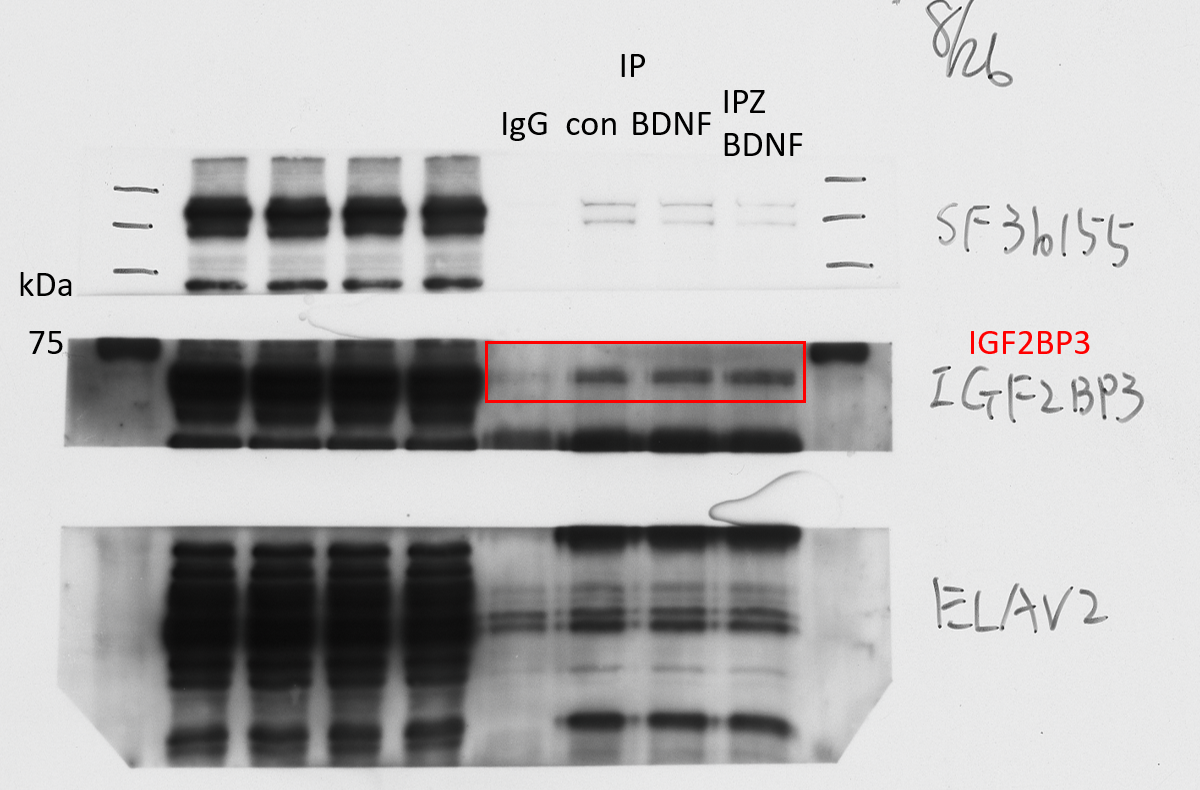

Supplement: Supplementary file 5 — Source data Fig. 4 [file 44318_2025_560_MOESM5_ESM.zip › Figure4/4C/Primary neuron_DIV7_IP-PXN_western_IGF2BP3_IP.tif]

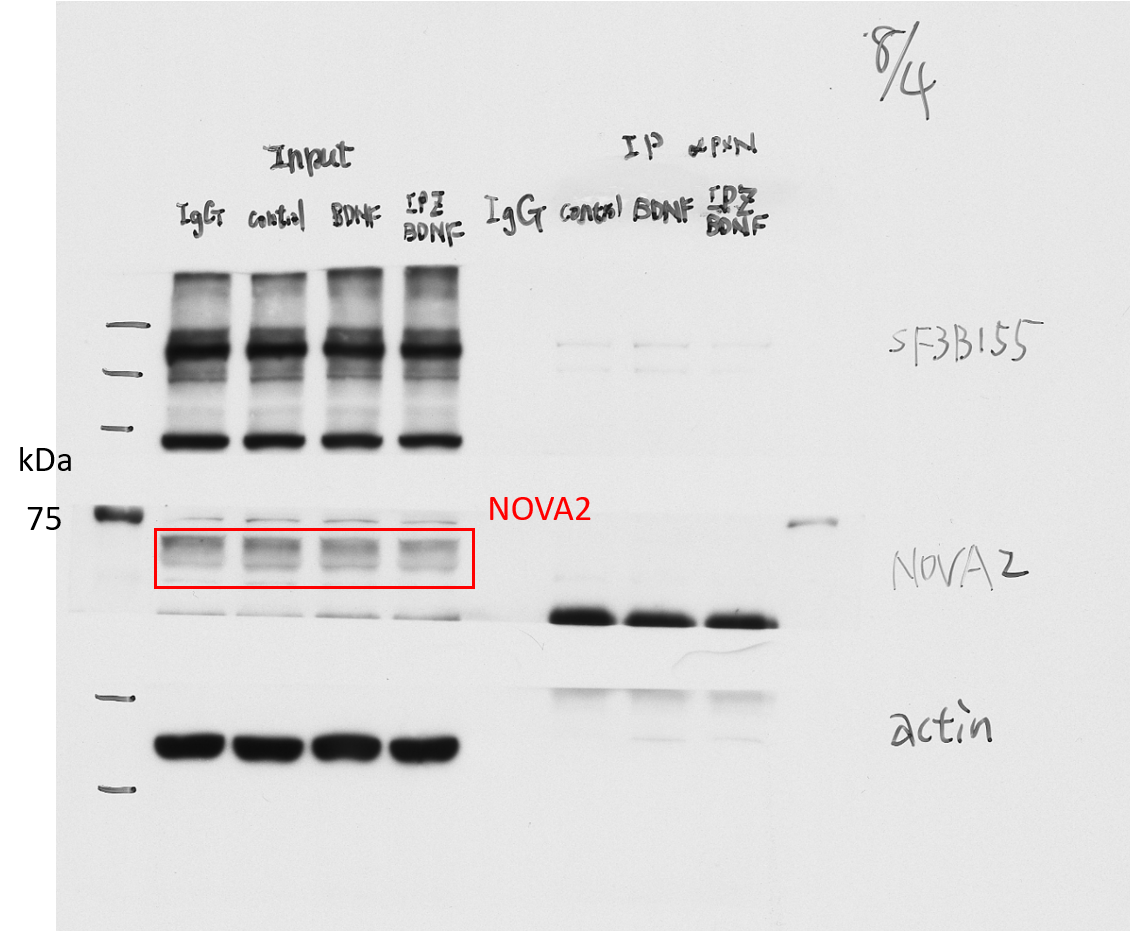

Supplement: Supplementary file 5 — Source data Fig. 4 [file 44318_2025_560_MOESM5_ESM.zip › Figure4/4C/Primary neuron_DIV7_IP-PXN_western_NOVA2_input.tif]

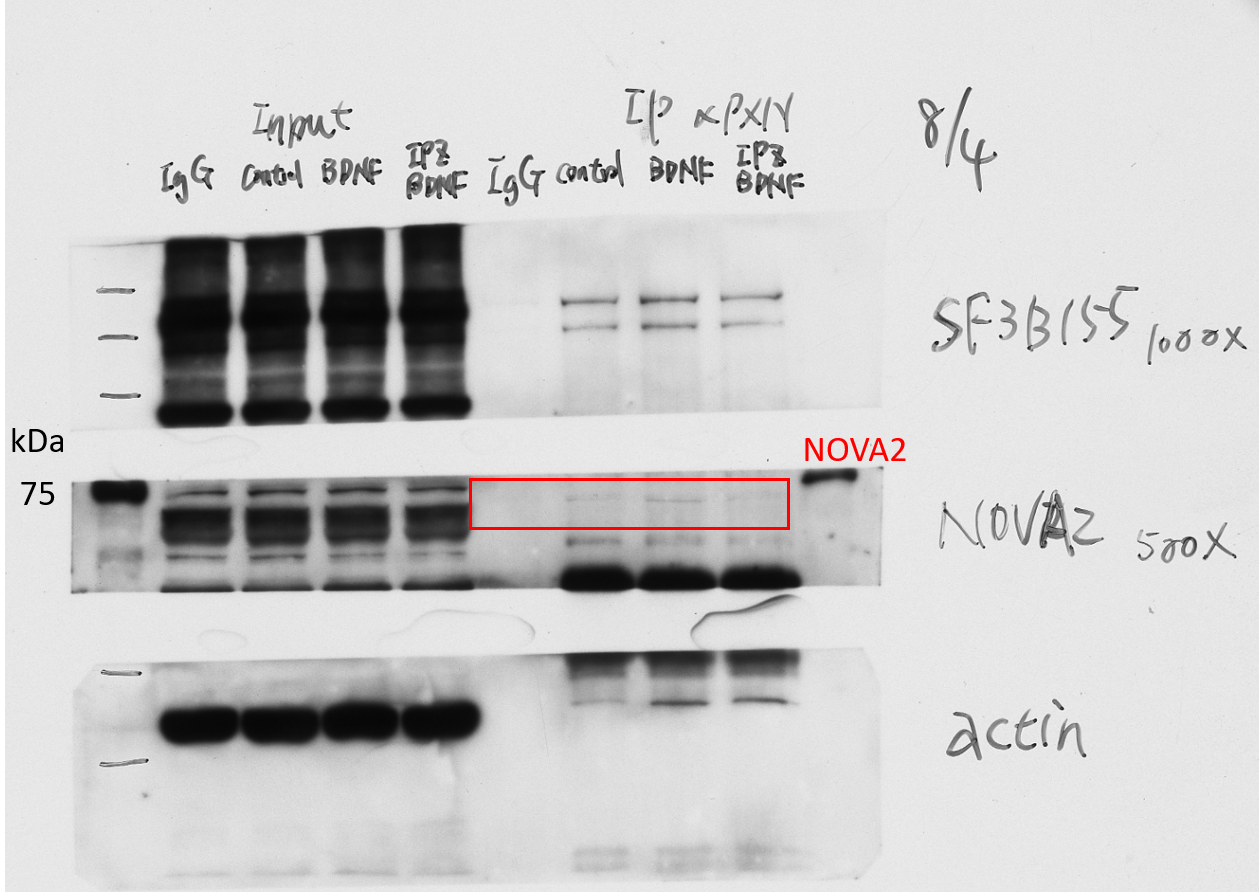

Supplement: Supplementary file 5 — Source data Fig. 4 [file 44318_2025_560_MOESM5_ESM.zip › Figure4/4C/Primary neuron_DIV7_IP-PXN_western_NOVA2_IP.tif]

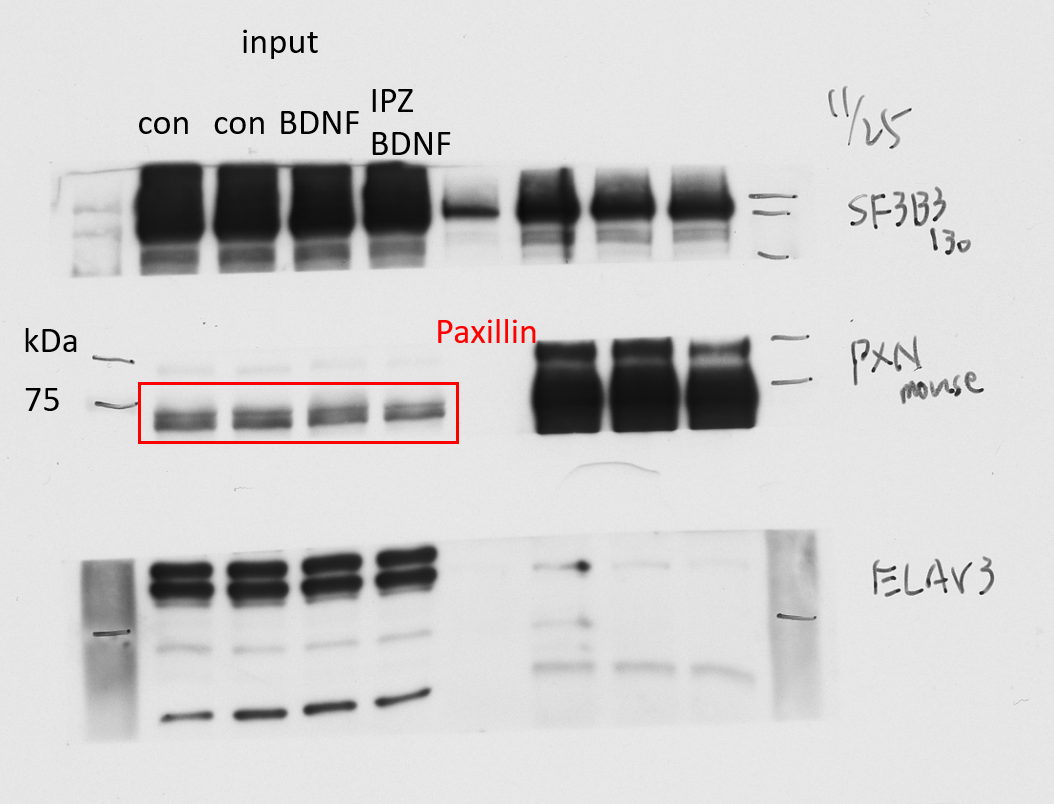

Supplement: Supplementary file 5 — Source data Fig. 4 [file 44318_2025_560_MOESM5_ESM.zip › Figure4/4C/Primary neuron_DIV7_IP-PXN_western_Paxillin_input.tif]

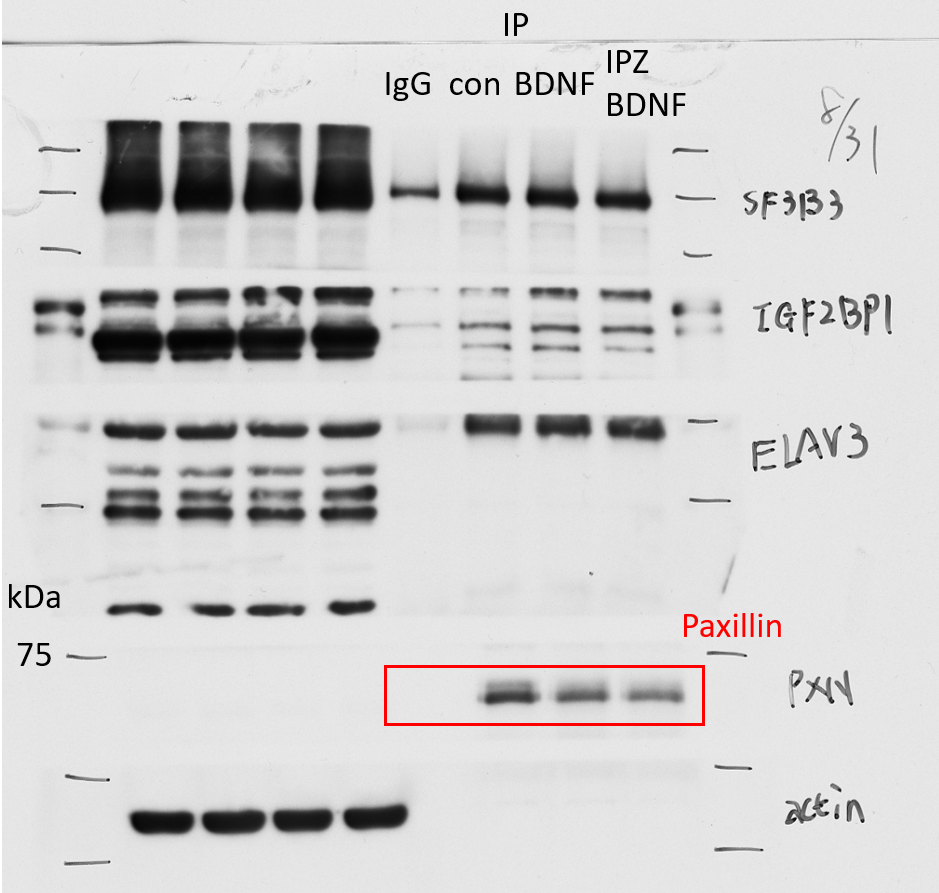

Supplement: Supplementary file 5 — Source data Fig. 4 [file 44318_2025_560_MOESM5_ESM.zip › Figure4/4C/Primary neuron_DIV7_IP-PXN_western_Paxillin_IP.tif]

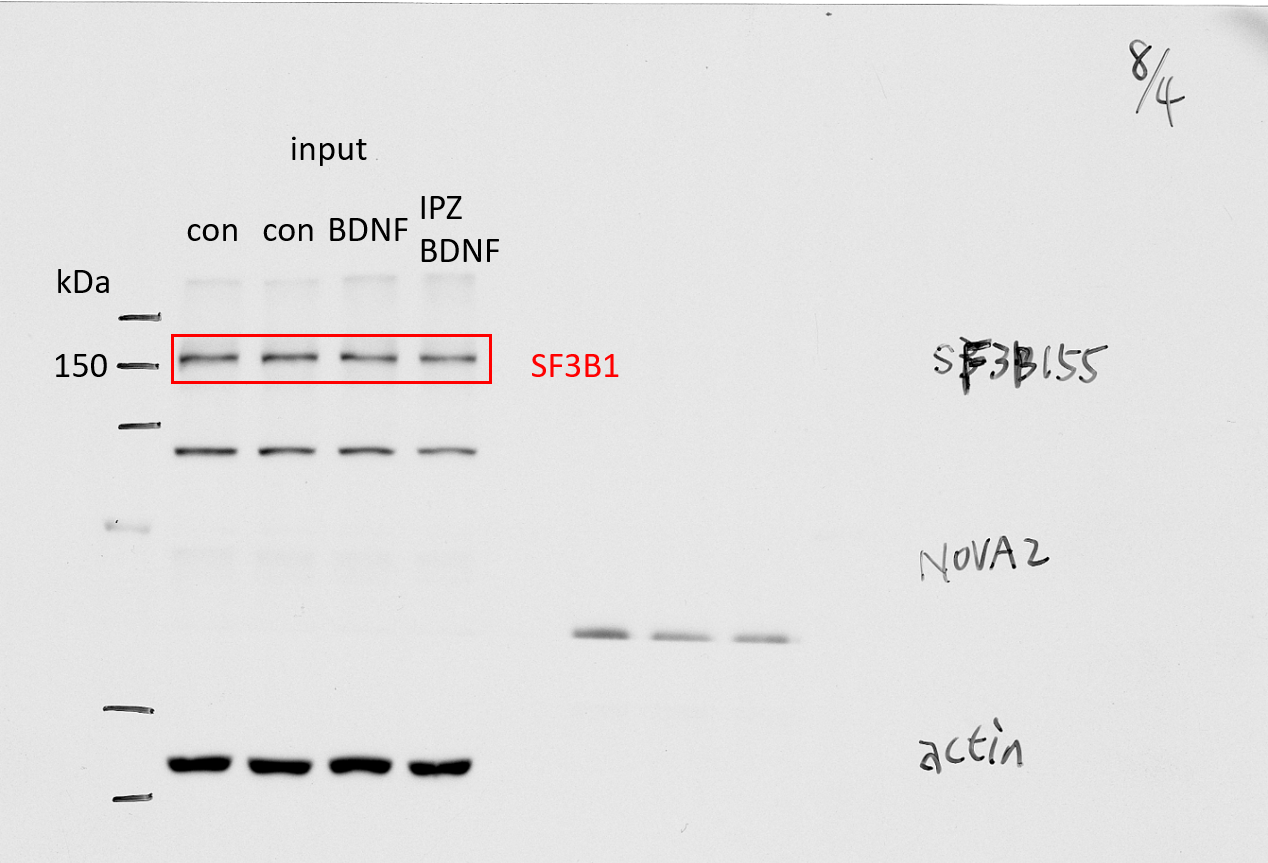

Supplement: Supplementary file 5 — Source data Fig. 4 [file 44318_2025_560_MOESM5_ESM.zip › Figure4/4C/Primary neuron_DIV7_IP-PXN_western_SF3B1_input.tif]

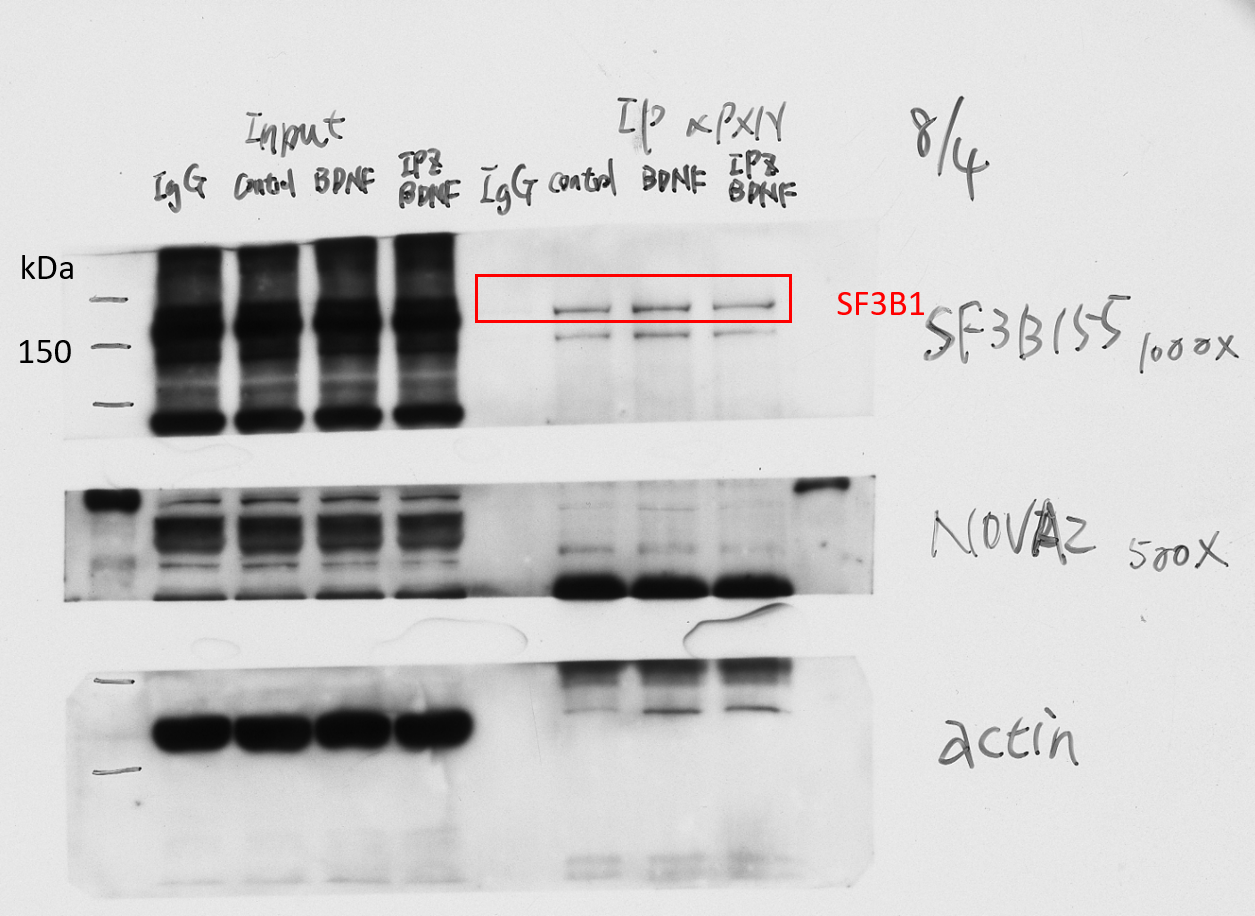

Supplement: Supplementary file 5 — Source data Fig. 4 [file 44318_2025_560_MOESM5_ESM.zip › Figure4/4C/Primary neuron_DIV7_IP-PXN_western_SF3B1_IP.tif]

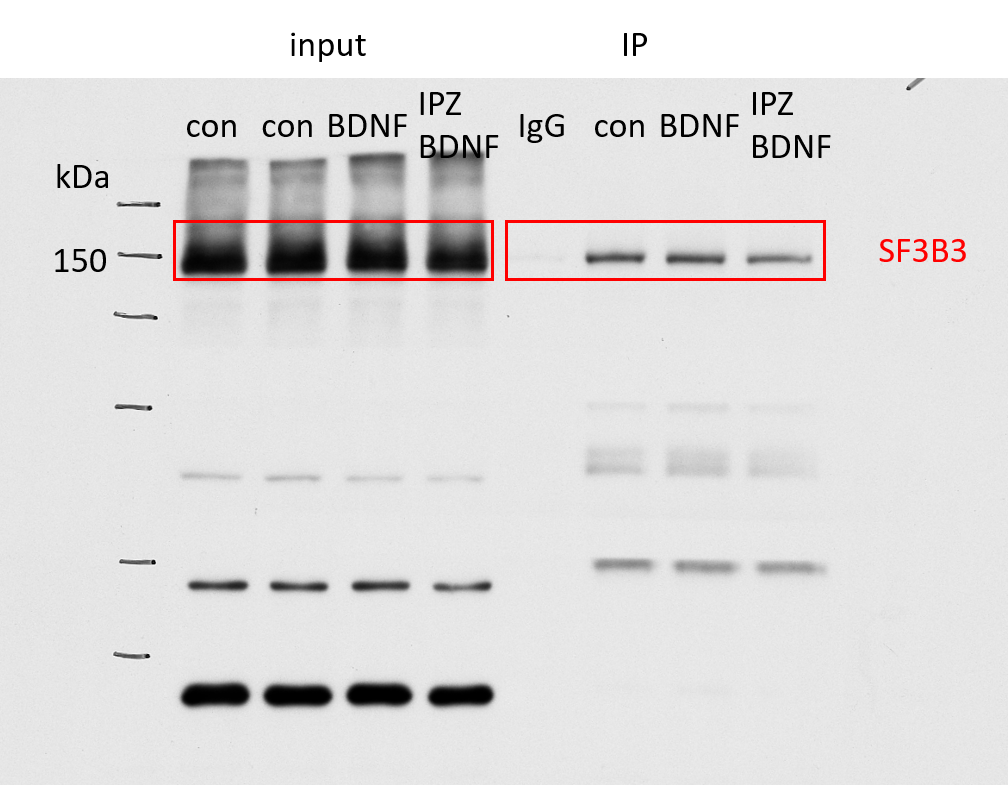

Supplement: Supplementary file 5 — Source data Fig. 4 [file 44318_2025_560_MOESM5_ESM.zip › Figure4/4C/Primary neuron_DIV7_IP-PXN_western_SF3B3_input,IP.tif]

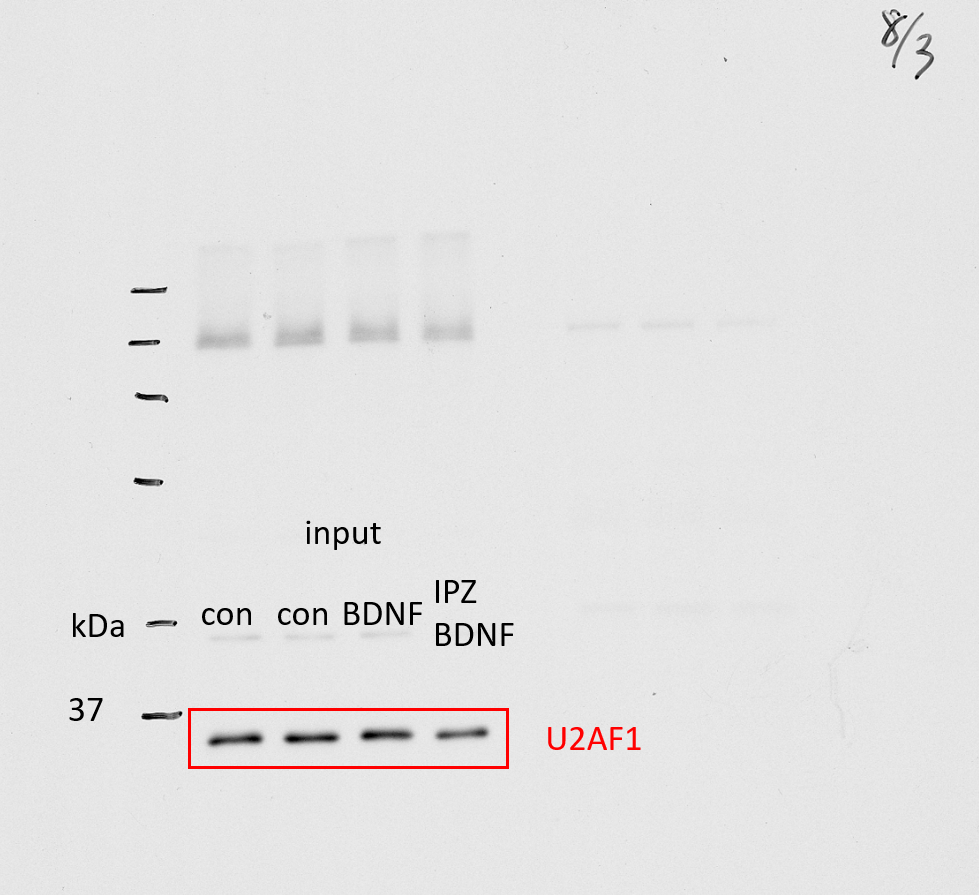

Supplement: Supplementary file 5 — Source data Fig. 4 [file 44318_2025_560_MOESM5_ESM.zip › Figure4/4C/Primary neuron_DIV7_IP-PXN_western_U2AF1_input.tif]

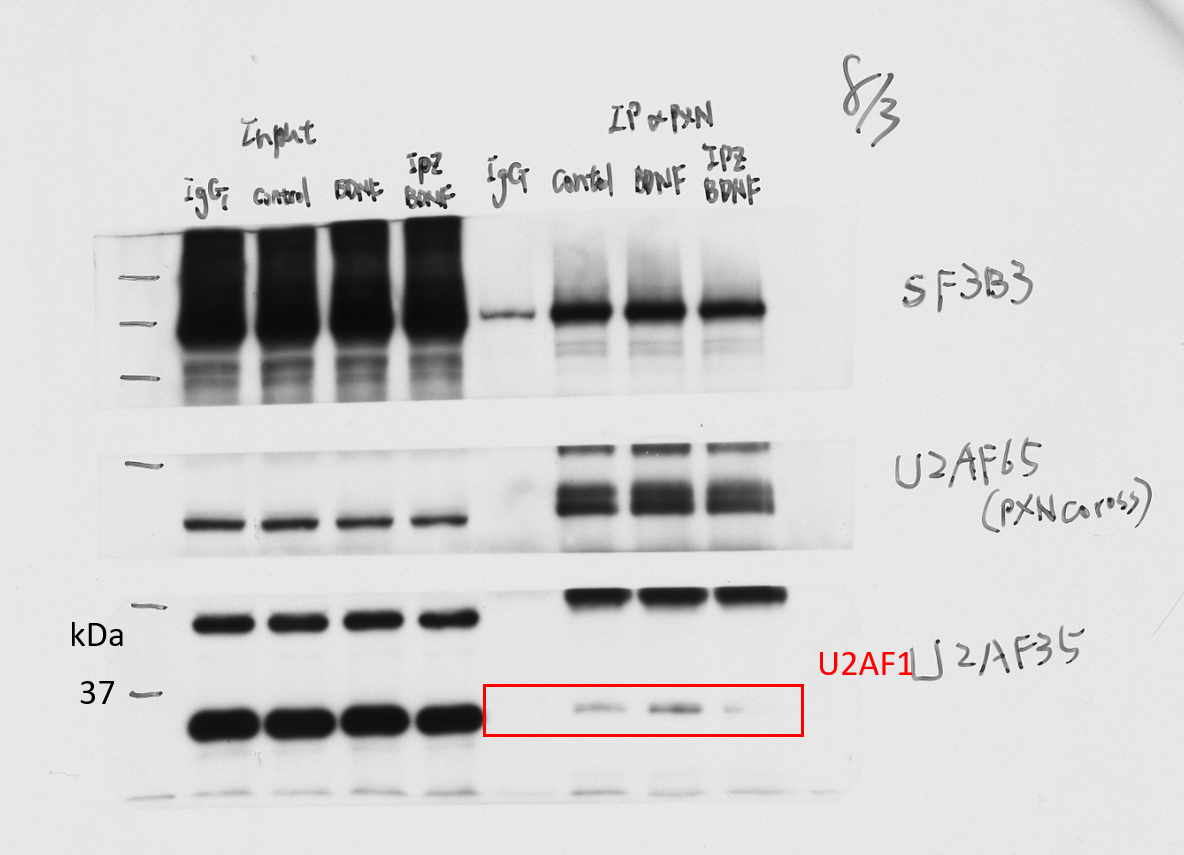

Supplement: Supplementary file 5 — Source data Fig. 4 [file 44318_2025_560_MOESM5_ESM.zip › Figure4/4C/Primary neuron_DIV7_IP-PXN_western_U2AF1_IP.tif]

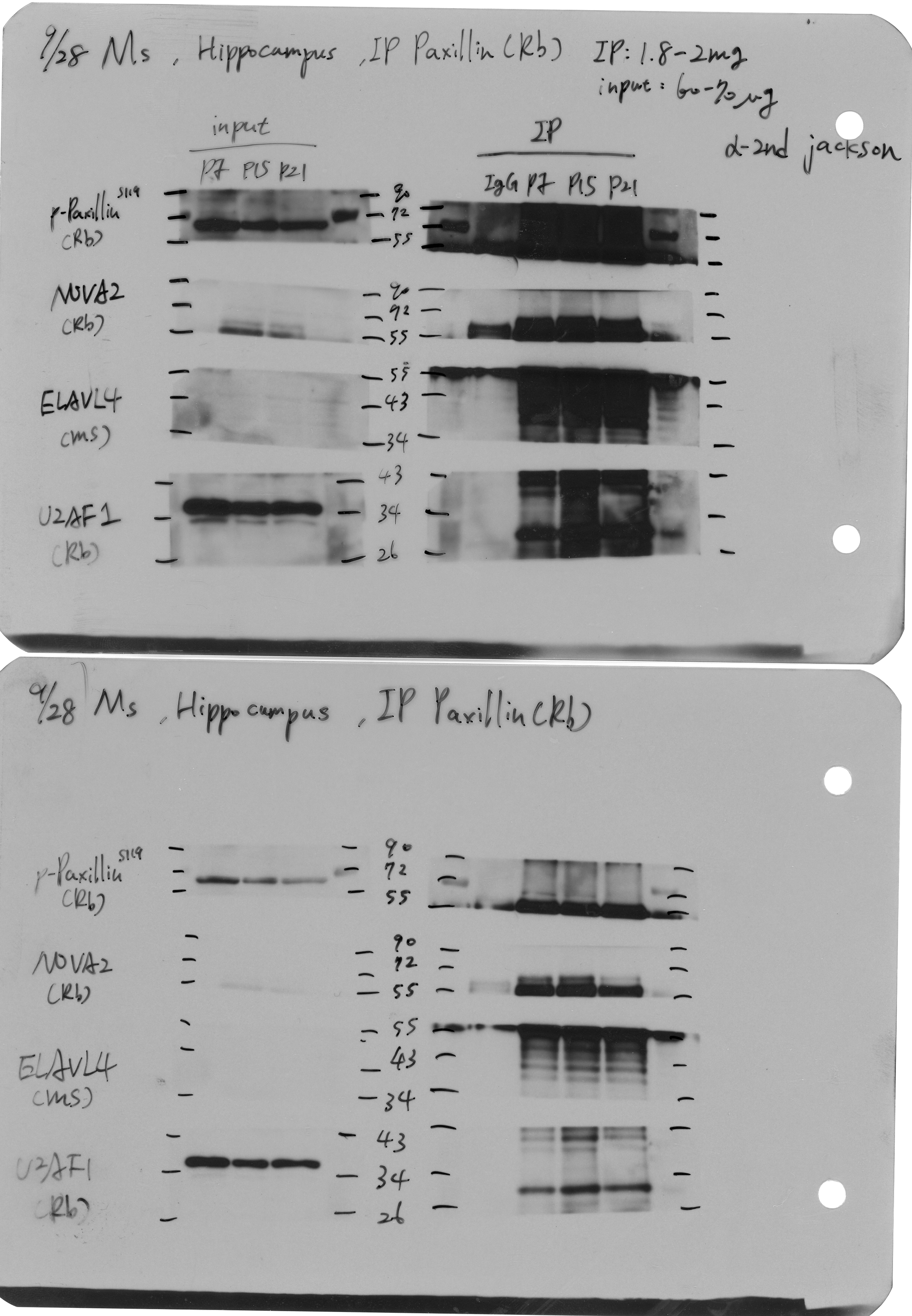

Supplement: Supplementary file 5 — Source data Fig. 4 [file 44318_2025_560_MOESM5_ESM.zip › Figure4/4E/Mouse brain_IP-Paxillin western_NOVA2_p-PaxillinS119_ELAVL4_U2AF1-1.tif]

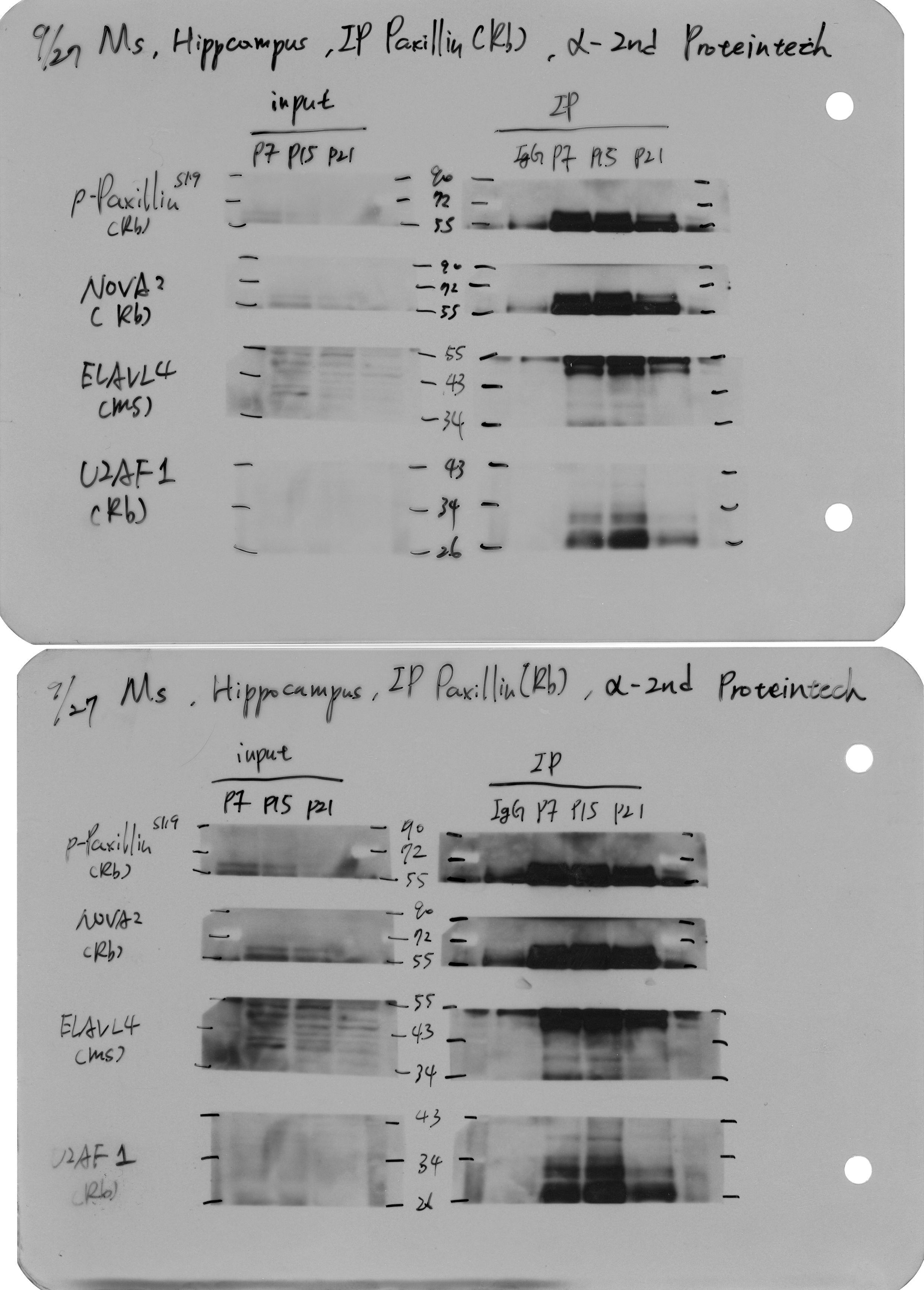

Supplement: Supplementary file 5 — Source data Fig. 4 [file 44318_2025_560_MOESM5_ESM.zip › Figure4/4E/Mouse brain_IP-Paxillin western_NOVA2_p-PaxillinS119_ELAVL4_U2AF1-2.tif]

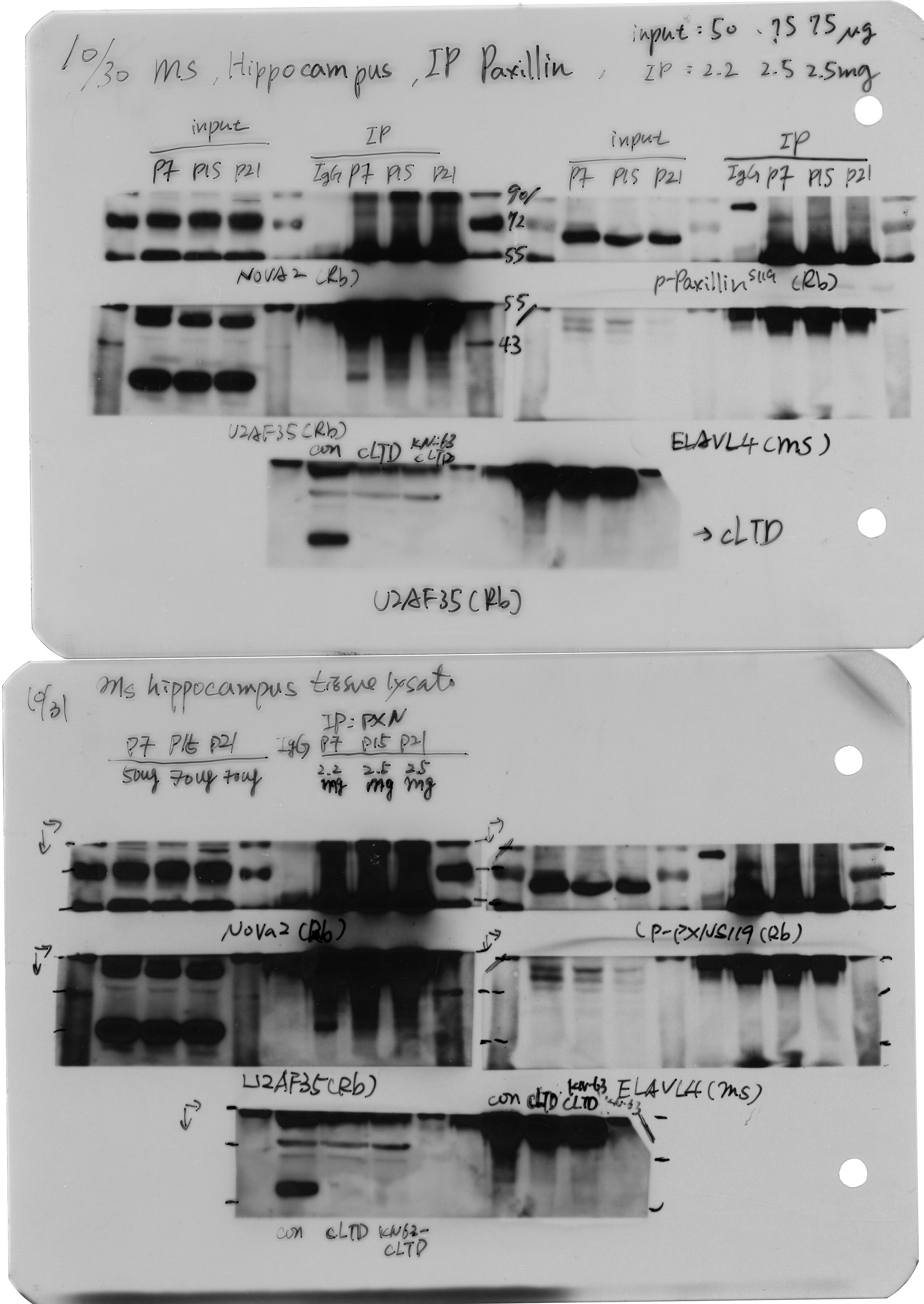

Supplement: Supplementary file 5 — Source data Fig. 4 [file 44318_2025_560_MOESM5_ESM.zip › Figure4/4E/Mouse brain_IP-Paxillin western_NOVA2_p-PaxillinS119_ELAVL4_U2AF1-3.tif]

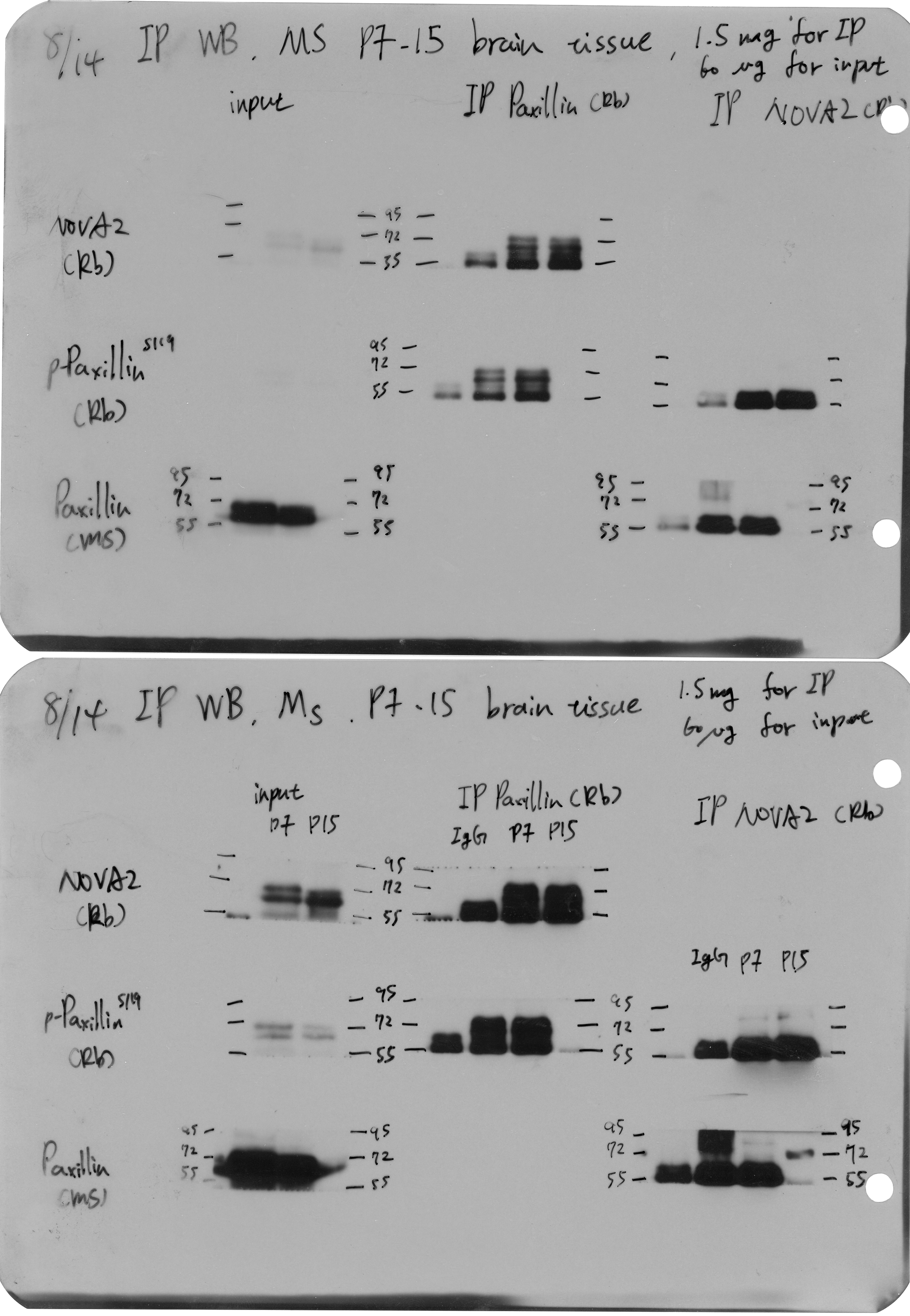

Supplement: Supplementary file 5 — Source data Fig. 4 [file 44318_2025_560_MOESM5_ESM.zip › Figure4/4E/Mouse brain_IP-Paxillin western_NOVA2_Paxillin_p-PaxillinS119-1.tif]

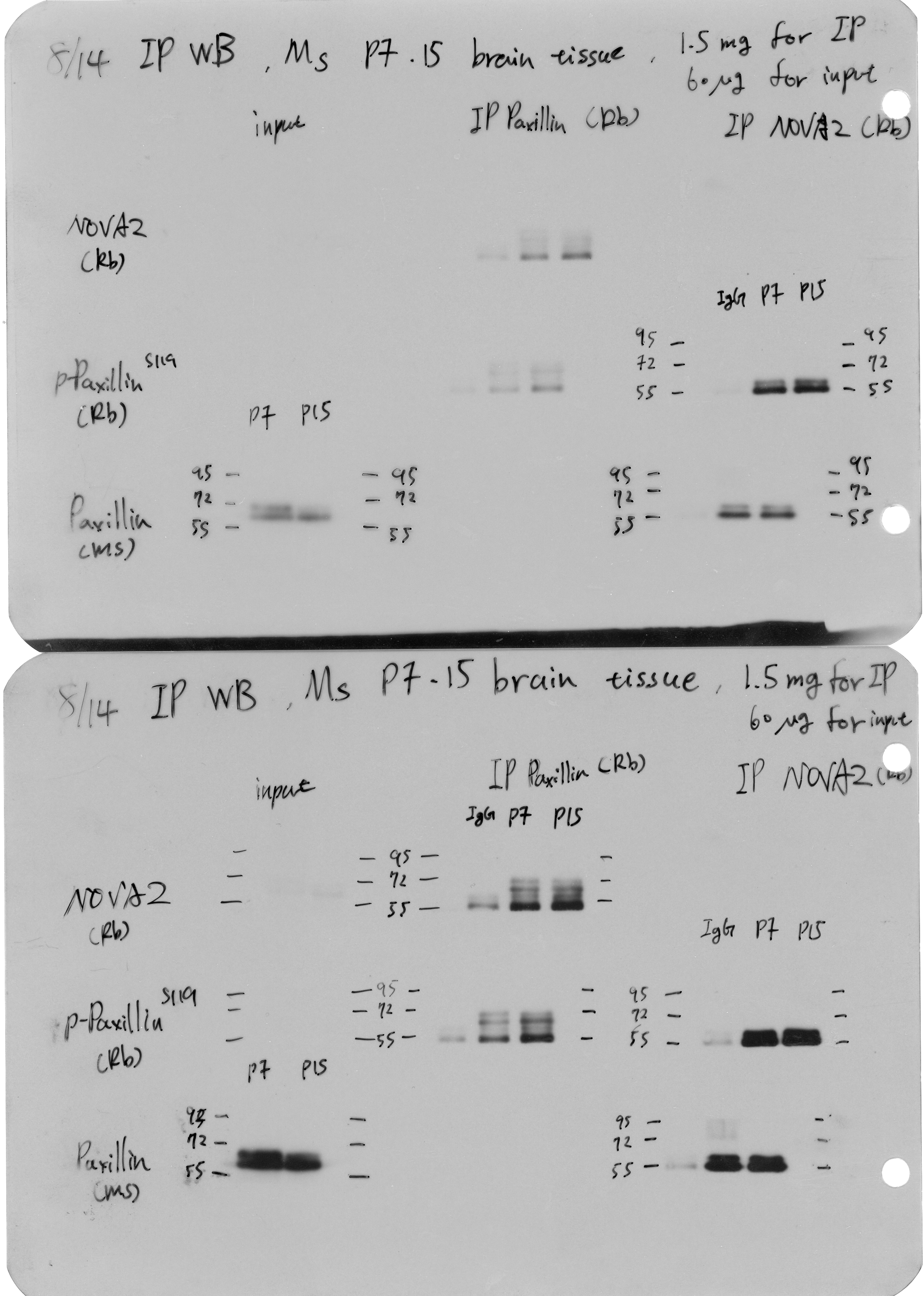

Supplement: Supplementary file 5 — Source data Fig. 4 [file 44318_2025_560_MOESM5_ESM.zip › Figure4/4E/Mouse brain_IP-Paxillin western_NOVA2_Paxillin_p-PaxillinS119-2.tif]

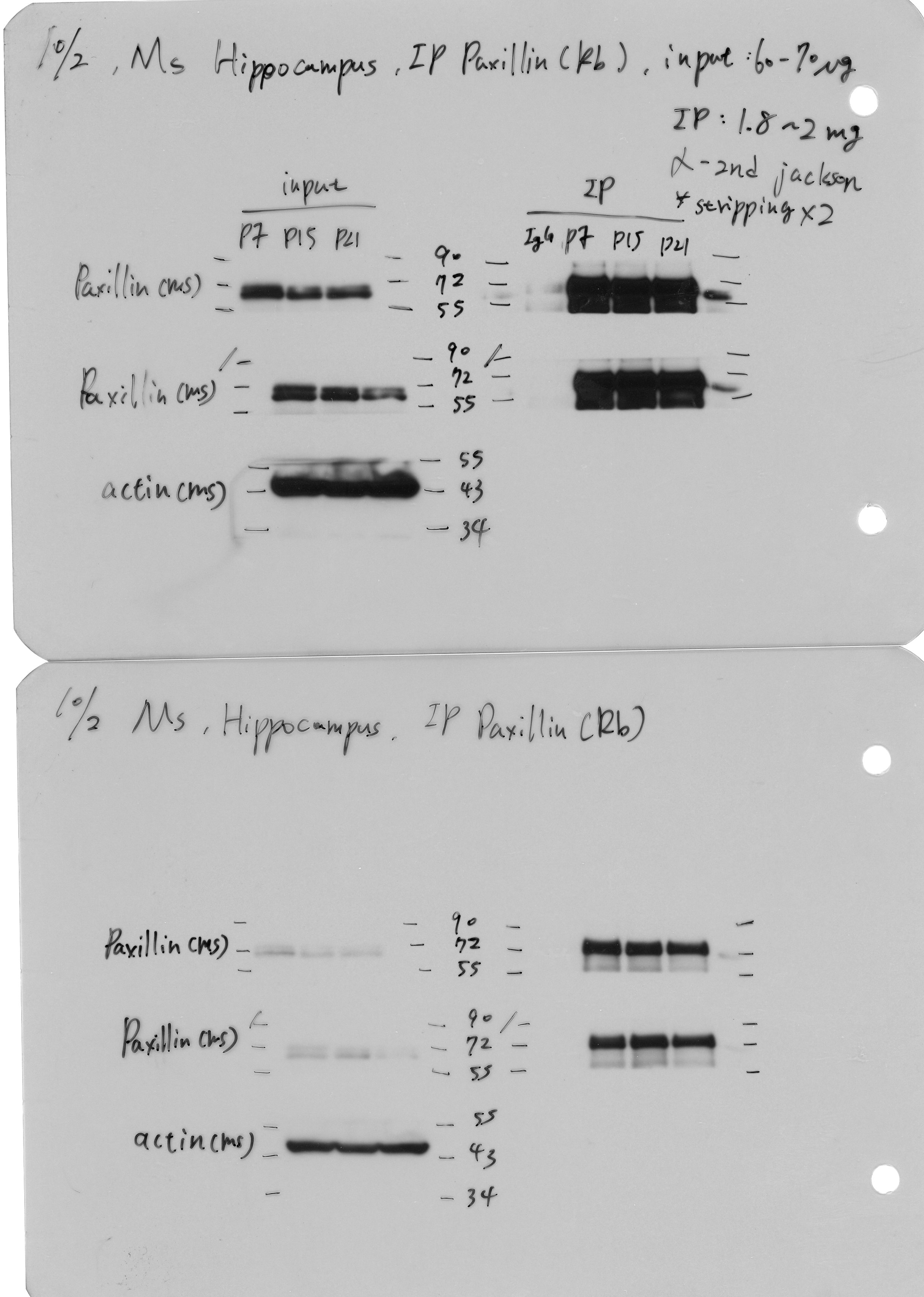

Supplement: Supplementary file 5 — Source data Fig. 4 [file 44318_2025_560_MOESM5_ESM.zip › Figure4/4E/Mouse brain_IP-Paxillin western_Paxillin_Actin.tif]

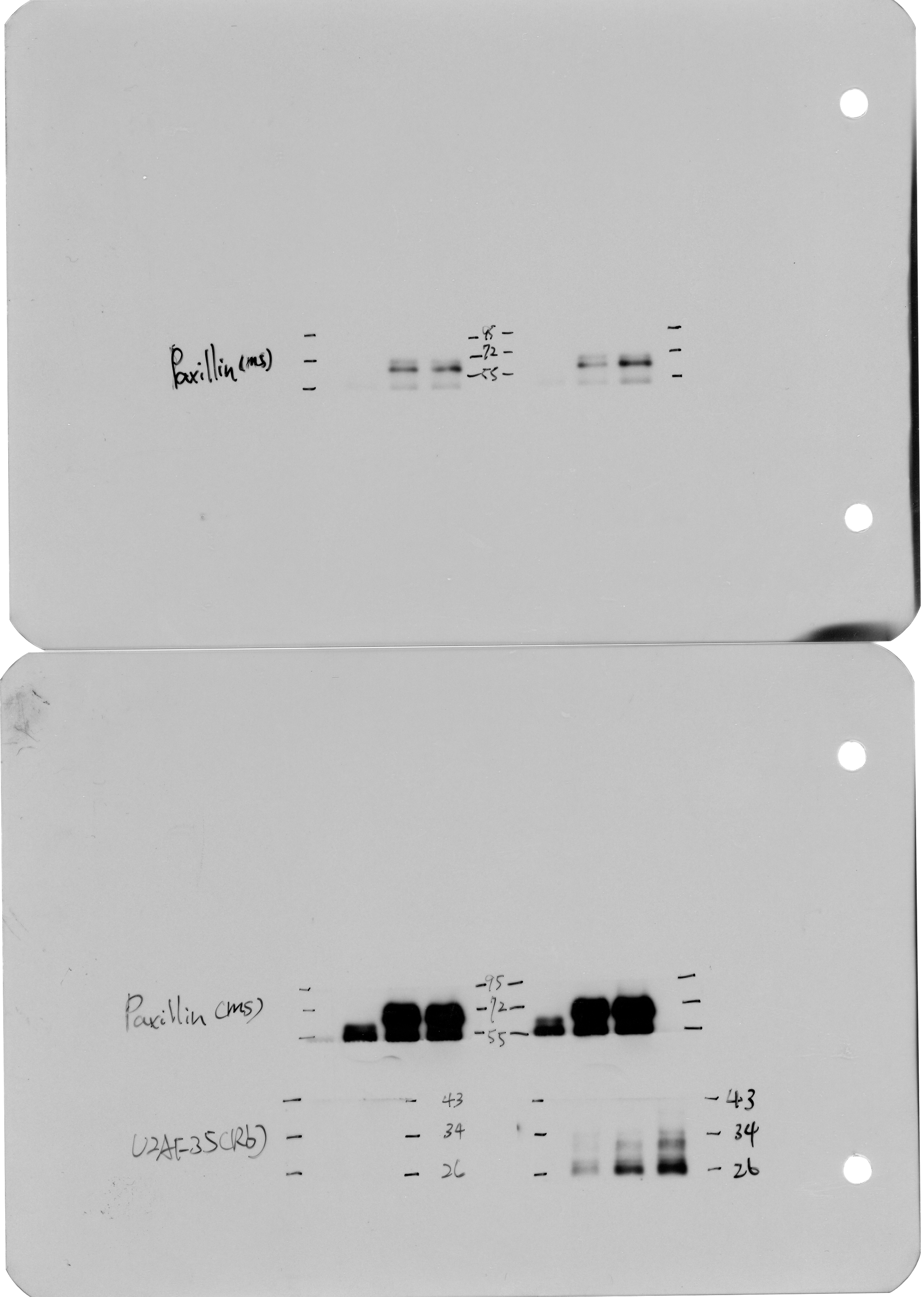

Supplement: Supplementary file 5 — Source data Fig. 4 [file 44318_2025_560_MOESM5_ESM.zip › Figure4/4E/Mouse brain_IP-Paxillin western_Paxillin_U2AF1.tif]

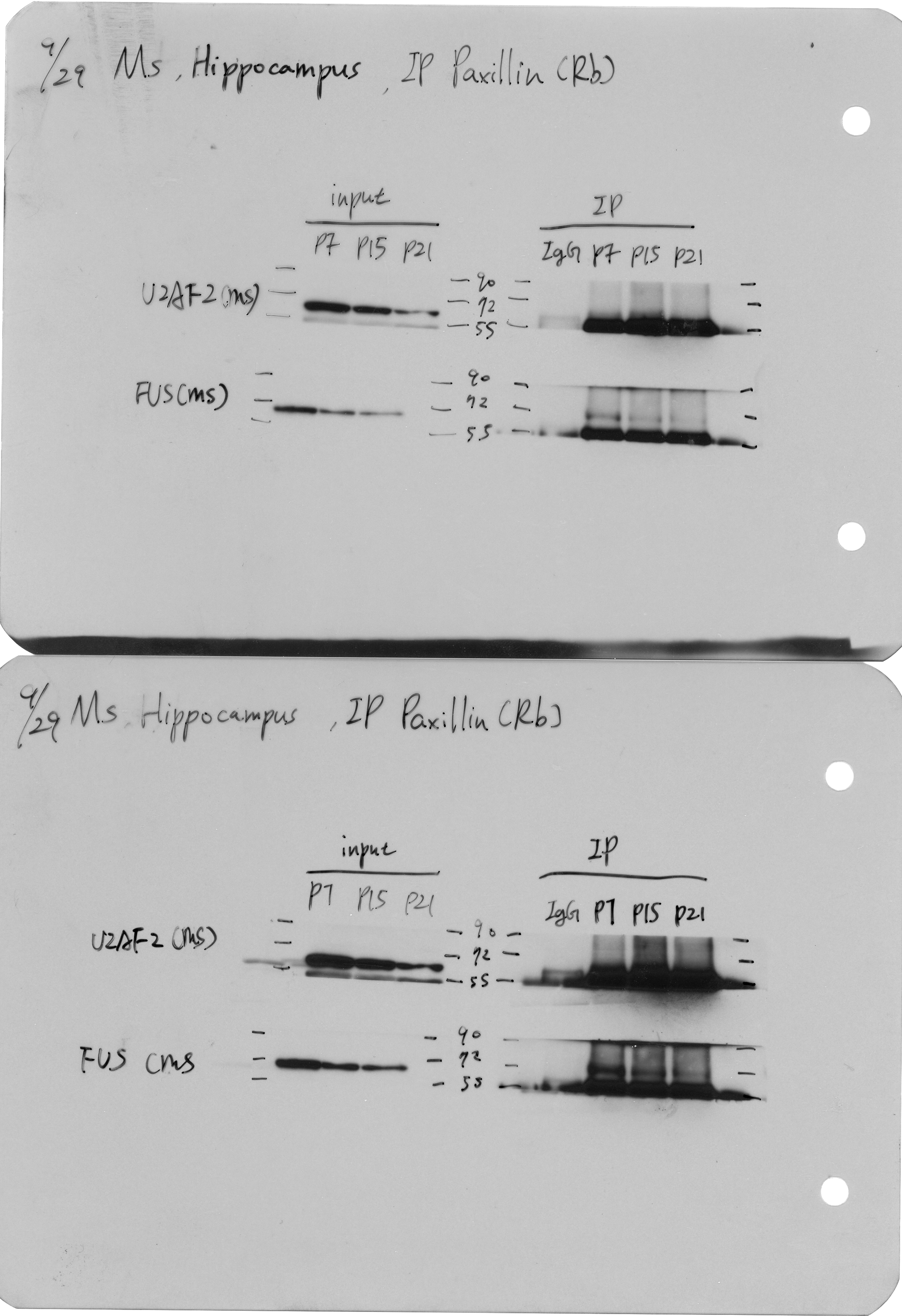

Supplement: Supplementary file 5 — Source data Fig. 4 [file 44318_2025_560_MOESM5_ESM.zip › Figure4/4E/Mouse brain_IP-Paxillin western_U2AF2_FUS.tif]

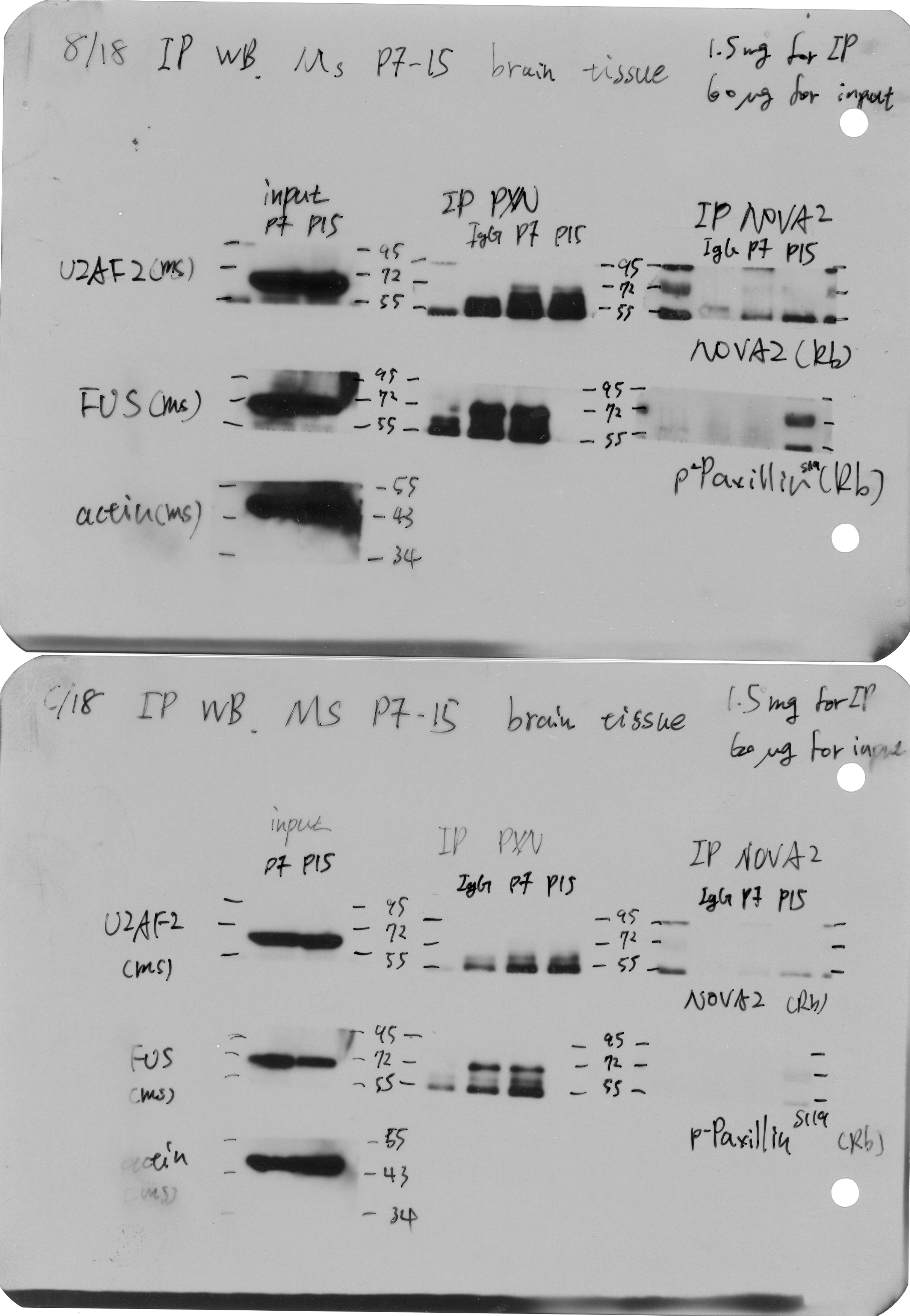

Supplement: Supplementary file 5 — Source data Fig. 4 [file 44318_2025_560_MOESM5_ESM.zip › Figure4/4E/Mouse brain_IP-Paxillin western_U2AF2_FUS_Actin-1.tif]

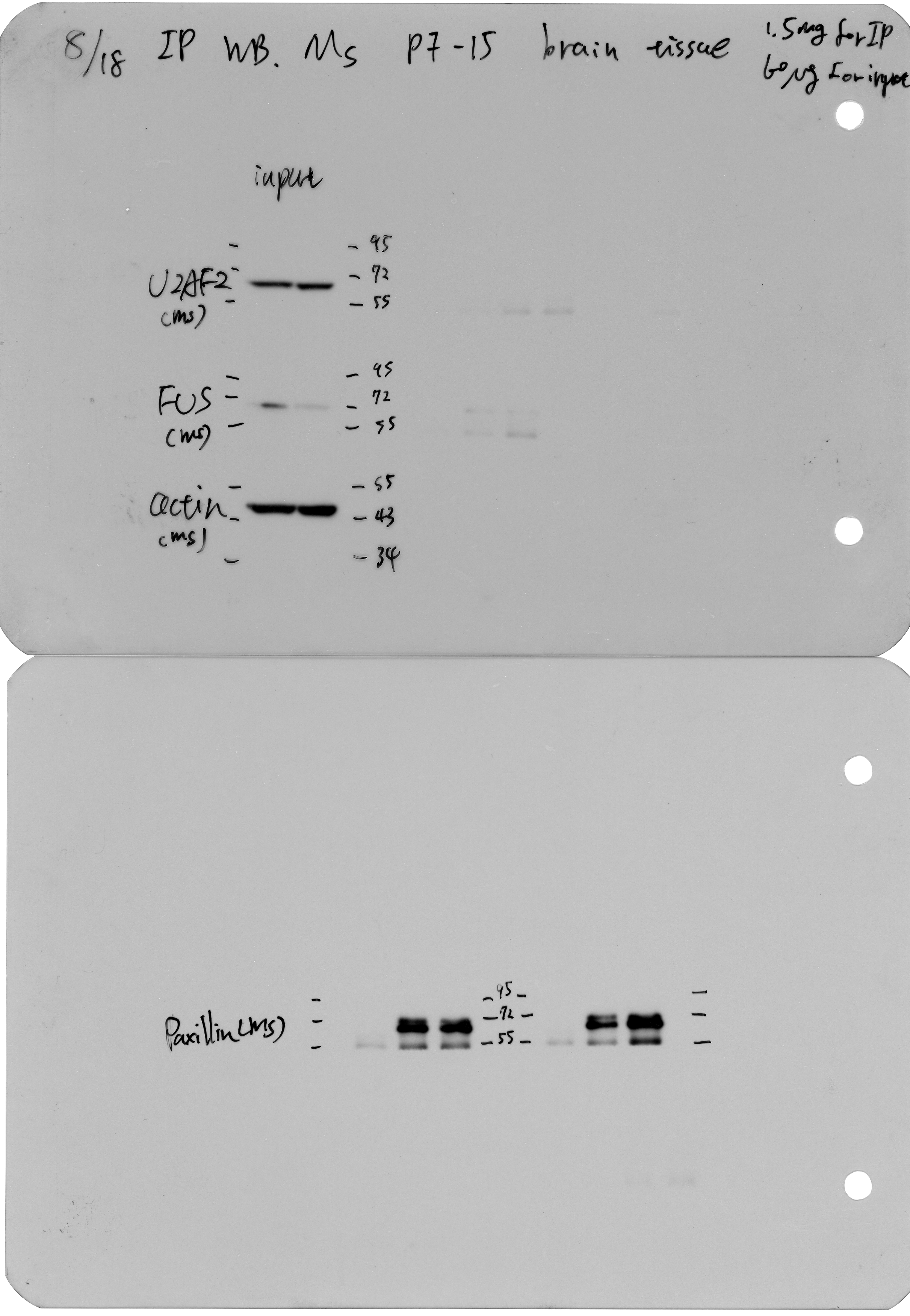

Supplement: Supplementary file 5 — Source data Fig. 4 [file 44318_2025_560_MOESM5_ESM.zip › Figure4/4E/Mouse brain_IP-Paxillin western_U2AF2_FUS_Actin-2.tif]

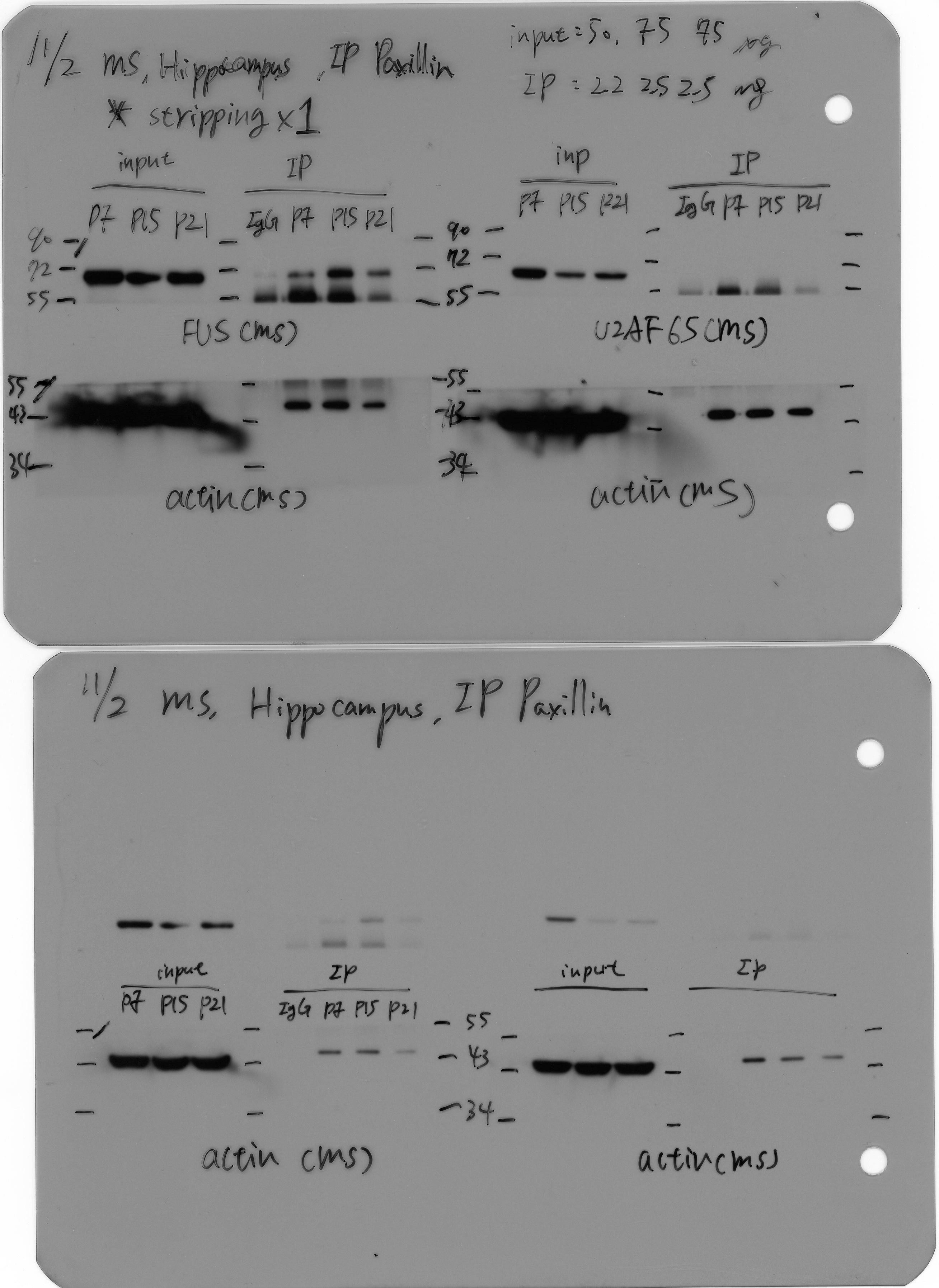

Supplement: Supplementary file 5 — Source data Fig. 4 [file 44318_2025_560_MOESM5_ESM.zip › Figure4/4E/Mouse brain_IP-Paxillin western_U2AF2_FUS_Actin-3.tif]

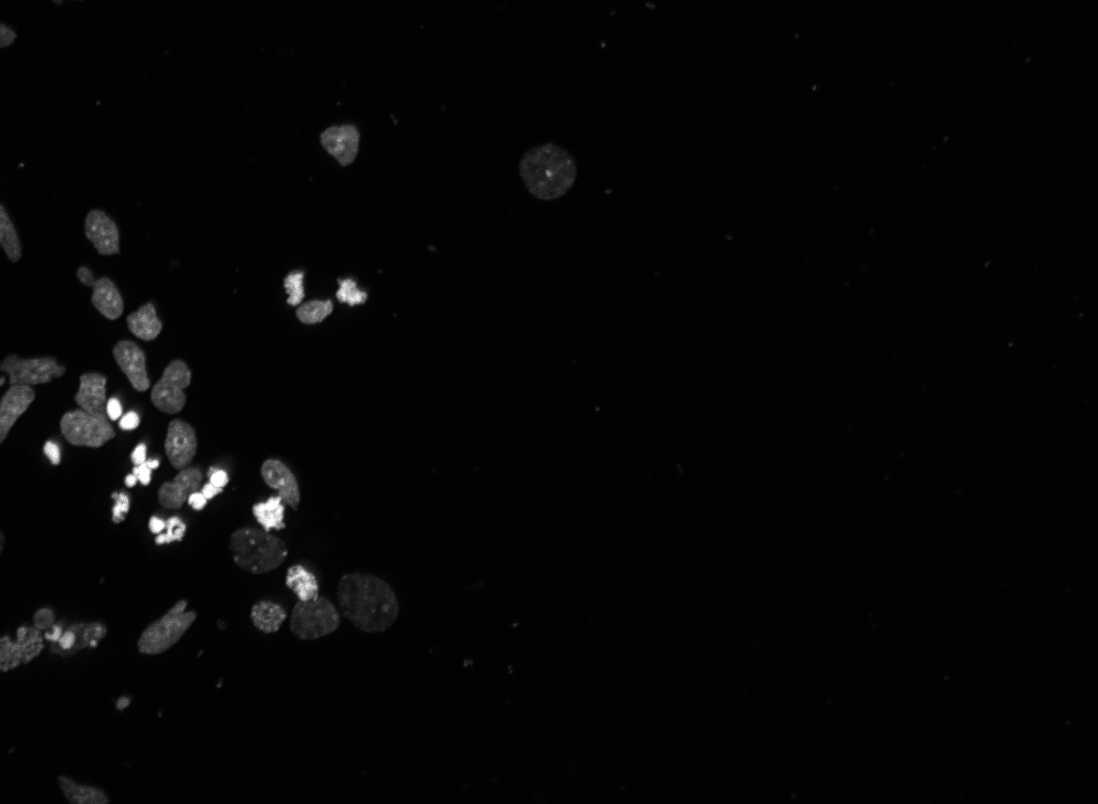

Supplement: Supplementary file 5 — Source data Fig. 4 [file 44318_2025_560_MOESM5_ESM.zip › Figure4/4F/Primary neuron_DIV7_DAPI.tif]

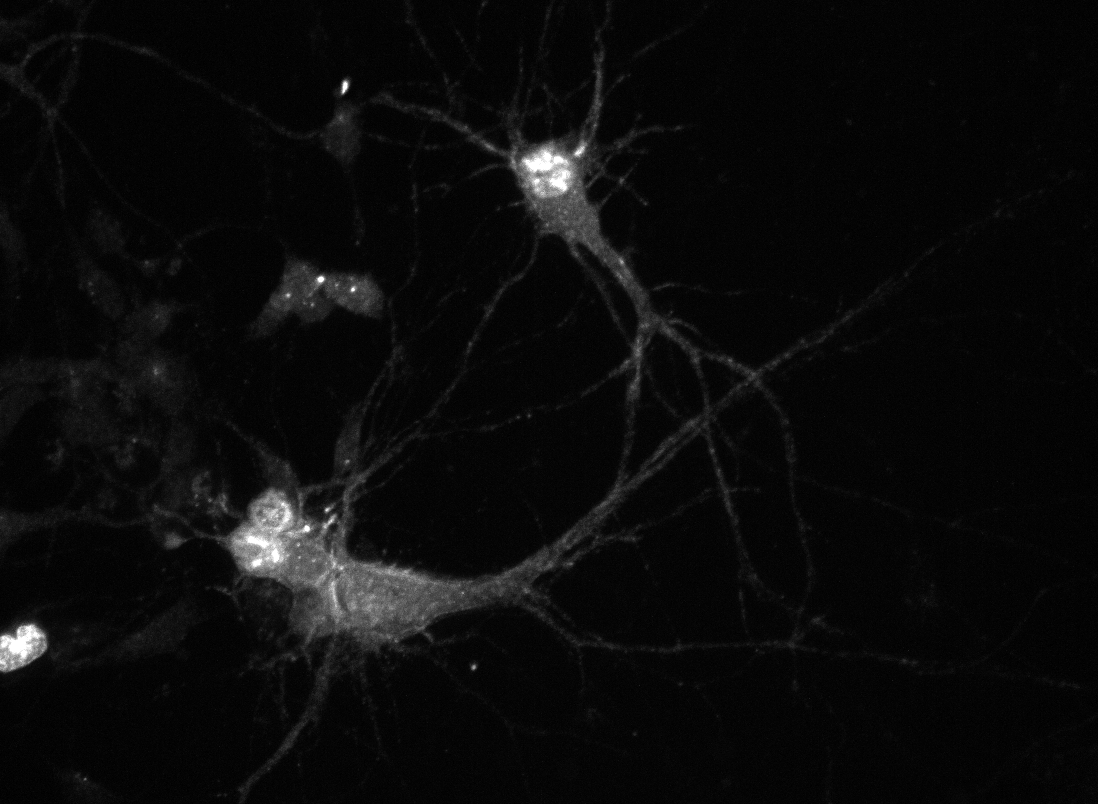

Supplement: Supplementary file 5 — Source data Fig. 4 [file 44318_2025_560_MOESM5_ESM.zip › Figure4/4F/Primary neuron_DIV7_p-PaxillinS119.tif]

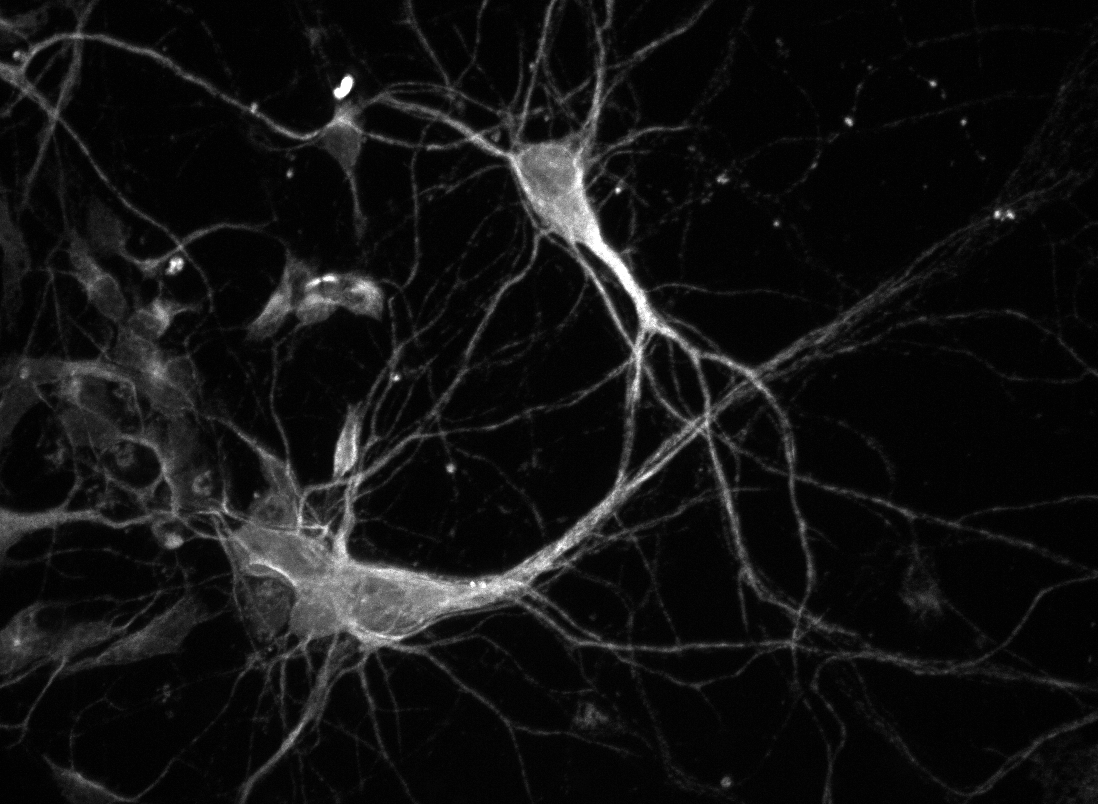

Supplement: Supplementary file 5 — Source data Fig. 4 [file 44318_2025_560_MOESM5_ESM.zip › Figure4/4F/Primary neuron_DIV7_Tuj-1.tif]

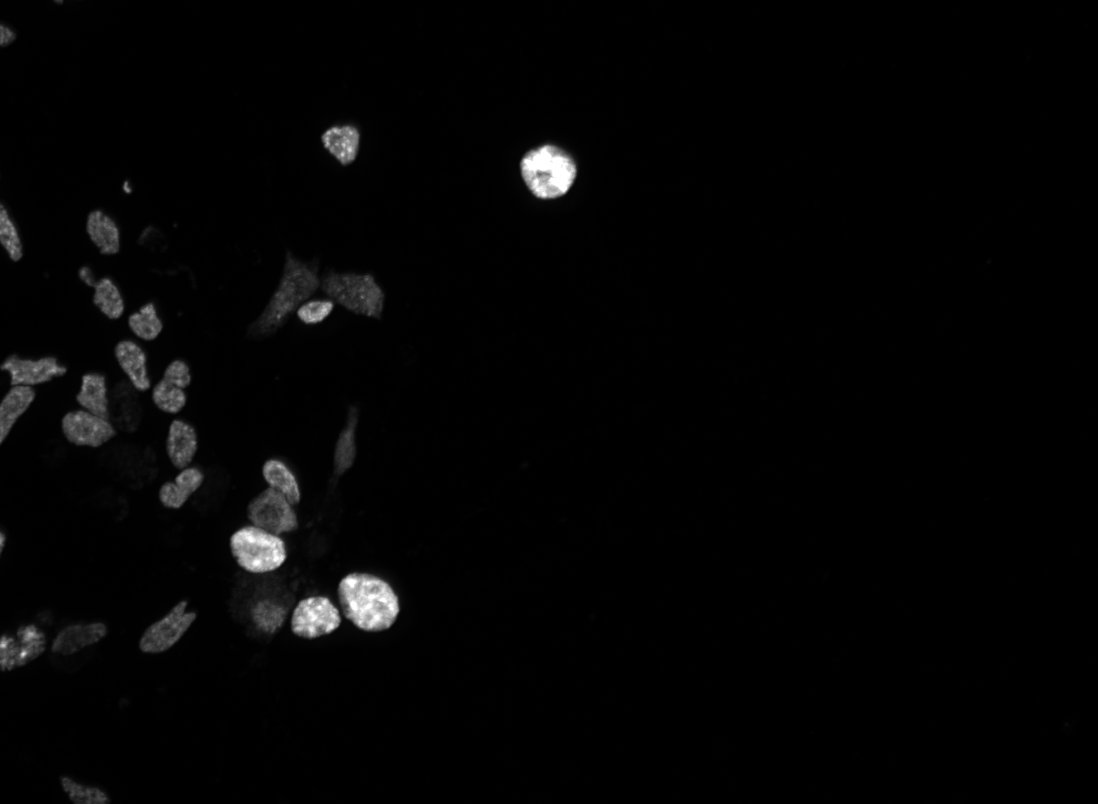

Supplement: Supplementary file 5 — Source data Fig. 4 [file 44318_2025_560_MOESM5_ESM.zip › Figure4/4F/Primary neuron_DIV7_U2AF2.tif]

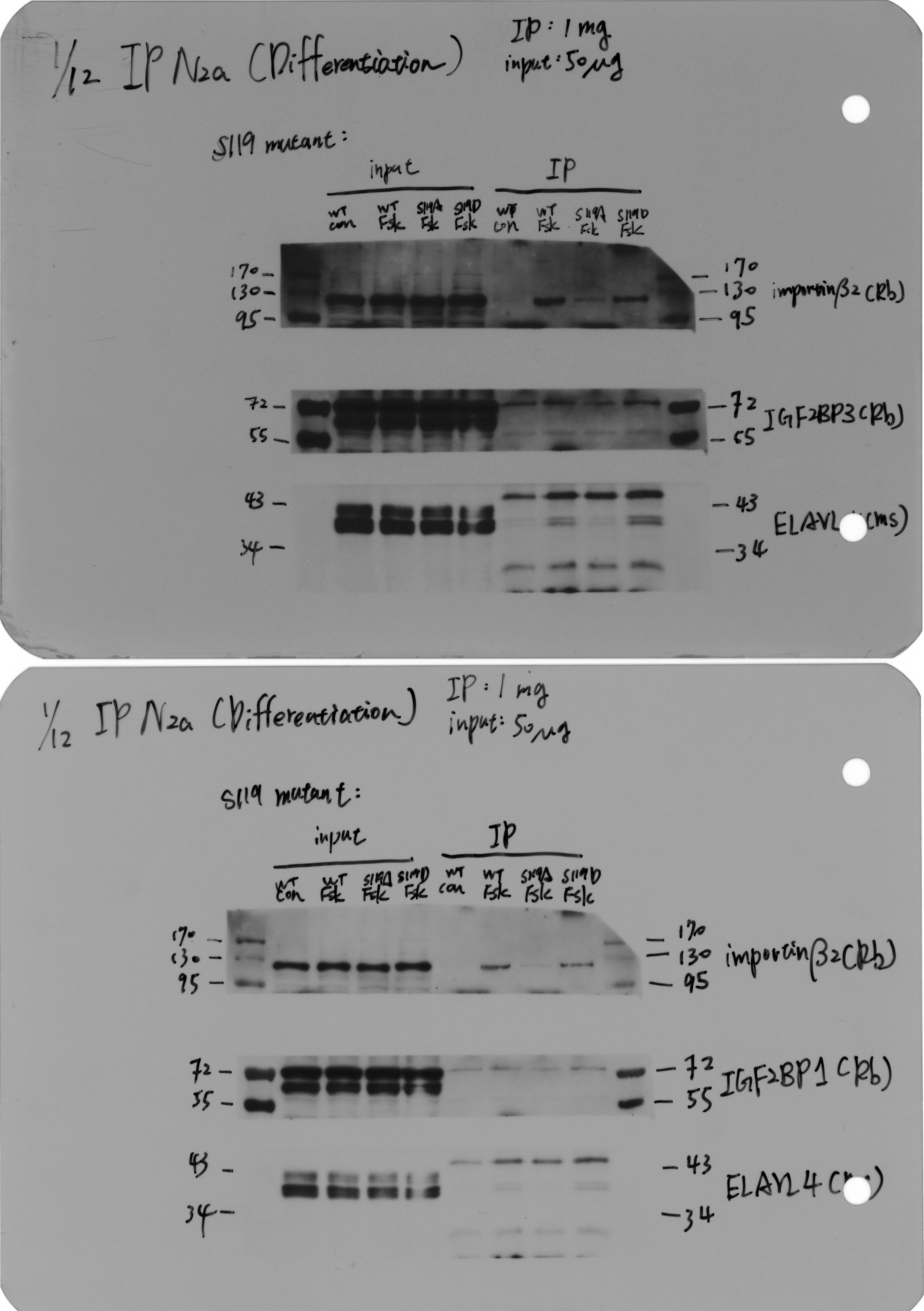

Supplement: Supplementary file 5 — Source data Fig. 4 [file 44318_2025_560_MOESM5_ESM.zip › Figure4/4G/N2a_Differentiation_IP-FLAG western_importinB2_IGF2BP1_ELAVL4.tif]

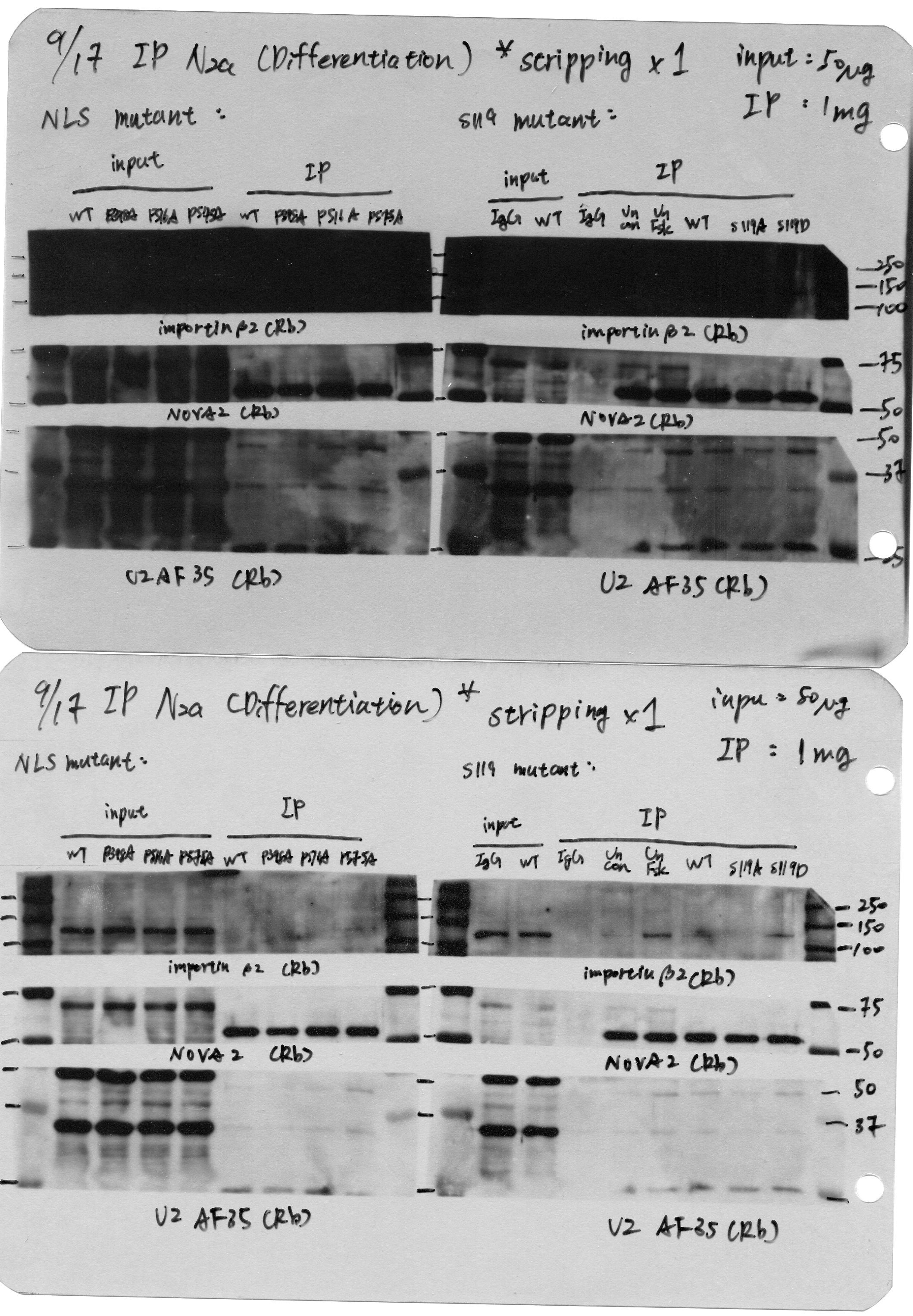

Supplement: Supplementary file 5 — Source data Fig. 4 [file 44318_2025_560_MOESM5_ESM.zip › Figure4/4G/N2a_Differentiation_IP-FLAG western_importinB2_NOVA2_U2AF1-2.tif]

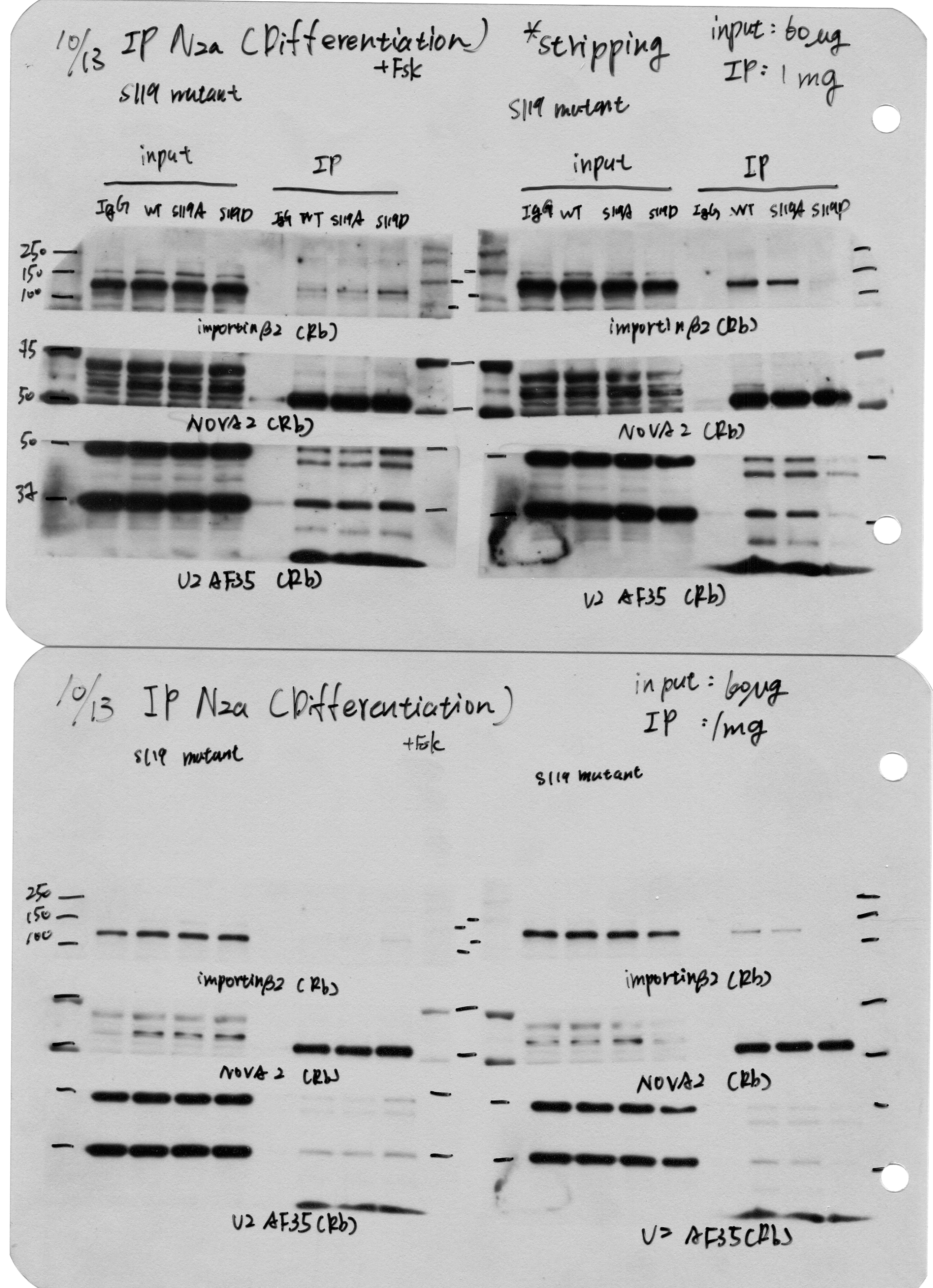

Supplement: Supplementary file 5 — Source data Fig. 4 [file 44318_2025_560_MOESM5_ESM.zip › Figure4/4G/N2a_Differentiation_IP-FLAG western_importinB2_NOVA2_U2AF1-3.tif]

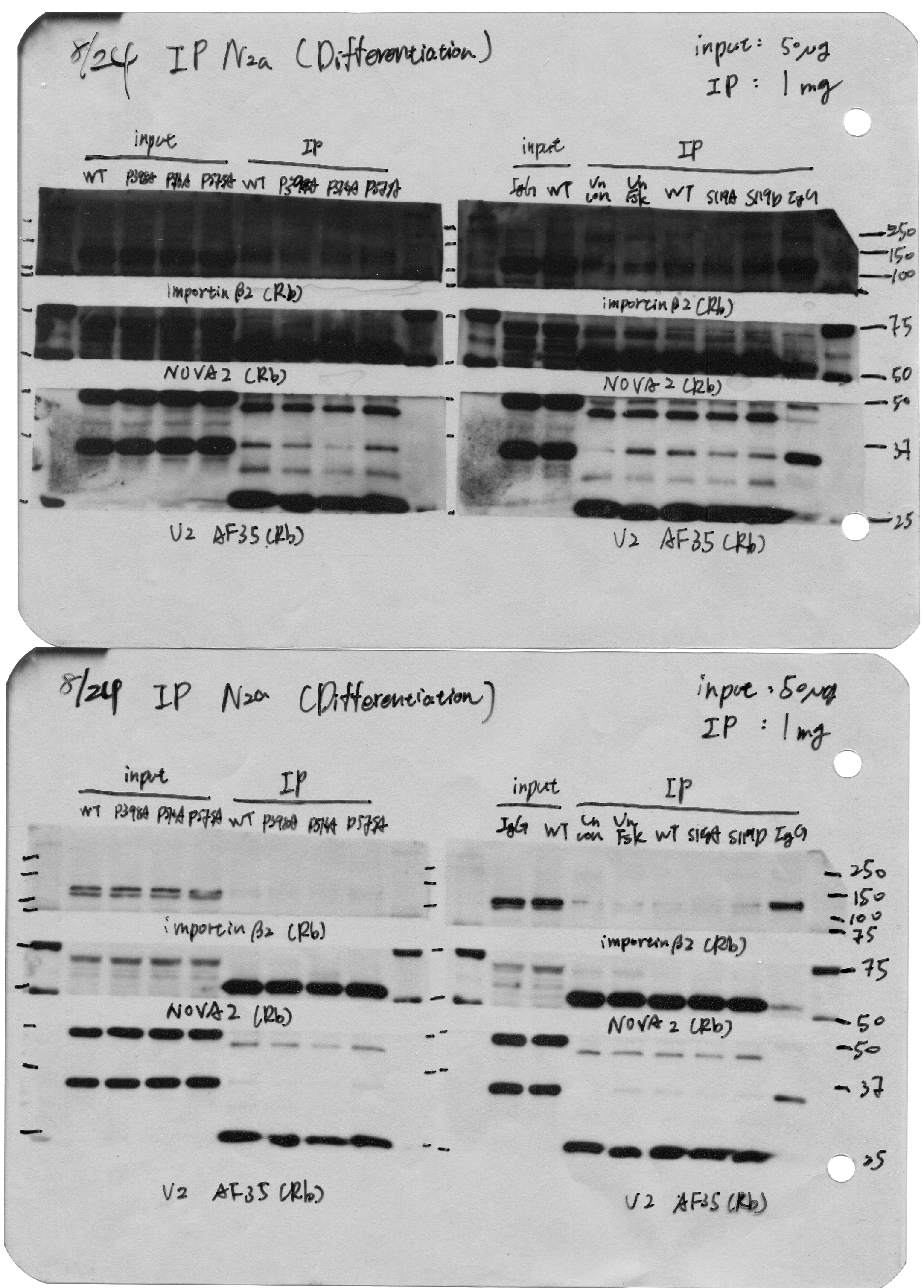

Supplement: Supplementary file 5 — Source data Fig. 4 [file 44318_2025_560_MOESM5_ESM.zip › Figure4/4G/N2a_Differentiation_IP-FLAG western_importinB_NOVA2_U2AF1-1.tif]

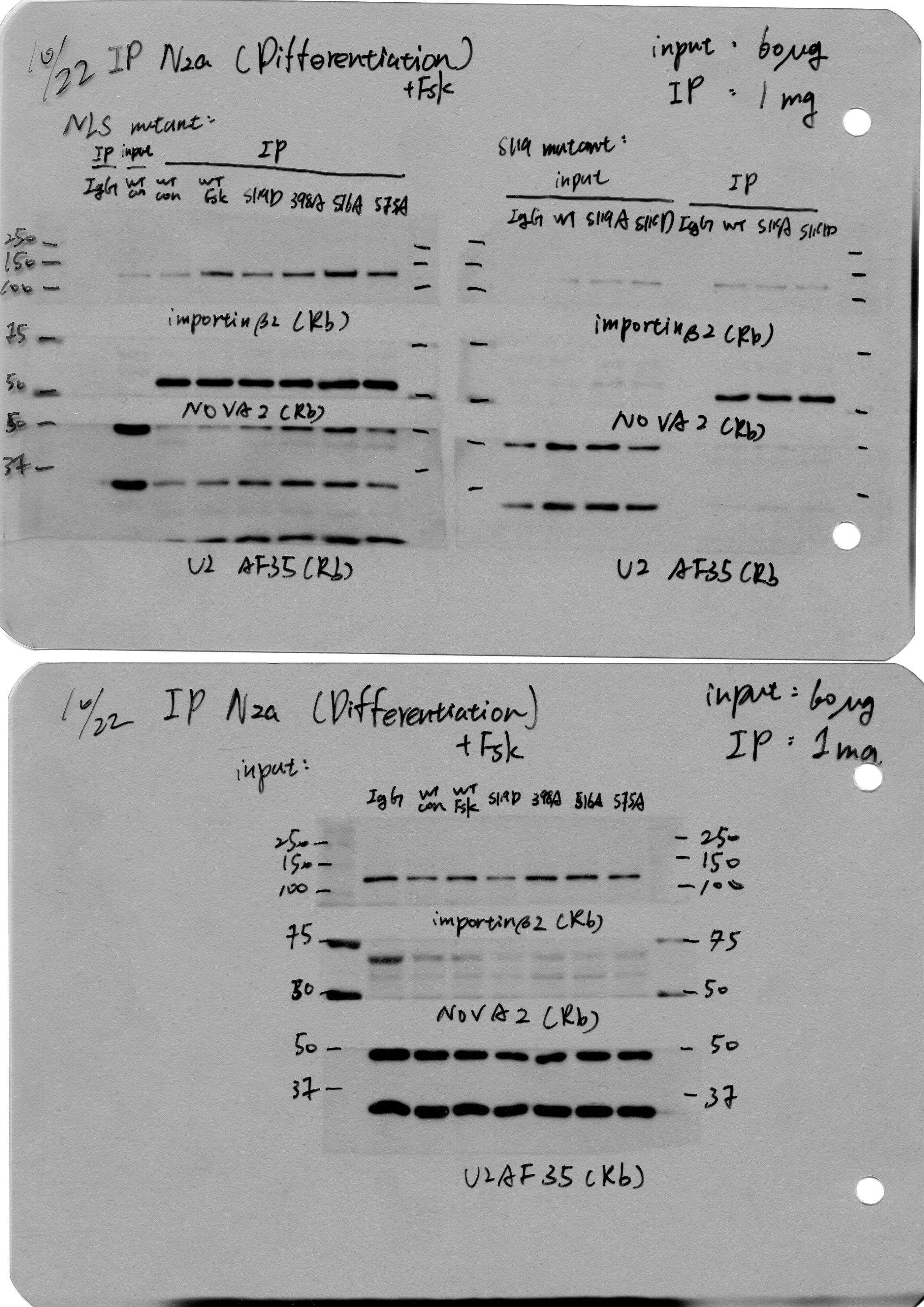

Supplement: Supplementary file 5 — Source data Fig. 4 [file 44318_2025_560_MOESM5_ESM.zip › Figure4/4G/N2a_Differentiation_IP-FLAG western_importinB_NOVA2_U2AF1-4.tif]

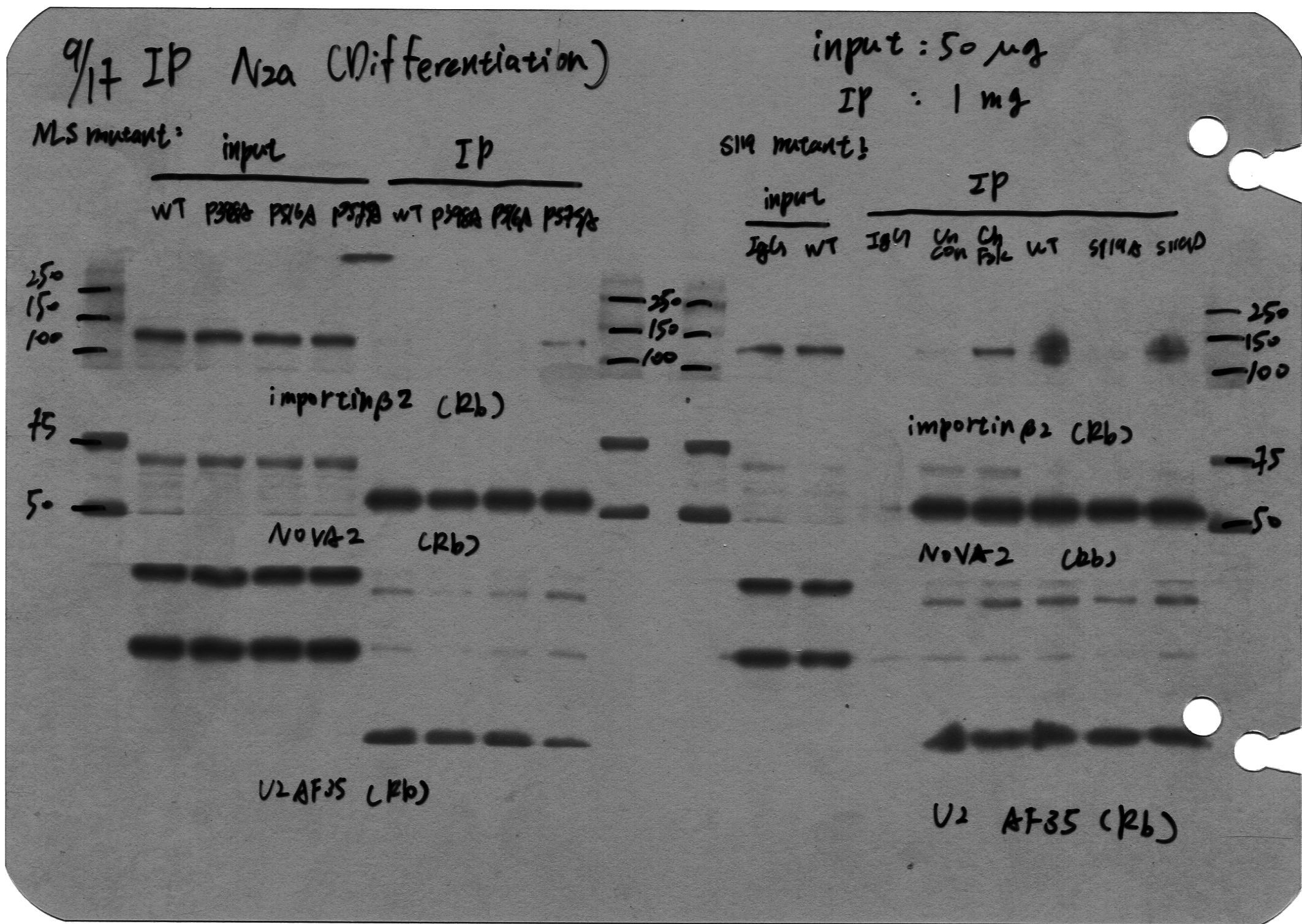

Supplement: Supplementary file 5 — Source data Fig. 4 [file 44318_2025_560_MOESM5_ESM.zip › Figure4/4G/N2a_Differentiation_IP-FLAG western_importinB_NOVA2_U2AF1-5.tif]

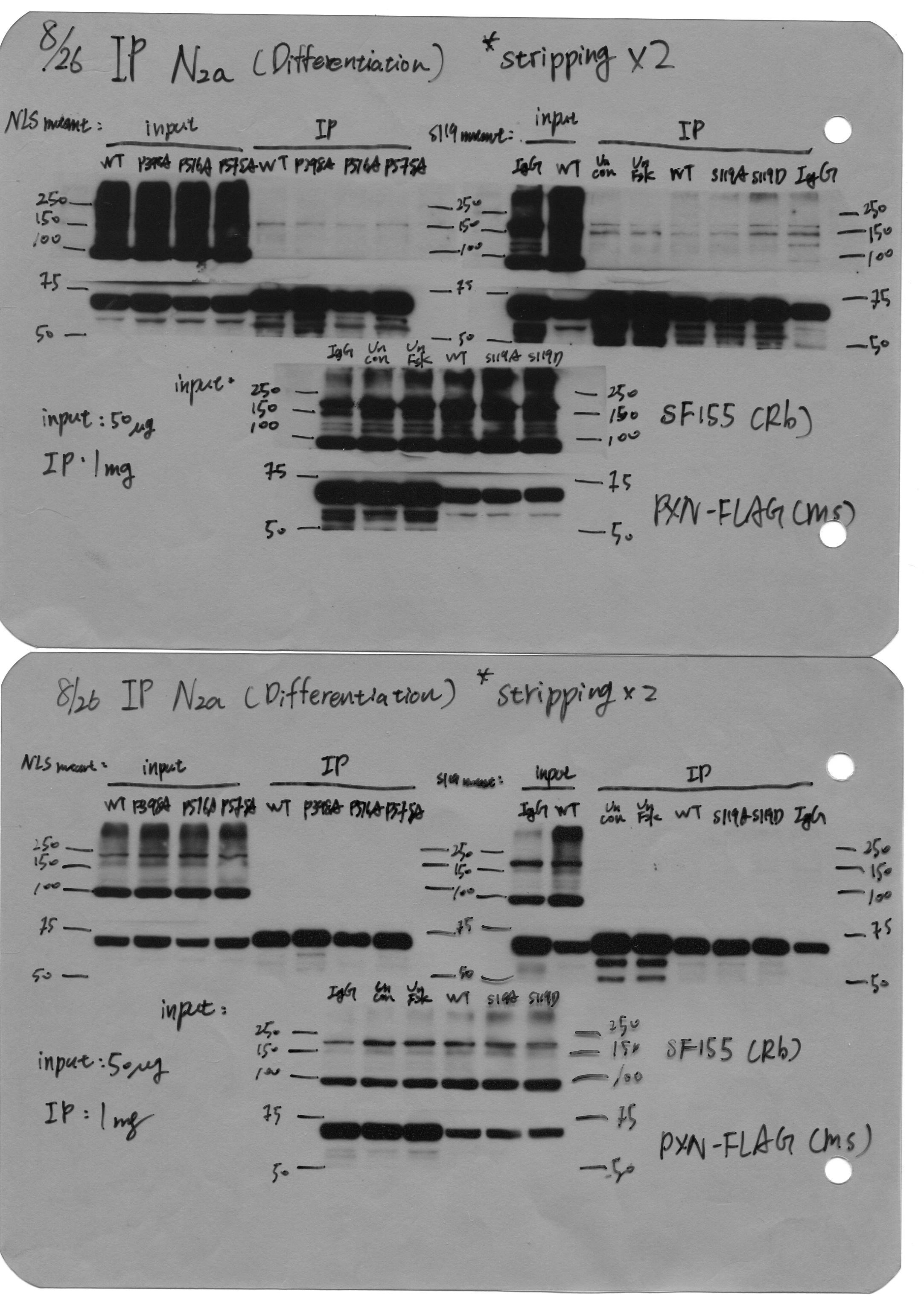

Supplement: Supplementary file 5 — Source data Fig. 4 [file 44318_2025_560_MOESM5_ESM.zip › Figure4/4G/N2a_Differentiation_IP-FLAG western_SF3b155_FLAG-1.tif]

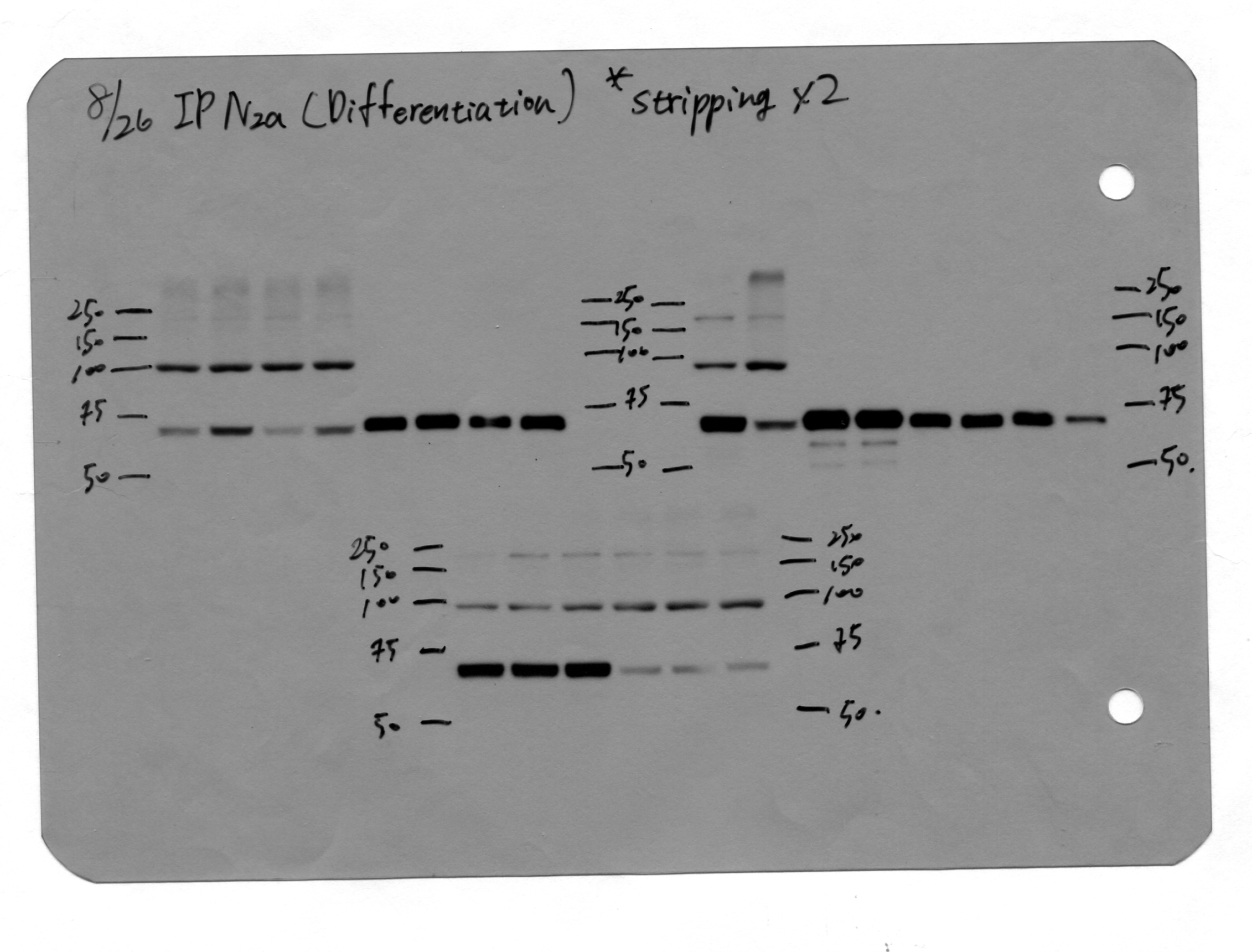

Supplement: Supplementary file 5 — Source data Fig. 4 [file 44318_2025_560_MOESM5_ESM.zip › Figure4/4G/N2a_Differentiation_IP-FLAG western_SF3b155_FLAG-2.tif]

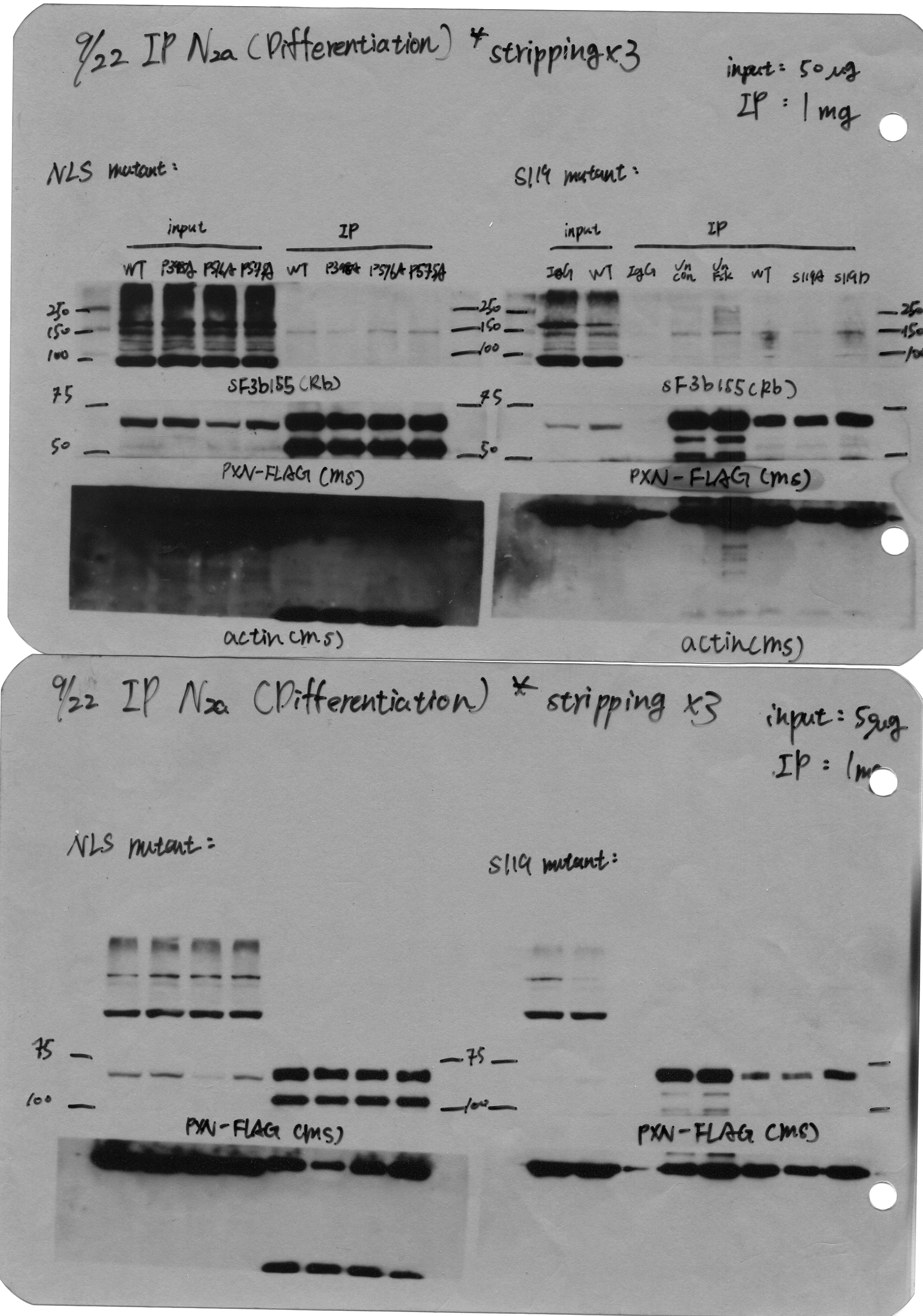

Supplement: Supplementary file 5 — Source data Fig. 4 [file 44318_2025_560_MOESM5_ESM.zip › Figure4/4G/N2a_Differentiation_IP-FLAG western_SF3b155_FLAG_Actin-1.tif]

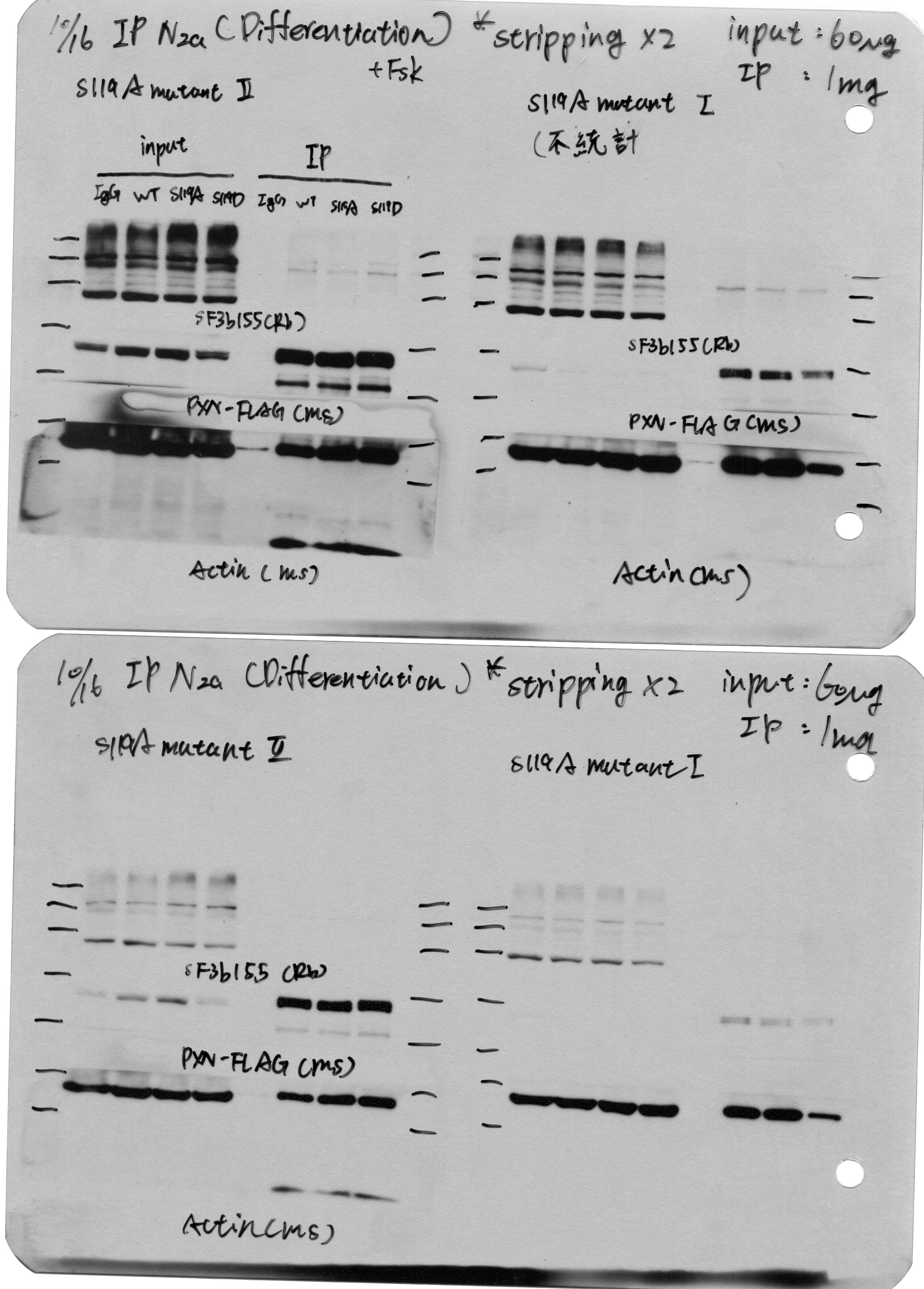

Supplement: Supplementary file 5 — Source data Fig. 4 [file 44318_2025_560_MOESM5_ESM.zip › Figure4/4G/N2a_Differentiation_IP-FLAG western_SF3b155_FLAG_Actin-2.tif]

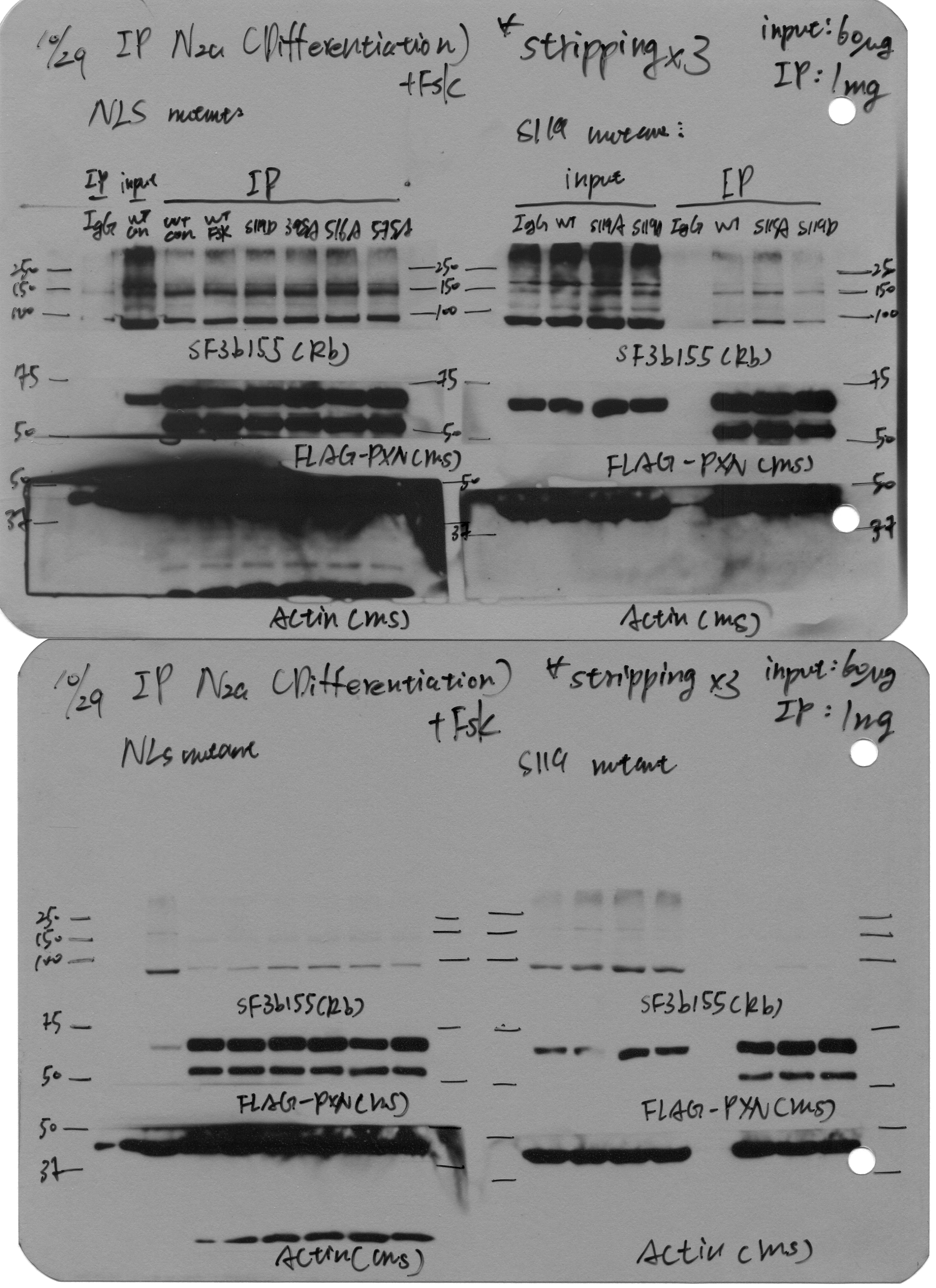

Supplement: Supplementary file 5 — Source data Fig. 4 [file 44318_2025_560_MOESM5_ESM.zip › Figure4/4G/N2a_Differentiation_IP-FLAG western_SF3b155_FLAG_Actin-3.tif]

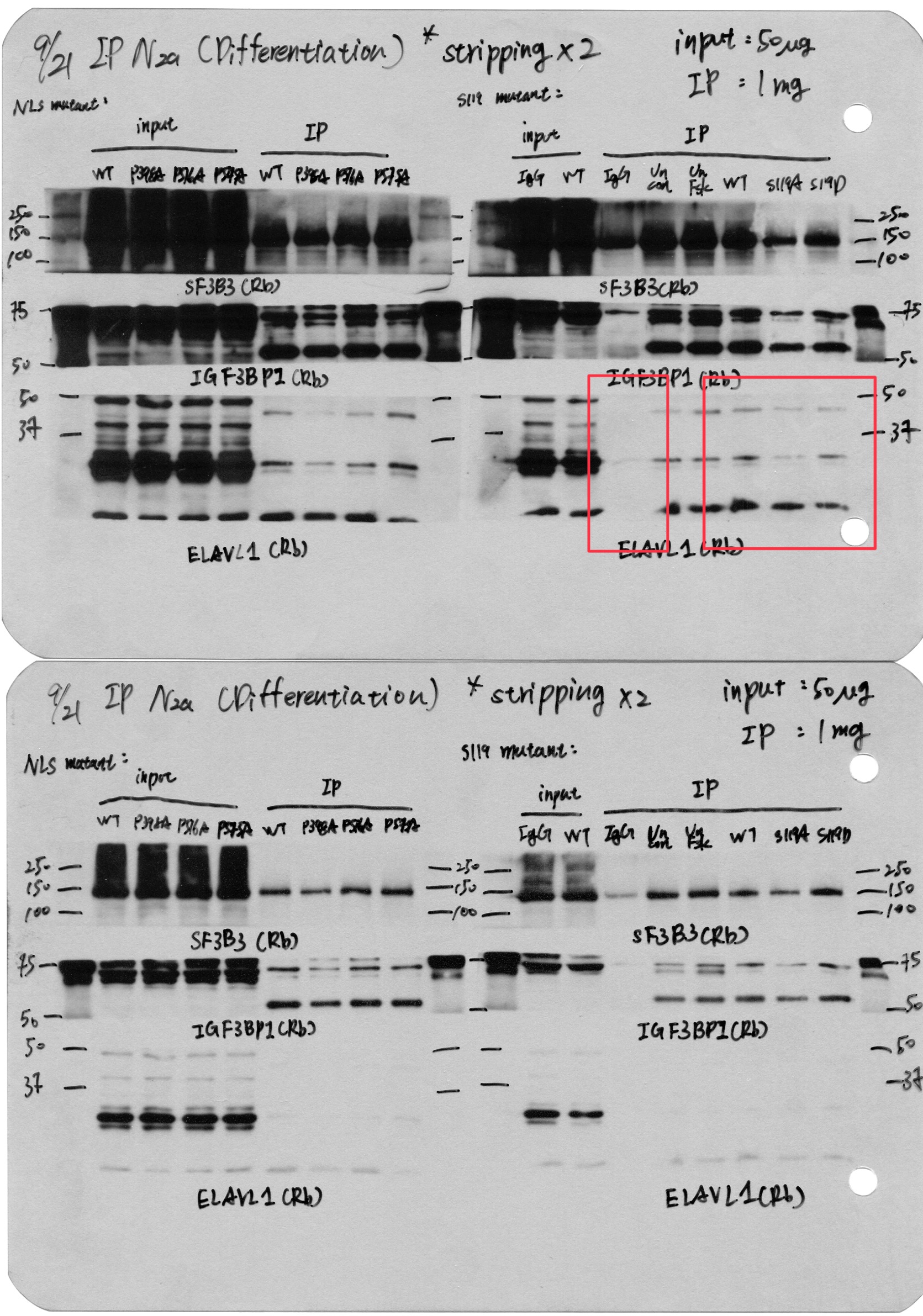

Supplement: Supplementary file 5 — Source data Fig. 4 [file 44318_2025_560_MOESM5_ESM.zip › Figure4/4G/N2a_Differentiation_IP-FLAG western_SF3B3_IGF2BP1_ELAVL1-1-refine.tiff]

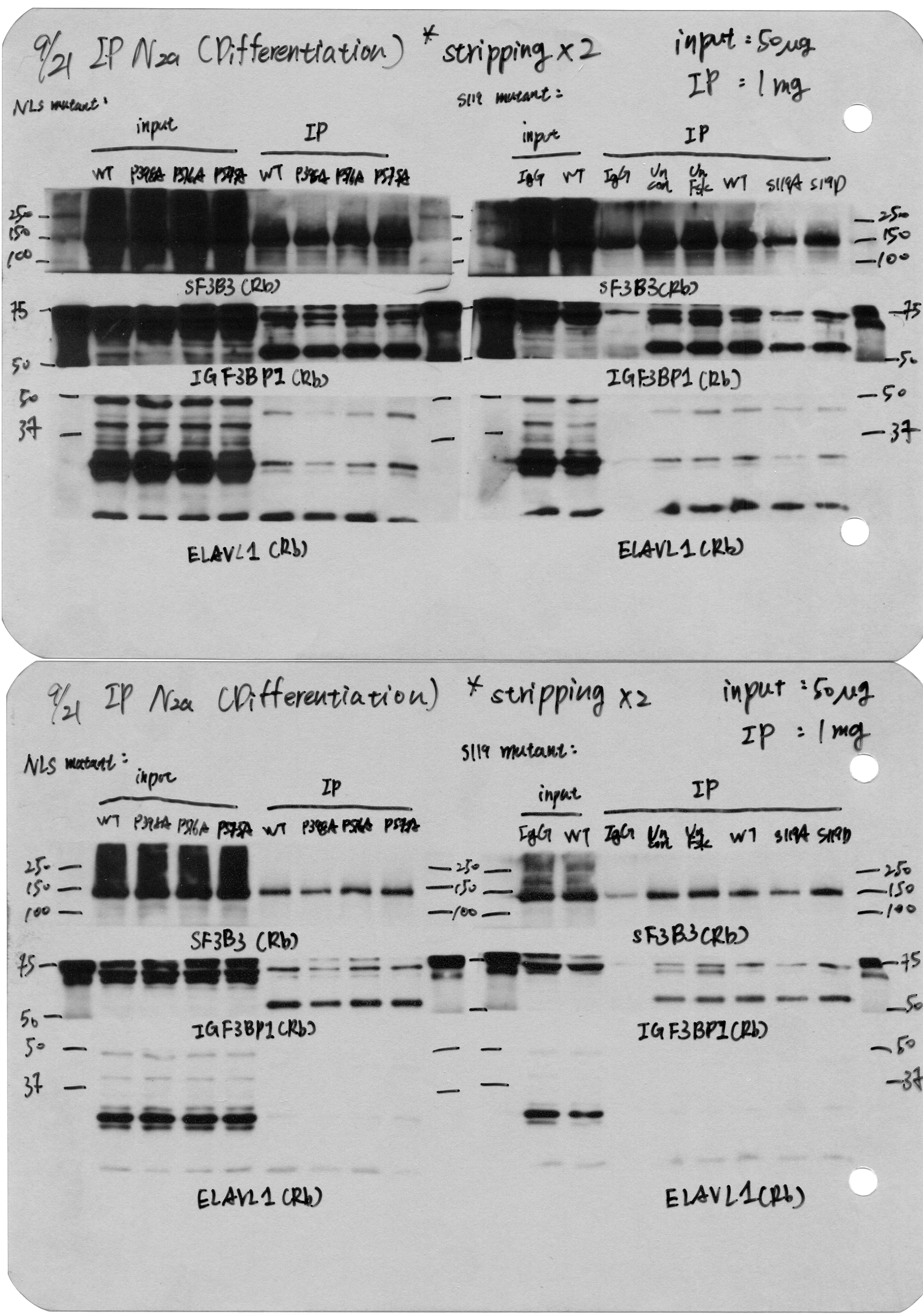

Supplement: Supplementary file 5 — Source data Fig. 4 [file 44318_2025_560_MOESM5_ESM.zip › Figure4/4G/N2a_Differentiation_IP-FLAG western_SF3B3_IGF2BP1_ELAVL1-1.tif]

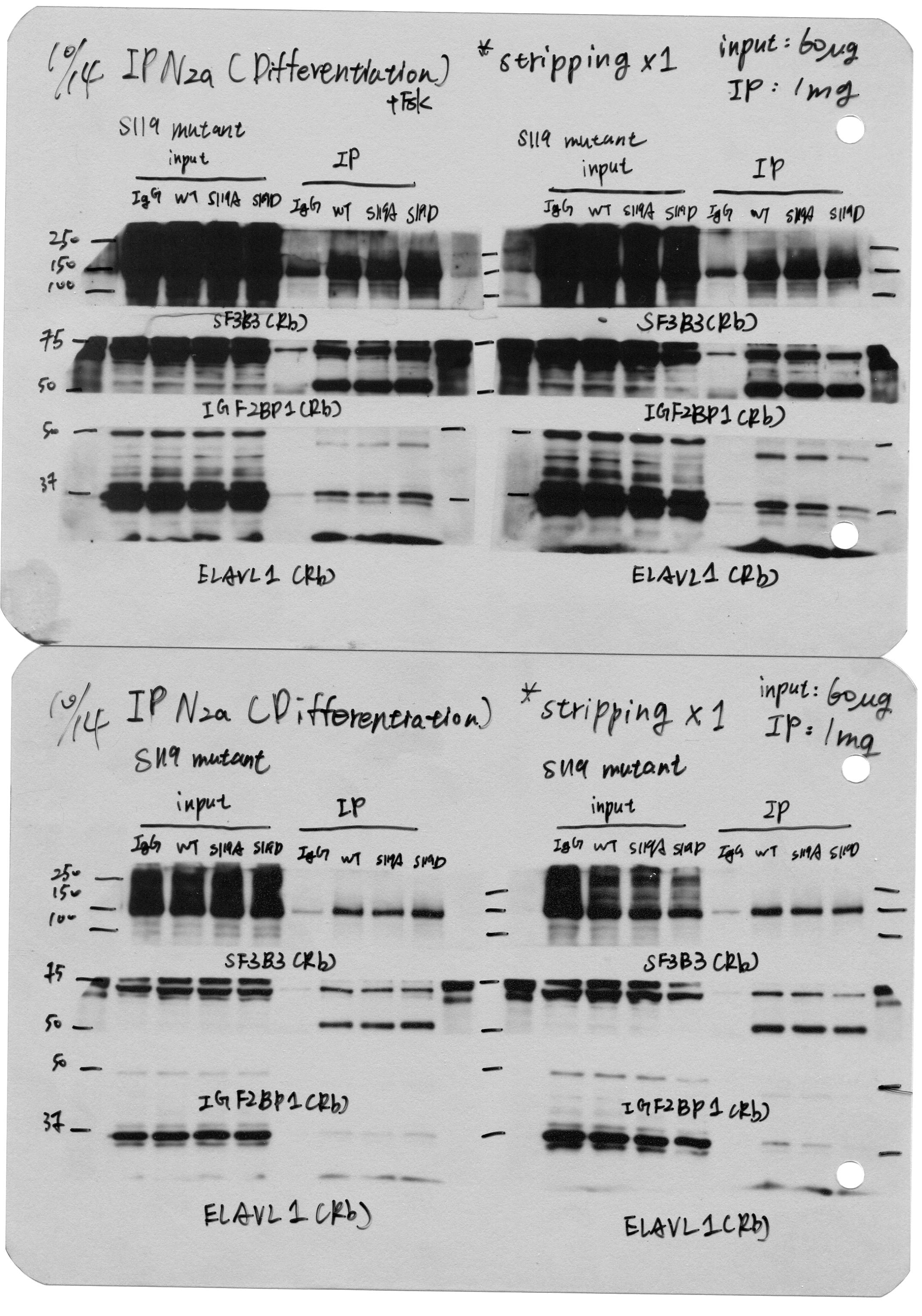

Supplement: Supplementary file 5 — Source data Fig. 4 [file 44318_2025_560_MOESM5_ESM.zip › Figure4/4G/N2a_Differentiation_IP-FLAG western_SF3B3_IGF2BP1_ELAVL1-2.tif]

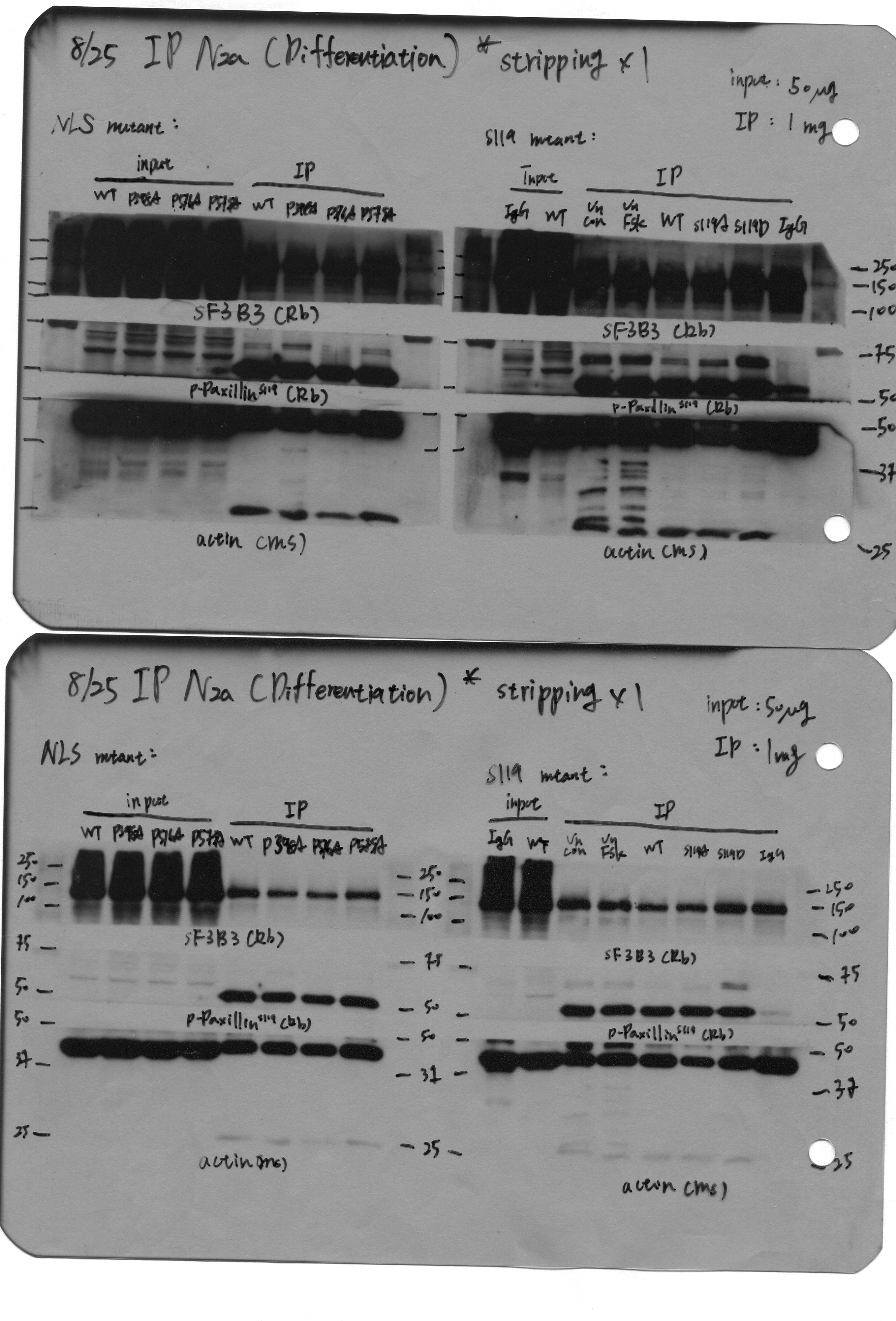

Supplement: Supplementary file 5 — Source data Fig. 4 [file 44318_2025_560_MOESM5_ESM.zip › Figure4/4G/N2a_Differentiation_IP-FLAG western_SF3B3_p-PaxillinS119_Actin-1.tif]

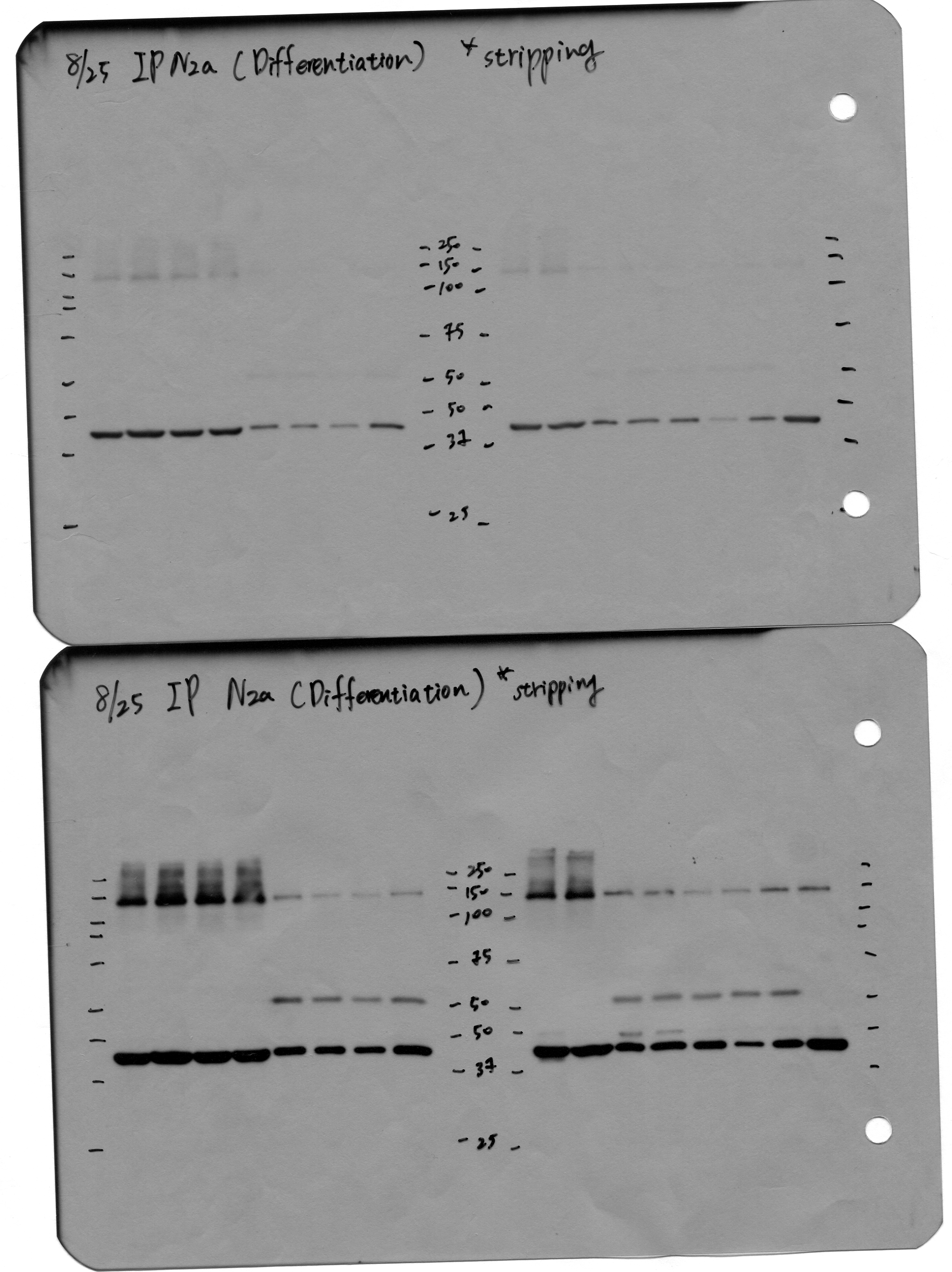

Supplement: Supplementary file 5 — Source data Fig. 4 [file 44318_2025_560_MOESM5_ESM.zip › Figure4/4G/N2a_Differentiation_IP-FLAG western_SF3B3_p-PaxillinS119_Actin-2.tif]

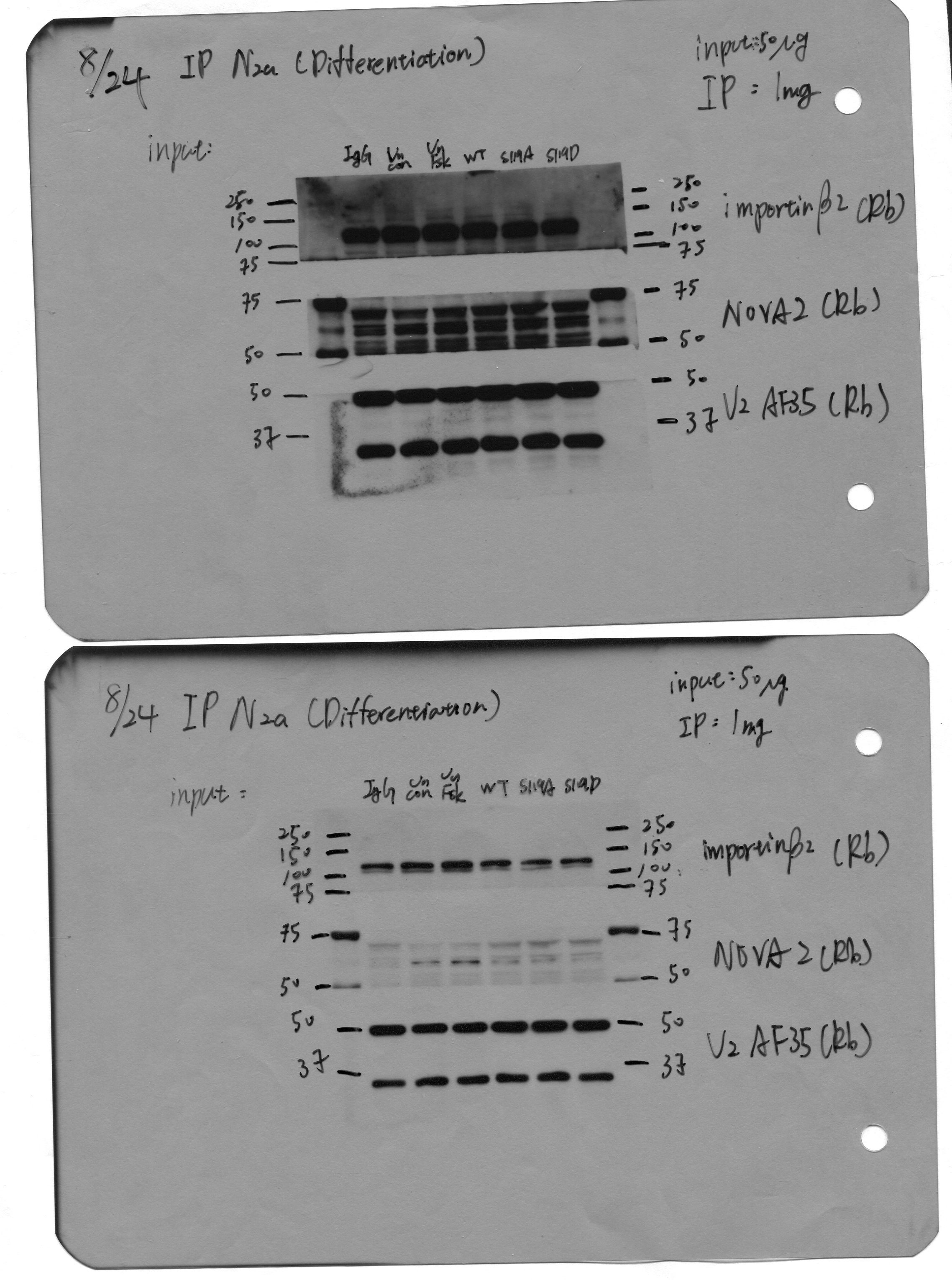

Supplement: Supplementary file 5 — Source data Fig. 4 [file 44318_2025_560_MOESM5_ESM.zip › Figure4/4G/N2a_Differentiation_western_importinB2_NOVA2_U2AF1-1.tif]

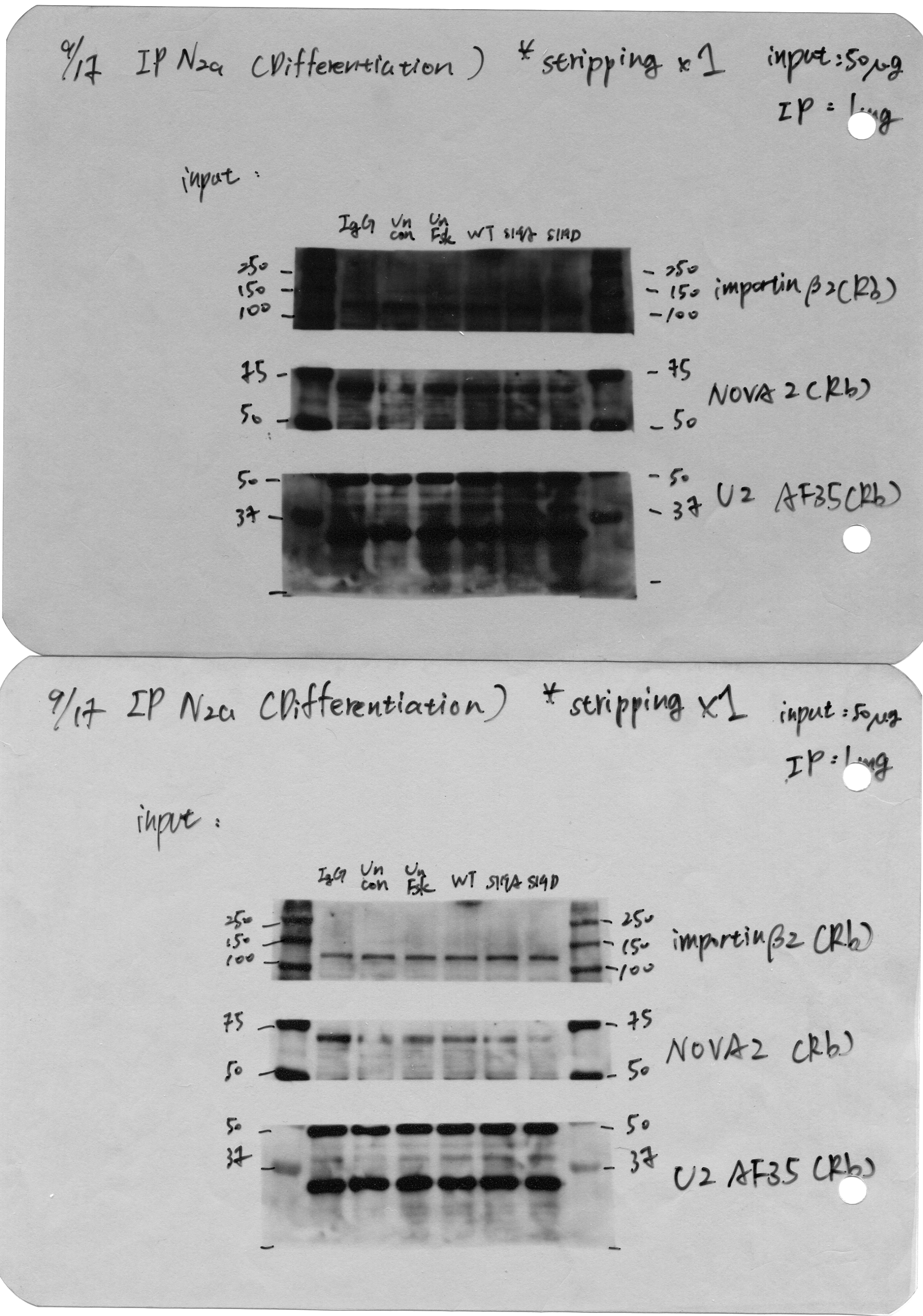

Supplement: Supplementary file 5 — Source data Fig. 4 [file 44318_2025_560_MOESM5_ESM.zip › Figure4/4G/N2a_Differentiation_western_importinB_NOVA2_U2AF1-2.tif]

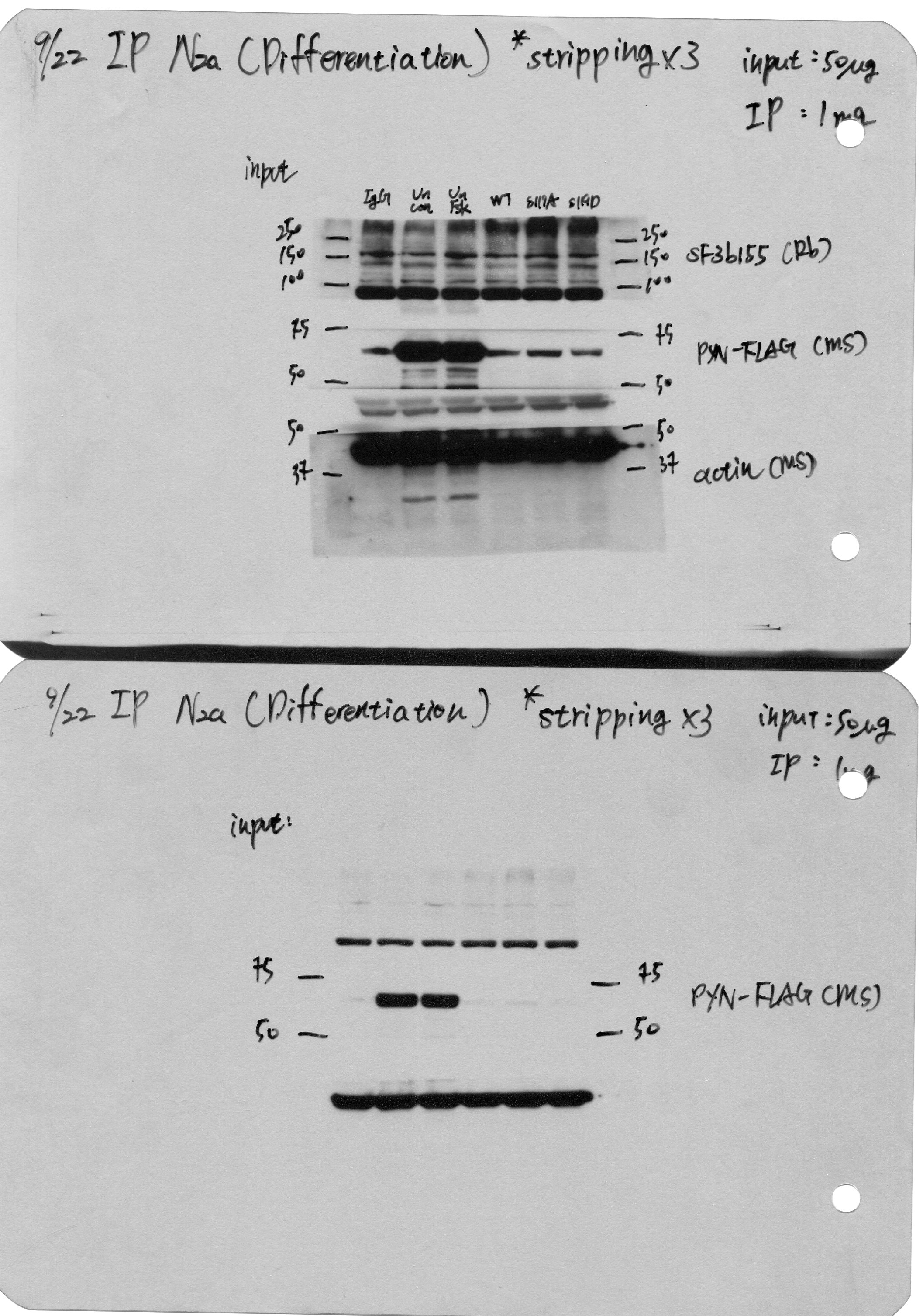

Supplement: Supplementary file 5 — Source data Fig. 4 [file 44318_2025_560_MOESM5_ESM.zip › Figure4/4G/N2a_Differentiation_western_SF3b155_FLAG_Actin.tif]

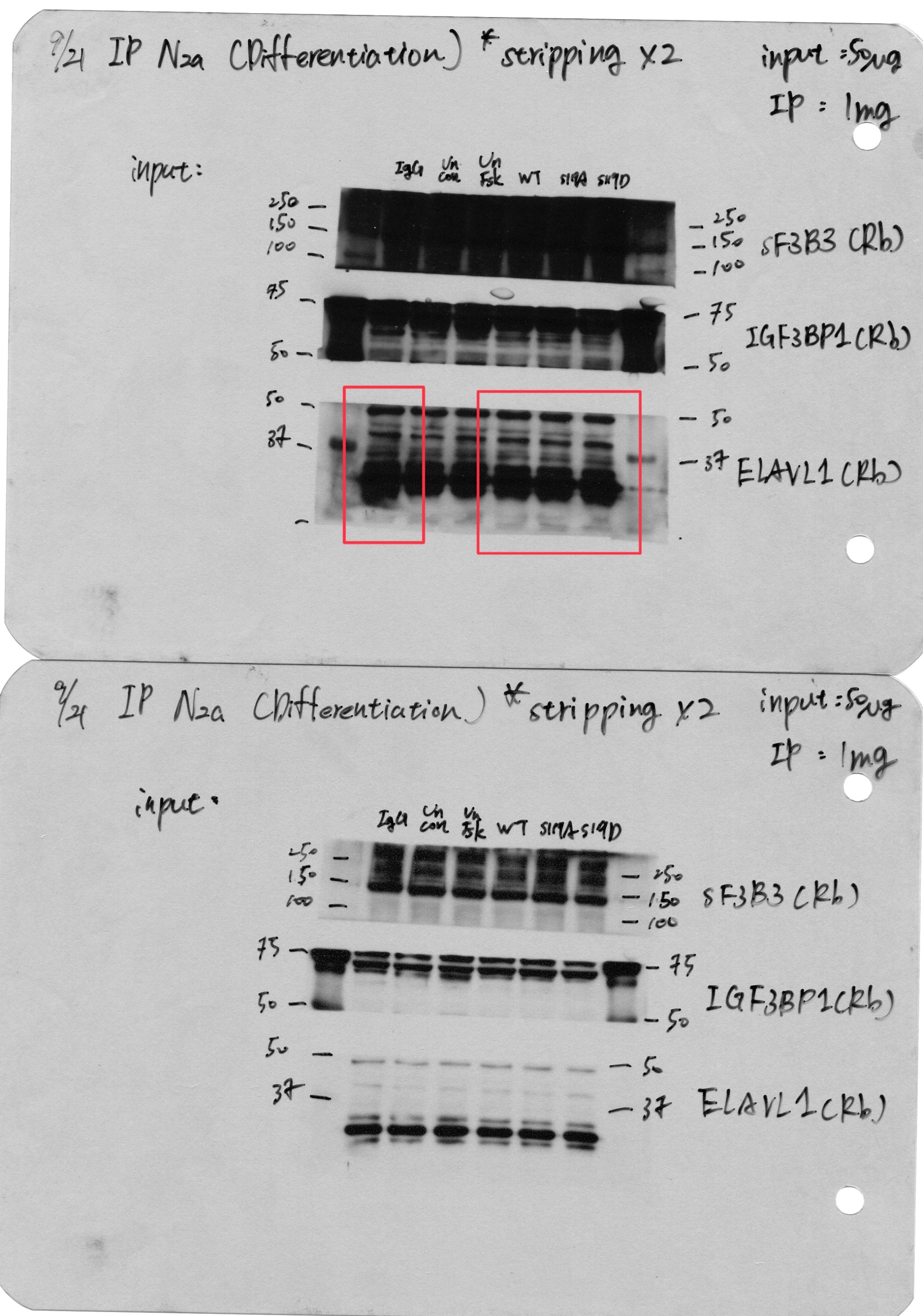

Supplement: Supplementary file 5 — Source data Fig. 4 [file 44318_2025_560_MOESM5_ESM.zip › Figure4/4G/N2a_Differentiation_western_SF3B3_IGF2BP1_ELAVL1-refine.tiff]

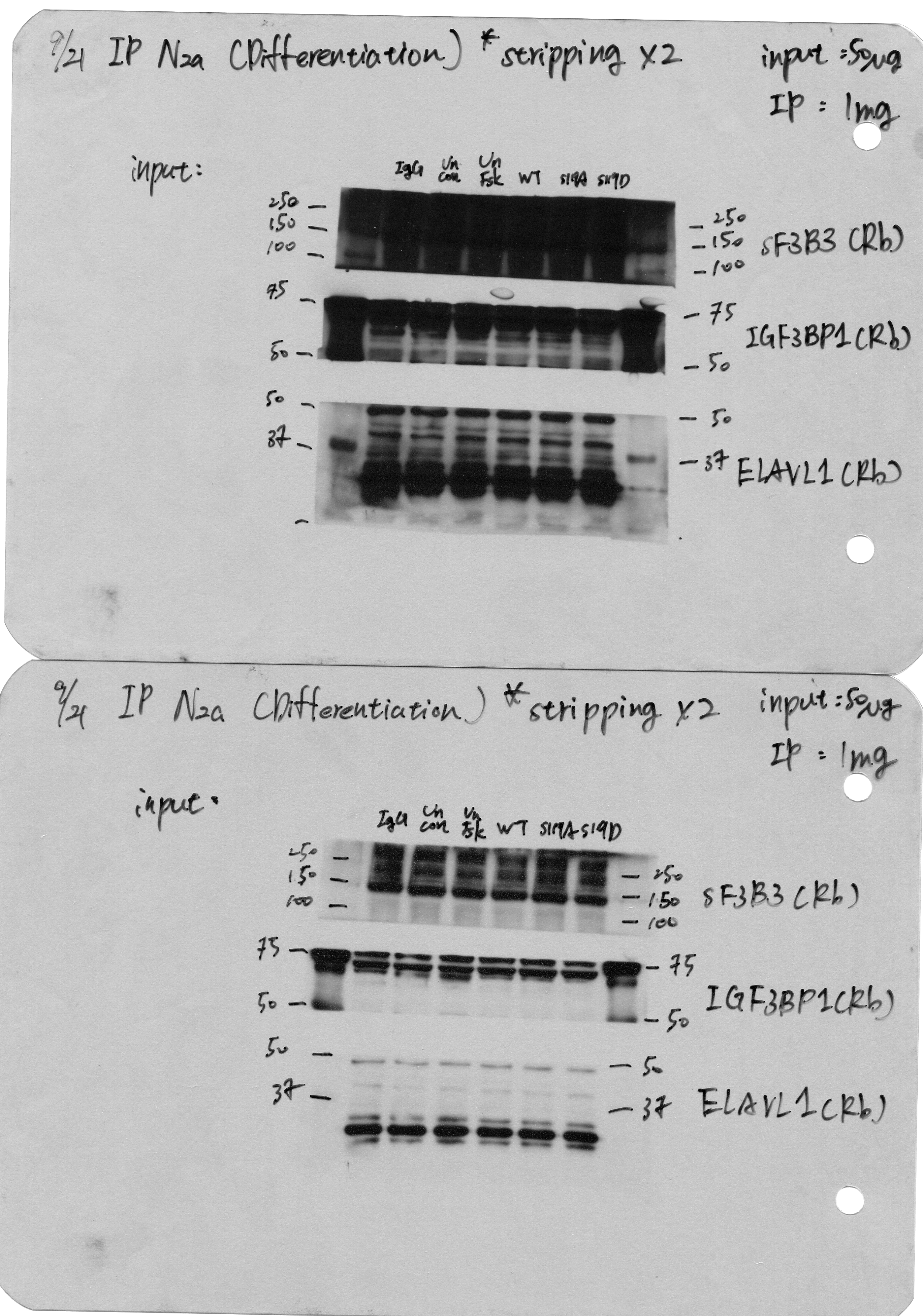

Supplement: Supplementary file 5 — Source data Fig. 4 [file 44318_2025_560_MOESM5_ESM.zip › Figure4/4G/N2a_Differentiation_western_SF3B3_IGF2BP1_ELAVL1.tif]

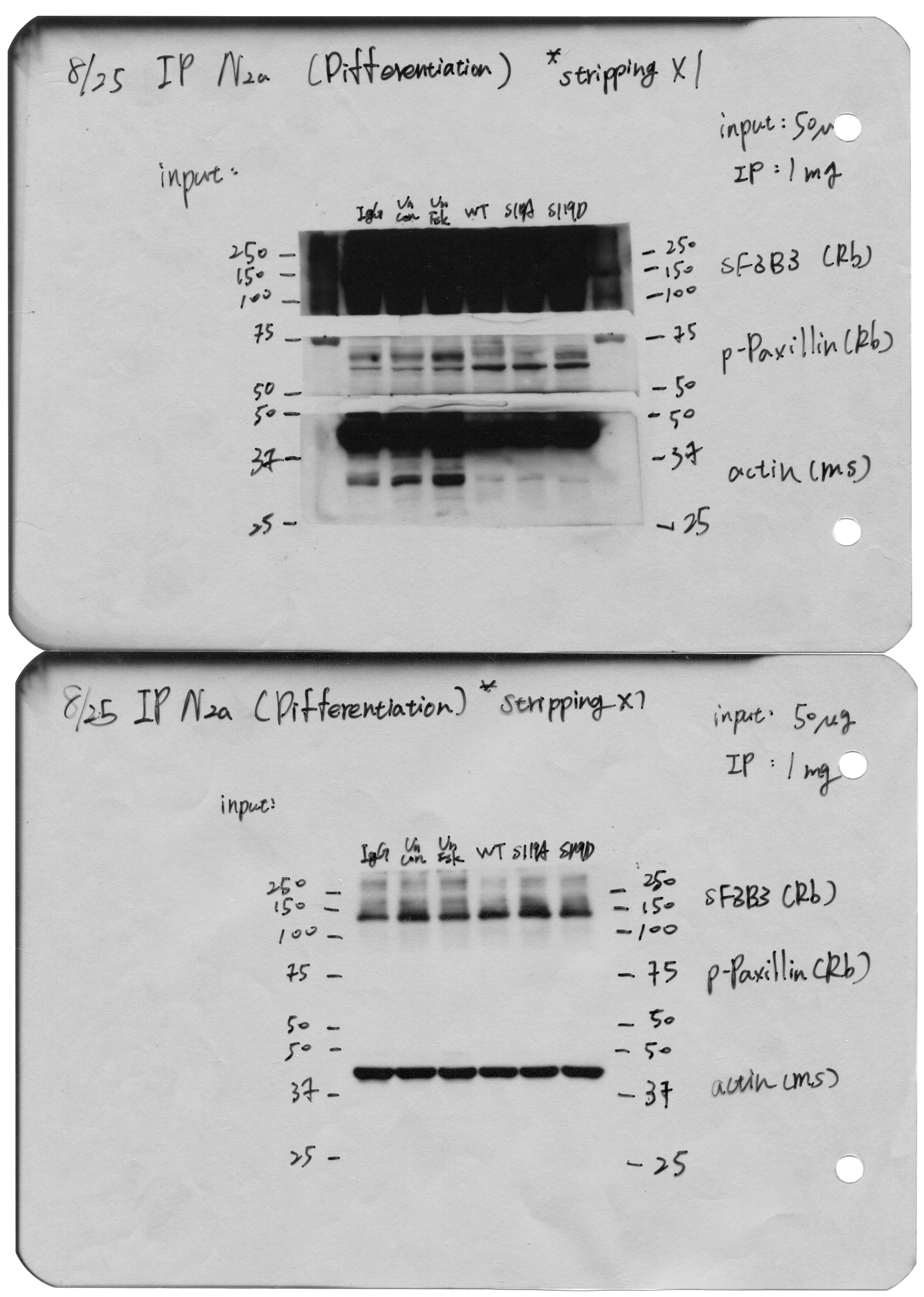

Supplement: Supplementary file 5 — Source data Fig. 4 [file 44318_2025_560_MOESM5_ESM.zip › Figure4/4G/N2a_Differentiation_western_SF3B3_p-PaxillinS119_Actin.tif]

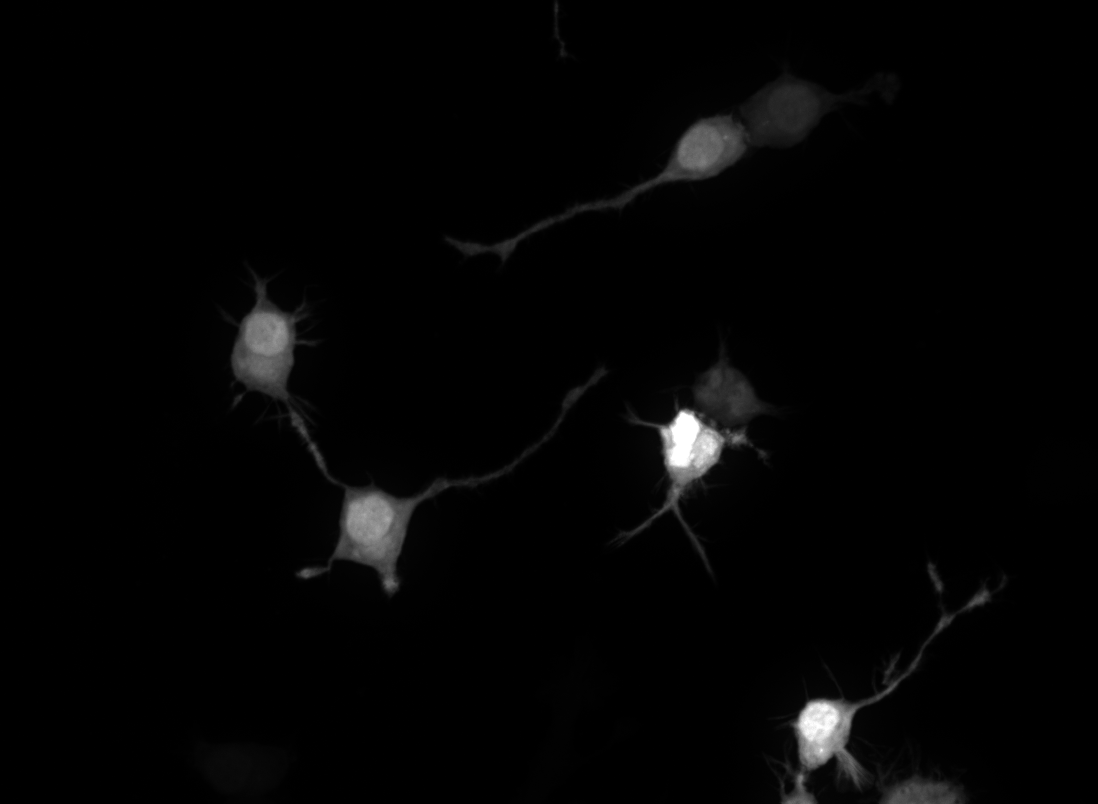

Supplement: Supplementary file 5 — Source data Fig. 4 [file 44318_2025_560_MOESM5_ESM.zip › Figure4/4H/N2a_Differentiation_PXN-S119A_EGFP.tif]

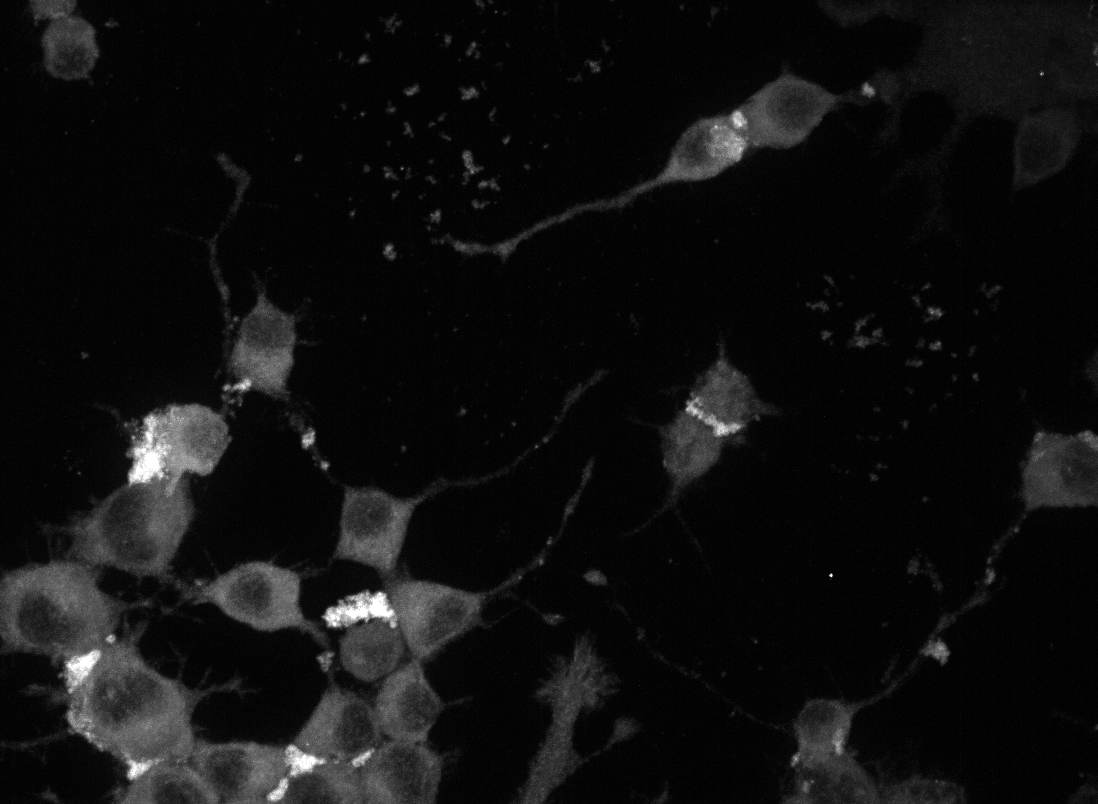

Supplement: Supplementary file 5 — Source data Fig. 4 [file 44318_2025_560_MOESM5_ESM.zip › Figure4/4H/N2a_Differentiation_PXN-S119A_mCherry.tif]

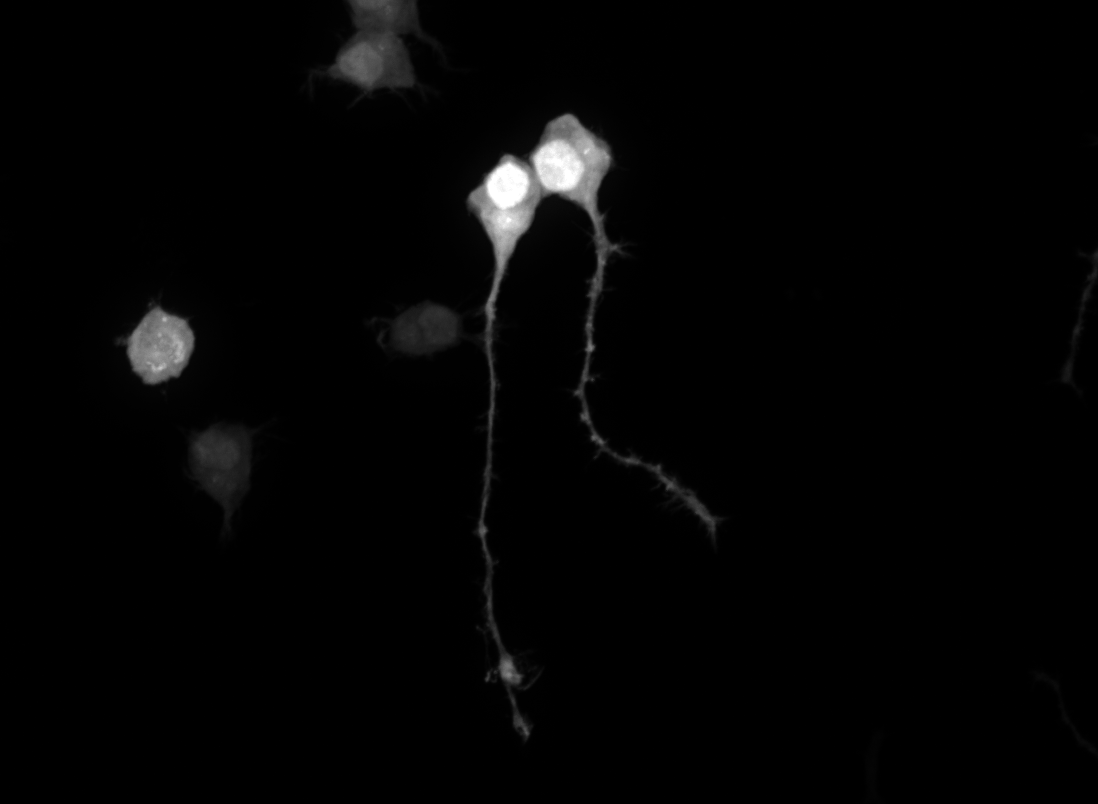

Supplement: Supplementary file 5 — Source data Fig. 4 [file 44318_2025_560_MOESM5_ESM.zip › Figure4/4H/N2a_Differentiation_PXN-S119D_EGFP.tif]

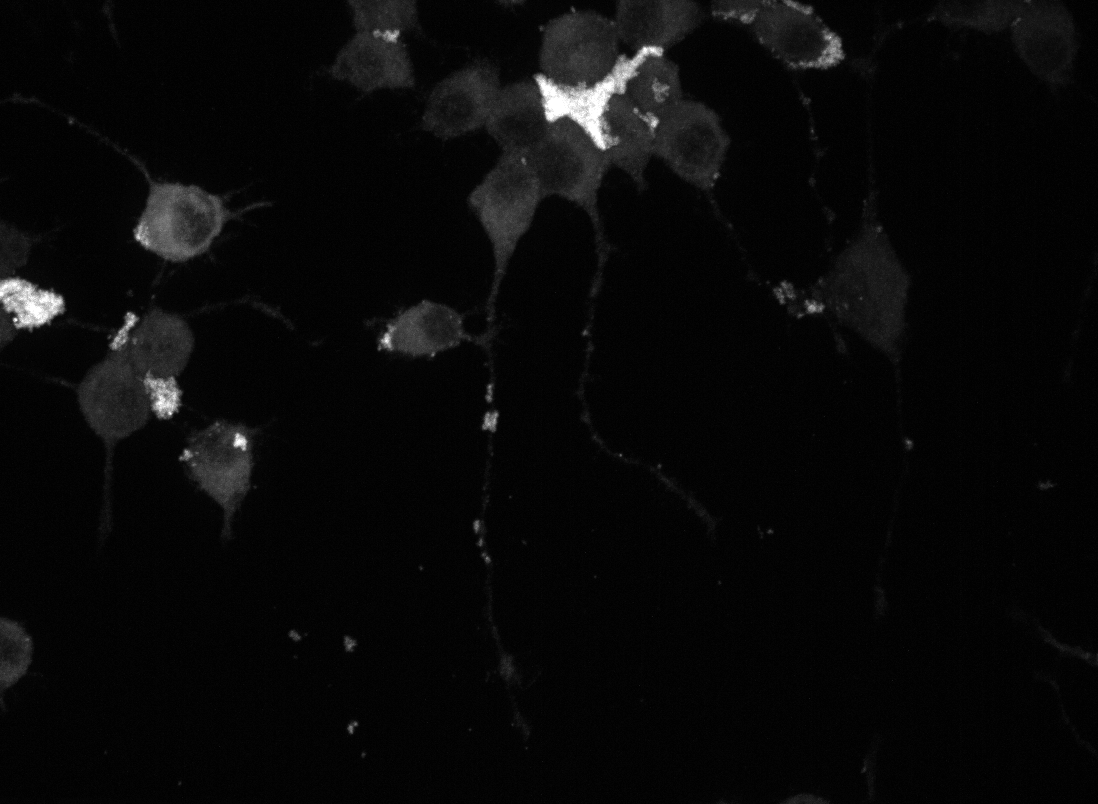

Supplement: Supplementary file 5 — Source data Fig. 4 [file 44318_2025_560_MOESM5_ESM.zip › Figure4/4H/N2a_Differentiation_PXN-S119D_mCherry.tif]

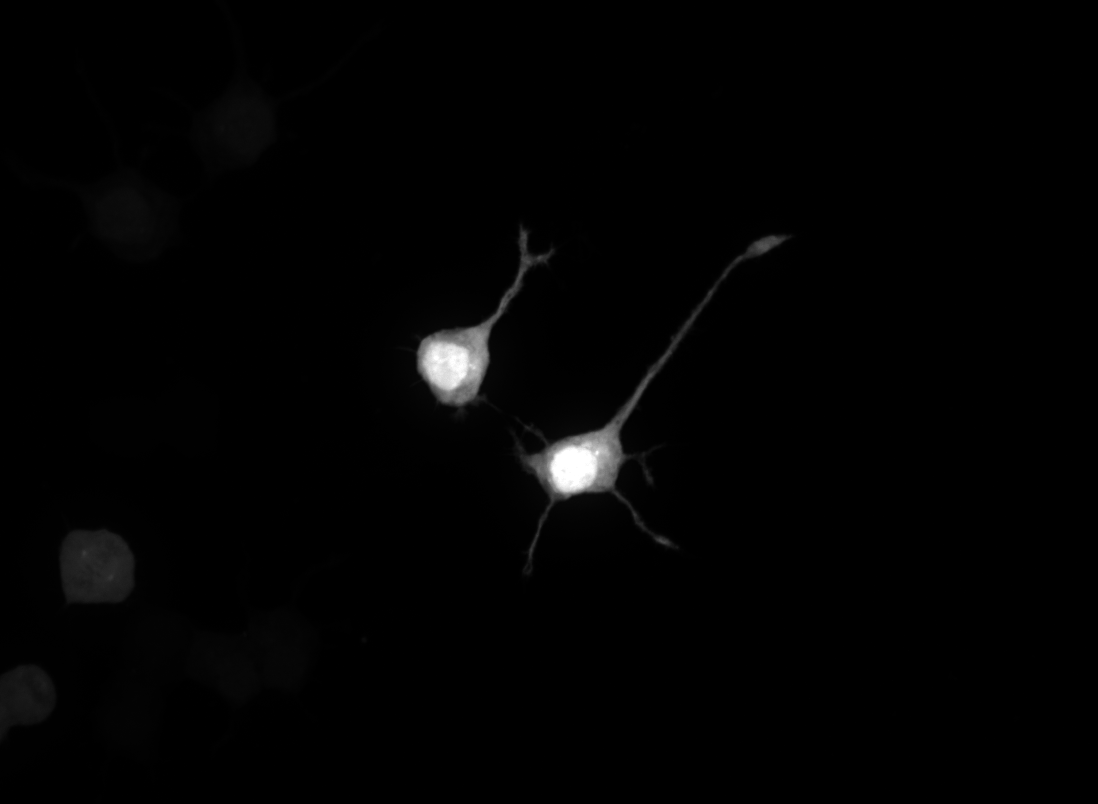

Supplement: Supplementary file 5 — Source data Fig. 4 [file 44318_2025_560_MOESM5_ESM.zip › Figure4/4H/N2a_Differentiation_PXN-WT_EGFP.tif]

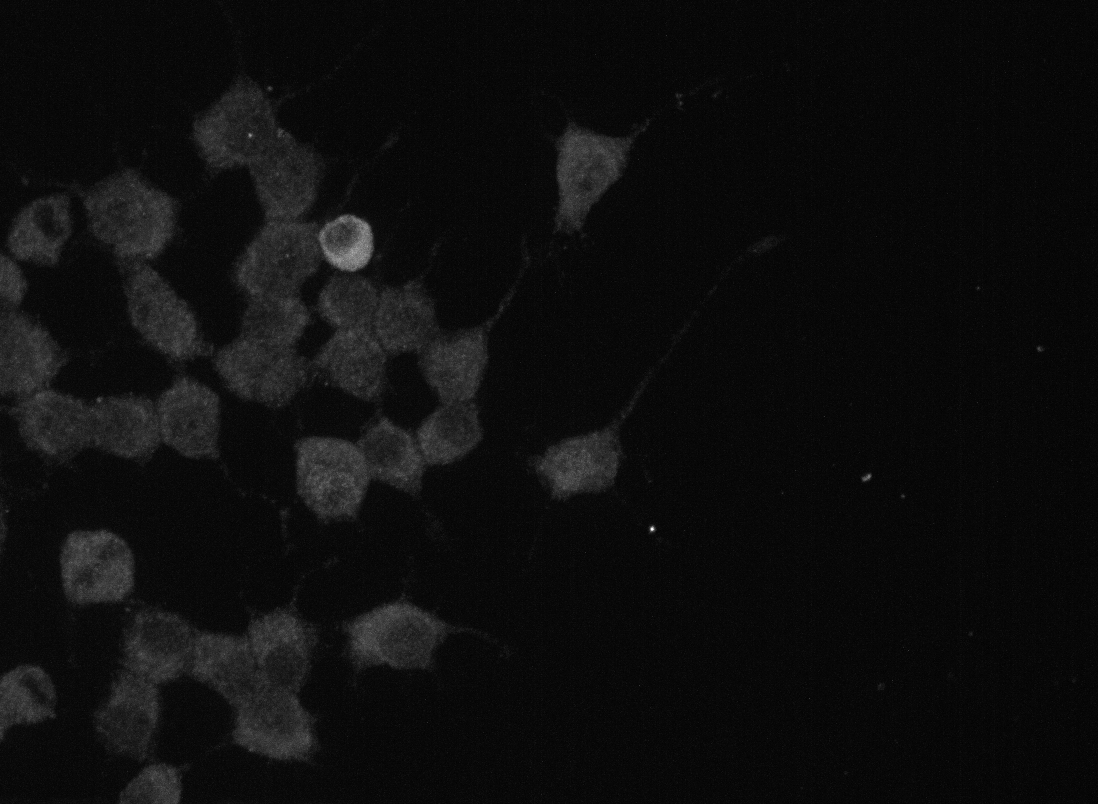

Supplement: Supplementary file 5 — Source data Fig. 4 [file 44318_2025_560_MOESM5_ESM.zip › Figure4/4H/N2a_Differentiation_PXN-WT_mCherry.tif]
