# Supplementary material for: Nuclear paxillin functions as a molecular switch for alternative splicing in neurons during a critical period of brain development
Source: EMBO J. 2025 Sep 9;44(21):5965–92. doi: 10.1038/s44318-025-00560-8 (PMC12583701; doi:10.1038/s44318-025-00560-8)
Supplement: Supplementary file 17 — Expanded View Figures [file 44318_2025_560_MOESM17_ESM.pdf]

Expanded View Figures

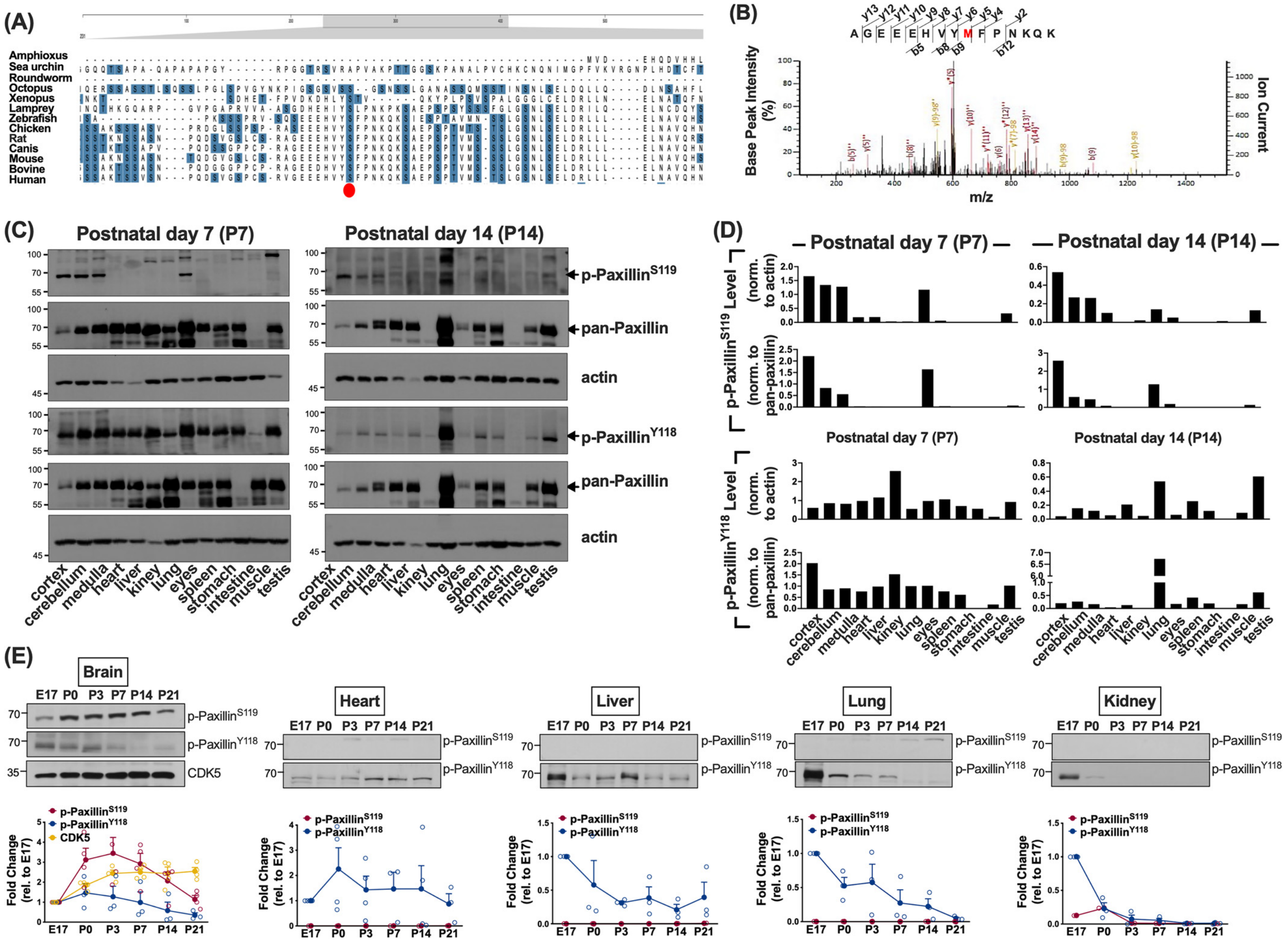

Figure EV1. Identification of a brain-enriched phosphorylation site at paxillin serine119.

(**A**) Paxillin S119 is phylogenetically conserved. Alignment of sequences found around paxillin S119 (red dot), illustrating conservation across indicated species. (**B**) Mass spectrometric identification of phosphopeptides in paxillin protein IP'd from P14 mouse brain lysates. The phosphopeptide AGEEEHVYMFPNKQK (phospho-paxillin^{S119}; p-Paxillin^{S119}) was identified by precursor ion scanning for neutral loss of phosphoric acid (M-H3PO4). M, peptide precursor ion. (**C**) Western blots show p-Paxillin^{S119} enrichment in mouse brain lysates at P7 and P14. Lysates were prepared from multiple tissues, as indicated. The pan-Paxillin blots shown here were derived from membranes that were first probed with either anti–p-Paxillin^{S119} or anti–p-Paxillin^{Y118}, and after antibody stripping were subsequently re-probed with anti–pan-Paxillin to serve as total Paxillin controls for the same samples. (**D**) Quantification of data shown in blots in (**C**). (**E**) Developmental profiles of p-Paxillin^{S119} and p-Paxillin^{Y118} across multiple tissues. Western blots showing p-Paxillin^{S119} and p-Paxillin^{Y118} levels over various developmental stages in various tissues. Graph shows average protein intensity ± SEM, compared with corresponding levels at E17 ($n = 3$–4 mice per group). Source data are available online for this figure.

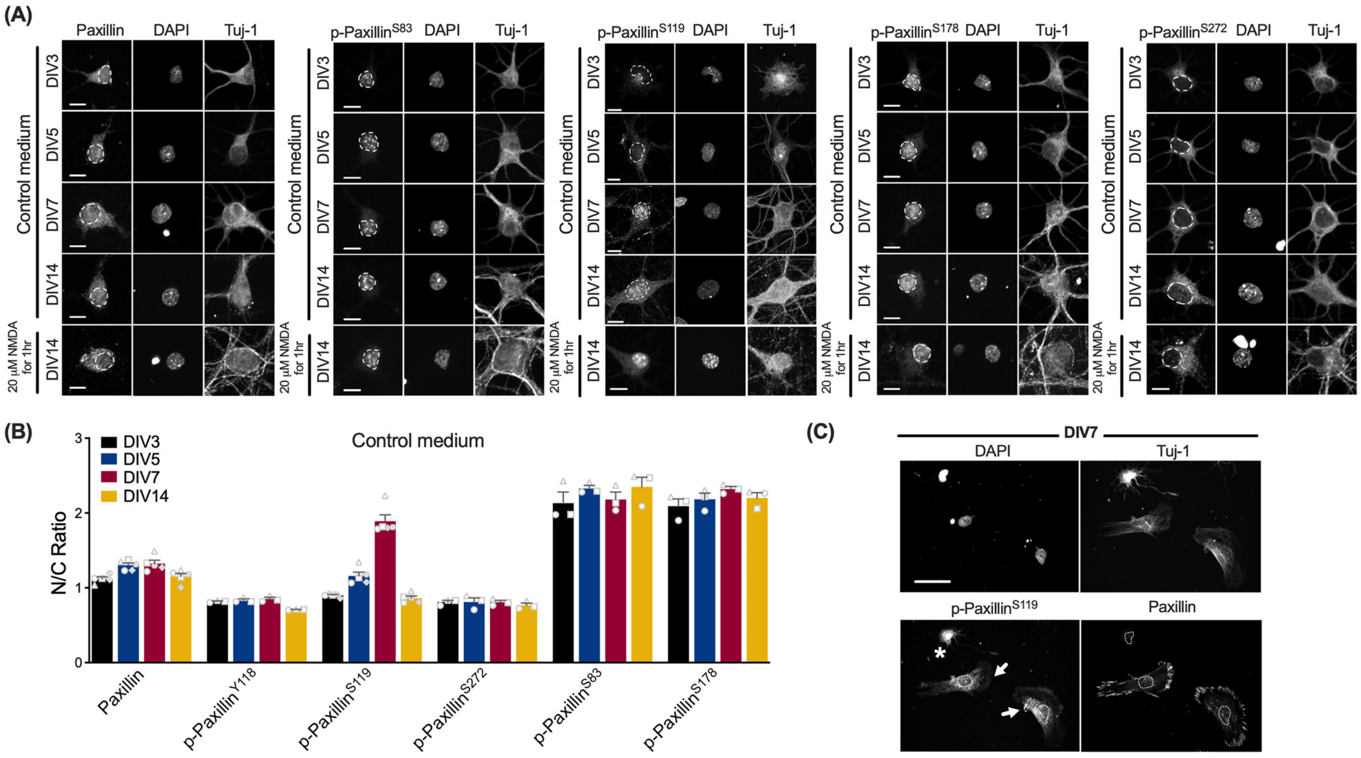

Figure EV2. Site-specific phosphorylation determines paxillin subcellular localization in neurons and glia.

(**A**, **B**) DIV7 neurons show transient increases in nuclear p-Paxillin^{S119} levels. (**A**) Representative images of rat primary neuronal cultures stained with antibodies against different phosphorylated forms of paxillin at the indicated time points. Scale bar, 5 μm. (**B**) Histograms summarizing the nucleus-to-cytoplasm (N/C) ratios of various phosphorylated paxillin forms from experiments similar to those in (**A**). Data represent mean ± SEM ($n = 3$–5 independent cultures; >6 cells per group). (**C**) Differential distribution of p-Paxillin^{S119} in neurons vs. glia. Representative image of a DIV7 primary neuronal culture showing p-Paxillin^{S119} signals in a neuron (asterisk) versus in neuroglia (arrows). Scale bar, 10 μm.

 © The Author(s)

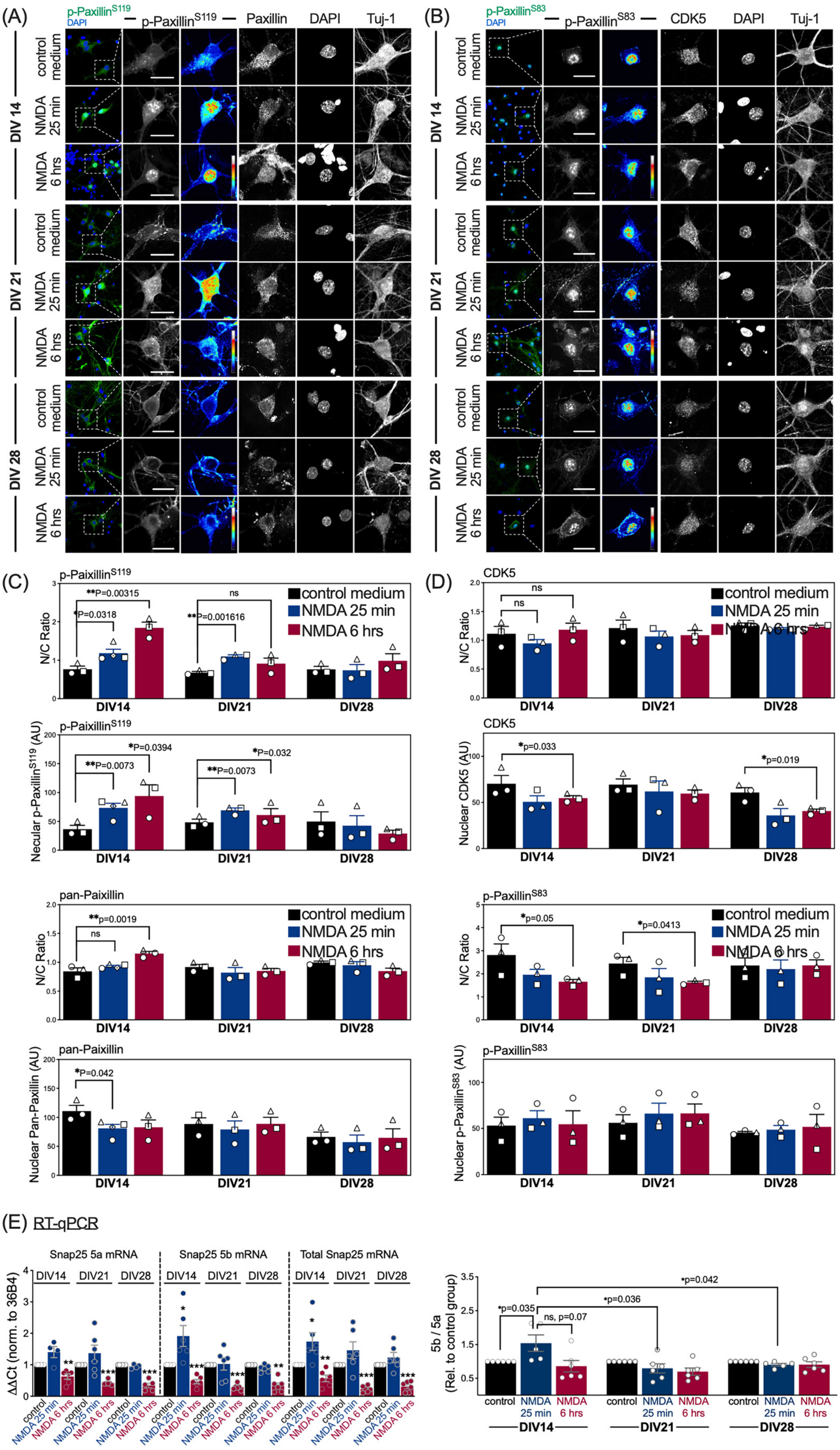
(A)
p-Paxillin S119 DAPI
p-Paxillin S119
Paxillin
DAPI
Tuj-1
(B)
p-Paxillin S83 DAPI
p-Paxillin S83
CDK5
DAPI
Tuj-1
DIV 14
DIV 21
DIV 28
control medium
NMDA 25 min
NMDA 6 hrs
(C)
p-Paixillin S119
N/C Ratio
**P=0.00315
*P=0.0318
**P=0.001616
ns
control medium
NMDA 25 min
NMDA 6 hrs
DIV14
DIV21
DIV28
Necular p-Paxillin S119 (AU)
*P=0.0394
**P=0.0073
*P=0.032
**P=0.0073
pan-Paixillin
**p=0.0019
Nuclear Pan-Paxillin (AU)
*P=0.042
(D)
CDK5
Nuclear CDK5 (AU)
*p=0.033
*p=0.019
p-Paxillin S83
*p=0.05
*p=0.0413
Nuclear p-Paxillin S83 (AU)
(E) RT-qPCR
Snap25 5a mRNA
Snap25 5b mRNA
Total Snap25 mRNA
ΔΔCt (norm. to 36B4)
5b / 5a (Rel. to control group)
*p=0.042
*p=0.036
*p=0.035
ns, p=0.07
control
NMDA 25 min
NMDA 6 hrs

Figure EV3. Both NMDA-induced nucleocytoplasmic translocation of p-Paxillin^{S119} and Snap25 exon 5b-to-5a inclusion in neurons decrease after DIV14.

(**A–D**) Subcellular distribution of endogenous p-Paxillin^{S119}, pan-Paxillin, p-Paxillin^{S83}, and CDK5 in primary cortical neurons at the indicated days in vitro (DIV), following acute ("25 min") or prolonged ("6 h") stimulation with 20 µM NMDA. (**A**, **B**) Representative images of neurons cultured in standard Neurobasal medium under the indicated conditions. Cells were immunostained for p-Paxillin^{S119} (**A**, green), pan-Paxillin, p-Paxillin^{S83} (**B**, green), CDK5 and the neuronal marker Tuj-1, with DAPI counterstain (blue) at the indicated conditions and time points. Scale bar, 15 µm. The intensity of p-Paxillin^{S119} is also displayed using a "royal" look-up table, highlighting the range from low (blue) through medium (green) to high (red) intensity. (**C**, **D**) Histogram summarizing nuclear to cytoplasmic (N/C) ratio and nuclear abundance of p-Paxillin^{S119}, pan-Paxillin, p-Paxillin^{S83}, and CDK5 in primary neuronal cultures at indicated conditions and time points. Data represent mean ± SEM ($n = 3$ independent cultures; >30 cells per group; *$p < 0.05$, **$p < 0.01$, ***$p < 0.001$, ns not significant compared to control, by one-way ANOVA with Dunnett's multiple comparisons test). (**E**) Real-time qPCR analysis of Snap25 exon 5a, 5b, and total mRNA expression in neuronal cultures at the indicated time points under control or NMDA treatment conditions. Data represent mean ± SEM ($n = 5$–6 cultures per group; *$p < 0.05$, **$p < 0.01$, ***$p < 0.001$, ns not significant compared to DIV14 NMDA 25 min group, multiple *t*-tests). Source data are available online for this figure.

© The Author(s)

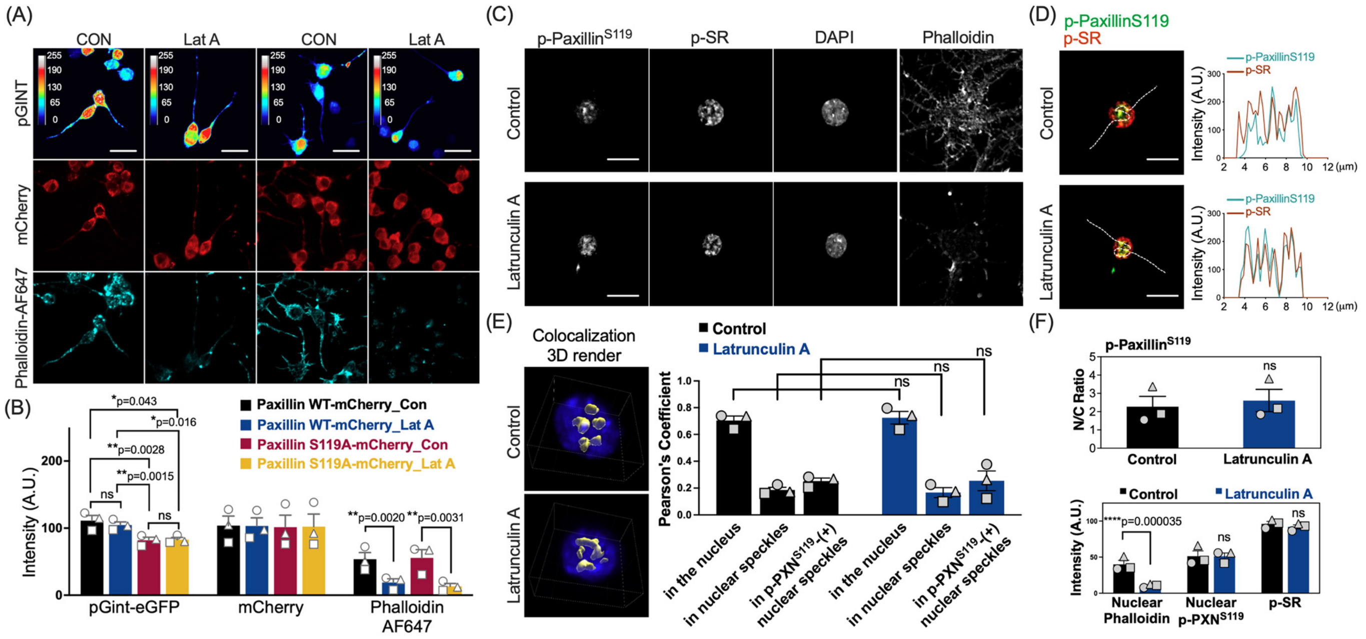

Figure EV4. Disruption of cytoplasmic actin polymerization does not alter mRNA splicing efficiency.

(**A**) Representative images showing differentiated Neuro2A cells transduced with either mCherry-tagged paxillin wild-type (WT) or its S119A variant via lentiviral transduction, followed by transfection with the pGint splicing reporter. Cells were treated with or without the actin polymerization inhibitor Latrunculin A ("Lat A"; 330 nM for 1 h) and then stained with Phalloidin-AF647 to visualize F-actin. eGFP fluorescence intensity is shown using a "royal" look-up table, highlighting the range from low (blue) through medium (green) to high (red) intensity. Scale bar, 20 µm. (**B**) Plot summarizing average eGFP intensity, mCherry signal, and Phalloidin-AF647 staining (±SEM; $n = 21$–30 cells from three independent experiments; ****$p < 0.0001$ compared to WT by Two-way ANOVA with Tukey's multiple comparisons test). (**C–F**) p-Paxillin^{S119} colocalization with phospho-SR proteins (p-SR) is maintained after disruption of actin polymerization. (**C**) Immunofluorescence images of DIV7 neurons treated with or without latrunculin A (330 nM, 1 h), stained for p-Paxillin^{S119}, p-SR, DAPI, and Phalloidin. (**D**) Fluorescence intensity profiles (right panel) across p-Paxillin^{S119} puncta show colocalization with p-SR. Scale bar, 10 µm. (**E**) Left: 3D renderings showing colocalization of p-Paxillin^{S119} with p-SR in nuclear speckles. Right: Bar graphs quantify Pearson's correlation coefficients (±SEM; $n = 3$ cultures; 15 cells per condition; ns not significant, by multiple unpaired t-tests) for p-Paxillin^{S119} and p-SR colocalization within the nucleoplasm, nuclear speckles, and p-Paxillin^{S119}-positive nuclear speckles. (**F**) Quantification of the N/C ratio of p-Paxillin^{S119} (top), and nuclear intensity of p-Paxillin^{S119}, p-SR, and Phalloidin staining (bottom) in DIV7 neurons with or without Lat A treatment. Data represent mean (±SEM; $n = 3$ independent cultures; >30 cells per group; ****$p < 0.0001$; ns not significant, by multiple unpaired t-tests). Source data are available online for this figure.

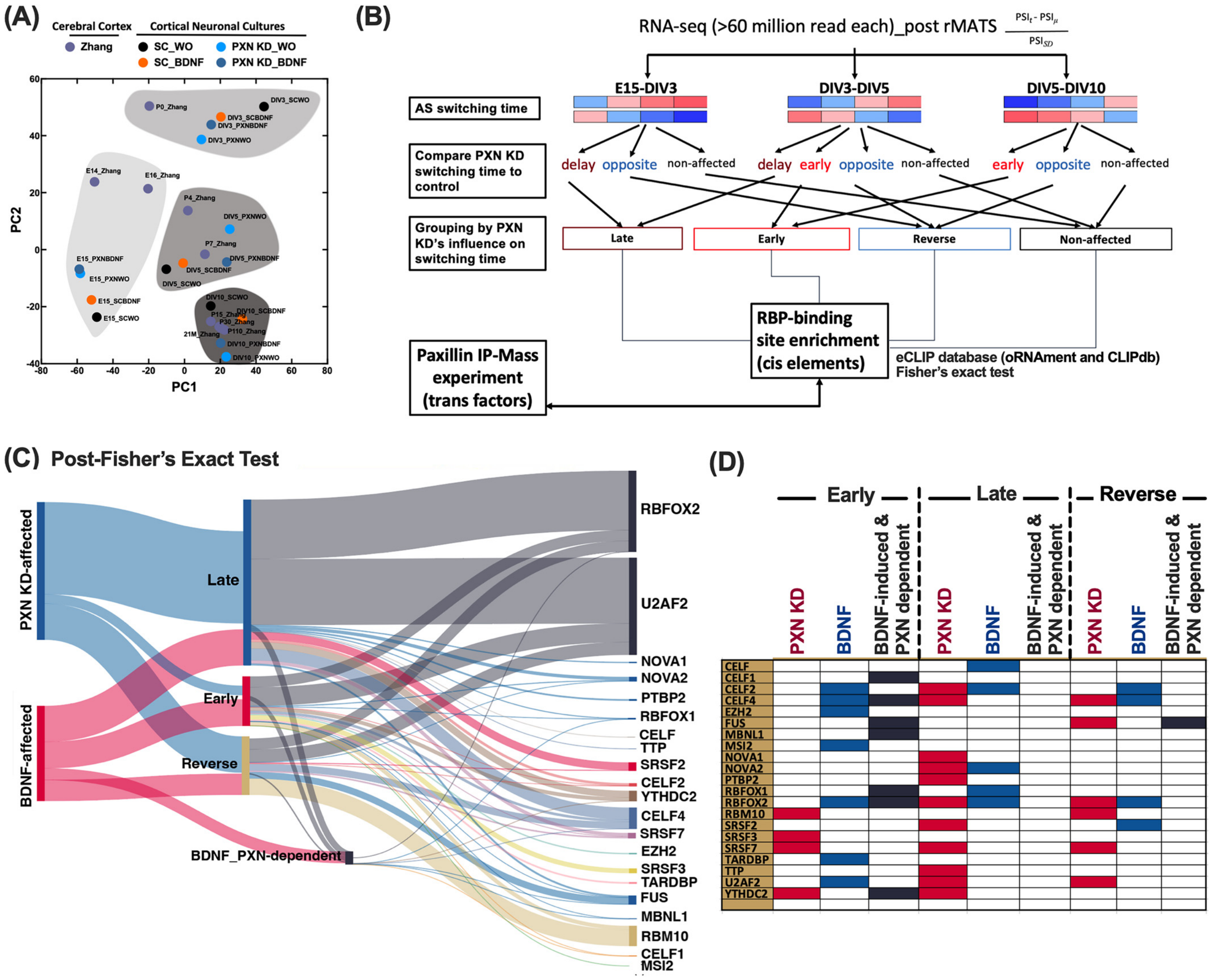

Figure EV5. Identification of alternative splicing events associated with paxillin knockdown and/or BDNF treatment in primary neuronal cultures.

(**A**) PCA analysis of cassette exon profiles. Cassette exon usage patterns from E15 mouse primary neuronal cultures at various DIV were compared with those seen in brain lysates ("Zhang"; dataset from (Weyn-Vanhentenryck et al, 2018) at corresponding postnatal days. Note that DIV3 clustered with P0, DIV5 with P4 and P7, and DIV19 with >P15. (**B**) Analysis pipeline for splicing event identification. Schematic illustrating workflow used to detect cassette exon switching events altered by paxillin knockdown ("PXN KD") and/or BDNF treatment, followed by prediction of trans-acting RNA-binding proteins (RBPs). (**C**) Sankey diagram of splicing time switches and RBPs. Diagram indicating how timing of cassette exon switching is influenced by PXN KD and/or BDNF treatment, and highlighting associated RBPs. Notably, most late-switched cis-elements affected by PXN KD contain U2AF2 and RBFOX2 binding sites. (**D**) Table listing RBPs significantly linked to the PXN KD- and/or BDNF-affected splicing events compared with those in the non-affected group ($p < 0.05$ by Fisher's exact test).

 © The Author(s)
